# Radiological outcomes of surgical techniques for spastic hip in cerebral palsy: a systematic review and meta-analysis

**DOI:** 10.1186/s10195-025-00827-0

**Published:** 2025-02-28

**Authors:** Iman Menbari Oskouie, Alireza Hakiminejad, Amirali Yazdanmehr, Keihan Mostafavi, Asma Mafhoumi, Amir H. Sajedi, Ali Roosta, Alireza Arvin, Ana Presedo, Mohammad Hossein Nabian, Amir Kasaeian

**Affiliations:** 1 Urology Research Center, Tehran University of Medical Sciences, Tehran, Iran; 2 Center for Orthopedic Trans-Disciplinary Applied Research, Tehran University of Medical Sciences, Tehran, Iran; 3 Department of Mechanical Engineering, Sharif University of Technology, Tehran, Iran; 4 Bone and Joint Reconstruction Research Center, Shafa Orthopedic Hospital, Iran University of Medical Sciences, Tehran, Iran; 5 Department of Pediatric Orthopedics, Robert Debré University Hospital, Paris, France; 6 Digestive Oncology Research Center, Digestive Diseases Research Institute, Shariati Hospital, Tehran University of Medical Sciences, Tehran, Iran; 7 Research Center for Chronic Inflammatory Diseases, Shariati Hospital, Tehran University of Medical Sciences, Tehran, Iran; 8 Clinical Research Development Unit, Shariati Hospital, Tehran University of Medical Sciences, Tehran, Iran

**Keywords:** Cerebral Palsy, Spastic Hip, Radiologic Outcomes

## Abstract

**Background:**

In patients with cerebral palsy (CP), spastic hip is a prevalent complication. Various surgical approaches,, including pelvic osteotomy (PO), femoral osteotomy (FO), combined femoral and pelvic osteotomy (CFPO), and soft tissue surgery (STS), have been used to address this problem. This systematic review and meta-analysis was designed to compare the radiologic outcomes of these interventions for spastic hip in patients with CP.

**Methods:**

To identify relevant studies, databases were searched using specific keywords. Initially, duplicates were removed, then the titles and abstracts were screened, followed by a comprehensive full-text review. Data extraction took place from the studies that met the inclusion criteria. Subsequently, a meta-analysis was conducted.

**Results:**

The analysis of 6116 hips from 4546 patients across 81 studies demonstrated that PO significantly enhanced the center–edge angle (CEA), reduced the acetabular index (AI) and migration percentage (MP), and improved the Sharp and Tönnis angles. FO led to a substantial decrease in AI and MP, though CEA did not show a significant change, while CFPO resulted in significant improvements across AI, MP, neck-shaft angle (NSA), CEA, Sharp angle, and Tönnis angle. STS did not show significant changes in AI or CEA, but MP was notably reduced. Tone-decreasing procedures, such as selective dorsal rhizotomy and botulinum toxin injections, did not significantly alter MP, whereas guided growth techniques showed a significant reduction. MP improvements in FO decreased over time, with other radiologic parameters remaining relatively stable as follow-up increased. Age-specific trends indicated that children under 6 years primarily underwent tone-decreasing procedures and STS, while those around 7 years favored FO and guided growth, and older children (over 9 years) more commonly underwent PO, CFPO, or percutaneous osteotomy. Comparative analysis showed PO and percutaneous osteotomy were particularly more effective in reducing MP, with PO also being superior for AI improvement; whereas CFPO provided better outcomes for enhancing CEA. No significant differences were found among surgical methods for improving NSA.

**Conclusions:**

This systematic review and meta-analysis underscores the superior efficacy of PO and CFPO in correcting spastic hip deformity in children with CP. Radiological outcomes demonstrate significant improvements following these procedures. The findings suggest that these approaches are particularly effective for complex cases where procedures such as FO, STS, or TDS may fall short. Future studies should focus on refining surgical protocols and exploring the long-term functional outcomes of these interventions.

**Supplementary Information:**

The online version contains supplementary material available at 10.1186/s10195-025-00827-0.

## Introduction

Cerebral palsy (CP) is a heterogeneous neuromuscular disease that is defined by a group of posture and movement disabilities [1]. The most common motor dysfunction (MD) is spasticity, which is an increase in the resistance and stiffness of muscles when they are stretched, resulting in limited and uneasy movement [2]. One common musculoskeletal abnormality associated with CP is the lateral migration of the femoral head in the acetabulum [3, 4], commonly known as hip displacement, which can range from subluxation to complete dislocation [5, 6]. Limitations caused by hip displacement include pain, impaired walking ability, perineal nursing problems, difficulty with posture and hygiene, skin breakdown, reduced range of motion, and pelvic obliquity [7–9].

Radiological outcome measures provide a quantitative advantage in assessing spasticity and hip deformity in patients with CP. For instance, migration percentage (MP) is closely correlated with MD and the severity of hip adductor spasticity. When MP exceeds 40%, preventive or reconstructive orthopedic surgery is likely necessary [10–12]. Other radiological outcomes, such as center–edge angle (CEA) and acetabular index (AI), are also used to assess hip displacement, though they are considered less reliable than MP. In addition, the femoral neck angle and femoral shaft angle have been found to correlate with a patient’s ability to walk and the severity of hip adductor spasticity [13].

Interventions range from femoral or pelvic osteotomies, soft-tissue releases/tenotomy, and salvage surgeries, to the injection of botulinum toxin A [14, 15]. Varus derotation osteotomy, pelvic osteotomies, releases of hip adductors and flexors, and open reduction of the femoral head, are often classified as reconstructive surgeries; and bony hip salvage procedures, such as valgus osteotomies, are commonly used to correct hip dislocation or subluxation, and prevent skeletal traction [16–18]. However, there is considerable uncertainty within the surgical community regarding the most effective procedure, as current literature is largely composed of case series or limited comparative studies that do not provide a comprehensive overview or systematic review of all potential interventions and radiological outcomes.

This systematic review addressed these gaps by evaluating both the short- and long-term radiologic outcomes of different surgical interventions for spastic hip in children with CP. It provided a thorough comparison of mean differences in MP and other radiological outcomes for each procedure, as well as analyzing age distribution trends related to each surgical method. Understanding these outcomes is critical for improving surgical decision-making, optimizing patient care strategies, and enhancing long-term functional and quality of life outcomes for patients. It was hypothesized that in children with CP and spastic hip deformities, superior radiological outcomes (such as improved migration percentage, center–edge angle, and acetabular index) are achieved through surgical interventions such as pelvic osteotomy and combined femoral and pelvic osteotomy, as compared with other surgeries, such as femoral osteotomy and soft tissue surgery.

## Materials and methods

The design and methodology of this review adhered to the guidelines outlined in the Centre for Reviews and Dissemination (CRD) Guidance for Undertaking Reviews in Healthcare [19].

### Registration and protocol

This study followed the Preferred Reporting Items for Systematic Reviews and Meta-Analyses (PRISMA) guidelines [20]. The study protocol was pre-registered in PROSPERO (CRD42023439598).

### Information Sources

The search strategy was conducted across PubMed, Embase, Scopus, Web of Science, and Cochrane Central in March 2024. Furthermore, gray literature was explored through OpenGrey, the Center for Research Libraries Online Catalogue (CRL), and Open Access Theses and Dissertations (OATD) to identify potentially relevant unpublished studies.

To identify additional eligible studies or reports, a “snowball” search was conducted using citation tracking (both forward and backward) through Scopus for all studies included in this review. As a final step, the reference lists of related reviews identified through our search were examined to determine if any other potentially relevant studies could be included.

### Search

The search strategy was designed and reported in accordance with the Preferred Reporting Items for Systematic Reviews and Meta-Analyses Literature Search Extension (PRISMA-S) guidelines [21]. No restrictions or search filters were applied. Free-text terms and keywords were identified through the MeSH Browser [22] and the PubMed PubReMiner word frequency analysis tool [23]. The search strategy was reviewed by IMO following the Peer Review of Electronic Search Strategies (PRESS) guidelines [24]. A detailed description of the search strategy can be found in Appendix A.

### Eligibility criteria

*Population*: all CP patients under 18 years old with spastic hip.

*Intervention*: surgical methods used in spastic hip surgery including pelvic osteotomy (PO), femur osteotomy (FO), a combination of the femur and pelvic osteotomy (CFPO), soft tissue surgery (STS, such as tenotomies), tone-decreasing surgery (TDS, such as botulinum toxin injections), open reduction (OR), guided growth surgery (GGS), and percutaneous osteotomy (PCO).

*Comparator*: comparison of each patient’s radiological outcomes and parameters before and after surgery.

*Study Design*: clinical trials and case series.

*Outcome*: radiological parameters such as migration percentage (MP), neck shaft angle (NSA), acetabular index (AI), center–edge angle (CEA), Sharp’s angle (ShA), and Tönnis angle (TA).

### Study selection

The citations from literature searches were imported into EndNote [25]. Duplicates were identified and manually removed. The titles and abstracts of the initial 50 records were independently screened by six reviewers working in pairs. Inter-rater reliability was assessed using Cohen’s kappa, yielding a score of 0.87, which is indicative of almost perfect agreement. The same reviewers in the previous teams, independently assessed the titles and abstracts. In case of any disagreements, the reviewers discussed the issues; if they couldn’t reach a consensus, a third reviewer intervened to make the final decision. Subsequently, full texts of all potentially eligible records were obtained, and the same teams screened these studies for inclusion. A study was included if both reviewers agreed it met the criteria. Disagreements were again discussed, with a third reviewer consulted when necessary [26].

### Data collection process

A data extraction spreadsheet was created using Google Sheets. After a meeting to discuss and resolve discrepancies, the reviewers independently extracted data from eligible studies. The extracted data were then compared, and any differences were resolved through additional discussion. In cases where data were missing or information was unclear, efforts were made to contact the study authors [27].

### Data extraction

The extracted data included:

- Study identifiers and design: study title, first author, publication year, and study design.
- Characteristics of the record: intervention type, sample size (patients and hip), gender, age, follow-up duration, ambulatory status (Gross Motor Function Classification System [GMFCS]), and radiologic outcomes (as mentioned above).

### Risk of bias

The risk of bias and methodological validity of all included studies were assessed by two authors using the JBI Critical Appraisal Checklist for Qualitative Research standardized by the Joanna Briggs Institute (JBI) [28]. All manuscripts selected for inclusion in this study underwent rigorous evaluation on the basis of study type.

The quality of evidence was evaluated using the Grading of Recommendations Assessment, Development, and Evaluation (GRADE) approach. Five factors were considered for each outcome: study limitations, publication bias, indirectness of evidence, inconsistency of results, and imprecision, with the latter assessed on the basis on the results from the Trial Sequential Analysis (TSA)[29, 30].

### Publication bias assessment

Publication bias was defined as the failure to publish study findings on the basis of their direction or strength. The primary reasons for this bias included journal rejection by editors and reviewers, as well as a lack of motivation to write, despite the study being conducted [31].

Consequently, if publication bias occurred in a meta-analysis, the synthetic effect estimates might have been exaggerated in a favorable direction. Funnel plots were employed in systematic reviews and meta-analyses to determine whether the included studies were biased or systematically heterogeneous. Egger’s regression test was utilized to quantify funnel plot asymmetry, and the trim-and-fill method was applied to correct funnel plot asymmetry in such instances.

### Statistical analysis

R version 4 [32], was employed, and the SCSmeta function [33] was used to carry out the statistical analyses. For the meta-analysis, the Hartung–Knapp adjustment was applied to the random effects model. To evaluate heterogeneity, H statistics, Cochran’s Q test, and Higgins and Thompson’s I^2^ statistics were utilized [34].

### Sensitivity analysis

To assess the reliability of the study, a sensitivity analysis was conducted. This involved evaluating the stability across different modalities. Furthermore, to verify the consistency of the reported results, multiple sensitivity analyses were performed by sequentially removing one study at a time from each meta-analysis, a process known as leave-one-out meta-analysis [35].

## Results

**Fig. 1:**
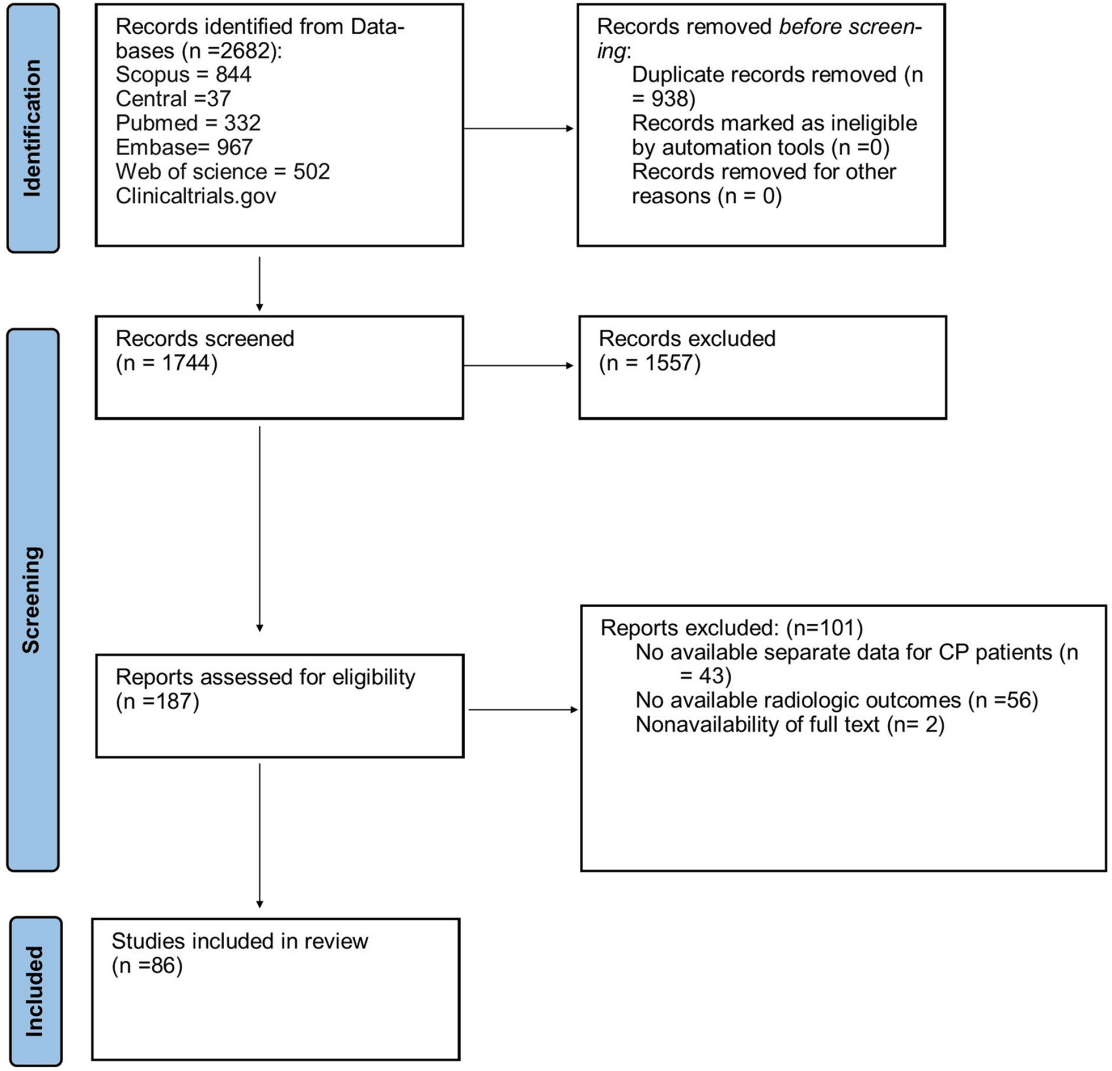
A PRISMA flow diagram of the systematic review, detailing the database searches, the number of abstracts screened, and the full texts reviewed

The initial search yielded 2682 papers from electronic databases. After manually removing 938 duplicates, 1744 studies remained. Of these, 1557 were excluded following title and abstract screening, leaving 187 articles for full-text review based on eligibility criteria. Finally, 86 studies were selected for qualitative and quantitative analysis. All 86 studies were included in the qualitative synthesis (systematic review), while those with sufficient data were used for quantitative synthesis (meta-analysis) (Fig. 1). No relevant studies were found in gray literature electronic databases.

### Characteristics of the included studies

**Fig. 2:**
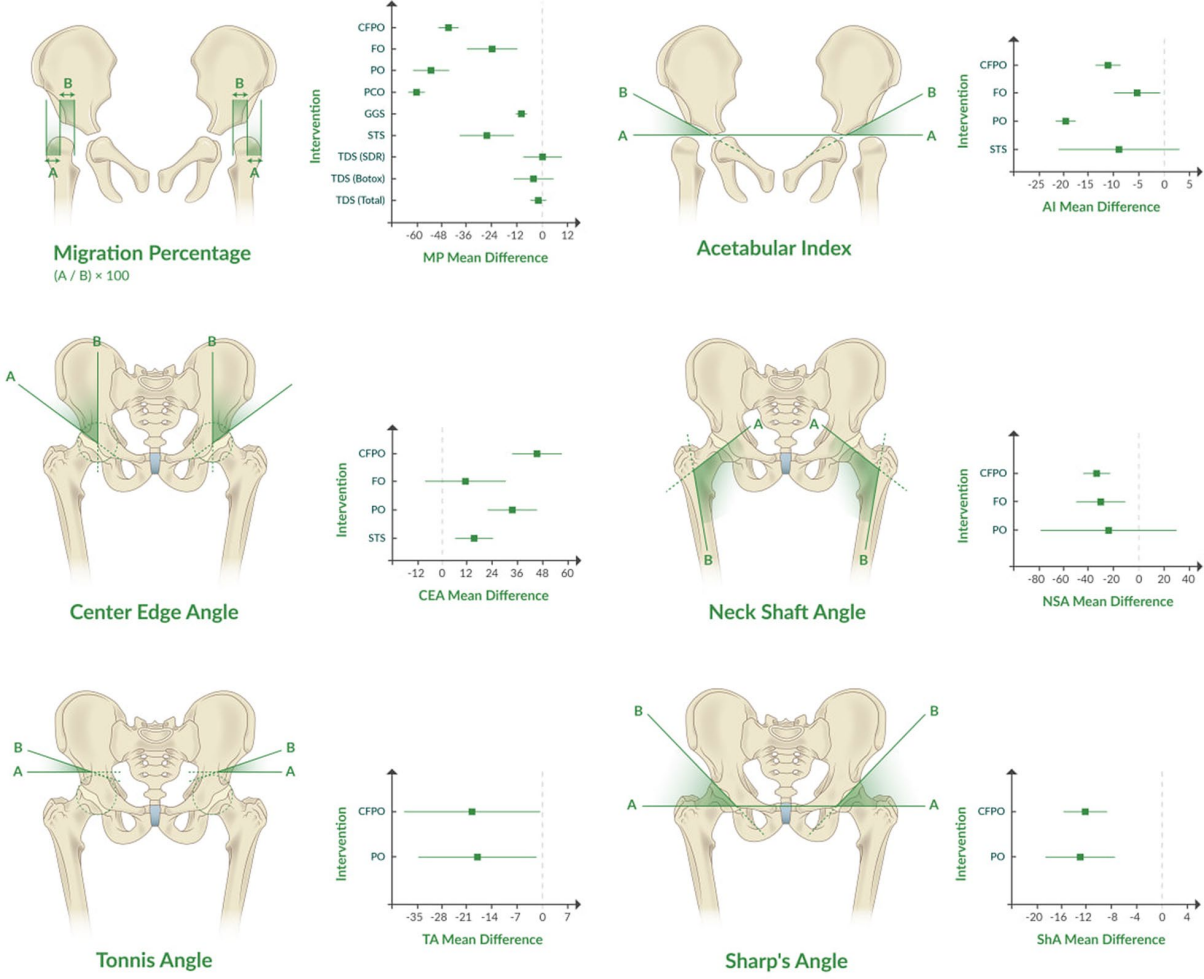
Schematic representation of the mean difference (95% CI) for each angle in various interventions

The studies included were conducted between 1964 and 2023, and involved 4546 patients and 6116 hips. Eight surgical procedures were examined for their impact on radiologic outcomes in patients with CP with spastic hip deformity: PO (*n* = 16) [36–51], FO (*n* = 13)[18, 52–63], CFPO (*n* = 26) [42, 47, 51, 62, 64–85], STS (*n* = 14) [86–99], TDS (*n* = 9)[100–108], OR (*n* = 6)[109–114], GGS (*n* = 3)[115–117], and PCO (*n* = 3)[118–120]. The included studies involved 4546 patients and 6116 hips. Patient ages ranged from 3.1 to 16 years, and follow-up durations spanned 9–153.6 months. Detailed study characteristics are provided in Table 1, with Table 2 and Fig. 2 summarizing meta-analysis results. Sufficient data for meta-analysis led to 22 forest plots (Appendix B), with Baujot plots in Appendix C illustrating “overall heterogeneity contribution” and “influence on pooled results.”

**Table 1:** Characteristics of included studies

Author	Year	Patient (*n*)	Feet (*n*)	M/F	Age mean ± SD (years)	Follow-up mean ± SD (months)	Pre-op GMFCS	Radiographic outcomes	Complications (*n*)
*Pelvic osteotomy*									
Robb, J.E. [1]	2006	47	52	N/A	14 ± 4.5	48 ± 35.25	N/A	MP, NSA	• Fracture of the acetabulum [5] • AVN [1]
Brooks, R.A [2]	2000	10	16	N/A	9.7 ± 1.725	N/A	N/A	NSA	• No complications
Roposch, A [3]	2005	32	41	18/14	9.5 ± 2.9	63.6 ± 29.1	N/A	AI, MP, Sharp’s Angle	• Moderate sclerosis of the femoral head and acetabulum [3] • Painful hip during follow-up [1] • Redislocation [1] • Unstable hip at follow-up [1]
Georgiadis, A.G [4]	2018	24	24	17/7	16.6 ± 4.025	28.8 ± N/A	I and II: 15; III: 4; IV and V: 5	CEA, MP, Tönnis angle,	• The CPHCS worsened [2] • AVN [1] • Suprafascial wound infection [1] • Blood transfusion [3] • UTI [1]
Schlemmer, T [5]	2022	37	43	20/17	15.17 ± 2.78	162 ± 68.4	I: 1; II: 1; III: 2; IV: 17; V: 22	MP, Sharp’s Angle	• Hip pain [3] • Deteriorated in GMFCS level [4]
Fucs, P.M.D.M [6]	2006	58	78	30/28	7.59 ± 3.225	53.76 ± 23	N/A	AI, MP	• Unsatisfactory hip functionally [1]
Miller, ML [7]	2021	14	16	N/A	17.7 ± 3.7	39.6 ± 3.15	I: 7; II: 7; III: 1; IV: 1	Tönnis angle, CEA	• Problematic lower extremity uncontrolled posturing [1] • Marked acetabular deficiency and soft tissue hip abductor and flexor contractures [1] • Spontaneous anterior wound drainage [1] • Superficial wound dehiscence [1] • Bilateral (staged) [1] • Grade IV heterotopic ossification [1]
Karlen, J.W. [8]	2009	22	26	5/17	3.1 ± 2.125	51 ± 14.75	N/A	AI, CEA	• Graft dislodgement [1] • Collapse of the graft [1] • Recurrent subluxation [1] • Asymptomatic lateralization of the femoral head [2]
Karlen, J.W. [8]	2009	22	24	15/7	6.3 ± 2.5	56 ± 17	N/A	AI, CEA, MP	
Bor, N. [9]	2020	25	27	9/16	5 ± 2.9375	N/A	N/A	AI, CEA	NR
Rebello, G [10]	2009	26	31	15/11	9.7 ± N/A	36	N/A	AI, CEA, MP	• Nonunion of the pubic ramus and sciatic nerve palsy [1] • Persistent hip subluxation [2] • Persistent dysplasia [1] • No premature closure of the triradiate cartilage
Sung, K.H [11]	2018	110	150	68/42	8.7 ± 2.4	34.8 ± 31.2	III: 17; IV: 39; V: 54	AI, MP, NSA	• No case of graft-related complications
Cottrill, E.J. [12]	2019	38	55	22/16	10.2 ± 5.5	N/A	I: 0; II: 3; III: 8; IV: 11; V: 16	MP	• Wound dehiscence [2] • Wound-related infections [5]
Osterkamp, J. [13]	1988	11	12	N/A	12.5 ± N/A	40.3 ± N/A	N/A	AI, CEA	• Dislocated the involved hip [2] • Severe oblique obliquity and scoliosis [1] • Fixed abduction contracture [1] • Superficial wound infection [1] • Supracondylar fracture [1]
Dietz, F.R. [14]	1995	23	24	N/A	12.5 ± 3.475	86 ± 31.24	N/A	MP, CEA, Sharp’s angle	• Cast sores [3] • Superficial wound infection [1] • Stress ulcers [2] • Intraoperative midshaft femur fracture [1] • Deep femoral vein thrombosis [1]
Osebold, W.R. [15]	2002	10	10	N/A	11.5 ± 3.4175	128 ± 16.5	N/A	AI, CEA, Sharp’s angle	NR
Chen, K [16]	2022	5	5	11/8	18 ± 6	44 ± 28.3	I: 0; II: 8; III: 8; IV: 2; V: 1	MP, CEA, Sharp’s angle, NSA	• Re-subluxation [7] • Lateral femoral cutaneous nerve impairment [4] • No AVN, complete redislocation, surgical site infection, sciatic nerve impairment, or pressure sore
*Femor osteotomy*									
Shore, B.J. [17]	2016	54	54	N/A	6.5 ± 3.1	93.6 ± 18	I: 5; II: 15; III: 8; IV: 14; V: 13	AI, CEA, MP, NSA	NR
Chang, F.M. [18]	2016	87	174	N/A	4.6 ± 1.6	61.2 ± 26.4	I, II, and III: 26; IV and V: 61	Acetabular depth ratio	NR
Al-Ghadir, M. [19]	2009	28	36	22/14	9.4 ± 3.2	50 ± N/A	N/A	AI, CEA, MP, NSA	• Required revision procedures [4] • No delayed unions, AVN of the femoral head, or postoperative infections • Asymptomatic heterotrophic ossification of the lesser trochanter [1]
Settecerri, J.J. [20]	2000	89	130	55/44	7.7 ± 2.9	64.6 ± 38.72	N/A	CEA, MP, NSA	• Dislocation after surgery [12] • Painful after surgery [12] • Death within 2 years after surgery [3]
Schmale, GA. [21]	2006	16/6	44	38/22	4 ± N/A	114 ± 45	N/A	AI, CEA, MP, NSA, acetabular angle	• AVN of the femoral head, nonunion at a repeated femoral osteotomy site, and painful ectopic bone formation at the lesser trochanter [1]
Wagner, P. [22]	2022	158	158	96/62	5.3 ± 2.62	> 36	III: 16; IV: 57; V: 85	MP	NR
Rutz, E. [23]	2012	11	11	6/5	11.1 ± 2.7	78 ± 25.25	II: 10; III: 1	MP, PO	• Superficial wound infections [3]
Davids, J.R [24]	2013	75	137	42/33	7 ± 2.67	139 ± 43	N/A	NSA, HAS	• Delayed union [5] • Osteonecrosis of the femoral head [6] • Hip dislocation [4] • Late fractures of the femur [1]
Mazur, J.M. [25]	2004	44	75	18/26	8 ± 4	N/A	N/A	NSA	NR
Tomov, A.D. [26]	2020	28	44	N/A	6.98 ± 2.2	36 ± N/A	IV: 30; V: 19	AI, CEA, MP	NR
Larsson, M. [27]	2012	24	24	19/5	7.6 ± 2.6	60 ± N/A	III: 1; IV: 4; V: 19	MP	NR
Park, H. [28]	2020	72	144	48/24	6.2 ± 2.25	84 ± 42	IV: 40; V: 32	AI, MP, NSA, HSA	• Re-subluxations or dislocations [31] • Re-dislocations occurred in all cohorts [6] • Radiological signs of AVN [38] • Superficial cast ulcers [7] • Superficial wound infections [6]
Huh, K. [29]	2011	75	92	45/30	7 ± 2.5	55.2 ± 26.1	I: 0; II: 4; III: 19, IV:31, V:38	NSA, CEA, MP, AI, Sharp’s angle	NR
*Combined femoral and pelvic osteotomy*									
Reidy, K. [30]	2016	40	57	20/20	8.9 ± 3.05	65.4 ± 24.75	I: 2; II: 5; III: 4; IV: 4; V: 25	MP, NSA	NR
Cottrill, EJ [12]	2019	38	55	22/16	10.2 ± 6	20.4 ± 7.5	II: 3; III: 8; IV: 11; V: 16	MP	• Wound dehiscence [2] • Wound-related infections. [5]
Zenios, M [31]	2012	18	20	9/9	7.3 ± 2.52	135.84 ± 8.4	N/A	CEA, MP, NSA, Sharp’s angle	• Severe scoliosis [11], which invariably compromised respiratory function as they entered adolescence. One patient died due to respiratory failure secondary to scoliosis
Debnath, U.K. [32]	2006	11	12	N/A	14.1 ± 2.17	157.2 ± 28.5	N/A	CEA, MP, NSA, Sharp’s angle	• Troublesome gastrooesophageal reflux [1] • No AVN of the femoral head • No wound infections, problems with the sciatic nerve or pressure sores
Kim, H.T. [33]	2012	23	32	13/10	8.6 ± 2.75	28.1 ± 8.25	III: 1; IV: 9; V: 13	AI, CEA, MP	• AVN of the femoral head [2]
Westberry, D.E. [34]	2023	16	16	9/7	8.2 ± 2.8	38.4 ± 18.3	IV: 3; V: 13	AI, MP, NSA	• Osteonecrosis [2] • PICU admission [2] • Medical readmission [3] • Dislocation [1]
Westberry, D.E. [34]	2023	44	88	27/17	7.5 ± 1.9	66 ± 37.85	IV: 18; V: 26	AI, MP, NSA	
Westberry, D.E. [34]	2023	39	39	23/16	7 ± 1.5	61.2 ± 33.6	IV: 7; V: 32	AI, MP, NSA	
Westberry, D.E. [34]	2023	39	39	23/16	7 ± 1.5	61.2 ± 33.6	IV: 7; V: 32	AI, MP, NSA	
Braatz, F. [35]	2016	72	72	45/27	7.6 ± 2.9	92.4 ± 20.7	II: 7; III: 23; IV: 26; V: 16	CEA, MP	NR
Inan, M. [36]	2007	27	33	11/16	15 ± 1.75	39 ± 16.5	N/A	MP, NSA, Sharp’s angle,	• Superficial skin breakdown [2] • Deep wound breakdown over the blade plate [1] • Deep wound infection at the femoral osteotomy site [3] • No chronic osteomyelitis or septic arthritis
Ma, M. [37]	2022	45	58	35/23	10.8 ± 3.1	103.2 ± 21.6	V:34 IV:24	AI, CEA, MP, NSA, Sharp’s angle	NR
Rutz, E. [38]	2015	121	168	101/2	11.3 ± 3.7	87.6 ± 55.2	II: 7; III: 4; IV: 29; V: 81	MP	• Septic arthritis [1] • Recurrent hip dislocations [2] • Radiographic progression of the femoral head deformity [11] • Acetabular necrosis [1] • Heterotopic ossification [1] • Required revision [1] • Supracondylar femoral shaft fracture [3] • Chest infection [16] • Aspiration [2] • Decubitus ulceration [12] • Deterioration of a seizure disorder [3] • UTI [3]
Wen, J. [39]	2020	23	35	15/8	8.3 ± 1.7	38 ± 9	I and II: 23	AI, MP, NSA	• AVN of the femoral head [1] • Mild inguinal incision infection [2] • Pressure ulcer on the heel [3]
Kapp, J.E. [40]	2018	42	45	24/18	7.7 ± 2.7	12 ± N/A	IV: 30; V: 12	AI, MP, NSA	• Deep wound infection [2] • No cases of AVN, hardware failure, or nonunion within the follow-up period
Krebs, A. [41]	2008	54	66	N/A	8.6 ± 4.1	57.6 ± 32.2	I–III: 10; IV and V: 54	AI, CEA, MP	• Superficial wound [1] • Increased sensitivity to pain [1] • Hematoma in the adductor region [1] • Moving of a K-wire [1]
Kamisan, N. [42]	2020	60	102	23/37	7.3 ± 2.2	38.22 ± 13.5	N/A	MP, PO	• Sub-trochanteric fracture [1] • No implant-related infection or wound dehiscence
Kamisan, N. [42]	2020	18	18	9/9	7.3 ± 2.2	38.22 ± 13.5	III: 1; IV: 1; V: 16	MP, PO	
Zhang, S.R. [43]	2014	34	58	21/13	5 ± 3	62.5 ± 36.25	IV: 21; V: 13	MP	• Reconstructive hip surgeries failed [15] • Re-subluxation [6]
Bayusentono, S. [44]	2014	76	144	57/19	8.5 ± 2.3	58.8 ± 28.8	II and III: 12; IV: 30; V: 34	MP, NSA, HSA	NR
Chen, B.P.J. [45]	2023	140	253	79/61	11.7 ± 3.3	54 ± 27.6	I: 5; II: 18; III: 32; IV: 81; V: 253	MP	NR
Min, J.J. [46]	2021	108	214	76/32	9.4 ± 3.2	62.4 ± 38.4	II: 1; III: 8; IV: 50; V: 49	MP, NSA	NR
Khalife, R. [47]	2010	50	89	33/17	7.4 ± 2.75	72 ± N/A	II: 13; III: 14; IV and V: 23	MP, NSA	• AVN [33]
Huh, K. [29]	2011	75	24	45/30	7 ± 2.5	55.2 ± 26.1	III: 1; IV: 5; V: 18	AI, CEA, MP, NSA, Sharp’s angle	NR
Oto, M. [48]	2018	22	25	9/13	8.7 ± 3.5	36.1 ± 10.4	III: 7; IV: 9; V: 6	AI, MP, NSA	• Hematoma [1] • Failure of femoral osteotomy fixation [1]
Refakis, C.A. [49]	2018	11	15	N/A	1.6 ± 0.66	40 ± 16	N/A	AI, MP	• Buckle fractures of the femur [2] • One symptomatic implant [1] • Unplanned surgery [1]
Canavese, F. [50]	2010	27	27	13/14	20.4 ± 2.75	60 ± N/A	III: 2; IV: 5; V: 20	MP, acetabular angle, PO	NR
Abousamra, O. [51]	2016	12	12	10/2	14 ± 3	48 ± 21	I: 4; II: 8	MP, NSA, Sharp’s angle, PO	• Limb-length discrepancy [7]
Chen, K. [16]	2022	14	16	11/8	15 ± 4	41.5 ± 17.2	II: 8; III: 8; IV: 2; V: 1	CEA, MP, NSA, Sharp’s angle	• Re-subluxation [7] • Lateral femoral cutaneous nerve impairment [4] • No patient developed AVN, complete re-dislocation, surgical site infection, sciatic nerve impairment, or pressure sore
Miller, M.L. [7]	2021	14	16	N/A	17.7 ± 3.75	39.6 ± 12.9	I: 7; II: 7; III: 1; IV: 1	CEA, Tönnis angle	• Problematic lower extremity uncontrolled posturing [1] • Marked acetabular deficiency and soft tissue hip abductor and flexor contractures [1] • Spontaneous anterior wound drainage [1] • Superficial wound dehiscence [1] • Bilateral (staged) [1] • Grade IV heterotopic ossification [1]
*Soft tissue surgery*									
Cobeljic, G. [52]	2009	20	20	8/12	6.65 ± 1.75	105.6 ± 39	N/A	MP	NR
Cobeljic, G. [52]	2009	22	22	13/9	5.6 ± 1.5	99.6 ± 42			
Martinsson, C. [53]	2021	269	269	151/118	4 ± 3.85	41 ± 24.6	IV: 7; V: 9	MP	NR
Wheeler, M.E. [54]	1984	25	41	N/A	5.75 ± 2	44.4 ± 24	I: 1; II: 6; III: 1; IV: 3; V: 14	AI, CEA	NR
Owers, K.L. [55]	2001	30	60	12/18	7.7 ± 2.27	36 ± 19	N/A	CEA, MP	• Supracondylar fractures of the femur [3] • Trochanteric bursitis [1] • Sinus over a plate [1] • Plaster sore [1]
Noonan, K.J. [56]	2000	35	35	16/19	5.5 ± N/A	50.4 ± N/A	N/A	AI, CEA, MP, NSA, Sharp’s angle	• Hardware failure [1] • Superficial cast pressure sore [1] • Windswept hip deformities [10]
Bozinovski, Z. [57]	2008	11	22	5/6	8.5 ± N/A	48 ± N/A	N/A	MP	NR
Yngve, D. [58]	2022	2	4	1/1	4.1 ± 0.15	72 ± N/A	IV:1 V:1	MP	NR
Pap, K. [59]	2005	41	38	N/A	4.9 ± 0.5	36 ± N/A	N/A	CEA, MP	NR
Pap, K. [59]	2005	41	38	N/A	4.9 ± 2	36 ± N/A	N/A	CEA, MP	
Ha, M. [60]	2018	27	43	N/A	4.83 ± 1.81	65 ± 32.75	III: 1; IV: 1; V: 25	AI, CEA, MP	NR
Heimke, B. [61]	2011	71	71	42/29	7 ± 2.25	153.6 ± 78	I: 12; II: 11; III: 12; IV: 20; V: 16	MP	NR
Presedo, A. [62]	2005	65	65	37/28	4.4 ± 1.7	129.6 ± 15	N/A	MP	NR
Bos, C.F.A. [63]	1987	10	10	2/8	9.35 ± 0.87	42 ± N/A	N/A	AI, NSA	NR
Terjesen, T. [64]	2017	37	37	21/16	5 ± 1.1	87.6 ± 14.1	III: 9; IV: 10; V: 18	AI, MP, PO	NR
Shea, J. [65]	2020	127	127	68/59	9 ± 4	N/A	IV: 59; V: 68	AI, MP, PO	• At least 1 major complication [29] • At least 1 minor complication [115]
*Tone decreasing surgery*									
Khot, A. [66]	2008	16	16	9/7	2–6	24 ± N/A	III: 5; IV: 11	MP	• No surgical complications, no groin hematomas or wound infections
Floeter, N. [67]	2014	33	33	19/14	6.7 ± 20.27	18 ± 4.25	I: 11; II: 16; III: 6	AI, MP	• No deterioration of hip geometry
Heim, R.C. [68]	1995	45	90	22/23	5.08 ± 1.75	20 ± 10.75	N/A	MP	NR
Yang, E.J. [69]	2008	60	130	34/31	3.32 ± 1.08	22.6 ± 7.9	I: 3; II: 17; III: 18; IV: 11; V: 16	MP	NR
Yang, E.J. [69]	2008	65	120	51/9	3.36 ± 0.88	22.5 ± 10.9	I: 2; II: 12; III: 21; IV: 18; V: 7	MP	
Jung, N.H. [70]	2011	27	27	18/9	5.2 ± 1.96	24 ± N/A	I: 1; II: 3; III: 3; IV: 12; V: 8	MP	NR
Jung, N.H. [70]	2011	27	27	18/9	5.2 ± 1.96	24 ± N/A	I: 1; II: 3; III: 3; IV: 12; V: 8	MP	
Park, E.S. [71]	2014	25	49	16/33	4.51 ± 1.37	18.46 ± 18.46	III: 18; IV: 17; V: 14	MP	NR
Placzek, R. [72]	2004	5	6	N/A	6.3 ± 0.67	24 ± 1.5	IV: 5	MP	NR
Kim, D.S. [73]	2002	200	200	N/A	6 ± 4.2	48 ± 24	N/A	MP	• Postoperative hypotonia (166) • Voiding difficulties [20] • Spinal deformity [12] • Temporary sensory [15] • Aspiration pneumonia [2] • Aggravation of involuntary movement of the arm [2]
Willoughby, K. [74]	2012	46	46	31/15	3 ± N/A	130 ± 15.75	II: 3; III: 11; IV: 20; V: 12	MP	NR
*Open reduction*									
Deignan, B.J. [75]	2020	44	61	18/26	7.08 ± 3.5	N/A	IV and V: 44	AI, CEA, NSA	• Hardware removal for pain [4] • No femur fracture below the rod • No infections or cases of AVN
Phillips, L. [76]	2017	47	70	31/16	8.82 ± 3.57	32.76 ± 17.16	II: 1; III: 3; IV: 17; V: 26	AI, MP, NSA, PO	• AVN [19] • Fragility fractures [3] • Pressure sores [3] • Infection [1]
Zhou, L. [77]	2015	25	45	13/12	7.75 ± 3.5	9 ± 4	II: 4; III: 1; IV: 5; V: 11; TD: 4	MP, NSA	• Infection [1]
Gavrankapetanovic, I. [78]	2007	31	45	8/23	5.2 ± 3.5	115.2 ± 75	N/A	M	• Superficial wound infection [2] • Bilateral supracondylar femur fracture [2] • Redislocation [3] • Hip subluxation [1] • Persisting pain [1]
Cobanoglu, M. [79]	2018	30	45	16/14	8.7 ± 3.25	57 ± 27	I: 1; II: 4; III: 5; IV: 9; V: 11	MP	NR
Gamble, J.G. [80]	1990	24	31	14/10	N/A	60	N/A	CEA, MP	NR
*Guided growth surgery*									
Hsieh, H.C. [81]	2019	24	48	17/7	8 ± 1.75	50 ± 11.75	I: 3; II: 4; III: 7; IV: 7; V: 3	AI, MP, HSA, HEA	• The proximal femoral physis grew off the transphyseal screw [21] • Replacement [15] • There was no wound infection or other surgical complications • Reconstructive surgery in eight hips
Lee, W.C. [82]	2016	9	13	4/9	6.2 ± 1.5	45.6 ± N/A	IV and V: 9	MP, HSA	• No wound infection or other surgical morbidities
Portinaro, N. [83]	2019	28	56	17/11	4–11	60 ± N/A	III: 7; IV: 9; V: 12	AI, MP, NSA	• No AVN, chondrolysis, wound infection, femoral neck fracture
*Percutaneous osteotomy*									
Canavese, F. [84]	2013	24	30	15/9	9.5 ± 2.8	35.9 ± 26.7	IV:14 V:10	MP, AI	• AVN [3] • Bone graft dislodgement [1] • Hip dislocation [1] • Pathological fracture [2] • Postoperative pain [4]
Canavese, F. [85]	2014	19	25	11/8	10.2 ± N/A	24 ± N/A	IV: 13; V: 6	MP, AI	• Pain ≥ 6 months [2] • Femoral fracture [2] • Death [1]
Canavese, F. [85]	2014	21	22	13/8	8.5 ± N/A	24 ± N/A	IV:17 V:4	MP, AI	• Pain ≥ 6 months [2] • Graft migration [1] • Recurrent dislocation [1] • Necrosis of the femoral epiphysis [3] • Femoral fracture [1]
Canavese, F. [86]	2017	54	64	34/20	9.1 ± 3.3	43.9 ± 19.5	IV: 38; V: 16	MP	• Recurrent dislocation [1] • Bone graft dislodgment [1] • AVN [4] • Pain > 6–12 months [4]

*AI* acetabular index, *CEA* center edge angle, *MP* migration percentage, *NSA* neck shaft angle, *HSA* head shaft angle, *PO* pelvic obliquity, *AVN* avascular necrosis, *UTI* urinary tract infection

**Table 2:** Meta-analysis of radiologic outcomes in various interventions

Type of Intervention		Radiographic outcome	Studies (*n*)	Feet (*n*)	Mean age (95% CI)	Mean pre-op (95% CI)	Mean post-op (95% CI)	Mean difference (95% CI)	*p*-Value	*I*^2^ (%)	Egger’s test *p*-value	Grade
Tone decreasing surgery	Total	MP	8	698	5.03 (4.23–5.84)	30.76 (23.75–37.77)	28.48 (23.37–33.59)	−1.90 (−5.73, 1.93)	0.292	80.4	0.097	Low ^b,d^ ⨁⨁◯◯
	Botox	MP	4	206	4.98 (3.63–6.34)	33.40 (21.14–45.66)	29.52 (22.80–36.23)	−4.20 (−13.68, 5.29)	0.286	76.1	0.464	low ^b,d^ ⨁⨁◯◯
	SDR	MP	3	323	5.88 (3.86–7.89)	22.15 (3.18–41.12)	22.01 (10.54–33.49)	0.12 (−9.08, 9.32)	0.962	85.7	0.294	low ^b,d^ ⨁⨁◯◯
Soft Tissue Surgery		AI	5	256	6.88 (4.25–9.52)	26.72 (23.80–29.64)	17.27 (6.53–29.01)	−9.00 (−21.00, 3.00)	0.106	92.6	0.634	low ^b,d^ ⨁⨁◯◯
		CEA	5	260	5.58 (4.43–6.74)	2.90 (−7.01- 12.82)	18.47 (9.98–26.96)	16.10 (6.59–25.61)	0.007	89.0	0.374	Moderate^b^ ⨁⨁⨁◯
		MP	12	851	5.83 (4.89–6.76)	50.39 (37.72–63.06)	23.62 (16.43–30.81)	−26.44 (−39.41, −13.48)	< 0.001	99.5	0.370	Moderate^b^ ⨁⨁⨁◯
Guided growth surgery		MP	3	117	7.10 (4.89–9.32)	41.28 (17.39–65.18)	31.87 (3.64–60.09)	−9.97 (−12.65, −7.3)	0.004	0	0.095	High ⨁⨁⨁⨁
Percutaneous osteotomy		MP	3	141	9.31 (8.31–10.32)	66.74 (63.19–70.29)	6.81 (3.86–9.75)	−59.94 (−63.90, −55.97)	< 0.001	0	0.604	High ⨁⨁⨁⨁
Pelvic osteotomy		AI	6	334	6.71 (4.20–9.23)	36.12 (29.77–42.48)	17.21 (9.77–24.64)	−19.57(−21.56, −17.59)	< 0.001	57.9	0.809	High ⨁⨁⨁⨁
		CEA	8	149	13.51 (8.08–18.95)	−5.13 (−16.25 to 5.98)	30.47(23.43–37.52)	35.27 (22.88, 47.66)	< 0.001	88.1	0.018*	Low ^b,e^ ⨁⨁◯◯
		MP	11	527	11.70 (8.83–14.57)	65.62(57.19–74.05)	12.16 (8.51–15.81)	−53.06 (−61.69, −44.43)	< 0.001	92.1	0.434	Moderate^b^ ⨁⨁⨁◯
		NSA	3	634	11.94 (0–25.39)	150.9 (120.5–181.4)	127.1 (103.7–150.4)	−23.86 (−77.58, 29.86)	0.196	97.9	0.746	Low ^b,d^ ⨁⨁◯◯
		ShA	4	59	12.19 (8.35–16.03)	50.51 (49.08–51.94)	37.33 (33.15–41.50)	−13.02 (−18.54, −7.50)	0.005	79.8	0.366	Moderate^b^ ⨁⨁⨁◯
		TA	3	45	17.17 (15.11–19.23)	26.16 (15.62–36.70)	8.43 (2.18–14.68)	−17.96 (−34.32, −1.61)	0.042	84.9	0.686	Moderate^b^ ⨁⨁⨁◯
Femoral osteotomy		AI	5	370	7.16 (5.64–8.68)	22.87 (17.45–28.30)	17.40 (10.11–24.70)	−5.39 (−10.00, −0.77)	0.032	88.8	0.275	Moderate^b^ ⨁⨁⨁◯
		CEA	3	190	6.86 (4.00–9.71)	6.77 (−9.03- 22.57)	18.40 (8.21–28.59)	11.75 (−8.55, 32.06)	0.130	94.7	0.408	Low ^b,d^ ⨁⨁◯◯
		MP	10	737	7.43 (6.14–8.74)	49.07 (38.22–59.93)	25.07 (18.86–31.29)	−23.91 (−35.97, −11.85)	0.002	98.2	0.026*	Low ^b,e^ ⨁⨁◯◯
		NSA	8	712	7.34 (6.37–8.30)	151.4 (143.4–159.4)	121.2 (94.7–147.4)	−30.09 (−49.45, −10.73)	0.008	99.6	0.008*	Low ^b,e^ ⨁⨁◯◯
Combined femoral and pelvic osteotomy		AI	11	598	8.47 (6.18–10.76)	33.07 (29.63–36.51)	22.22 (17.63–26.80)	−11.18 (−13.68, −8.68)	< 0.001	92.2	0.037*	Low ^b,e^ ⨁⨁◯◯
		CEA	9	316	10.68 (7.69–13.67)	−19.49 (−32.98, −6.00)	27.94 (21.03–34.84)	47.65 (35.16–60.14)	< 0.001	91.8	0.184	Moderate^b^ ⨁⨁⨁◯
		MP	24	1766	9.43 (7.96–10.91)	59.68 (54.37–65.01)	14.77 (11.74–17.79)	−44.72 (−49.50, −39.94)	< 0.001	95.6	0.948	Moderate^b^ ⨁⨁⨁◯
		NSA	15	966	9.49 (8.06–10.93)	152.9 (149.3–156.6)	119.7 (107.9–131.4)	−33.34 (−44.01, −22.67)	< 0.001	98.8	0.205	Moderate^b^ ⨁⨁⨁◯
		ShA	6	159	11.35 (7.61–15.10)	52.18 (50.25–54.11)	40.14 (36.11–44.17)	−12.22 (−15.68, −8.75)	< 0.001	62.5	0.412	Moderate^b^ ⨁⨁⨁◯
		TA	3	44	15.55 (10.78–20.33)	29.98 (12.05–47.92)	10.22 (1.39–19.04)	−19.47 (−38.29, −0.65)	0.047	92.6	0.744	Moderate^b^ ⨁⨁⨁◯

*AI* acetabular index, *CEA* center edge angle, *MP* migration percentage, *NSA* neck shaft angle, *HSA* head shaft angle, *PO* pelvic obliquity, *ShA* Sharp’s angle, *TA* Tönnis angle, *Botox* botulinum toxin A, *SDR* selective dorsal rhizotomy. * Significant publication bias

GRADE Working Group grades of evidence: High quality—we have strong confidence that the true effect is very close to the estimated effect. Moderate quality—we are somewhat confident in the effect estimate, with the true effect likely to be close to the estimate; however, there is a possibility that it may differ substantially. Low quality—our confidence in the effect estimate is limited, and the true effect could differ significantly from the estimated effect. Very low quality—we have little confidence in the effect estimate, and the true effect is likely to differ substantially from the estimated effect

^a^ There were studies of unclear and high summarized risk of bias (risk of bias)

^b^ There was heterogeneity as noted by *I*^2^(inconsistency)

^c^ Indirectness

^d^ 95% confidence interval includes “no effect” (impression)

^e^ Publication bias

### Pelvic osteotomy

Out of 16 studies on patients with CP with spastic hip undergoing PO, six (334 patients) showed a significant reduction in AI [mean difference (MD) = −19.57°, *p* < 0.001]. Eight studies (149 patients) found an increase in CEA [MD = 35.27°, *p* < 0.001]. Eleven studies examined MP in 527 patients, showing a significant postoperative decrease (MD = −53.06, *p* < 0.001). NSA, evaluated in three studies with 634 patients, showed a reduction, but it was not statistically significant (MD = −23.86, *p* = 0.20).

### Femoral osteotomy

In 13 studies involving 957 patients with spastic-type CP who underwent FO, radiologic outcomes were assessed. Five studies (370 patients) analyzing AI found a significant reduction post-surgery (MD = −5.39°, *p* = 0.032). Similarly, ten studies (737 patients) reporting MP showed a significant decrease (MD = −23.91, *p* = 0.002). However, three studies (190 patients) assessing CEA showed an increase post-surgery, but the change was not statistically significant (MD = 11.75°, *p* = 0.13). Eight studies (712 patients) evaluating NSA demonstrated a significant reduction in postoperative NSA degrees (MD = −30.09, *p* = 0.008). Meta-analysis for other radiologic measures was not possible owing to insufficient data.

### Combined femoral and pelvic osteotomy

**Fig. 3:**
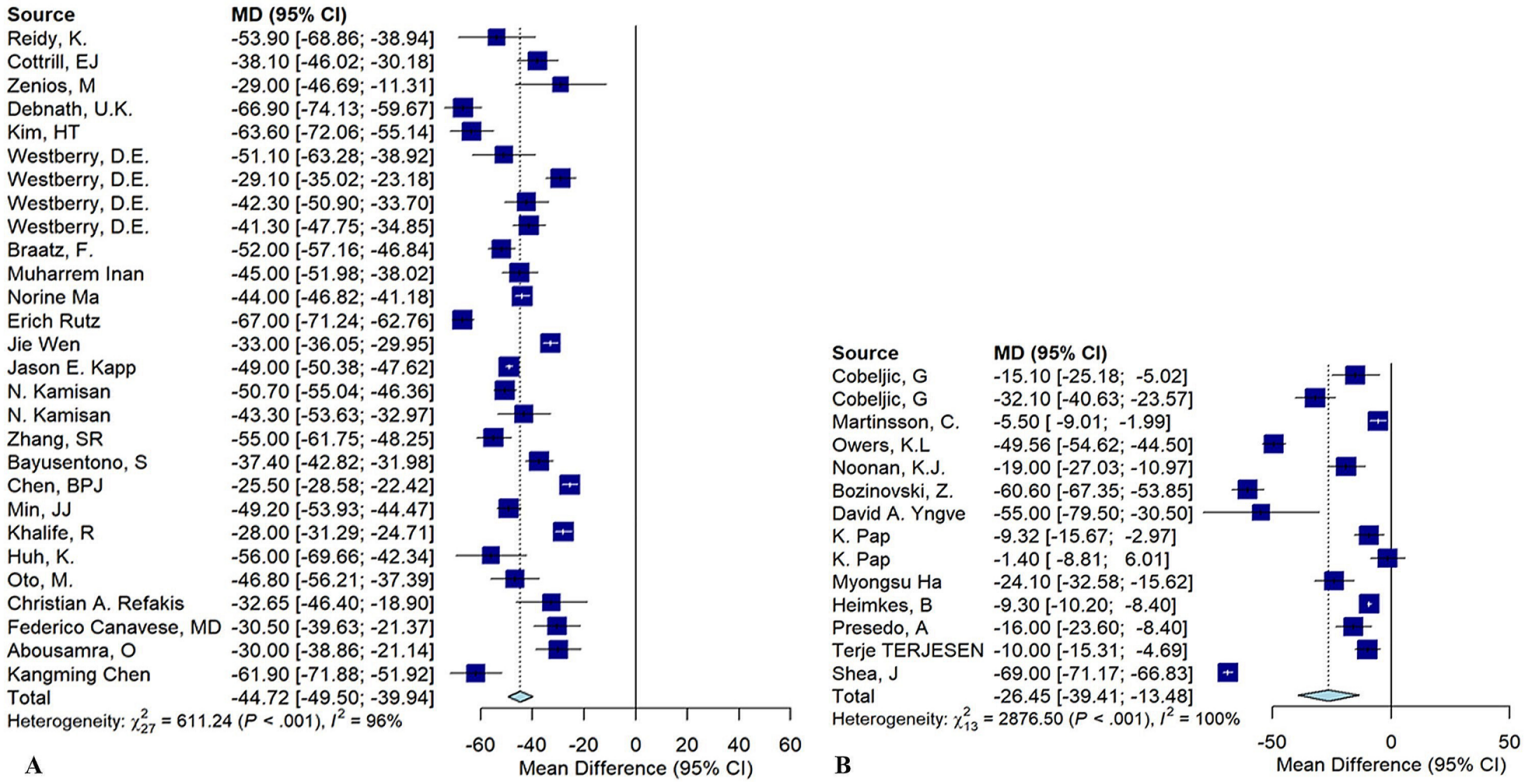
Forest plots showing **A** the combination of femur osteotomy and pelvic osteotomy, migration percentage; **B** soft tissue surgery, migration percentage

In 26 studies analyzing the effects of CFPO on patients with spastic CP, meta-analysis revealed significant postoperative reductions in AI (MD = −11.18, *p* < 0.001), MP (MD = −44.72, *p* < 0.001), NSA (MD =−33.34, *p* < 0.001), ShA (MD = −12.22, *p* < 0.001), and TA (MD = −19.47, *p* = 0.47), while CEA improved significantly (MD = 47.65°, *p* < 0.001) (Fig. 3A). Other radiologic values could not be quantitatively analyzed owing to insufficient data, highlighting the positive impact of combined osteotomy on key radiographic outcomes for these patients.

### Soft tissue surgery

In 14 studies examining radiologic outcomes for patients with spastic hip CP who underwent STS, postoperative AI decreased, but this change was not statistically significant (MD = −9.00, *p* = 0.106). In contrast, CEA significantly increased post-surgery (MD = 16.10, *p* = 0.007), while MP showed a substantial decrease (MD = −26.44, *p* < 0.001) (Fig. 3B). Owing to the limited number of studies, other radiologic variables could not be meta-analyzed.

### Tone decreasing surgery

Nine studies measured the radiological outcomes of TDS in individuals with spastic CP. Eight studies involving 622 patients with CP, examined nonsignificant reduction in MP (MD = −1.9, *p* = 0.292).

### Guided growth surgery

Three studies evaluated the outcomes of patients with spastic hip CP undergoing GGS. Owing to limited data available on other outcomes, only one meta-analysis was conducted, showing a significant decrease in MP (MD = −9.97°, *p* = 0.004).

### Percutaneous osteotomy

Three studies examined radiologic outcomes of PCO in patients with spastic CP, with quantitative analysis only performed for MP. The results showed a significant reduction in postoperative MP values (MD = −59.94,* p* < 0.001).

### Publication bias

**Fig. 4:**
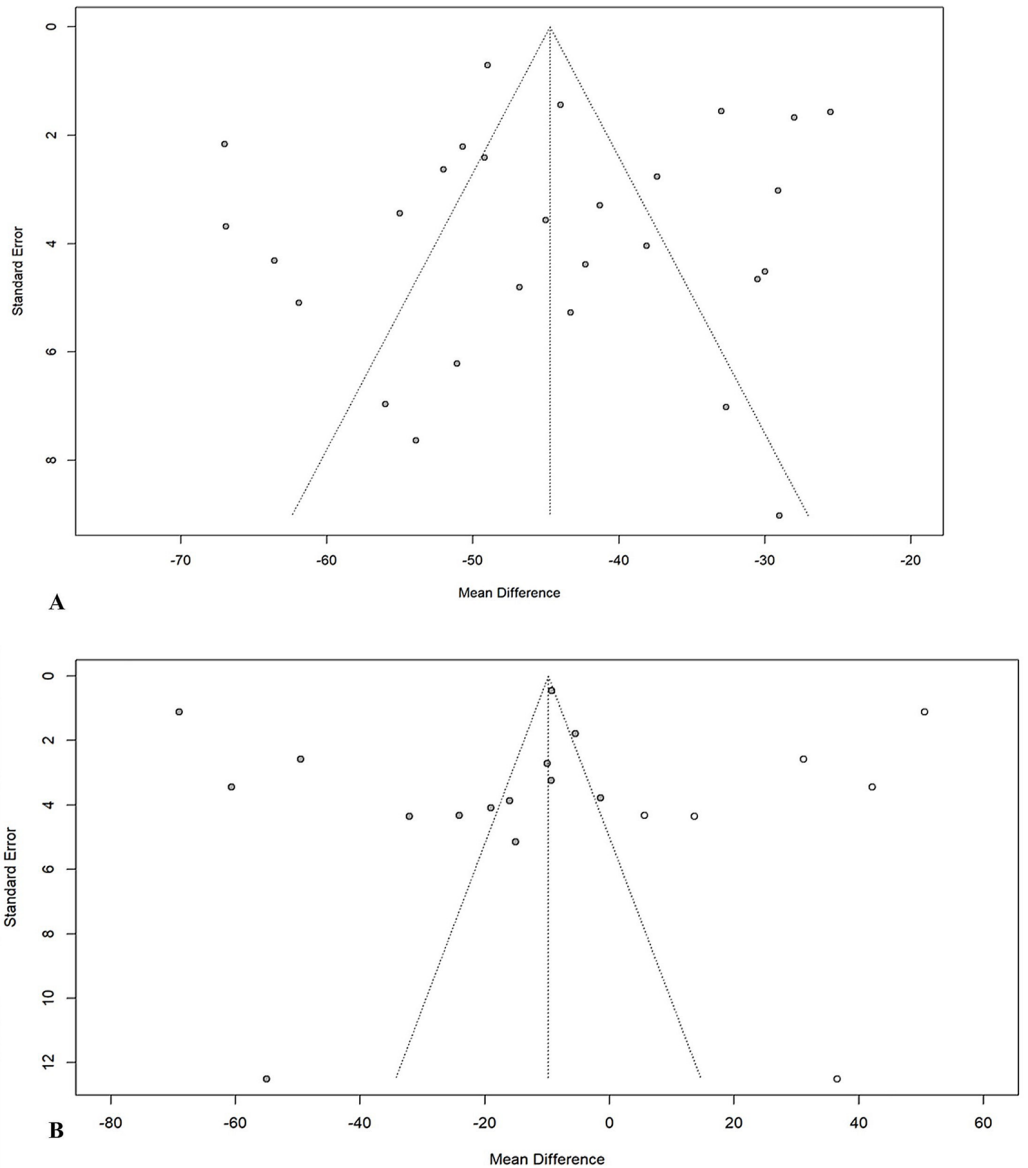
Funnel plots showing **A** the combination of femur osteotomy and pelvic osteotomy, migration percentage; **B** soft tissue surgery, migration percentage

To assess whether publication bias influenced the results of the analysis, Egger’s test was applied. A significant difference was detected in four domains of our meta-analysis, including CEA in the PO group (*p* = 0.018), AI in CFPO patients (*p* = 0.037), and MP and NSA in patients with FO (*p* = 0.026 and 0.008, respectively), suggesting probable publication bias in these outcomes. The effect size of these analyses was adjusted by conducting the trim and fill method (Table 3). Funnel plots depicting publication bias are shown in Fig. 4 and Appendix D.

**Table 3:** Trim and fill test results of the radiologic outcomes in various interventions

Type of intervention		Radiographic outcome	Studies (*n*)	Studies added (*n*)	Feet (*n*)	MD (95% CI)	*p*-Value	*I*^2^ (%)
Tone decreasing surgery	Total	MP	14	4	873	1.13 (−3.49, 5.76)	0.606	85.8
	Botox	MP	4	0	206	−4.20 (−13.68, 5.29)	0.286	76.1
	SDR	MP	5	2	556	4.00 (−3.70, 11.70)	0.222	91.2
Soft tissue surgery		AI	5	0	256	−8.13 (−23.47, 7.21)	0.215	96.1
		CEA	8	2	328	19.38 (1.90, 36.85)	0.034	95.5
		MP	19	5	1086	−7.57 (−24.7, 9.61)	0.367	99.7
Guided growth surgery		MP	3	0	117	−9.97 (−12.65, −7.3)	0.004	0
Percutaneous osteotomy		MP	4	0	141	−59.94 (−63.90, −55.97)	< 0.001	0
Pelvic osteotomy		AI	8	2	424	−19.97 (−21.58, −18.35)	< 0.001	49.9
		CEA	11	3	188	25.81 (10.54, 41.07)	0.004	90.7
		MP	15	4	642	−61.65 (−72.04, −51.26)	< 0.001	95.7
		NSA	3	0	171	−23.85 ( −77.58, 29.86)	0.196	97.9
		ShA	6	2	183	−16.06 (−21.84, −10.29)	0.001	89.6
		TA	5	2	66	−11.8 (−24.40, 0.79)	0.060	90.6
Femoral osteotomy		AI	9	3	548	−1.16 (−6.09, 3.77)	0.603	96.4
		CEA	5	2	336	6.00 (−7.54, 19.54)	0.286	95.0
		MP	14	4	1091	−10.53 (−25.87, 4.82)	0.162	98.8
		NSA	13	5	1161	−5.15 (−28.93, 18.63)	0.645	99.7
Combined femoral and pelvic osteotomy		AI	20	6	803	−14.56 (−17.77, −11.35)	< 0.001	94.4
		CEA	12	3	410	39.22 (25.90–52.54)	< 0.001	92.5
		MP	28	0	1766	−44.72 (−49.50, −39.94)	< 0.001	95.6
		NSA	24	6	1129	−24.36 (−36.19, −12.52)	< 0.001	99.1
		ShA	6	0	159	−12.22 (−15.68, −8.75)	< 0.001	62.5
		TA	3	0	44	−19.47 (−38.29, −0.65)	0.047	92.6

*AI* acetabular index, *CEA* center edge angle, *MP* migration percentage, *NSA* neck shaft angle, *HSA* head shaft angle, *PO* pelvic obliquity, *ShA* Sharp’s angle, *TA* Tönnis angle, *Botox* botulinum toxin A, *SDR* selective dorsal rhizotomy

### Sensitivity analysis

**Fig. 5:**
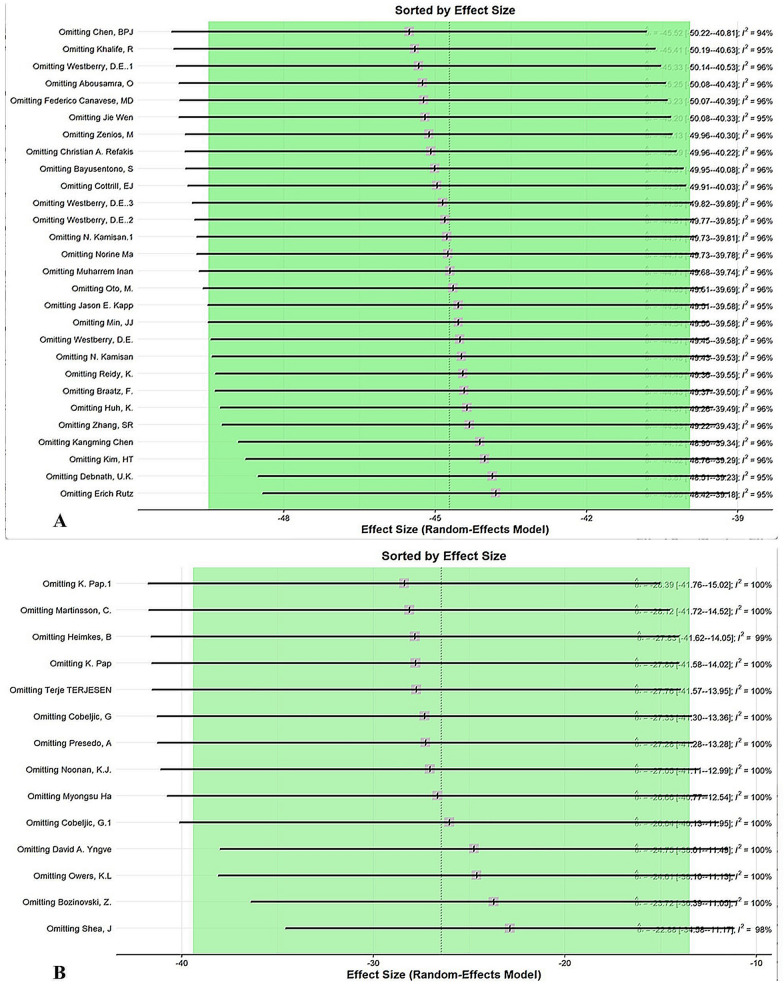
Sensitivity analysis using the leave-one-out method: **A** the combination of femur osteotomy and pelvic osteotomy, migration percentage; **B** soft tissue surgery, migration percentage

Regarding the presence of heterogeneity in our meta-analysis, sensitivity analysis was conducted by omitting one study at a time to reveal which study could potentially impact the results of our evaluations (Fig. 5 and Appendix E).

### Risk of bias assessment

Appendix F shows the risk of bias and concerns regarding applicability for each domain across the included studies.

The quality of evidence according to GRADE is presented in Table 2 for each meta-analysis. Among the studies, 10 were rated as having low-quality evidence, 11 as moderate-quality evidence, and 3 as high-quality evidence.

### Subgroup analysis

**Fig. 6:**
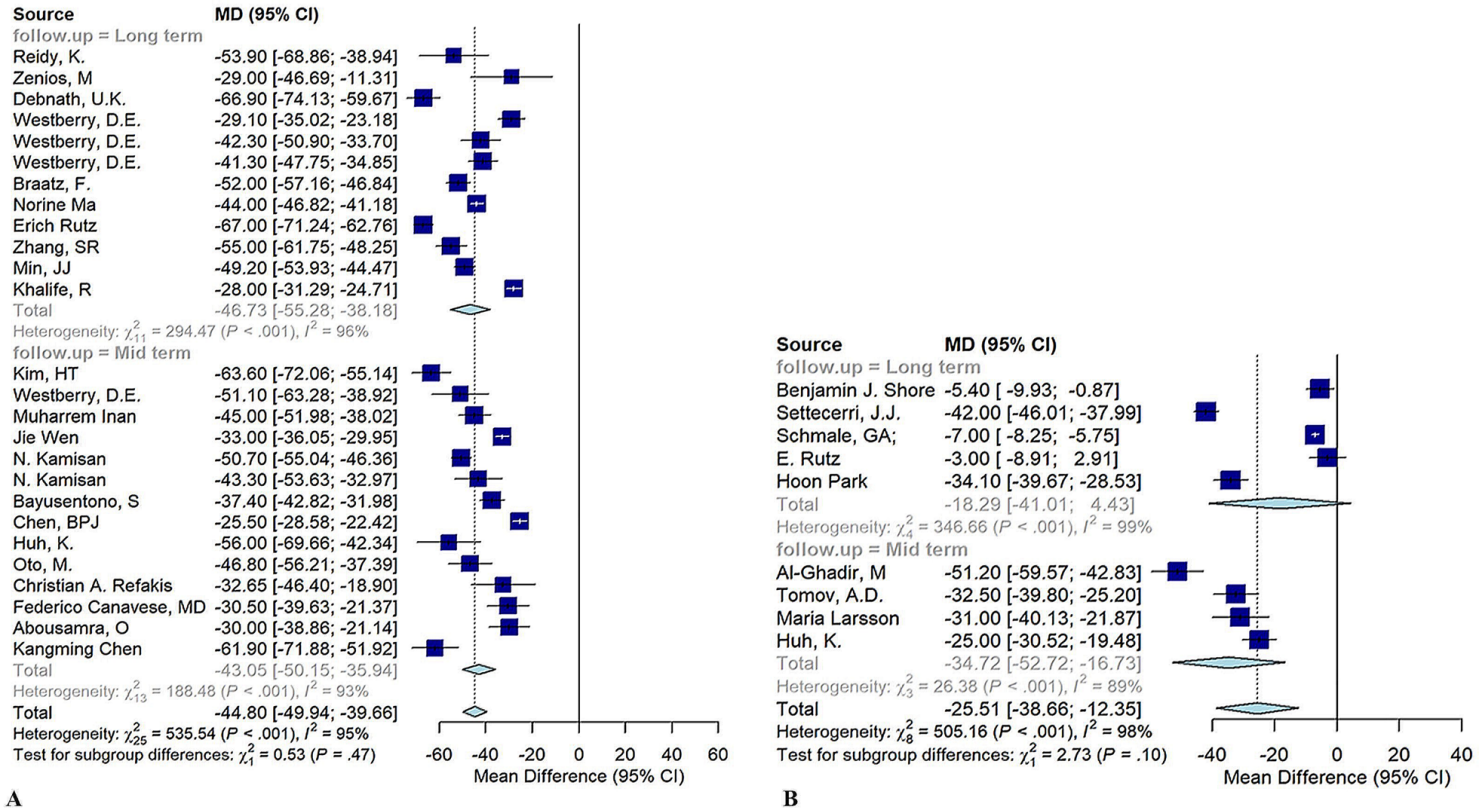
Subgroup analysis of **A** migration percentage after combined femoral and pelvic osteotomy intervention; **B** migration percentage after femoral osteotomy; in various follow-up durations

The results of each study within each intervention were divided into three subgroups on the basis of their follow-up time: less than two years (short term), between 2 and 5 years (midterm), and more than 5 years (long term). The results of each subgroup are described in Table 4, Fig. 6, and Appendix G. Our results suggest that radiologic parameters did not change significantly with increasing follow-up time (*p* > 0.05).

**Table 4:** Subgroup meta-analysis of radiologic angles across interventions at varying follow-ups

Type of surgery	Radiologic angle	Follow-up	Studies (*n*)	Feet (*n*)	MD	95% CI	*I*^2^ (%)	Subgroup *p*-value
Tone decreasing surgery	MP	Short term	4	422	−0.49	−4.69; 3.70	83.4	0.716
		Midterm	3	270	−1.45	−8.31; 5.40	51.3	
Soft tissue surgery	MP	Midterm	5	462	−24.24	−50.32; 1.84	98.6	0.725
		Long term	6	262	−20.24	−32.75; −7.72	89.1	
Femoral osteotomy	MP	Midterm	4	196	−34.72	−52.72; −16.73	88.6	0.098
		Long term	5	383	−18.29	−41.01; 4.43	98.8	
Combined femoral and pelvic osteotomy	AI	Midterm	8	240	−10.09	−13.40; −6.79	74.8	0.457
		Long term	5	313	−12.05	−18.23; −5.87	94.9	
	CEA	Midterm	5	154	47.07	28.24; 65.89	89.0	0.929
		Long term	4	162	48.15	15.50; 80.81	95.0	
	MP	Midterm	14	752	−43.04	−50.15; −35.94	93.1	0.469
		Long term	12	914	−46.73	−55.28; −38.18	96.3	

*AI* acetabular index, *CEA* center edge angle, *MP* migration percentage

**Fig. 7:**
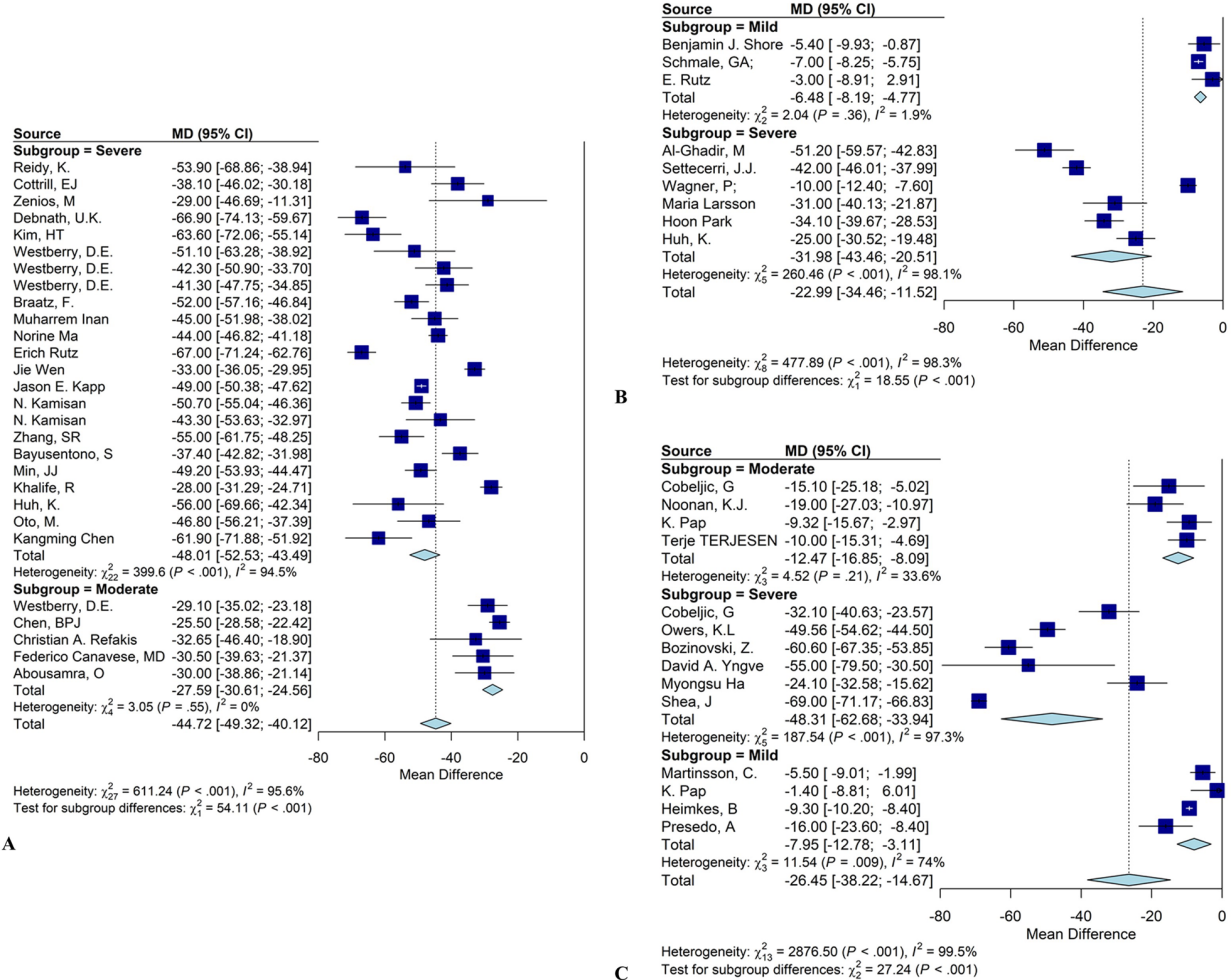
Subgroup analysis of **A** migration percentage after combined femoral and pelvic osteotomy intervention; **B** migration percentage after femoral osteotomy; **C** migration percentage after soft tissue surgeries; in various severities

In addition, subgroup analyses based on preoperative severity were performed. MP was categorized into three severity groups: mild (MP < 39°), moderate (39° ≤ MP ≤ 49°), and severe (MP > 49°) [121]. The results of this analysis are presented in Table 5 and Fig. 7. This subgroup analysis was conducted for STS, FO, and CFPO. Owing to the limited number of studies, subgroup analyses for other surgical procedures could not be performed. Our findings indicate that in all three interventions, patients in the severe group experienced significantly greater improvement in MP compared with mild and moderate groups (*p* < 0.0001).

**Table 5:** Subgroup meta-analysis of migration percentage across interventions at different severities

Type of surgery	Follow-up	Studies (*n*)	Feet (*n*)	MD	95% CI	*I*^2^ (%)	Subgroup *p*-value
Soft tissue surgery	Moderate	4	130	−12.47	−16.85; −8.09	99.5	< 0.0001
	Severe	6	278	−48.31	−62.68; −33.94		
	Mild	4	443	−7.94	−12.78; −3.11		
Femoral osteotomy	Mild	3	109	−6.48	−8.18; −4.77	98.3	< 0.0001
	Severe	6	584	−31.98	−43.46; −20.50		
Combined femoral and pelvic osteotomy	Severe	23	1371	−48.01	−52.53; −43.49	95.6	< 0.0001
	Moderate	5	395	−27.59	−30.61; −24.56

## Discussion

This systematic review and meta-analysis assessed the radiological outcomes of various surgical interventions for spastic hip deformities in patients with cerebral palsy, including PO, FO, CFPO, STS, TDS, OR, GGS, and PCO. It synthesized quantitative data for key radiological parameters, such as AI, CEA, and MP. This study is notable for being the most comprehensive meta-analytic comparison of these techniques in the literature, and includes recent studies; offering an updated perspective and deeper insights through quantitative synthesis.

This meta-analysis revealed significant improvements in several key radiographic parameters following PO in patients with CP. Specifically, the AI showed a large reduction (MD = −19.57, *p* < 0.001), indicating improved acetabular formation and coverage. The CEA increased substantially (MD = 35.27, *p* < 0.001), reflecting enhanced lateral femoral head containment. MP also decreased significantly (MD = −53.06, *p* < 0.001), suggesting reduced hip subluxation/dislocation. These findings align with previous studies demonstrating the ability of PO, such as the Dega, to reorient the dysplastic acetabulum and improve femoral head coverage [43, 44]. Femoral varus derotational osteotomy led to a significant reduction in AI (MD = −4.44, *p* = 0.042) and MP (MD = −23.91, *p* = 0.002). This indicates improved acetabular morphology and hip containment following femoral geometry correction, consistent with prior studies [52, 53]. However, the change in CEA (MD = 11.75, *p* = 0.130) was not statistically significant. Patients undergoing CFPO exhibited significant improvements across AI (MD = −11.18, *p* < 0.001), MP (MD = 47.65, *p* < 0.001), NSA (MD = −33.34, *p* < 0.001), ShA (MD = −12.22, *p* < 0.001), and TA (MD = −19.47, *p* = 0.047). The CEA also increased significantly (MD = 47.65, *p* < 0.001). These findings highlight the ability of combined bony procedures to comprehensively address both acetabular deficiencies and proximal femoral deformities [42, 72].

STS alone had a more modest impact on radiographic measures of hip dysplasia. No significant changes were observed in AI (MD = −8.13, *p* = 0.215) or CEA (MD = 11.34, *p* = 0.128) postoperatively. However, MP showed a statistically significant decrease (MD = −23.01, *p* = 0.006), suggesting that STS positively influences hip alignment and migration in these patients. These results also align with previous studies suggesting STS may help improve hip abduction, but do not adequately address severe bony deformities [96, 122]. STS is commonly performed as part of the overall management for addressing spastic hip in patients with CP. These procedures often include adductor release and iliopsoas release, which are frequently combined with other interventions. In our study, the STS group consisted of studies that focused exclusively on soft tissue surgeries, without any additional bony procedures. However, several limitations have been identified for STS alone. Notably, Owers et al. indicated no significant improvements were observed in the total range of hip motion following these procedures. Also, no significant differences in preoperative and postoperative changes in any parameters for both dystonic and hypertonic groups were observed [89]. Furthermore, Noonan et al. reported that soft tissue-only procedures were associated with a higher risk of deterioration in MP compared with patients who underwent bony reconstruction [90].

The meta-analysis did not demonstrate a significant change in MP (MD = −1.90, *p* = 0.292) following TDS procedures, such as selective dorsal rhizotomy or botulinum toxin injections. This corroborates prior evidence indicating these interventions primarily impact spasticity and range of motion, with limited effects on established hip dysplasia or subluxation [101, 104]. GGS resulted in a significant reduction in MP (MD = −9.97, *p* = 0.004). While data are limited, this finding supports previous studies demonstrating the ability of proximal femoral hemi-epiphysiodesis to gradually improve varus positioning and hip containment [115, 116]. Percutaneous pelvic and intertrochanteric osteotomies led to a significant decrease in MP (MD = −59.954, *p* < 0.001). Though few studies were available, this aligns with reports suggesting these minimally-invasive techniques can provide satisfactory radiographic correction [118, 120].

A subgroup analysis based on follow-up duration indicated that radiologic parameters remained stable, showing no significant changes with extended follow-up periods (*p* > 0.05). These findings suggest that relapse and undercorrection are unlikely to occur over time. In addition, a subgroup analysis based on preoperative MP severity demonstrated that patients with more severe deformities experienced greater improvements from the interventions compared with those with moderate or mild deformities. This suggests that patients with higher degrees of deformity may derive greater benefit from these interventions.

**Fig. 8:**
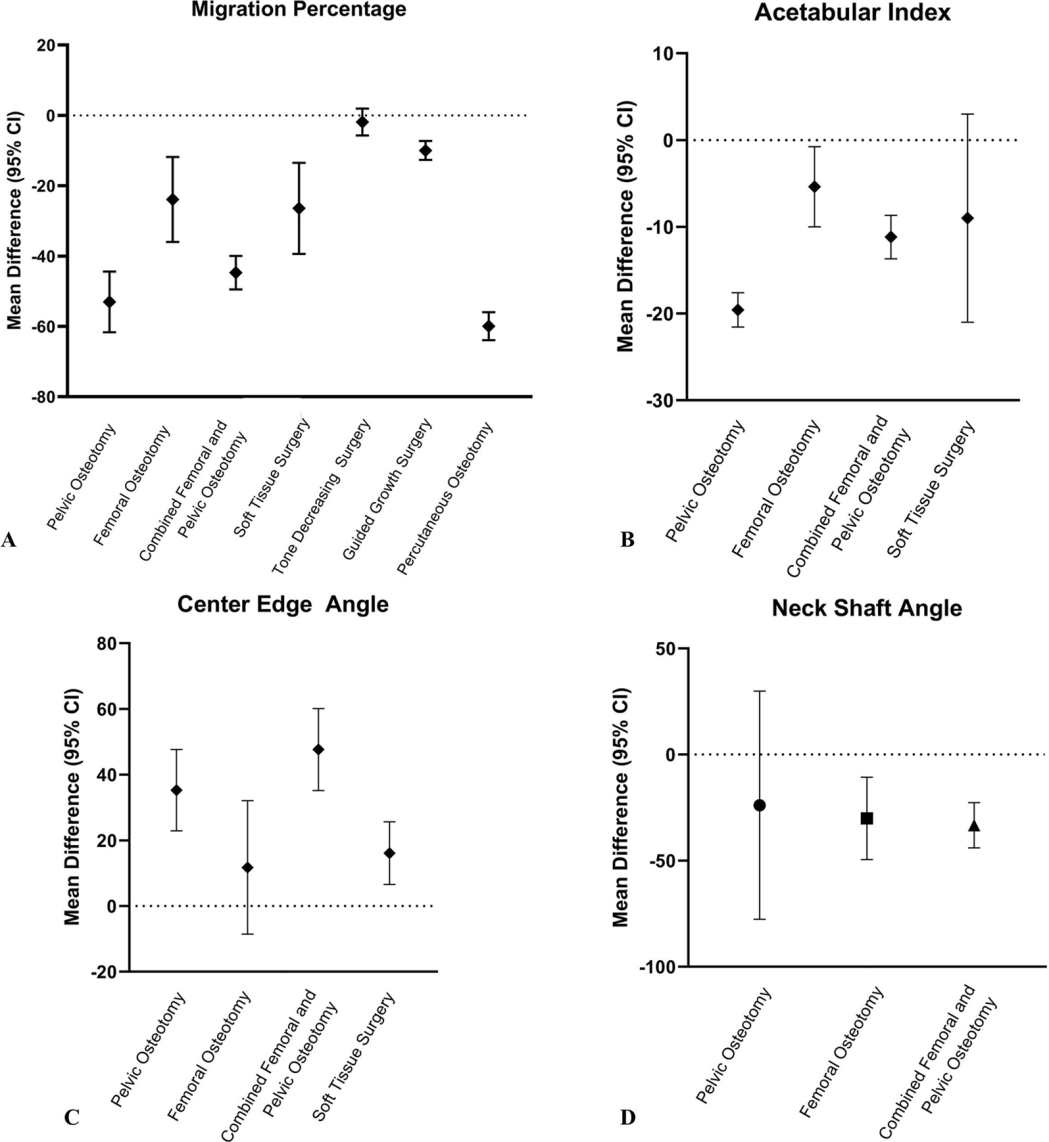
Mean differences with 95% confidence intervals of migration percentage, acetabular index, center–edge angle, and neck shaft angle in different surgical methods

**Fig. 9:**
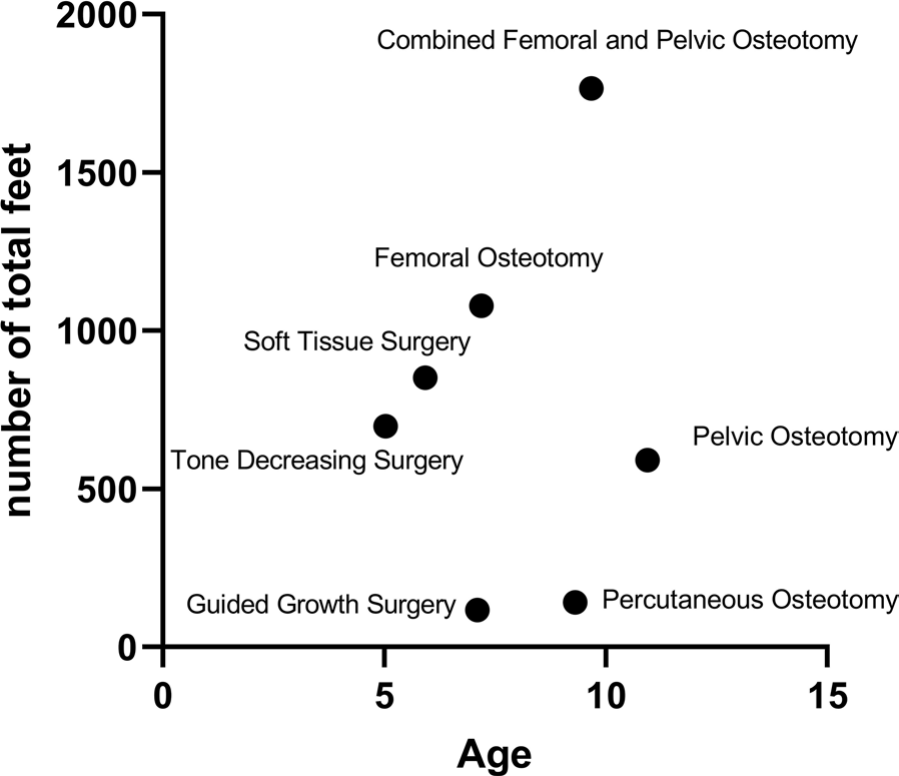
Total number of feet that have undergone surgery in each surgery type (from included studies) across different age groups (in years)

Figure 8 illustrates the mean difference (95% CI) of MP, AI, CEA, and NSA through different surgical methods. PO and PCO are two types of interventions that decrease MP more than other modalities (Fig. 8A). In addition, PO improves AI more than other surgical methods (Fig. 8B), but for enhancing the CEA, a CFPO suggests better outcomes (Fig. 8C). Finally, there was no significant difference among various surgical methods for NSA improvement (Fig. 8D). These findings were in line with our hypothesis that PO and CFPO show better outcomes. It is important to note that these results may be confounded by patients’ characteristics (e.g., age). Therefore, the interpretation of these results should be approached with caution. Figure 9 displays the total number of hips that have undergone surgery in each surgery type (from included studies) across different age groups (in years). Children under 6 years mostly underwent TDS and STS. In slightly older children (around 7 years), FO and GGS were more popular. Finally, in children over 9 years; PO, CFPO, and PCO were more common. In addition, the severity of disability (GMFCS level) may also be considered another potential confounding factor. In most of the studies included in this review, results were not differentiated on the basis of GMFCS levels. Instead, the populations were grouped together across all GMFCS levels, which made it impossible for the effect of GMFCS severity on the outcomes to be evaluated.

Despite limitations in analyzing the clinical outcomes of the included studies, some studies suggested that improvements in radiologic outcomes were associated with clinical improvements. Kim et al. reported significant improvements after femoral and pelvic osteotomy, with enhanced radiographic outcomes (MP, AI, and CEA). The median hip abduction range increased from 21.8° to 40.0°. Postoperatively, 75% of patients who could not sit independently pre-surgery were able to do so without support. Pain decreased in 83% of patients, and none experienced increased pain. In addition, 26% of patients improved from GMFCS level V to level IV [67]. Rutz et al. found significant reductions in both the intensity and frequency of pain following treatment. Preoperative femoral head shape did not significantly influence changes in pain, MCPHCS grade, or GMFCS level. However, the preoperative MP emerged as the most significant risk factor affecting postoperative outcomes [72].

Publication bias was quantitatively assessed using Egger’s test, which identified significant bias in four meta-analyses: CEA in the PO group (*p* = 0.018), AI in CFPO patients (*p* = 0.037), and MP and NSA among FO patients (*p* = 0.026 and *p* = 0.008, respectively). The retrospective nature of many included studies introduced inherent limitations, such as selection bias, incomplete data, and variability in study design, all of which may impact the reliability and generalizability of the findings. To address these challenges, more rigorous prospective studies are necessary to mitigate bias and enhance the overall quality of evidence in future research.

As summarized in Table 2, the evidence quality varied across the studies, with 10 rated as low-quality, 11 as moderate-quality, and 3 as high-quality. These variations in evidence quality could affect the robustness and applicability of our conclusions. The GRADE framework considers multiple factors that influence the overall quality of evidence, including study limitations, publication bias, indirectness, inconsistency, and imprecision. In particular, studies rated as low quality often had methodological issues, such as risk of bias or small sample sizes, which may undermine the reliability of their findings. Furthermore, imprecision in the results, which was assessed through trial sequential analysis (TSA), could have resulted in wide confidence intervals that limit the certainty of the effects observed. While moderate and high-quality studies provide stronger evidence, their relative scarcity in this analysis suggests that the findings should be interpreted with caution, especially for those outcomes based on lower-quality studies. In light of this, future research should aim to enhance the methodological rigor and sample size of studies in this field to improve the overall evidence quality and its subsequent impact on clinical recommendations.

The quantitative results provide important benchmarks for anticipating the degree of radiographic improvement following various surgical interventions in patients with CP and spastic hip disease. However, these findings must be interpreted with caution, owing to the significant limitations of the study, stemming primarily from the substantial heterogeneity across the included studies. This variability is driven by several factors, including differences in patient characteristics, such as age and sex, as well as variations in the radiographic assessment methods used. Moreover, some studies did not report the specific radiological techniques they employed, further complicating the ability to draw robust conclusions. In addition, while advanced imaging techniques, such as digital tomosynthesis (DTS), offer a more detailed and accurate view of structural changes, it is important to note that these methods have only recently been developed. As such, they were not utilized in the studies included in this review, which may limit the precision and reliability of the radiographic data reported. While the studies were categorized on the basis of the type of surgery (e.g., PO, FO, CFPO), it is important to acknowledge that differences in surgical technique may still exist due to variations in surgeon experience and expertise. As such, it cannot be assumed that all surgical procedures included in each subgroup were performed in an identical manner. These sources of heterogeneity highlight the need to interpret the pooled results of each meta-analysis with appropriate caution.

The clinical implications of our findings are significant in guiding treatment decisions for spastic hip deformity in patients with CP. Our study demonstrates that surgical interventions yield long-term stability in radiologic outcomes, providing clinicians with confidence in planning long-term management, and assuring patients and caregivers of minimal risk of relapse. In addition, recognizing that patients with more severe preoperative deformities show greater radiological improvements allows for personalized treatment plans and prioritization of intensive interventions for these cases. The data on the variance in effectiveness among surgical methods, such as PO and CFPO, for different radiologic outcomes (e.g., MP and CEA) supports a tailored approach to selecting appropriate interventions on the basis of deformity type and target outcomes. Furthermore, the age-dependent trends in surgical preferences underscore the importance of timing in selecting interventions, where younger children benefit from less invasive surgeries, such as TDS and STS, while older children often require more complex procedures, such as PO and CFPO. These insights collectively enhance clinical practice by ensuring that treatment strategies are both personalized and evidence-based, optimizing recovery and functional outcomes for children with CP.

This study is the most comprehensive systematic review and meta-analysis to date evaluating radiological outcomes of various surgical treatments for spastic hip deformity in cerebral palsy. A rigorous search strategy and bias prevention methods were used to minimize the risk of bias. A large number of included studies and patients allowed for a robust quantitative synthesis. However, limitations also exist. Considerable heterogeneity was present necessitating random effects modeling. Publication bias was detected in some domains. Variability in follow-up durations, definitions of outcomes, and study quality introduced heterogeneity. Confounding from additional interventions, insufficient adjustment for prognostic factors, and the retrospective nature of most studies impact interpretability. The lack of functional outcomes assessment is another limitation.

## Conclusions

This systematic review and meta-analysis underscores the superior efficacy of PO and CFPO in correcting spastic hip deformity in children with CP. Radiological outcomes, particularly the MP, demonstrated significant improvements following these procedures. The findings suggest that these approaches are particularly effective for complex cases where procedures such as FO, STS, or TDS may fall short. Given the complex nature of spastic hip deformity, a tailored surgical approach that addresses both skeletal and soft tissue abnormalities is recommended. Future studies should focus on refining surgical protocols and exploring the long-term functional outcomes of these interventions.

## Supplementary Information

Additional file 1.

Additional file 2.

## Data Availability

The datasets generated during and/or analyzed during the current study are available from the corresponding author on reasonable request.
