# Supplementary material for: Radiological outcomes of surgical techniques for spastic hip in cerebral palsy: a systematic review and meta-analysis
Source: J Orthop Traumatol. 2025 Feb 28;26:13. doi: 10.1186/s10195-025-00827-0 (PMC11871257; doi:10.1186/s10195-025-00827-0)
Supplement: Supplementary file 1 — Additional file 1. [file 10195_2025_827_MOESM1_ESM.docx]

**Appendix A (search strategy)**

**PubMed:**

("cerebral palsy"[tiab] OR "cerebral palsy"[MeSH Terms])

AND

(“Spastic hip”[tiab] OR “Hip dislocation”[tiab] OR “dislocated hip”[tiab] OR “hip joint dislocation”[tiab] OR “Hip displacement*”[tiab] OR “hip instability”[tiab] OR “Hip subluxation”[tiab] OR “hip joint subluxation”[tiab] OR “Hip lateralization”[tiab] OR “acetabular reorientation”[tiab] OR “femoral head medialization”[tiab] OR “Hip dysplasia”[tiab] OR “hip joint dysplasia”[tiab] OR “Femoral head dislocation”[tiab] OR “dislocated femoral head”[tiab] OR “Femoral head displacement”[tiab] OR “Femoral head subluxation”[tiab] OR “femoral head insurability”[tiab] OR “Femoral head lateralization”[tiab] OR “femoral head dysplasia”[tiab] OR “hip contracture”[tiab])

AND

(child*[MeSH Terms] OR infant*[MeSH Terms] OR pediatric*[MeSH Terms] OR kid[tiab] OR Adolescent[tiab] OR baby[tiab] OR toddler[tiab] OR Neonate[tiab] OR Juvenile[tiab] OR Minor[tiab] OR "Young patient"[tiab] OR "Preschool"[tiab])

AND

(Surg*[MeSH Terms] OR Procedure[Title/Abstract] OR Operation[Title/Abstract] OR Operative[tiab] OR Intervention[Title/Abstract] OR treatment[MeSH Terms] OR method[MeSH Terms] OR strateg*[Title/Abstract] OR technique[Title/Abstract] OR "VDRO"[Title/Abstract] OR Osteotomy[Title/Abstract] OR "Soft Tissue Release"[Title/Abstract] OR "Soft-tissue release"[Title/Abstract] OR release[tiab] OR Detachment[tiab] OR "Varus derotation"[Title/Abstract] OR "femoral derotation"[Title/Abstract] OR acetabular[tiab] OR Dega[tiab] OR Pemberton[tiab] OR reduction[tiab] OR lengthening[tiab] OR tenotomy[MeSH Terms] OR myotomy[MeSH Terms] OR “femoral shortening”[tiab] OR arthroplasty[MeSH Terms] OR colonna[tiab] OR resection[tiab] OR Girdlestone[tiab] OR “hip resurfacing”[tiab] OR THA[tiab] OR hemiarthroplasty[tiab] OR “hip replacement*”[tiab] OR hemiepiphysiodesis[tiab] OR “growth modulation”[tiab] OR “guided growth”[tiab] OR “physeal stapling”[tiab] OR “botulinum toxin”[tiab] OR botox[MeSH Terms] OR neurotoxin[tiab] OR AbobotulinumtoxinA[tiab] OR BoNT[tiab] OR “toxin injection”[tiab] OR “Hip reconstruction”[tiab] OR “Hip surgery”[tiab] OR “Closed physis”[tiab] OR “closed triradiate cartilage”[tiab] OR “salvage surgery”[tiab] OR “Chiari pelvic”[tiab] OR “selective dorsal rhizotomy”[tiab] OR “selective posterior rhizotomy”[tiab])

**SCOPUS:**

(

TITLE-ABS-KEY("cerebral palsy")

OR INDEXTERMS("cerebral palsy")

)
AND
(
TITLE-ABS-KEY("Spastic hip")
OR TITLE-ABS-KEY("Hip dislocation")
OR TITLE-ABS-KEY("dislocated hip")
OR TITLE-ABS-KEY("hip joint dislocation")
OR TITLE-ABS-KEY("Hip displacement*")
OR TITLE-ABS-KEY("hip instability")
OR TITLE-ABS-KEY("Hip subluxation")
OR TITLE-ABS-KEY("hip joint subluxation")
OR TITLE-ABS-KEY("Hip lateralization")
OR TITLE-ABS-KEY("acetabular reorientation")
OR TITLE-ABS-KEY("femoral head medialization")
OR TITLE-ABS-KEY("Hip dysplasia")
OR TITLE-ABS-KEY("hip joint dysplasia")
OR TITLE-ABS-KEY("Femoral head dislocation")
OR TITLE-ABS-KEY("dislocated femoral head")
OR TITLE-ABS-KEY("Femoral head displacement")
OR TITLE-ABS-KEY("Femoral head subluxation")
OR TITLE-ABS-KEY("femoral head insurability")
OR TITLE-ABS-KEY("Femoral head lateralization")
OR TITLE-ABS-KEY("femoral head dysplasia")
OR TITLE-ABS-KEY("Hip displacement")
OR TITLE-ABS-KEY("hip contracture")
)
AND
(
INDEXTERMS("child*")
OR TITLE-ABS-KEY("child*")
OR INDEXTERMS("Infant*")
OR TITLE-ABS-KEY("infant*")
OR AFFIL("pediatric*")
OR INDEXTERMS("pediatric*")
OR TITLE-ABS-KEY("kid")
OR TITLE-ABS-KEY("Adolescent")
OR TITLE-ABS-KEY("baby")
OR TITLE-ABS-KEY("toddler")
OR TITLE-ABS-KEY("Neonate")
OR TITLE-ABS-KEY("Juvenile")
OR TITLE-ABS-KEY("Minor")
OR TITLE-ABS-KEY("Young patient")
OR TITLE-ABS-KEY("Preschool")
)
AND
(
INDEXTERMS("Surg*")
OR TITLE-ABS-KEY("Procedure")
OR TITLE-ABS-KEY("Operation")
OR TITLE-ABS-KEY("Operative")
OR TITLE-ABS-KEY("Intervention")
OR INDEXTERMS("treatment")
OR INDEXTERMS("method")
OR TITLE-ABS-KEY("strateg*")
OR TITLE-ABS-KEY("technique")
OR TITLE-ABS-KEY("VDRO")
OR TITLE-ABS-KEY("Osteotomy")
OR TITLE-ABS-KEY("Soft Tissue Release")
OR TITLE-ABS-KEY("Soft-tissue release")
OR TITLE-ABS-KEY("release")
OR TITLE-ABS-KEY("Detachment")
OR TITLE-ABS-KEY("Varus derotation")
OR TITLE-ABS-KEY("femoral derotation")
OR TITLE-ABS-KEY("acetabular")
OR TITLE-ABS-KEY("Dega")
OR TITLE-ABS-KEY("Pemberton")
OR TITLE-ABS-KEY("reduction")
OR TITLE-ABS-KEY("lengthening")
OR TITLE-ABS-KEY("tenotomy")
OR INDEXTERMS("tenotomy")
OR INDEXTERMS("myotomy")
OR TITLE-ABS-KEY("femoral shortening")
OR TITLE-ABS-KEY("arthroplasty")
OR INDEXTERMS("arthroplasty")
OR TITLE-ABS-KEY("Colonna")
OR TITLE-ABS-KEY("resection")
OR TITLE-ABS-KEY("Girdlestone")
OR TITLE-ABS-KEY("hip resurfacing")
OR TITLE-ABS-KEY("THA")
OR TITLE-ABS-KEY("hemiarthroplasty")
OR TITLE-ABS-KEY("hip replacement*")
OR TITLE-ABS-KEY("hemiepiphysiodesis")
OR TITLE-ABS-KEY("growth modulation")
OR TITLE-ABS-KEY("guided growth")
OR TITLE-ABS-KEY("physeal stapling")
OR TITLE-ABS-KEY("botulinum toxin")
OR TITLE-ABS-KEY("botox")
OR INDEXTERMS("botox")
OR TITLE-ABS-KEY("neurotoxin")
OR TITLE-ABS-KEY("AbobotulinumtoxinA")
OR TITLE-ABS-KEY("BoNT")
OR TITLE-ABS-KEY("toxin injection")
OR TITLE-ABS-KEY("Hip reconstruction")
OR TITLE-ABS-KEY("Hip surgery")
OR TITLE-ABS-KEY("Closed physis")
OR TITLE-ABS-KEY("closed triradiate cartilage")
OR TITLE-ABS-KEY("salvage surgery")
OR TITLE-ABS-KEY("Chiari pelvic")

OR TITLE-ABS-KEY(“selective dorsal rhizotomy”)

OR TITLE-ABS-KEY(“selective posterior rhizotomy”)
)

**EMBASE:**

(

'cerebral palsy'/exp

OR 'cerebral palsy'

)

AND

 (

'Spastic hip'

OR 'Hip dislocation'

OR 'dislocated hip'

OR 'hip joint dislocation'

OR 'Hip displacement*'

OR 'hip instability'

OR 'Hip subluxation'

OR 'hip joint subluxation'

OR 'Hip lateralization'

OR 'acetabular reorientation'

OR 'femoral head medialization'

OR 'Hip dysplasia'

OR 'hip joint dysplasia'

OR 'Femoral head dislocation'

OR 'dislocated femoral head'

OR 'Femoral head displacement'

OR 'Femoral head subluxation'

OR 'femoral head insurability'

OR 'Femoral head lateralization'

OR 'femoral head dysplasia'

OR 'Hip displacement'

OR 'hip contracture'

)

AND

(

‘child'/exp

OR ‘infant'/exp

OR ‘pediatric'/exp

OR kid

OR Adolescent

OR baby

OR toddler

OR Neonate

OR Juvenile

OR Minor

OR 'Young patient'

OR 'Preschool'

)

AND

(

‘Surg'/exp

OR 'Procedure'

OR 'Operation'

OR 'Operative'

OR 'Intervention'

OR treatment/exp

OR method/exp

OR 'strateg*'

OR 'technique'

OR 'VDRO'

OR 'Osteotomy'

OR 'Soft Tissue Release'

OR 'Soft-tissue release'

OR 'release'

OR 'Detachment'

OR 'Varus derotation'

OR 'femoral derotation'

OR 'acetabular'

OR 'Dega'

OR 'Pemberton'

OR 'reduction'

OR 'lengthening'

OR 'tenotomy'

OR myotomy/exp

OR 'femoral shortening'

OR arthroplasty/exp

OR 'Colonna'

OR 'resection'

OR 'Girdlestone'

OR 'hip resurfacing'

OR 'THA'

OR 'hemiarthroplasty'

OR 'hip replacement*'

OR 'hemiepiphysiodesis'

OR 'growth modulation'

OR 'guided growth'

OR 'physeal stapling'

OR 'botulinum toxin'

OR 'botox'

OR 'neurotoxin'

OR 'AbobotulinumtoxinA'

OR 'BoNT'

OR 'toxin injection'

OR 'Hip reconstruction'

OR 'Hip surgery'

OR 'Closed physis'

OR 'closed triradiate cartilage'

OR 'salvage surgery'

OR 'Chiari pelvic'

OR ‘selective dorsal rhizotomy’

OR ‘selective posterior rhizotomy’

)

**COCHRANE CENTRAL:**

(

("cerebral palsy"):ti,ab,kw

)

AND

(

(“Spastic hip”):ti,ab,kw

OR (“Hip dislocation”):ti,ab,kw

OR (“dislocated hip”):ti,ab,kw

OR (“hip joint dislocation”):ti,ab,kw

OR (“Hip displacement*”):ti,ab,kw

OR (“hip instability”):ti,ab,kw

OR (“Hip subluxation”):ti,ab,kw

OR (“hip joint subluxation”):ti,ab,kw

OR (“Hip lateralization”):ti,ab,kw

OR (“acetabular reorientation”):ti,ab,kw

OR (“femoral head medialization”):ti,ab,kw

OR (“Hip dysplasia”):ti,ab,kw

OR (“hip joint dysplasia”):ti,ab,kw

OR (“Femoral head dislocation”):ti,ab,kw

OR (“dislocated femoral head”):ti,ab,kw

OR (“Femoral head displacement”):ti,ab,kw

OR (“Femoral head subluxation”):ti,ab,kw

OR (“femoral head insurability”):ti,ab,kw

OR (“Femoral head lateralization”):ti,ab,kw

OR (“femoral head dysplasia”):ti,ab,kw

OR (“Hip displacement”):ti,ab,kw

OR (“hip contracture”):ti,ab,kw

)

AND

(

(child*):ti,ab,kw

OR (infant*):ti,ab,kw

OR (pediatric*):ti,ab,kw

OR (kid):ti,ab,kw

OR (Adolescent):ti,ab,kw

OR (baby):ti,ab,kw

OR (toddler):ti,ab,kw

OR (Neonate):ti,ab,kw

OR (Juvenile):ti,ab,kw

OR (Minor):ti,ab,kw

OR ("Young patient"):ti,ab,kw

OR ("Preschool"):ti,ab,kw

)

AND

(

(Surg*):ti,ab,kw

OR (Procedure):ti,ab,kw

OR (Operation):ti,ab,kw

OR (Operative):ti,ab,kw

OR (Intervention):ti,ab,kw

OR (treatment):ti,ab,kw

OR (method):ti,ab,kw

OR (strateg*):ti,ab,kw

OR (technique):ti,ab,kw

OR ("VDRO"):ti,ab,kw

OR (Osteotomy):ti,ab,kw

OR ("Soft Tissue Release"):ti,ab,kw

OR ("Soft-tissue release"):ti,ab,kw

OR (release):ti,ab,kw OR

(Detachment):ti,ab,kw

OR ("Varus derotation"):ti,ab,kw

OR ("femoral derotation"):ti,ab,kw

OR (acetabular):ti,ab,kw

OR (Dega):ti,ab,kw

OR (Pemberton):ti,ab,kw

OR (reduction):ti,ab,kw

OR (lengthening):ti,ab,kw

OR (tenotomy):ti,ab,kw

OR (myotomy):ti,ab,kw

OR (“femoral shortening”):ti,ab,kw

OR (arthroplasty):ti,ab,kw

OR (colonna):ti,ab,kw

OR (resection):ti,ab,kw

OR (Girdlestone):ti,ab,kw

OR (“hip resurfacing”):ti,ab,kw

OR (THA):ti,ab,kw

OR (hemiarthroplasty):ti,ab,kw

OR (“hip replacement*”):ti,ab,kw

OR (hemiepiphysiodesis):ti,ab,kw

OR (“growth modulation”):ti,ab,kw

OR (“guided growth”):ti,ab,kw

OR (“physeal stapling”):ti,ab,kw

OR (“botulinum toxin”):ti,ab,kw

OR (botox):ti,ab,kw

OR (neurotoxin):ti,ab,kw

OR (AbobotulinumtoxinA):ti,ab,kw

OR (BoNT):ti,ab,kw

OR (“toxin injection”):ti,ab,kw

OR (“Hip reconstruction”):ti,ab,kw

OR (“Hip surgery”):ti,ab,kw

OR (“Closed physis”):ti,ab,kw

OR (“closed triradiate cartilage”):ti,ab,kw

OR (“salvage surgery”):ti,ab,kw

OR (“Chiari pelvic”):ti,ab,kw

OR (“selective dorsal rhizotomy”):ti,ab,kw

OR (“selective posterior rhizotomy”):ti,ab,kw

)

**WoS:**

(

TS=("cerebral palsy")

)

AND

(

TS=(“Spastic hip”)

OR TS=(“Hip dislocation”)

OR TS=(“dislocated hip”)

OR TS=(“hip joint dislocation”)

OR TS=(“Hip displacement*”)

OR TS=(“hip instability”)

OR TS=(“Hip subluxation”)

OR TS=(“hip joint subluxation”)

OR TS=(“Hip lateralization”)

OR TS=(“acetabular reorientation”)

OR TS=(“femoral head medialization”)

OR TS=(“Hip dysplasia”)

OR TS=(“hip joint dysplasia”)

OR TS=(“Femoral head dislocation”)

OR TS=(“dislocated femoral head”)

OR TS=(“Femoral head displacement”)

OR TS=(“Femoral head subluxation”)

OR TS=(“femoral head insurability”)

OR TS=(“Femoral head lateralization”)

OR TS=(“femoral head dysplasia”)

OR TS=(“Hip displacement”)

OR TS=(“hip contracture”)

)

AND

(

TS=(child*)

OR TS=(infant*)

OR TS=(pediatric*)

OR TS=(kid)

OR TS=(Adolescent)

OR TS=(baby)

OR TS=(toddler)

OR TS=(Neonate)

OR TS=(Juvenile)

OR TS=(Minor)

OR TS=("Young patient")

OR TS=("Preschool")

)

AND

(

TS=(Surg*)

OR TS=(Procedure)

OR TS=(Operation)

OR TS=(Operative)

OR TS=(Intervention)

OR TS=(treatment)

OR TS=(method)

OR TS=(strateg*)

OR TS=(technique)

OR TS=("VDRO")

OR TS=(Osteotomy)

OR TS=("Soft Tissue Release")

OR TS=("Soft-tissue release")

OR TS=(release) OR

TS=(Detachment)

OR TS=("Varus derotation")

OR TS=("femoral derotation")

OR TS=(acetabular)

OR TS=(Dega)

OR TS=(Pemberton)

OR TS=(reduction)

OR TS=(lengthening)

OR TS=(tenotomy)

OR TS=(myotomy)

OR TS=(“femoral shortening”)

OR TS=(arthroplasty)

OR TS=(colonna)

OR TS=(resection)

OR TS=(Girdlestone)

OR TS=(“hip resurfacing”)

OR TS=(THA)

OR TS=(hemiarthroplasty)

OR TS=(“hip replacement*”)

OR TS=(hemiepiphysiodesis)

OR TS=(“growth modulation”)

OR TS=(“guided growth”)

OR TS=(“physeal stapling”)

OR TS=(“botulinum toxin”)

OR TS=(botox)

OR TS=(neurotoxin)

OR TS=(AbobotulinumtoxinA)

OR TS=(BoNT)

OR TS=(“toxin injection”)

OR TS=(“Hip reconstruction”)

OR TS=(“Hip surgery”)

OR TS=(“Closed physis”)

OR TS=(“closed triradiate cartilage”)

OR TS=(“salvage surgery”)

OR TS=(“Chiari pelvic”)

OR TS=(“selective dorsal rhizotomy”)

OR TS=(“selective posterior rhizotomy”)

)
