# Supplementary material for: Radiological outcomes of surgical techniques for spastic hip in cerebral palsy: a systematic review and meta-analysis
Source: J Orthop Traumatol. 2025 Feb 28;26:13. doi: 10.1186/s10195-025-00827-0 (PMC11871257; doi:10.1186/s10195-025-00827-0)
Supplement: Supplementary file 2 — Additional file 2. [file 10195_2025_827_MOESM2_ESM.docx]

**Appendix B (forest plots):**


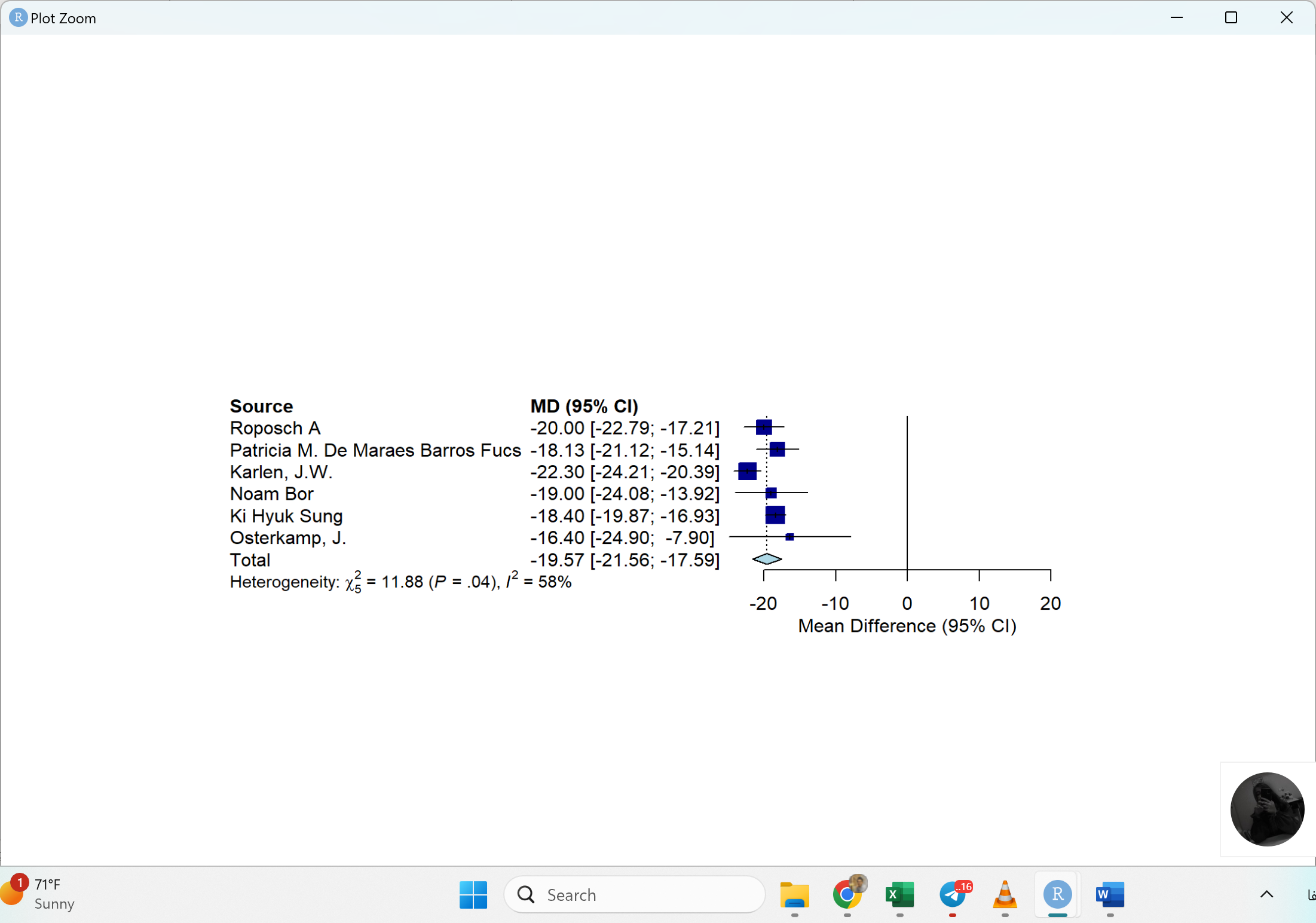


Supplementary Figure 1. Pelvic Osteotomy surgery, Acetabular index


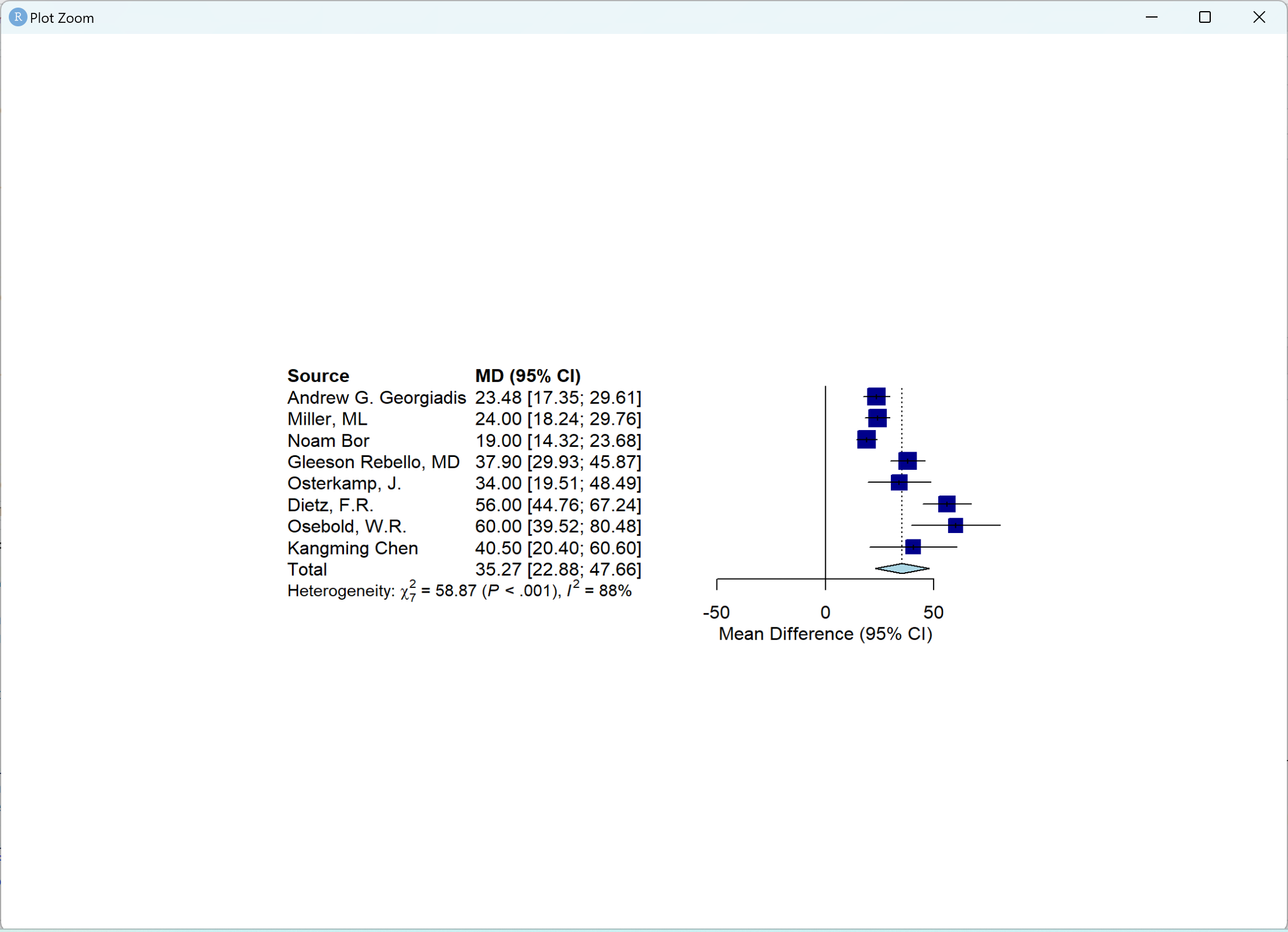


Supplementary Figure 2. Pelvic Osteotomy surgery, Center Edge Angle


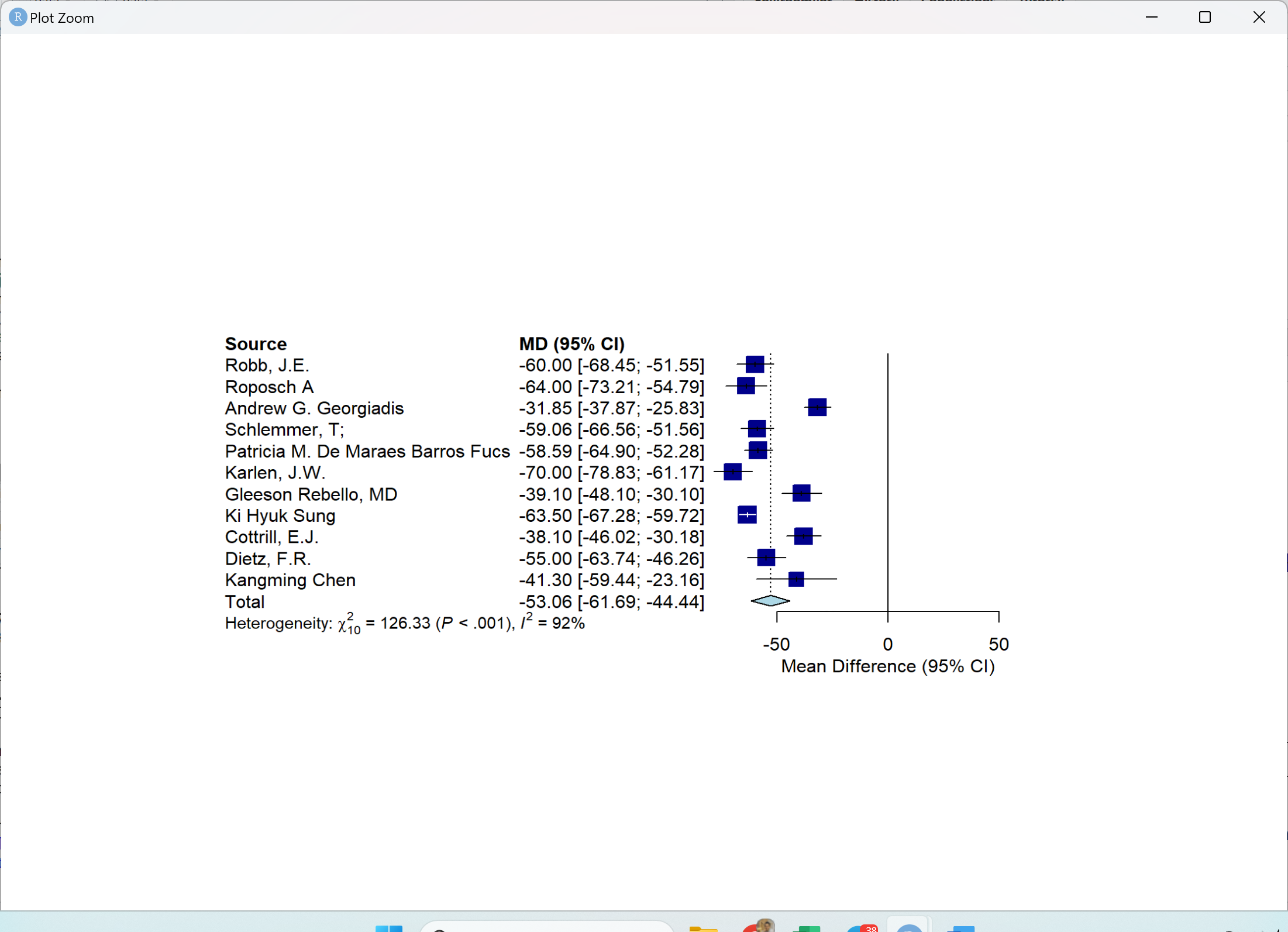


Supplementary Figure 3. Pelvic Osteotomy surgery, Migration Percentage


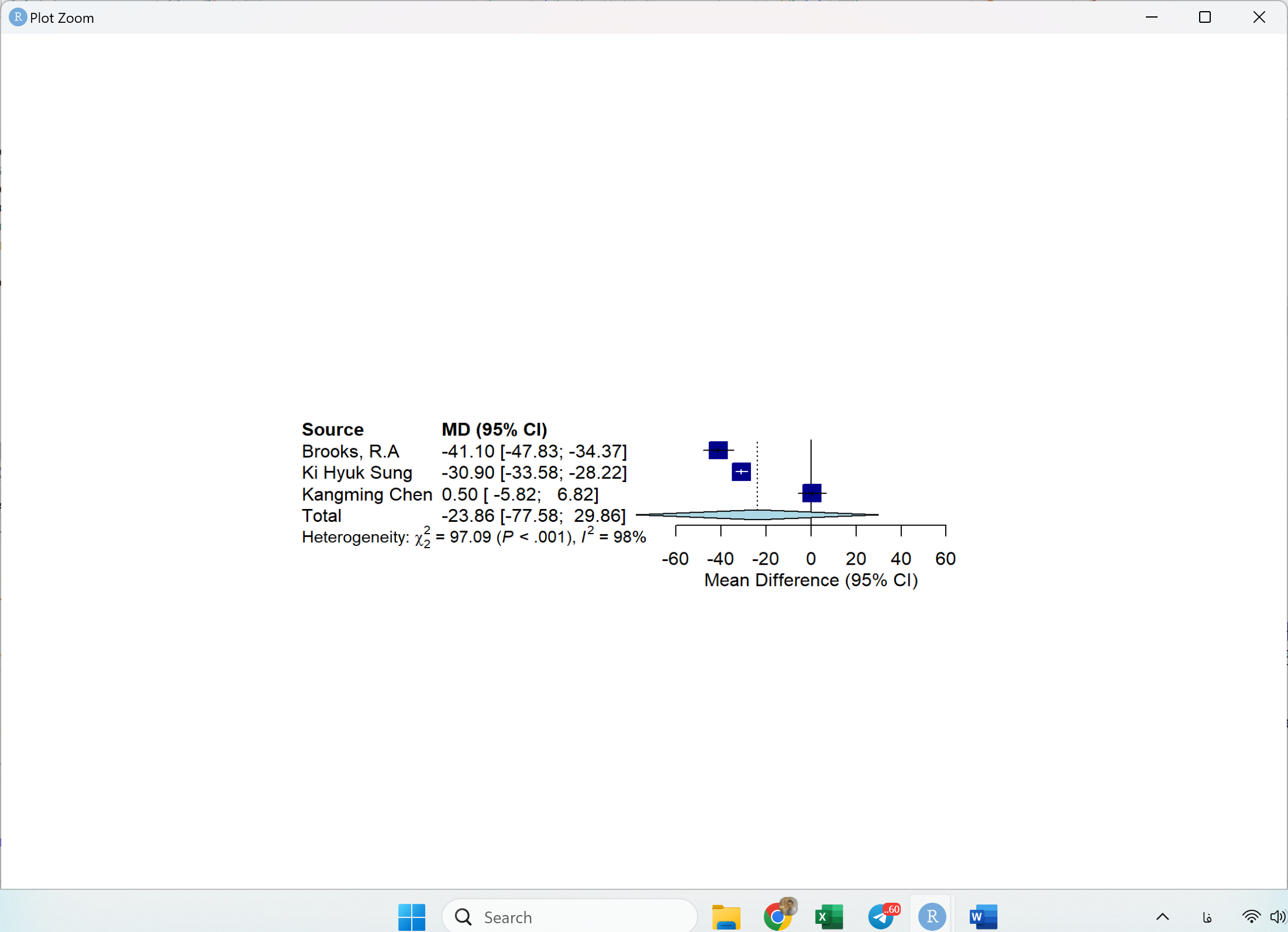


Supplementary Figure 4. Pelvic Osteotomy surgery, Neck Shaft Angle


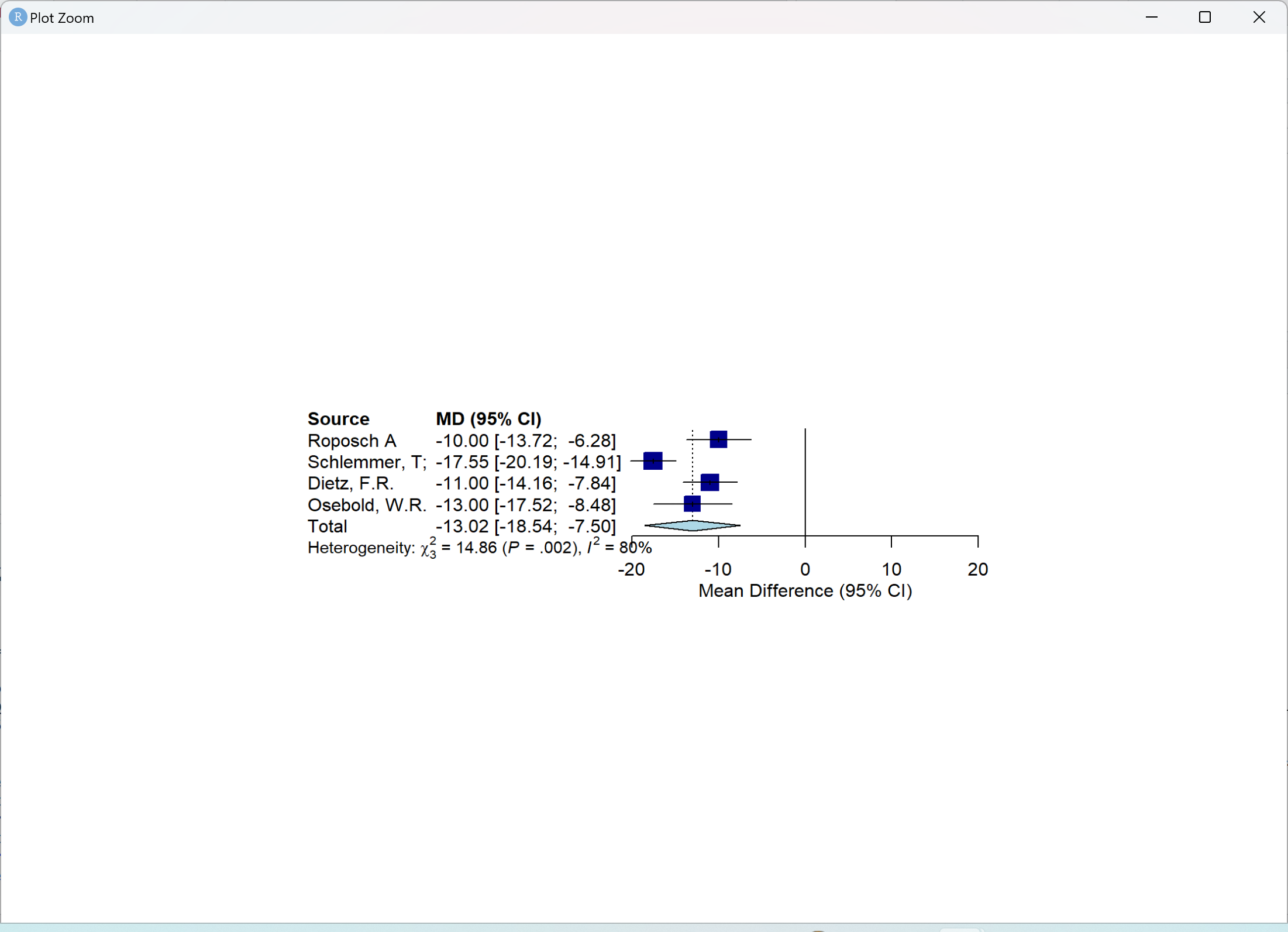


Supplementary Figure 5. Pelvic Osteotomy surgery, Sharp Angle


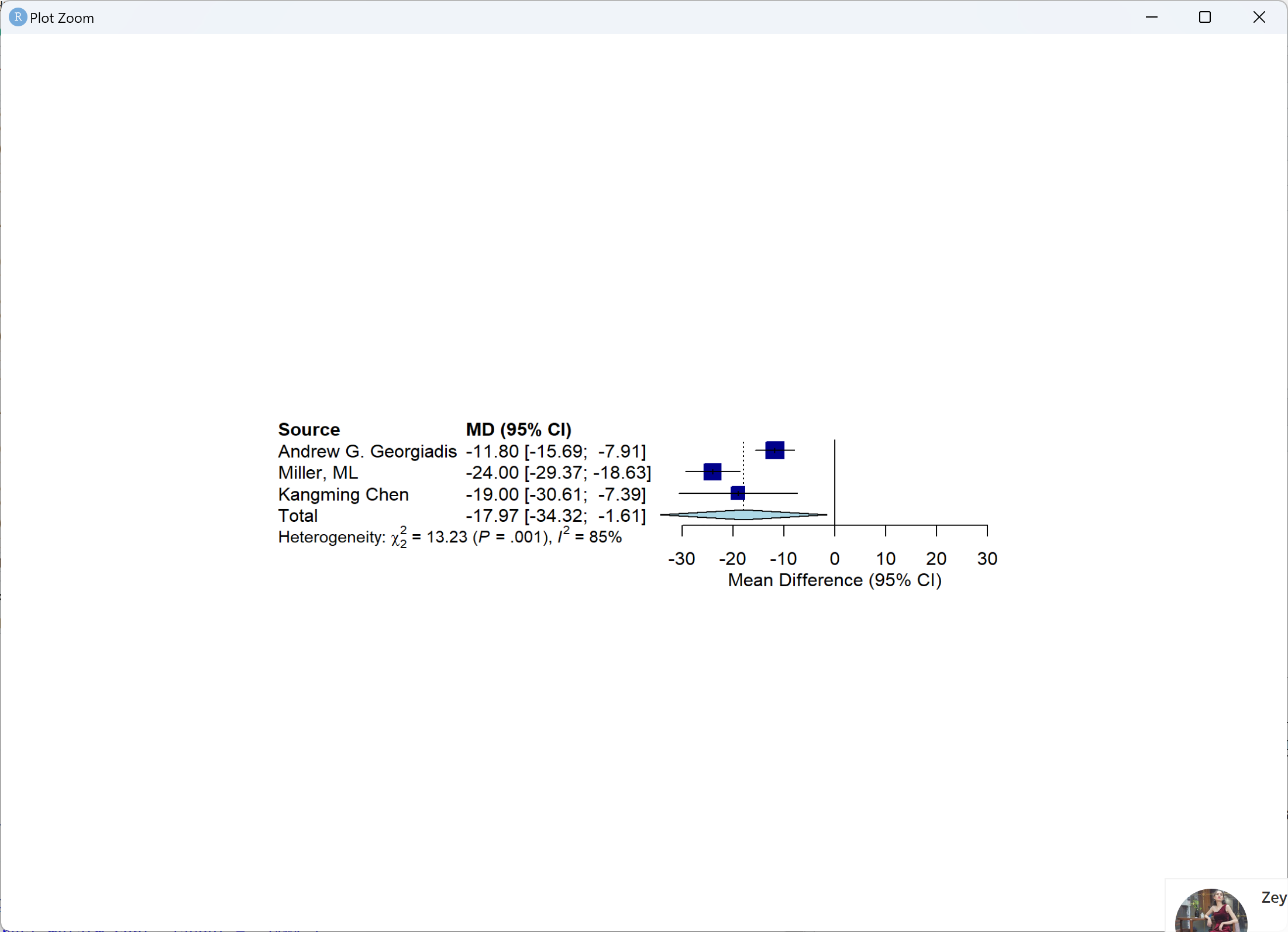


Supplementary Figure 6. Pelvic Osteotomy surgery, Tonnis Angle


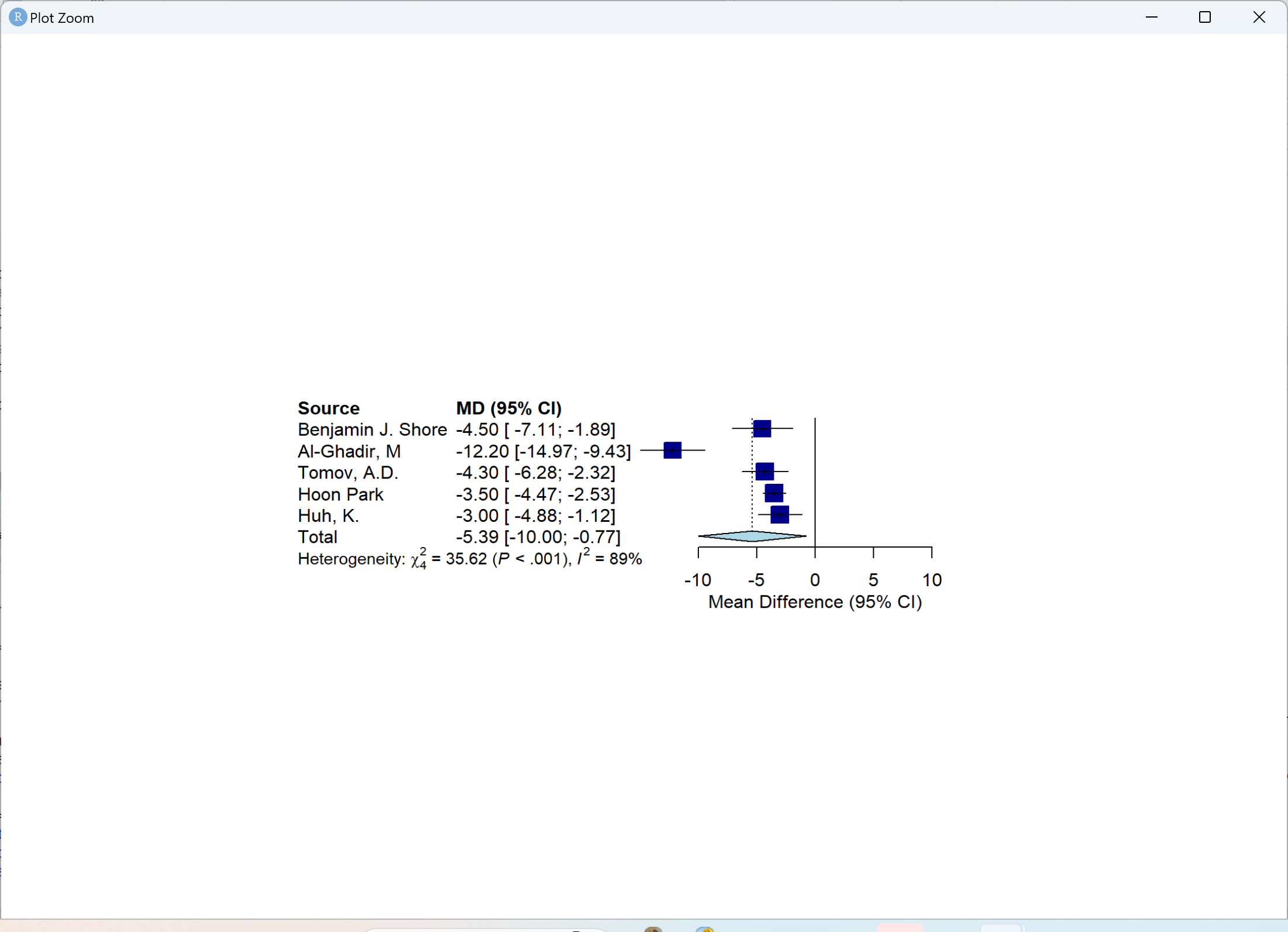


Supplementary Figure 7. Femur Osteotomy surgery, Acetabular index


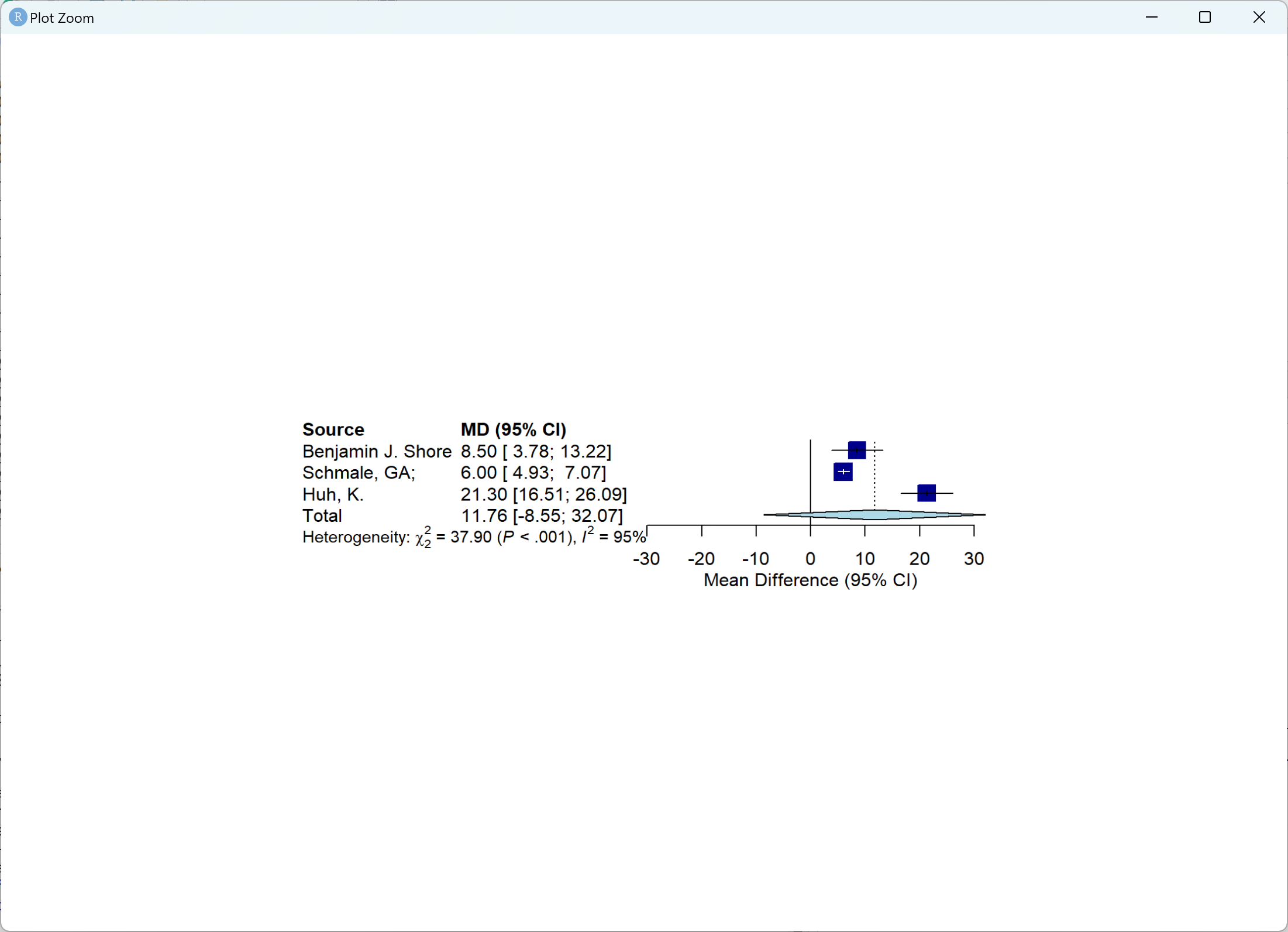


Supplementary Figure 8. Femur Osteotomy surgery, Center Edge Angle


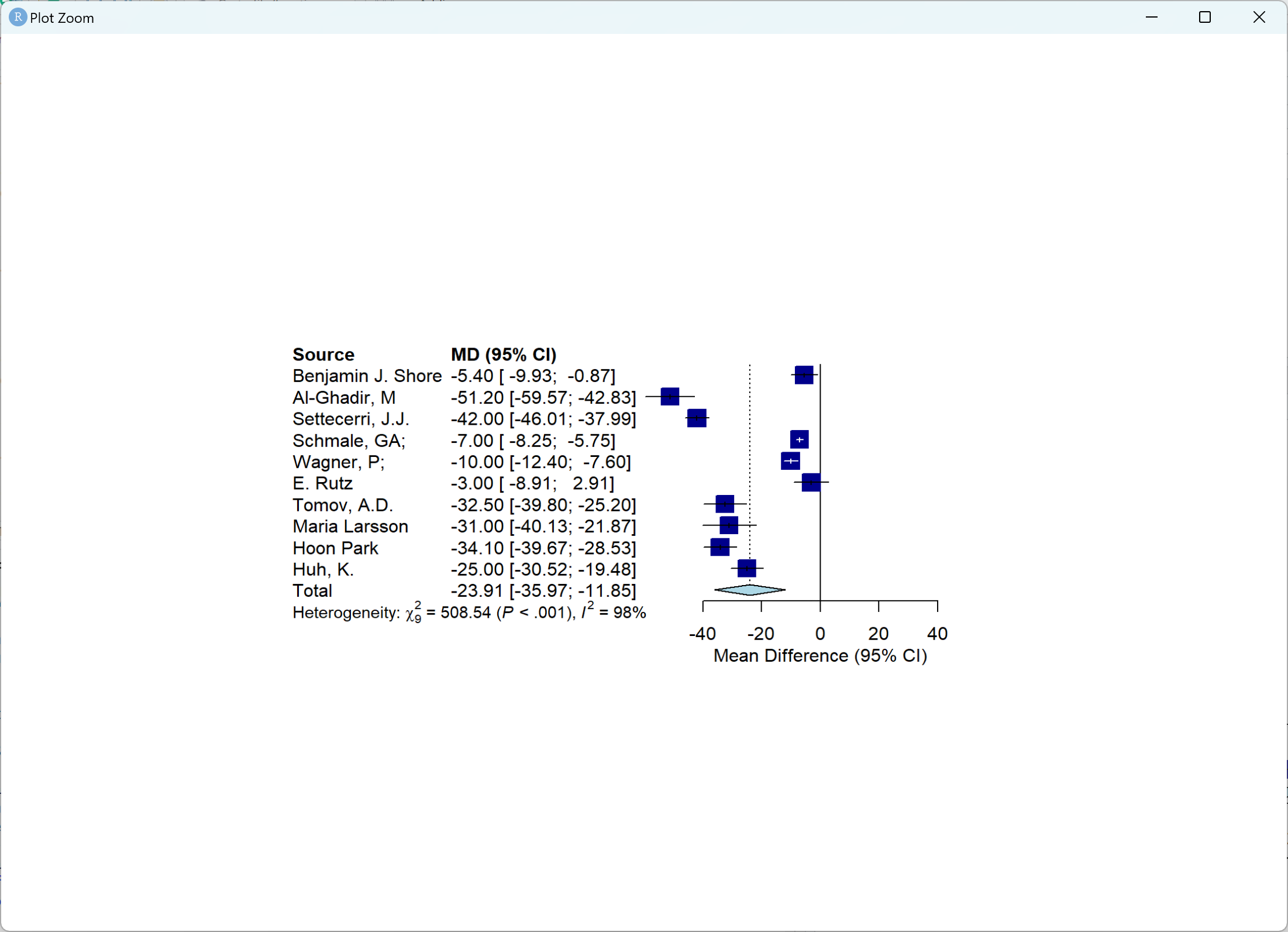


Supplementary Figure 9. Femur Osteotomy surgery, Migration Percentage


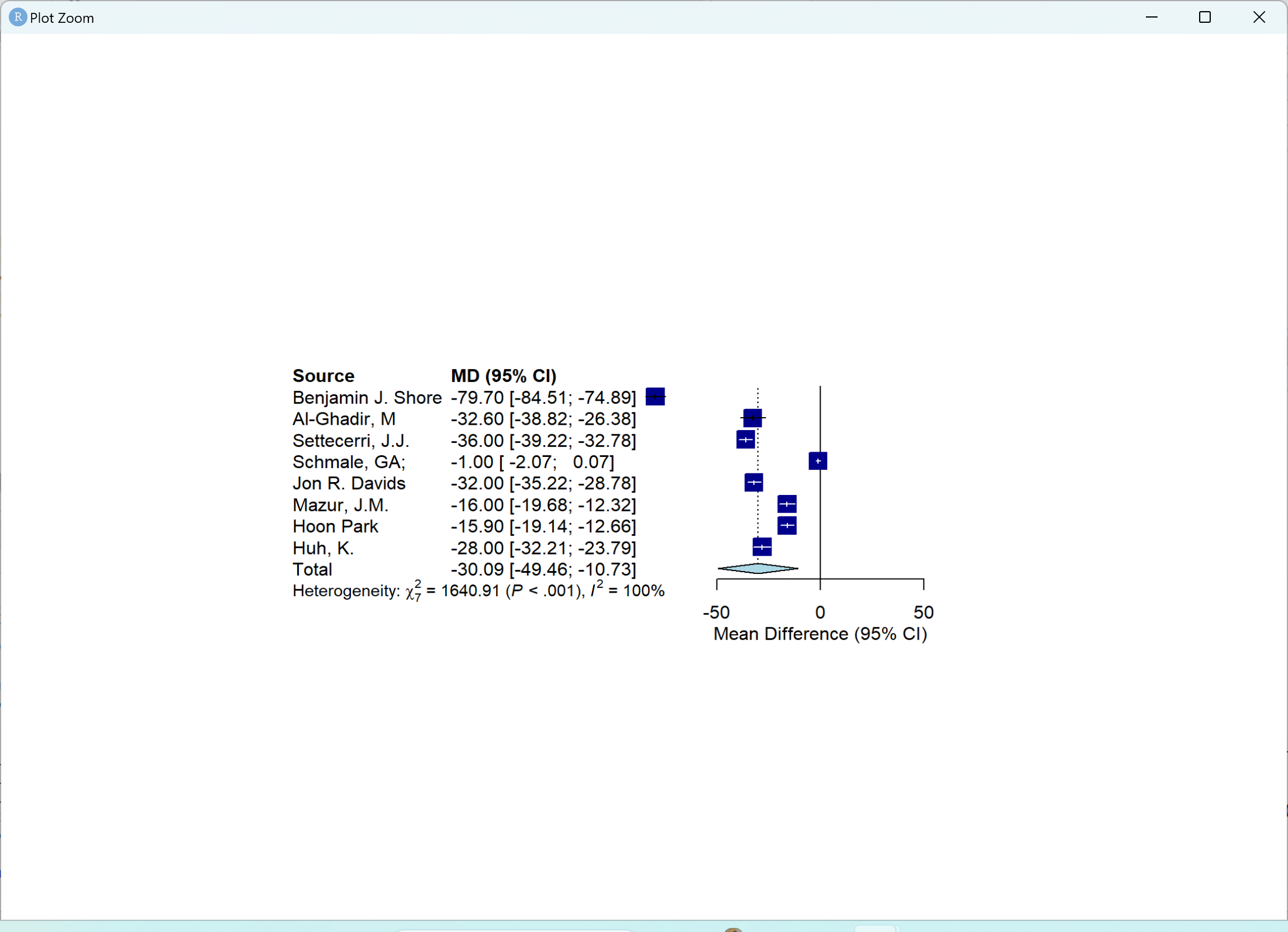


Supplementary Figure 10. Femur Osteotomy surgery, Neck Shaft Angle


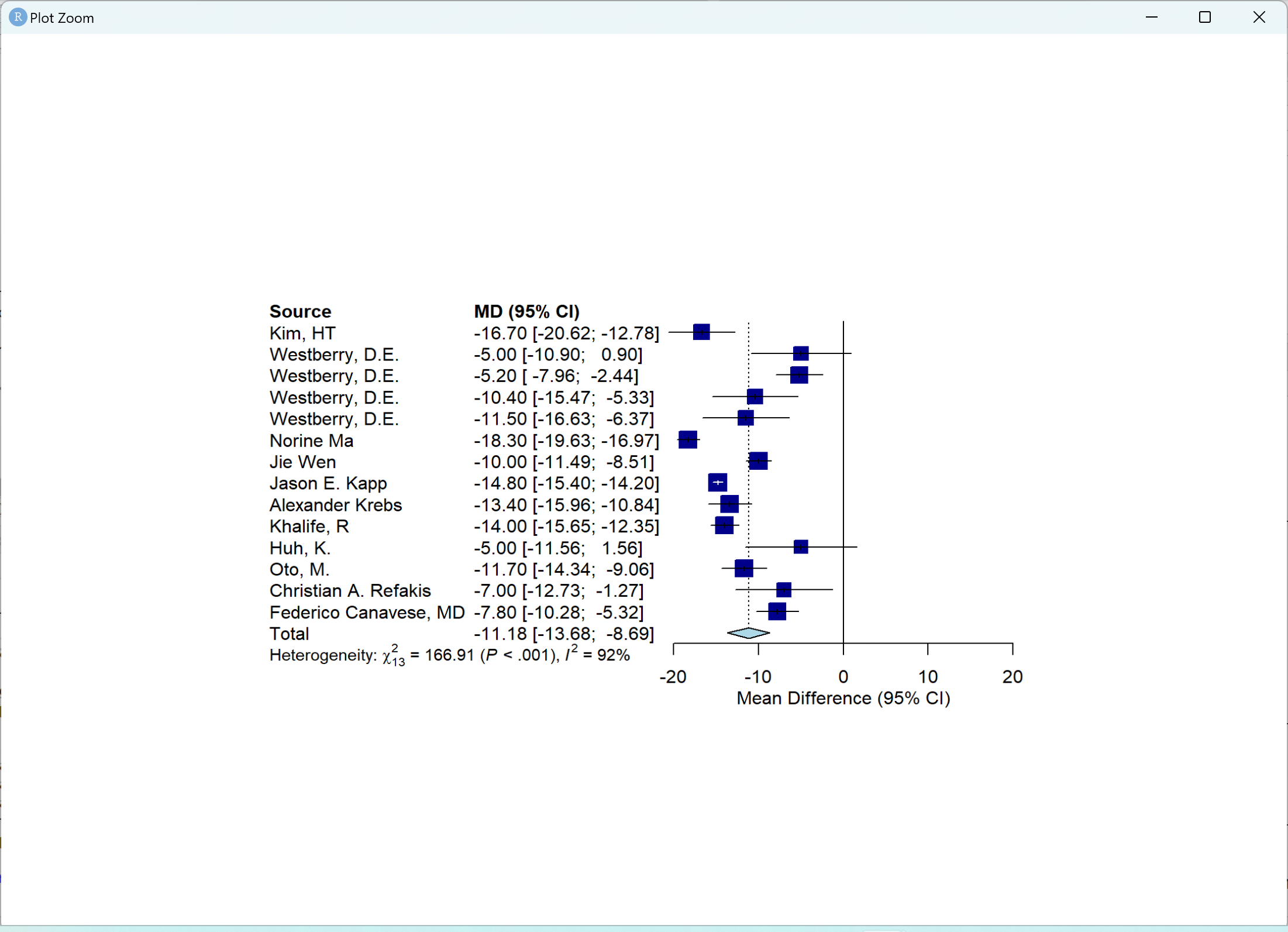


Supplementary Figure 11. Combination of Pelvic and Femur Osteotomy surgery, Acetabular Index


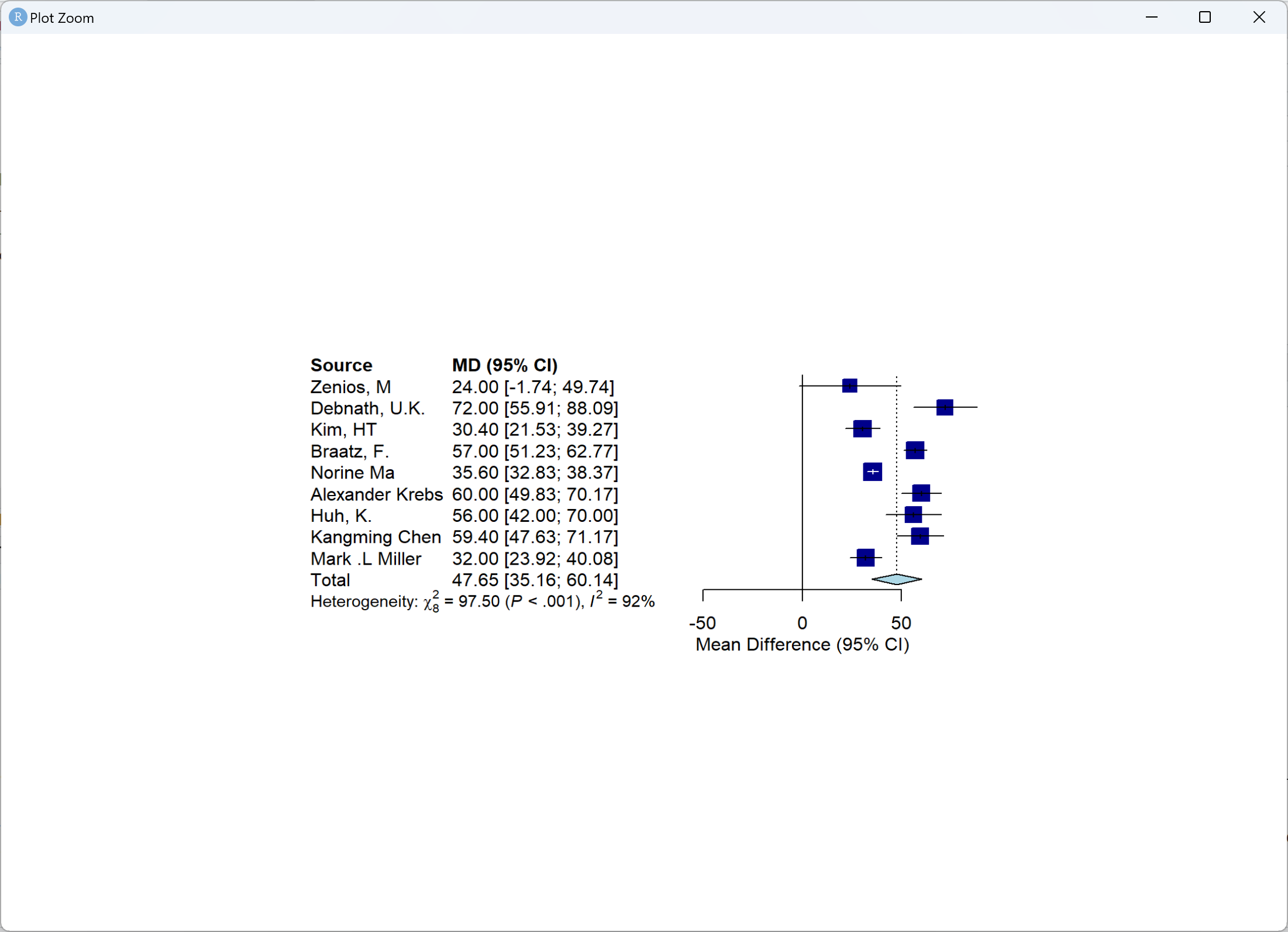


Supplementary Figure 12. Combination of Pelvic and Femur Osteotomy surgery, Center Edge Angle


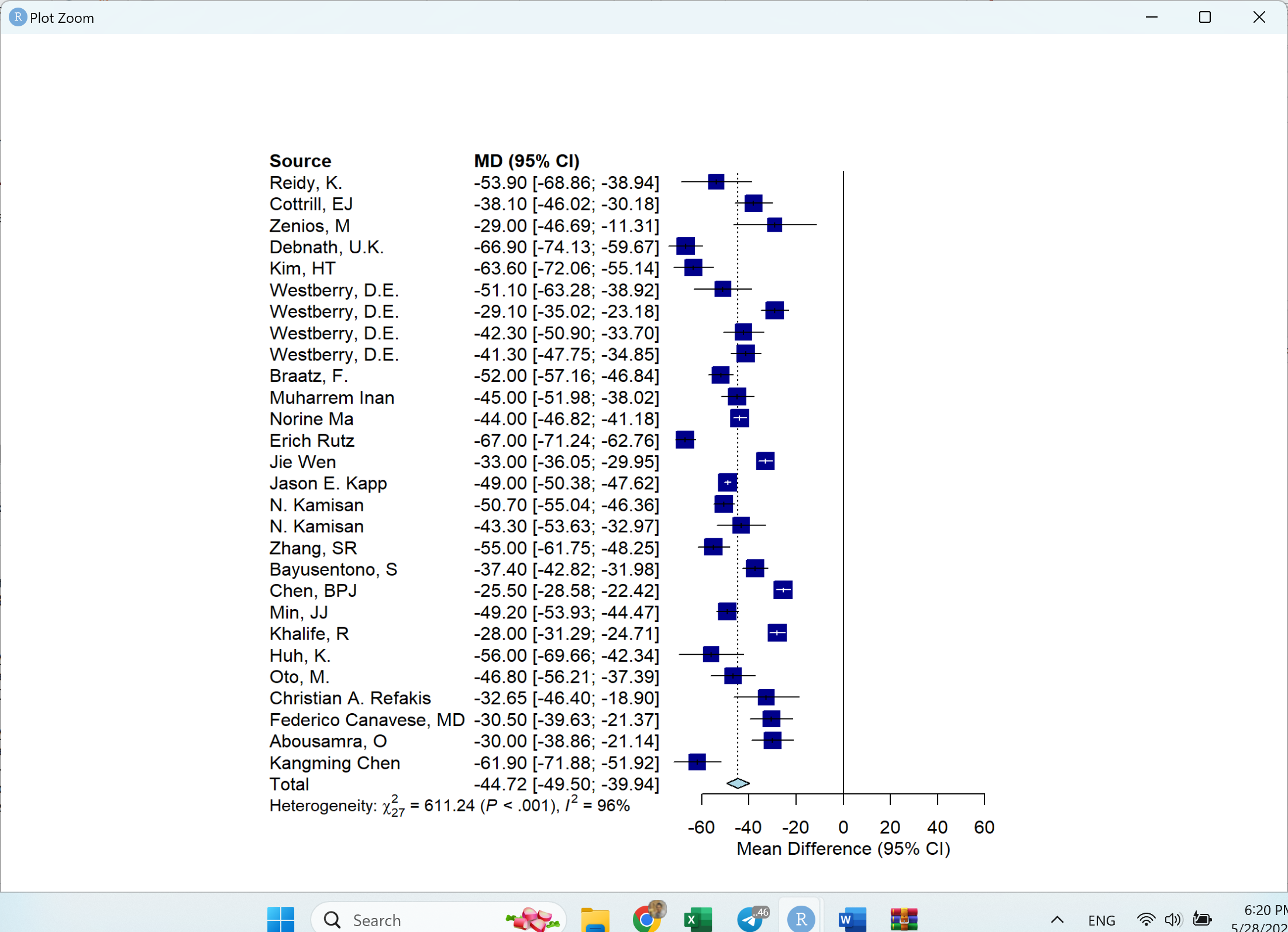


Supplementary Figure 13. Combination of Pelvic and Femur Osteotomy surgery, Migration Percentage


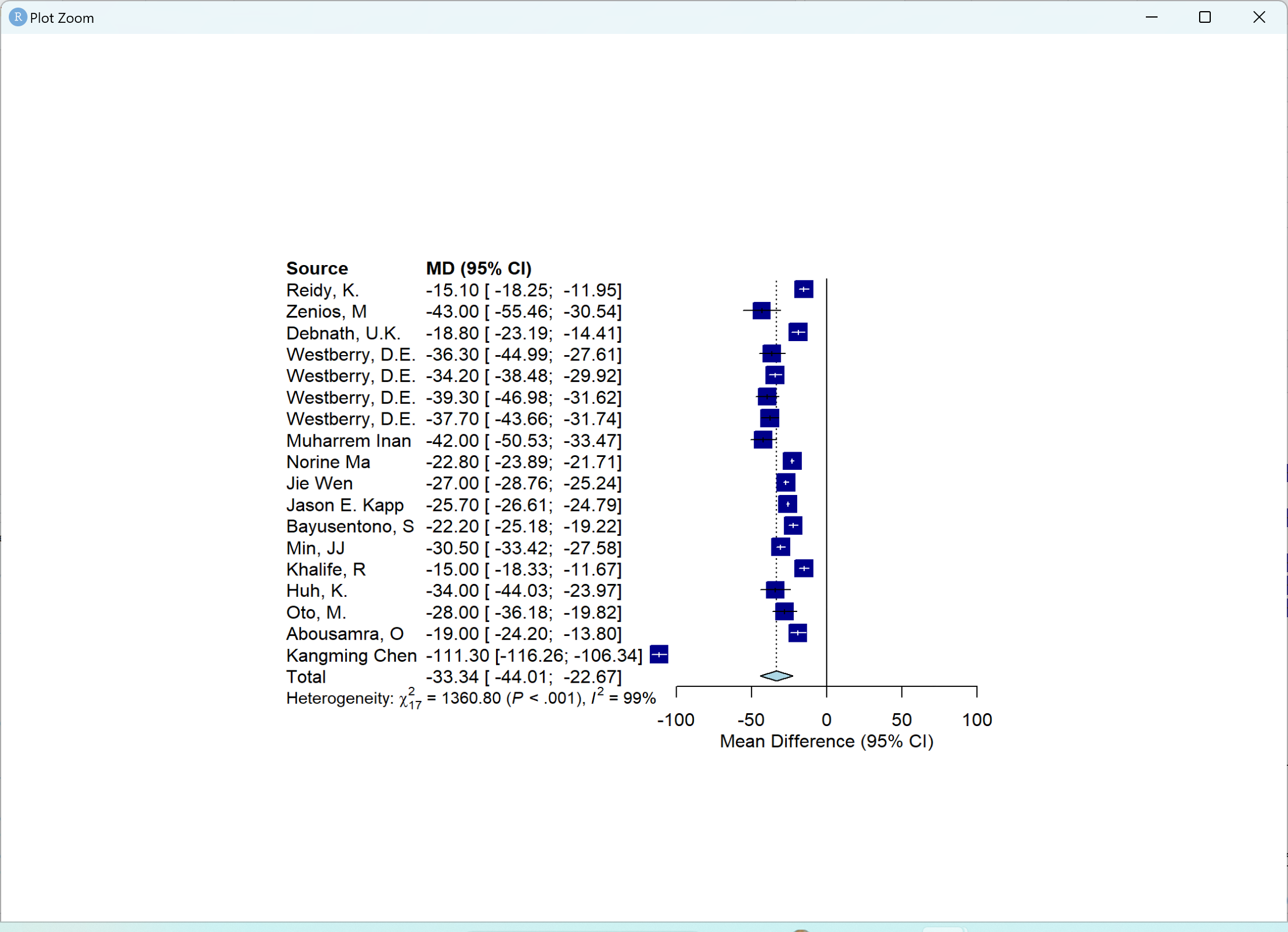


Supplementary Figure 14. Combination of Pelvic and Femur Osteotomy surgery, Neck Shaft Angle


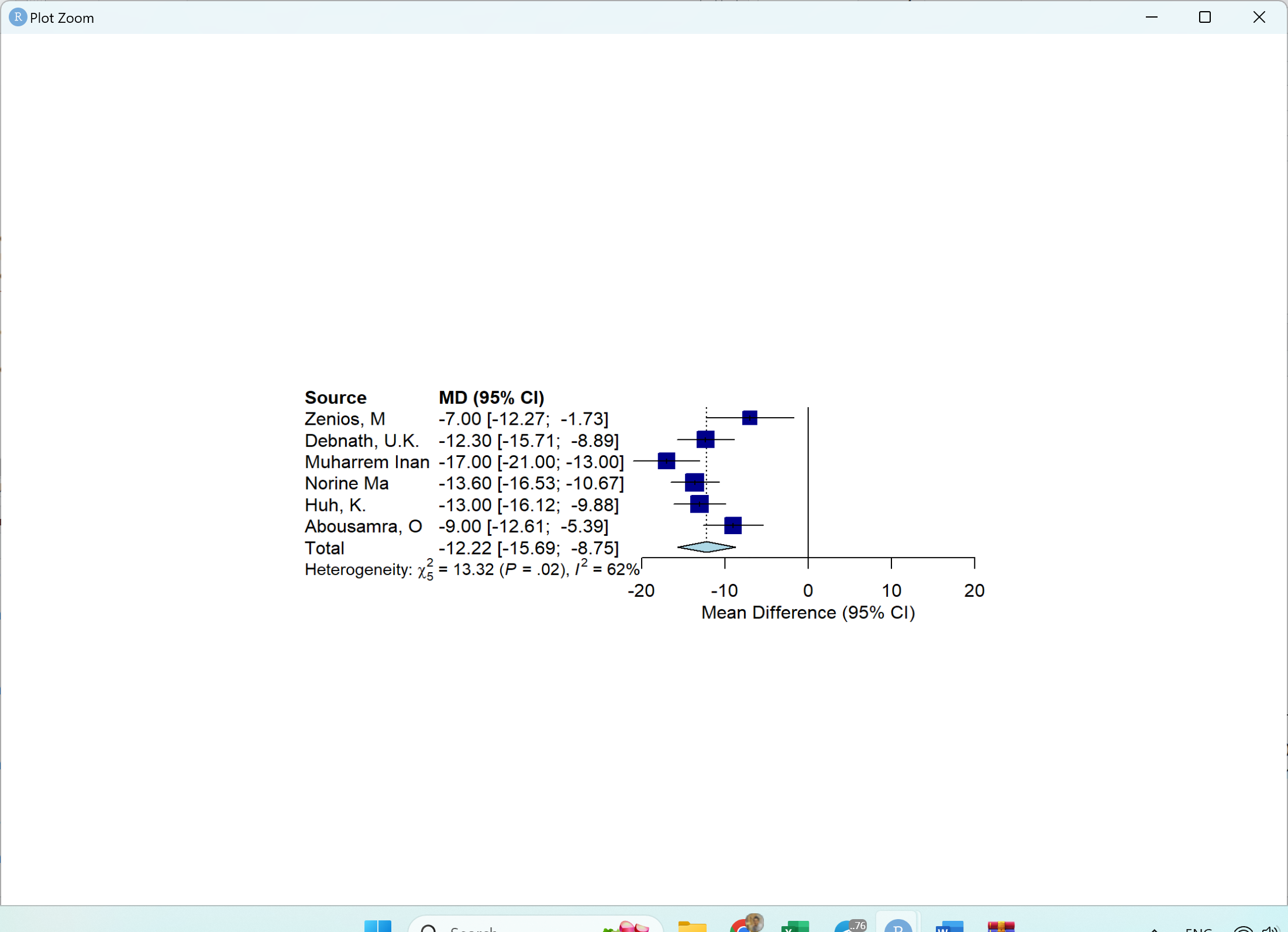


Supplementary Figure 15. Combination of Pelvic and Femur Osteotomy surgery, Sharp Angle


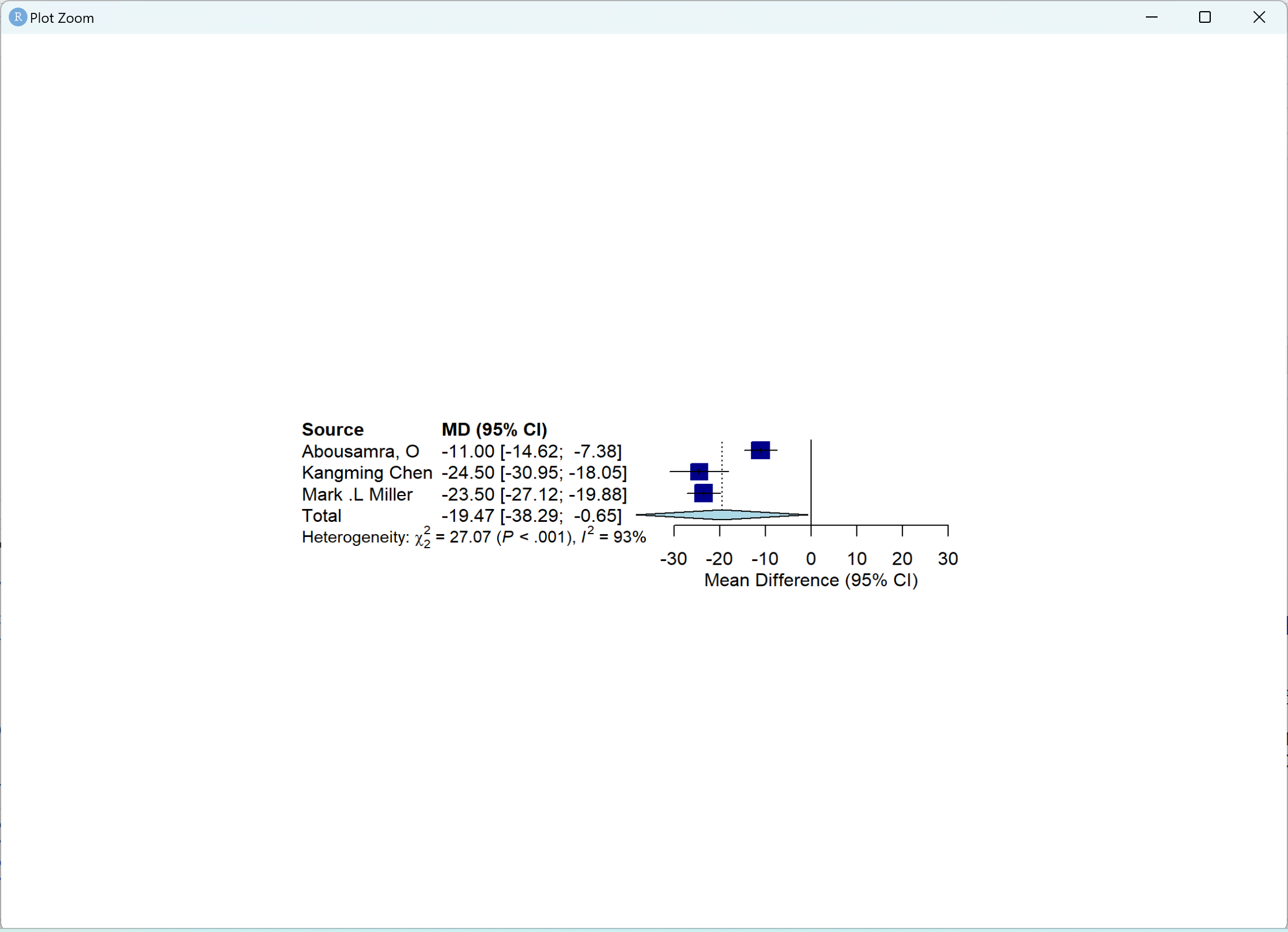


Supplementary Figure 16. Combination of Pelvic and Femur Osteotomy surgery, Tonnis Angle


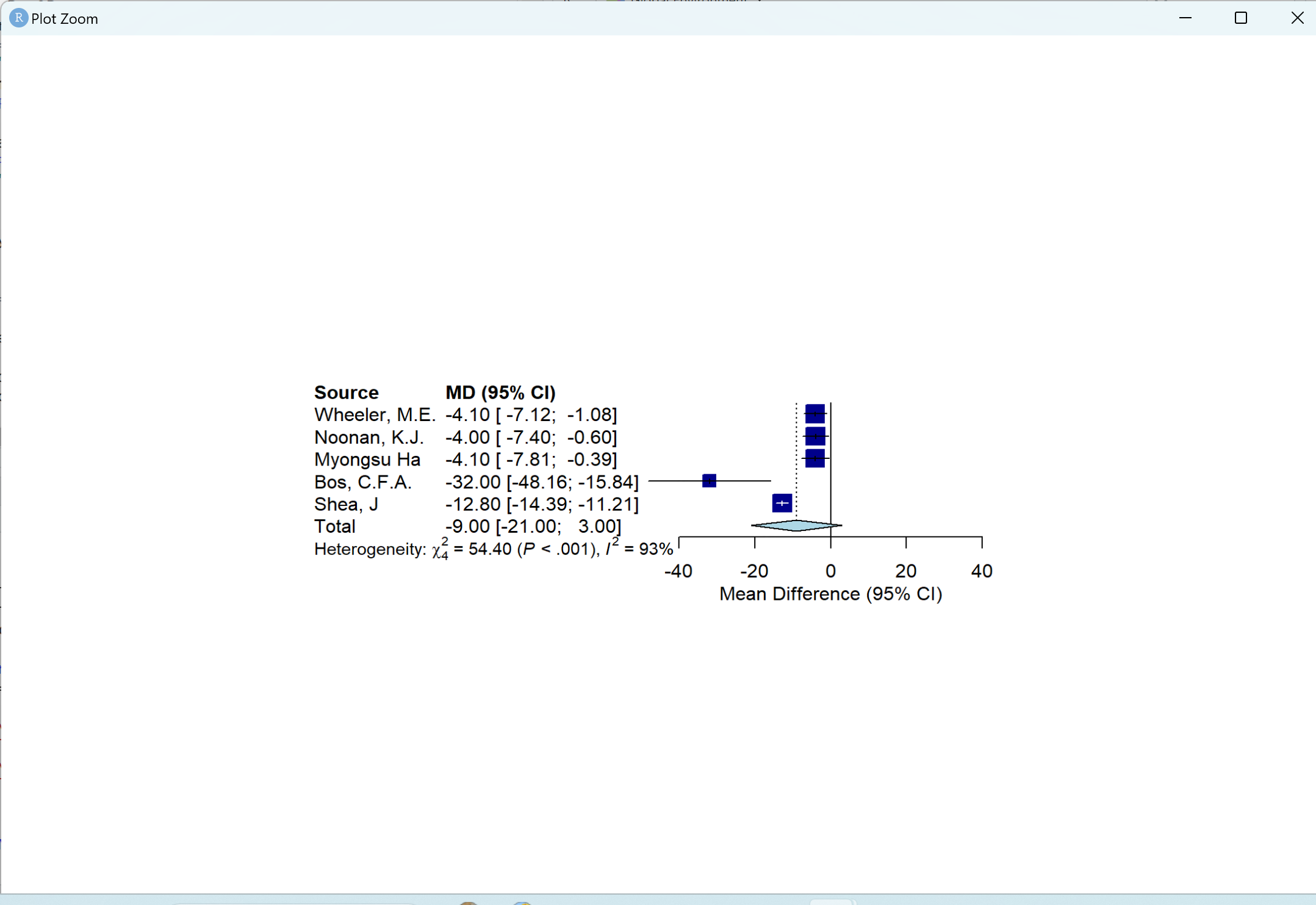


Supplementary Figure 17. Soft Tissue surgery, Acetabular Index


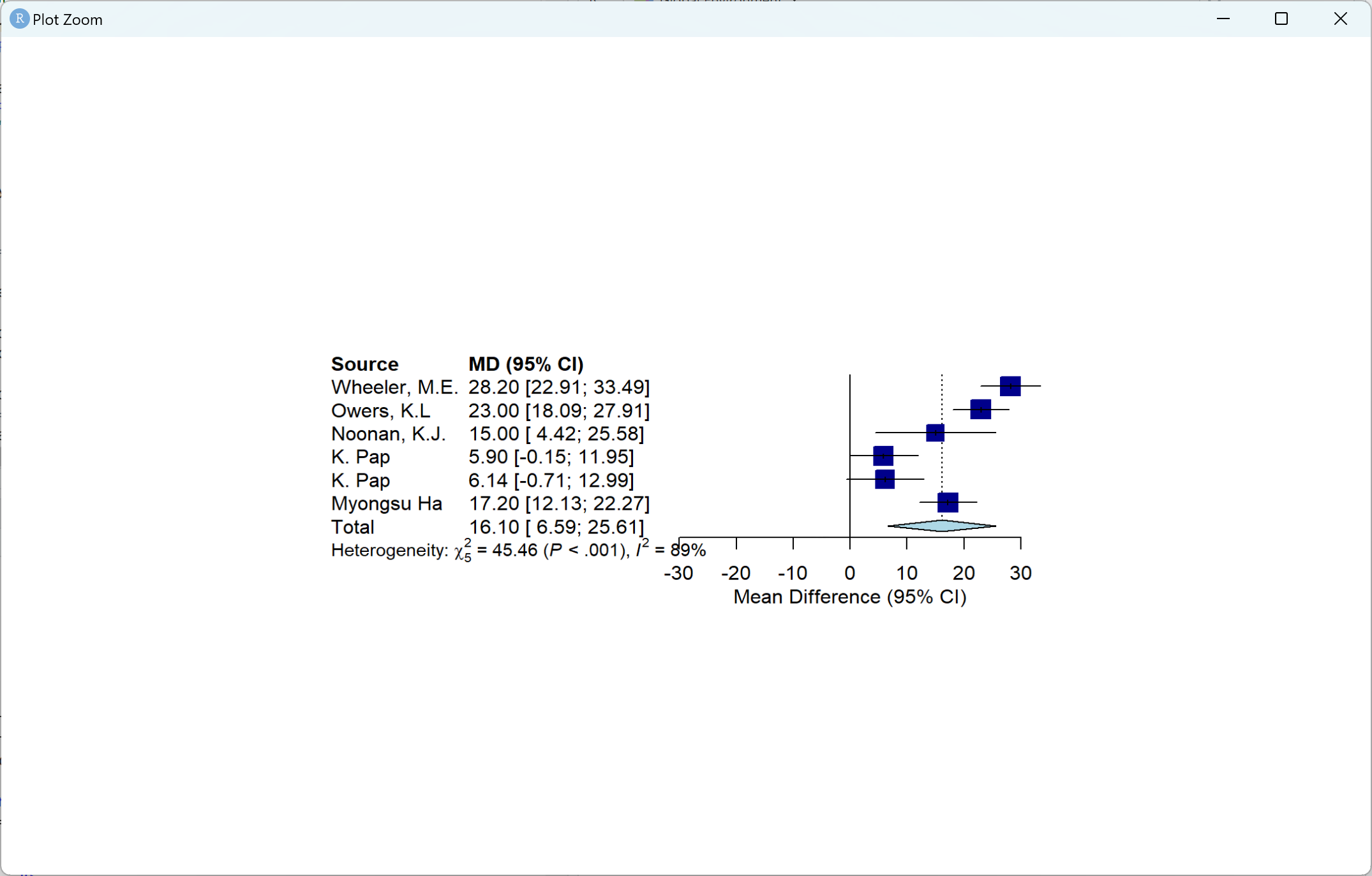


Supplementary Figure 18. Soft Tissue surgery, Center Edge Angle


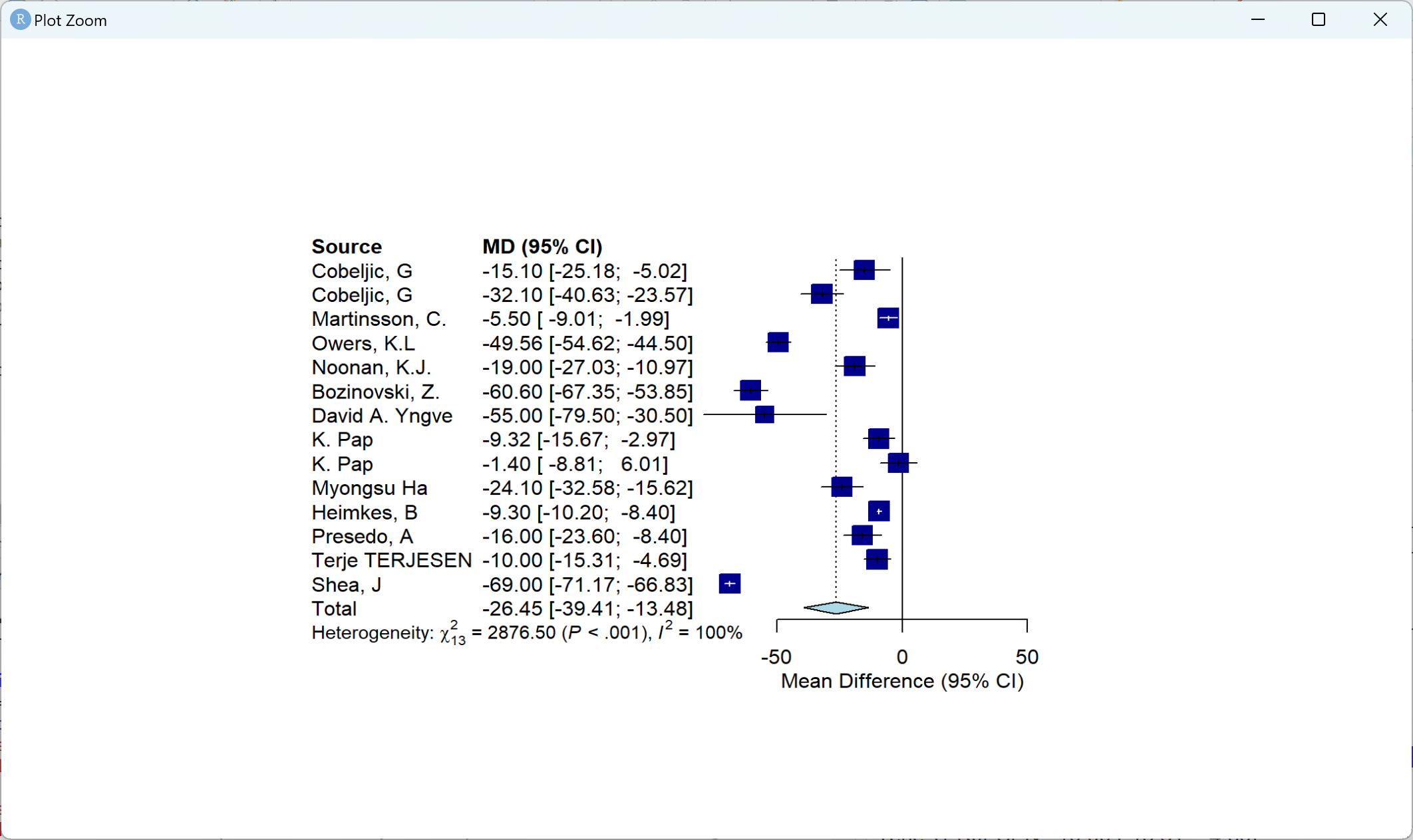


Supplementary Figure 19. Soft Tissue surgery, Migration Percentage


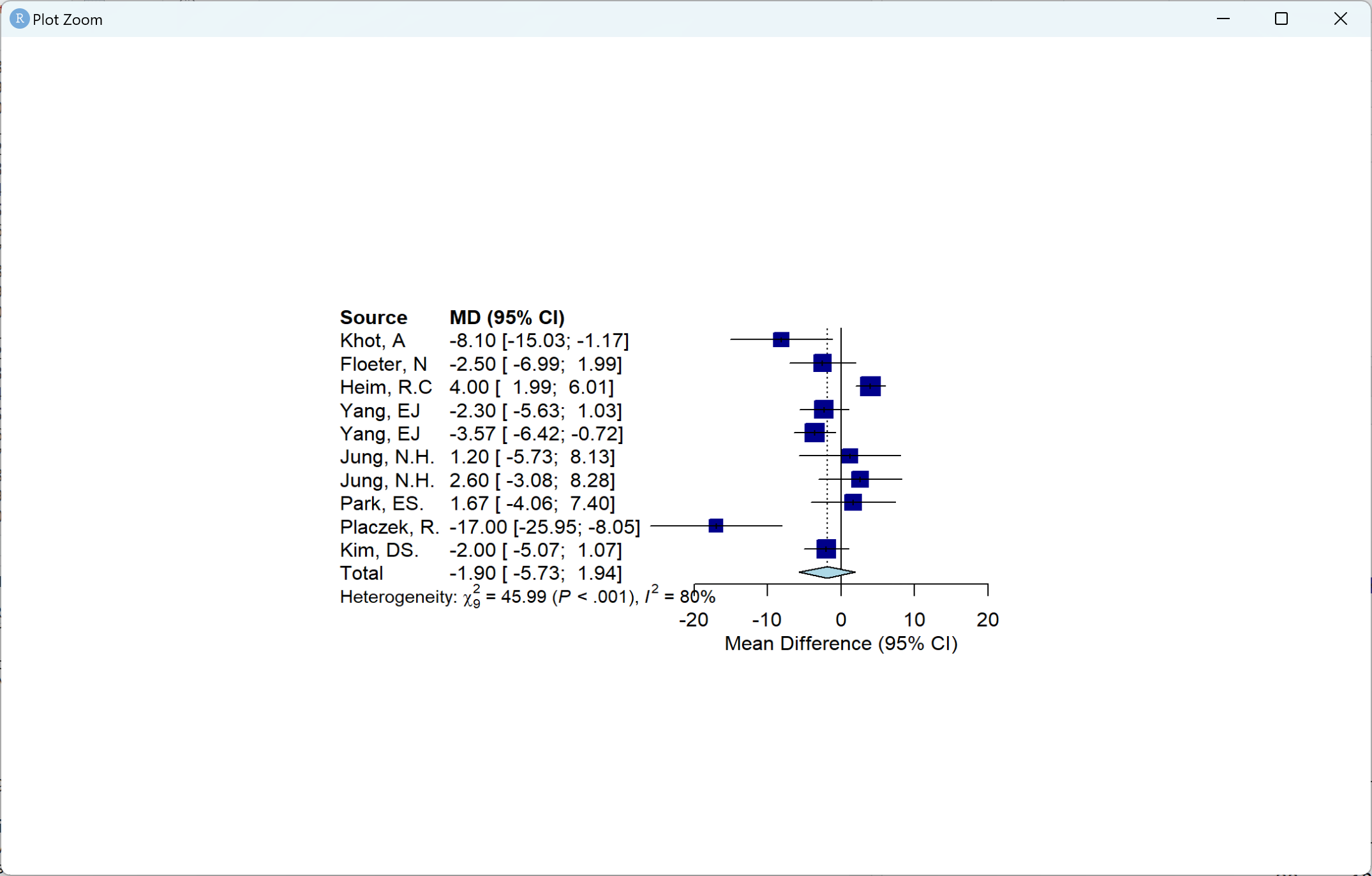


Supplementary Figure 20. Tone decrease, Migration Percentage


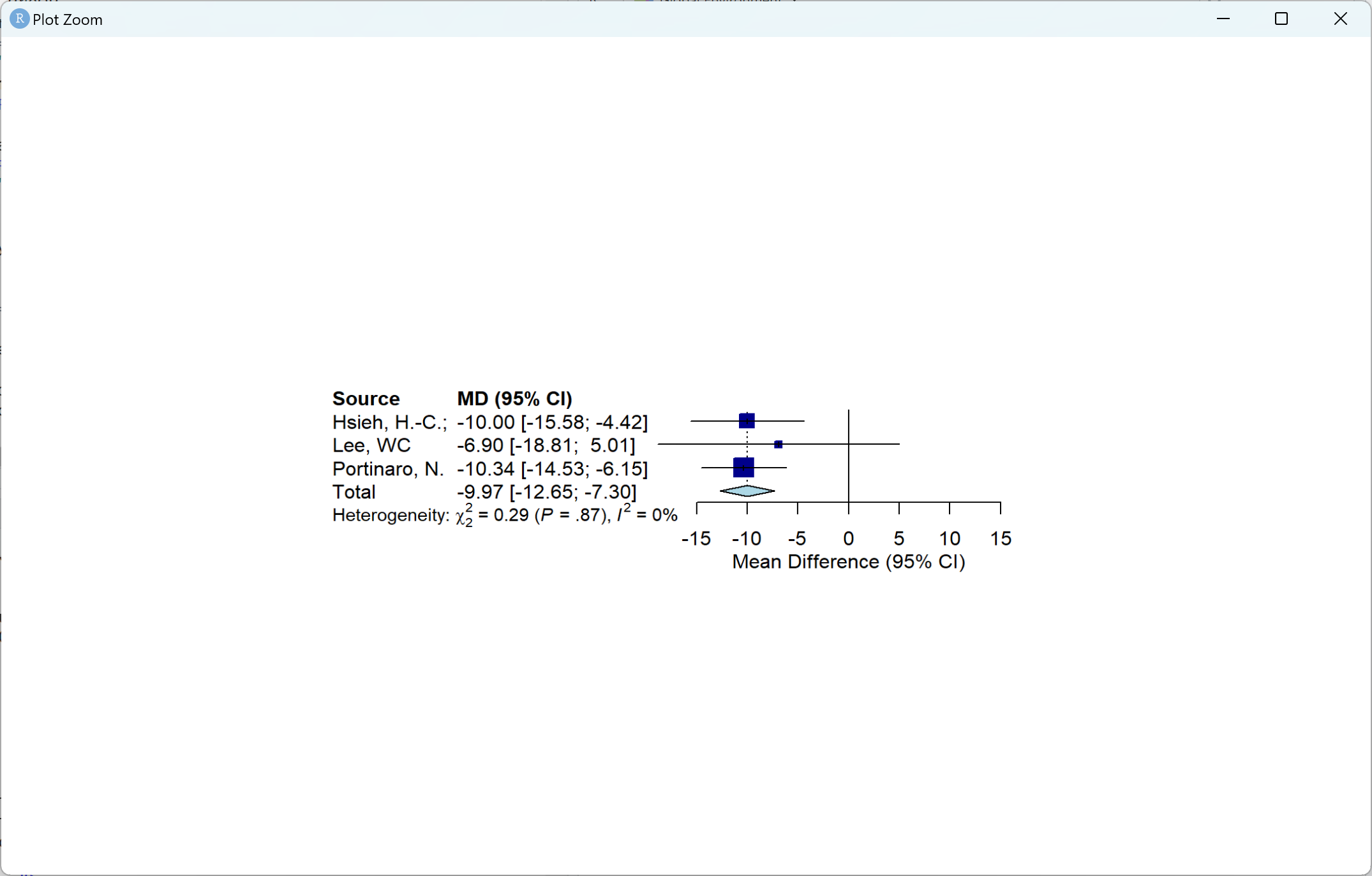


Supplementary Figure 21. Guided growth surgery, Migration Percentage


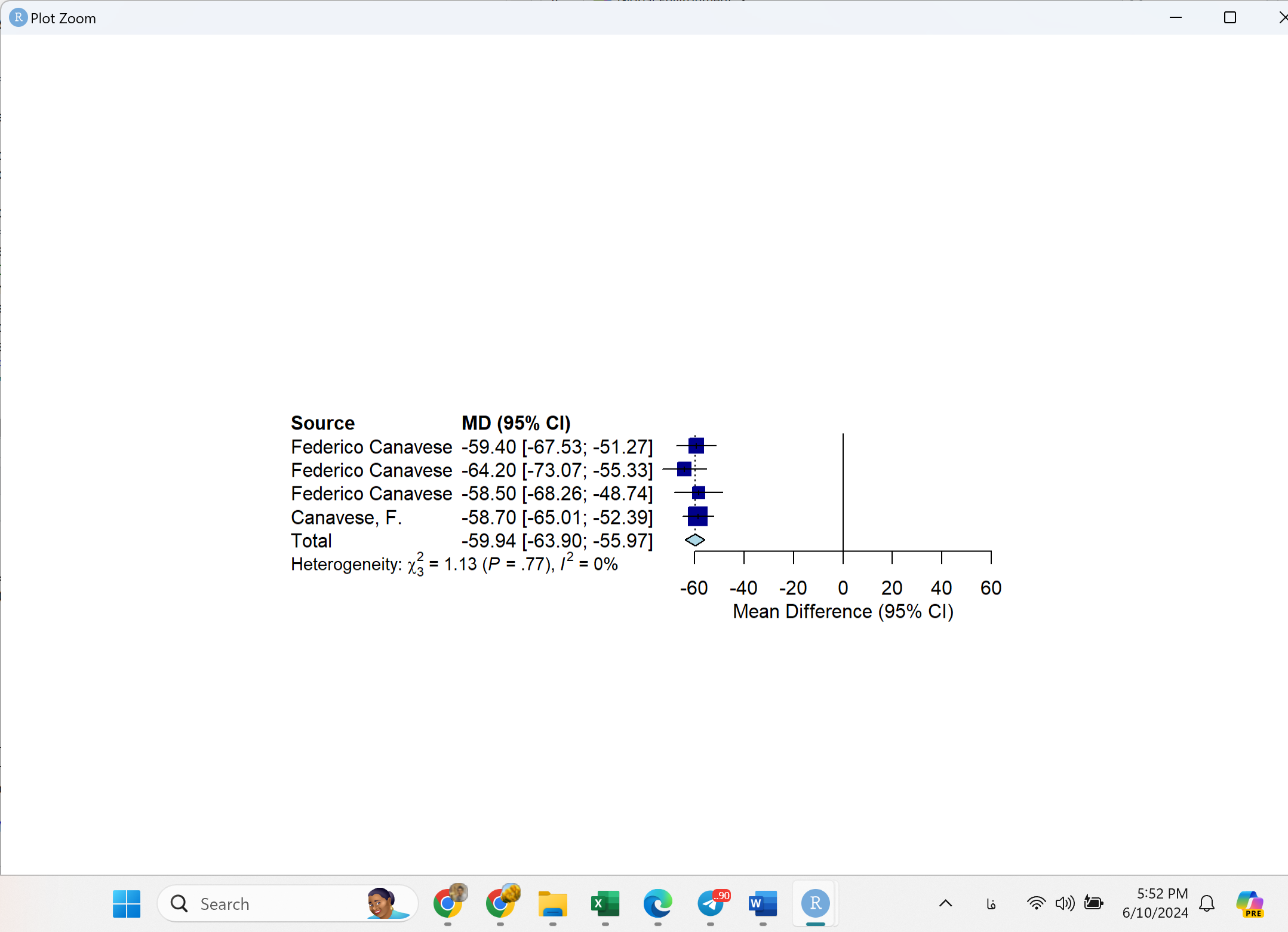


Supplementary Figure 22. Percutaneous Pelvic Osteotomy, Migration Percentage

**Appendix C (Baujot plots) :**


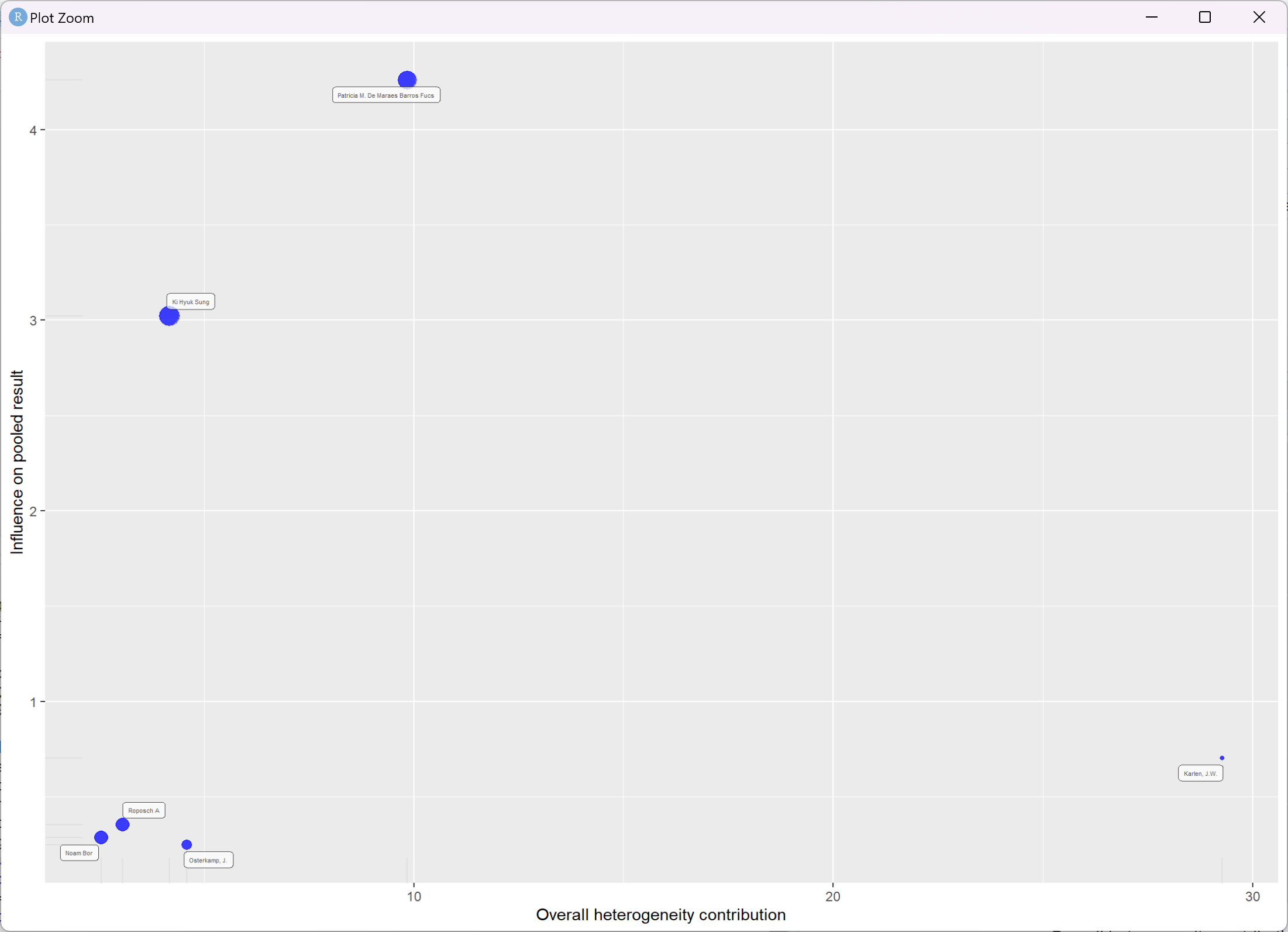


Supplementary Figure 23. Pelvic Osteotomy surgery, Acetabular index


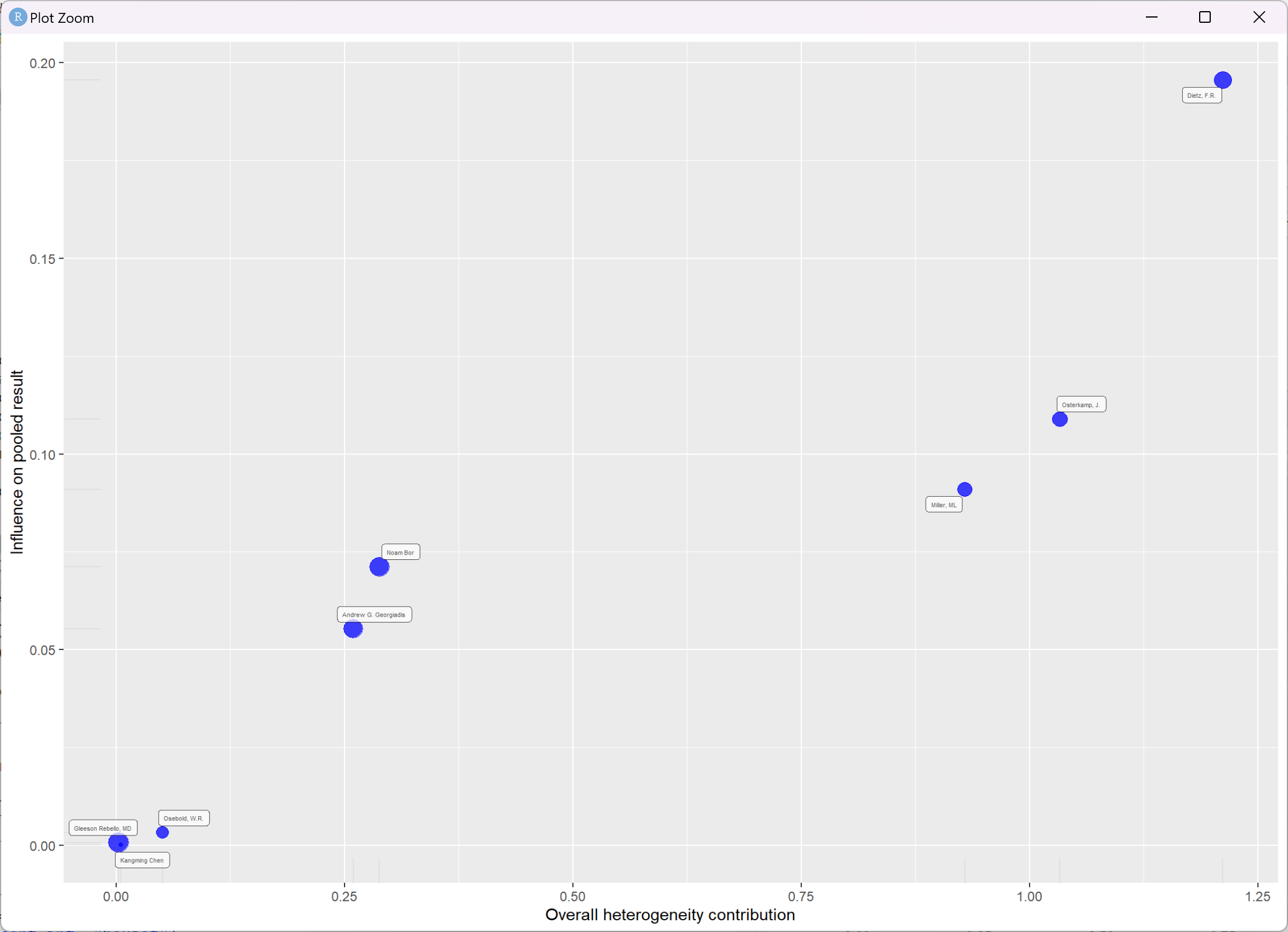


Supplementary Figure 24. Pelvic Osteotomy surgery, Center Edge Angle


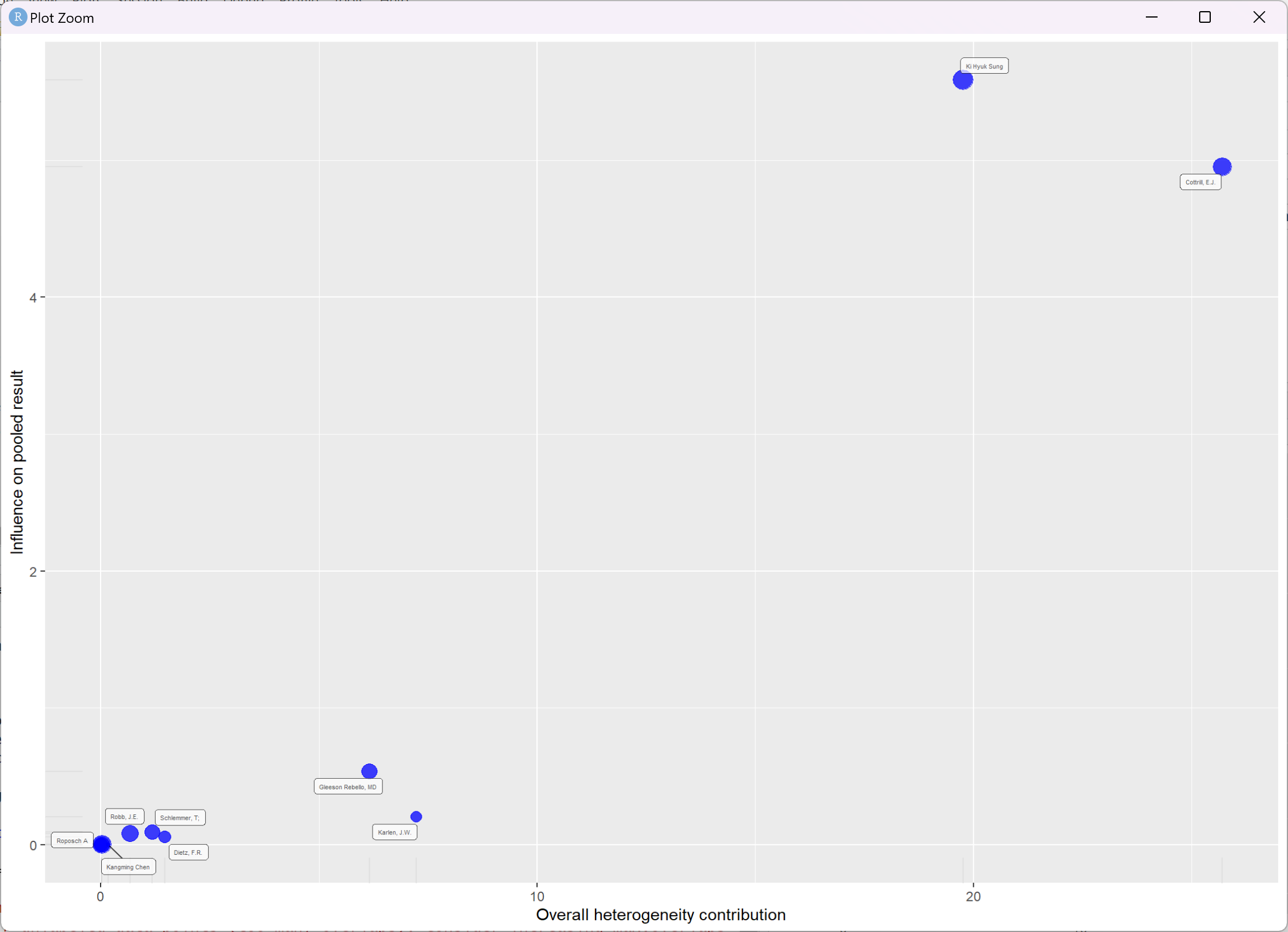


Supplementary Figure 25. Pelvic Osteotomy surgery, Migration Percentage


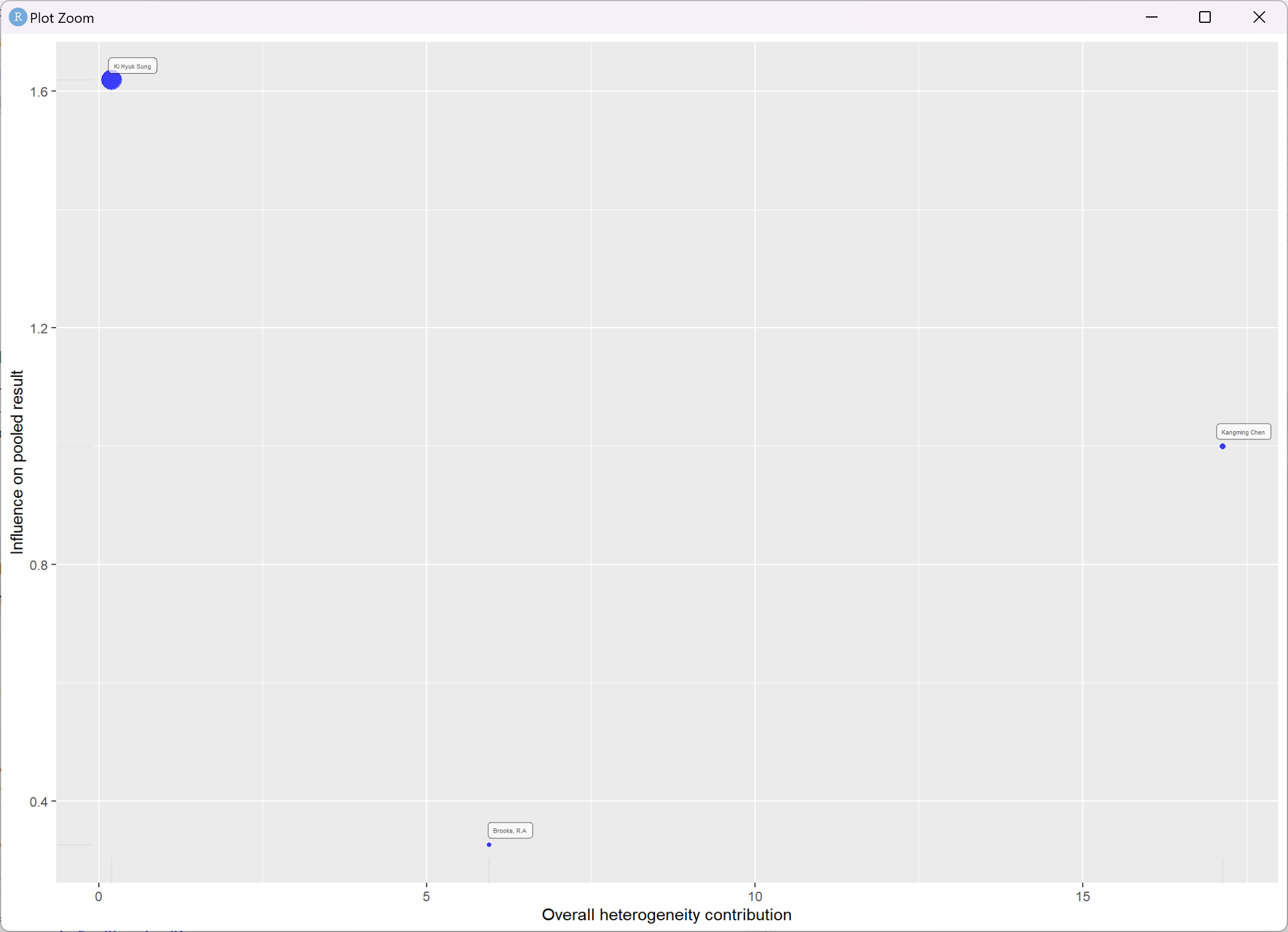


Supplementary Figure 26. Pelvic Osteotomy surgery, Neck Shaft Angle


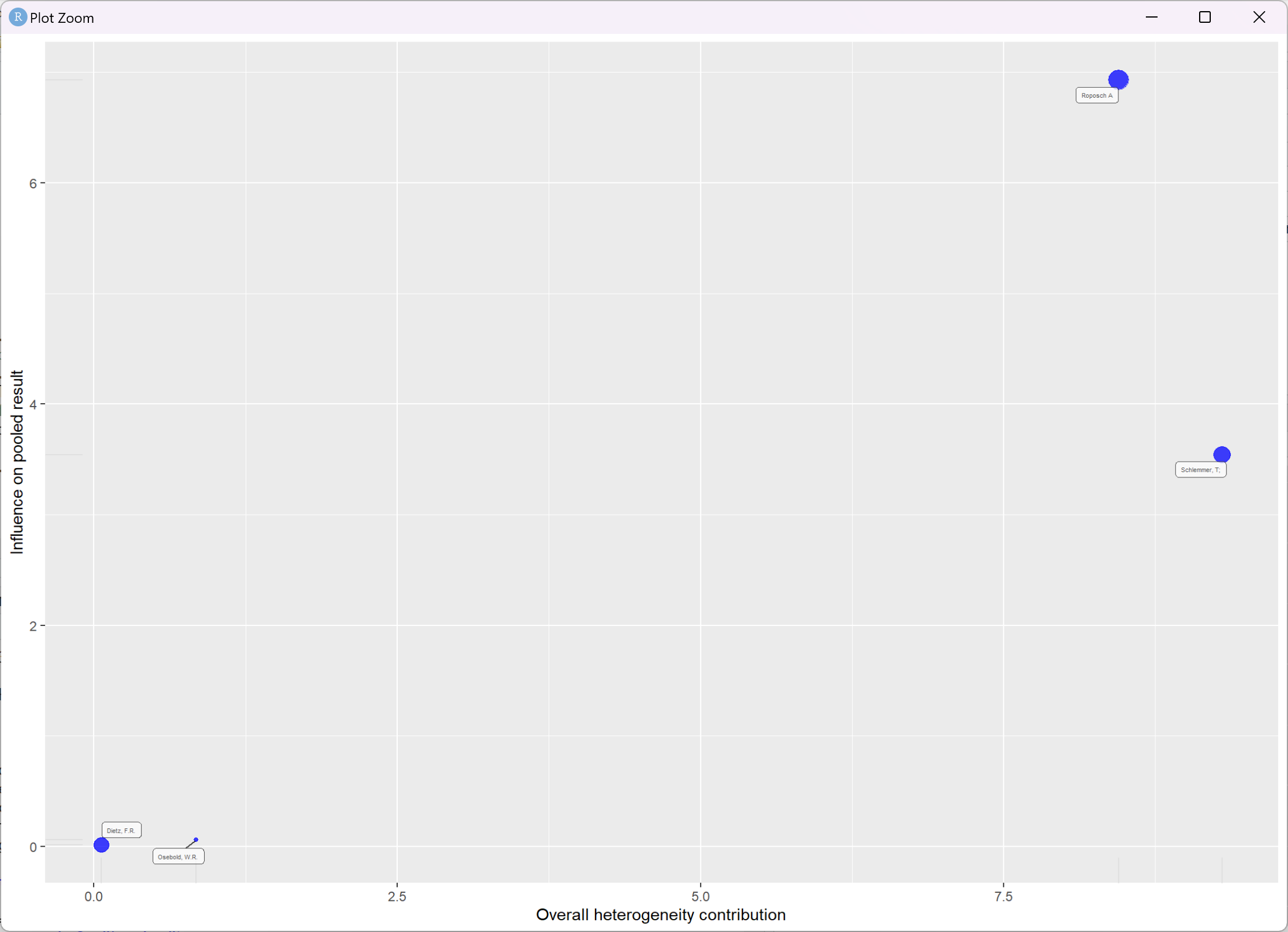


Supplementary Figure 27. Pelvic Osteotomy surgery, Sharp Angle


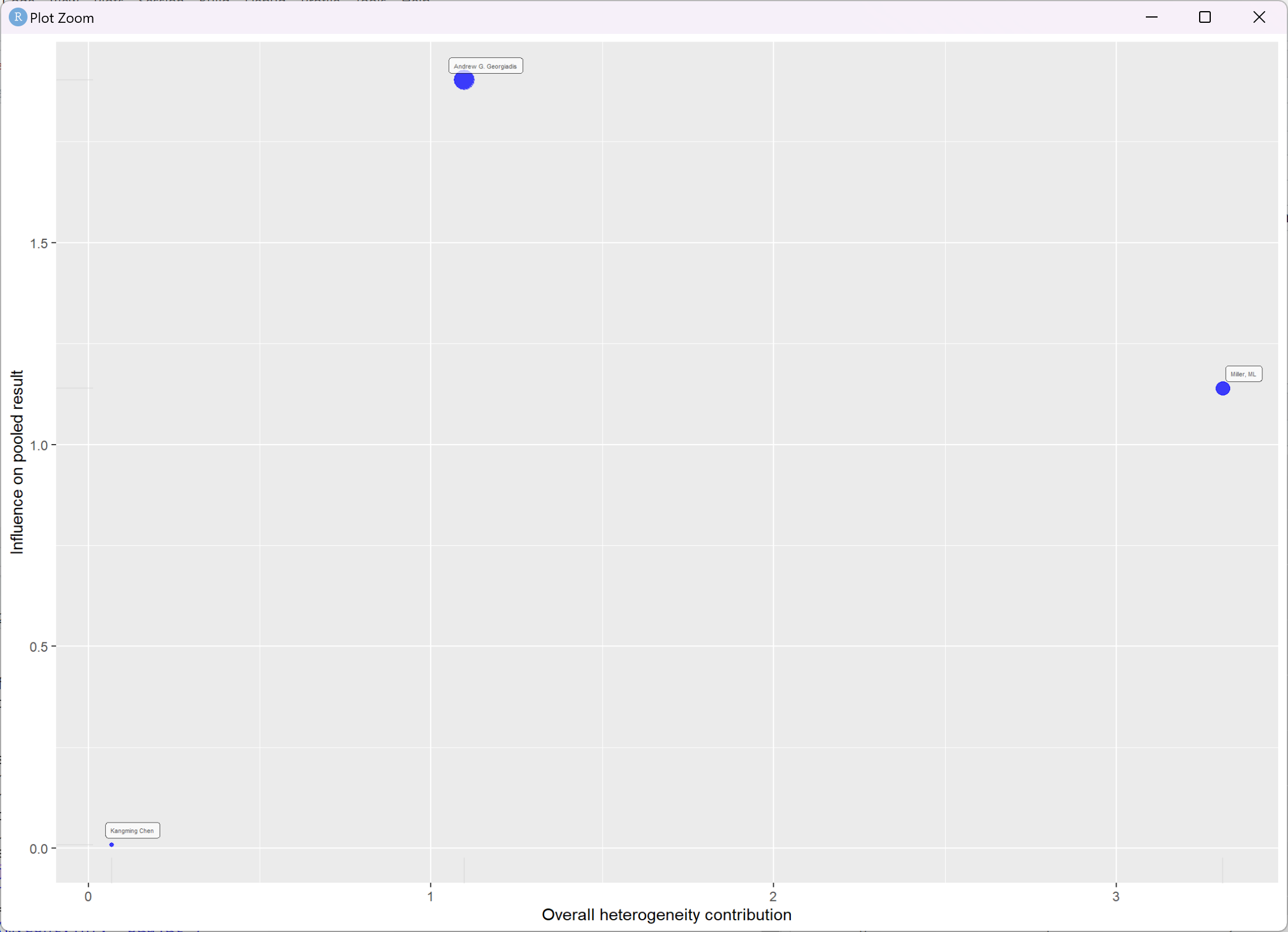


Supplementary Figure 28. Pelvic Osteotomy surgery, Tonnis Angle


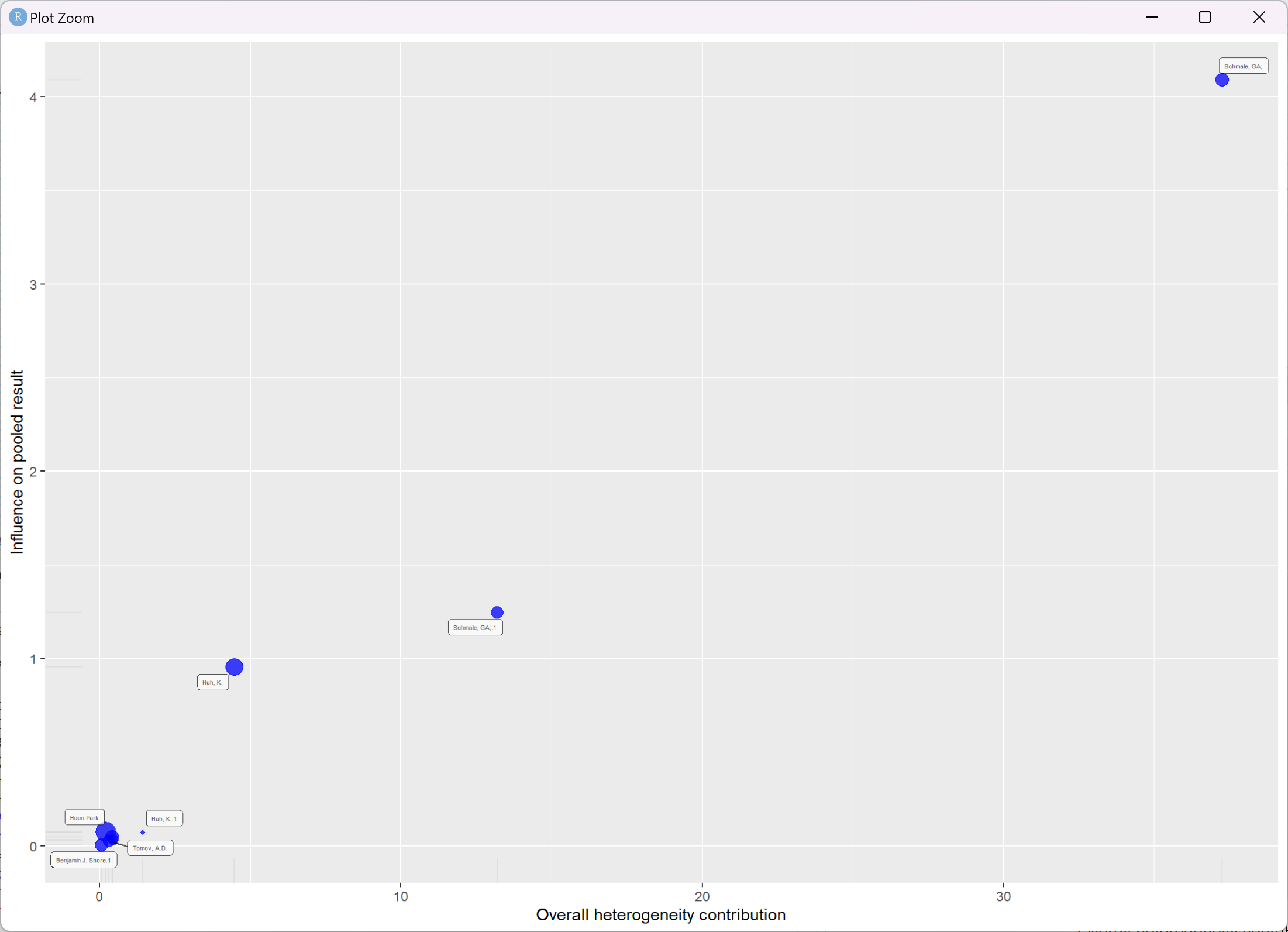


Supplementary Figure 29. Femur Osteotomy surgery, Acetabular index


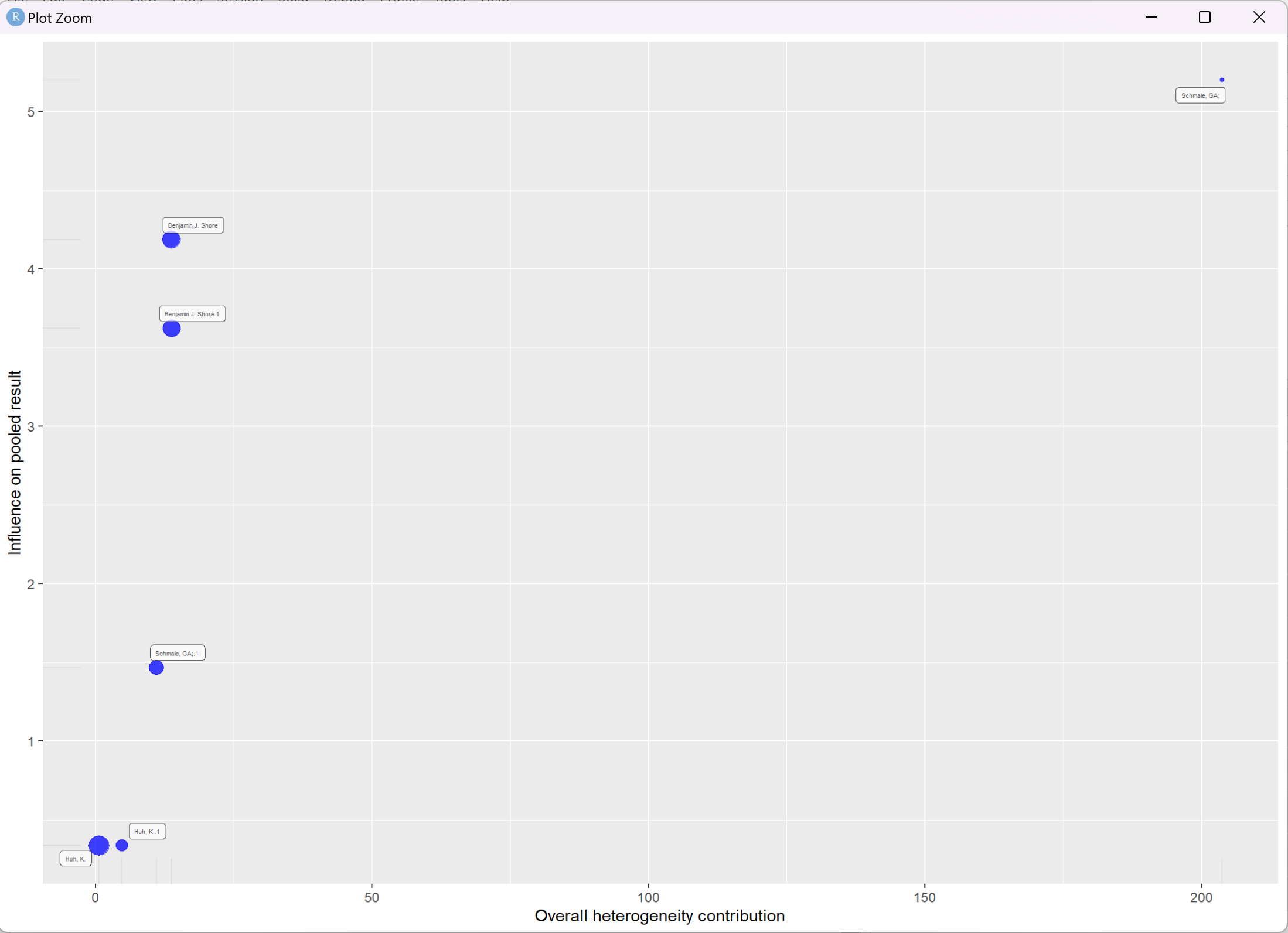


Supplementary Figure 30. Femur Osteotomy surgery, Center Edge Angle


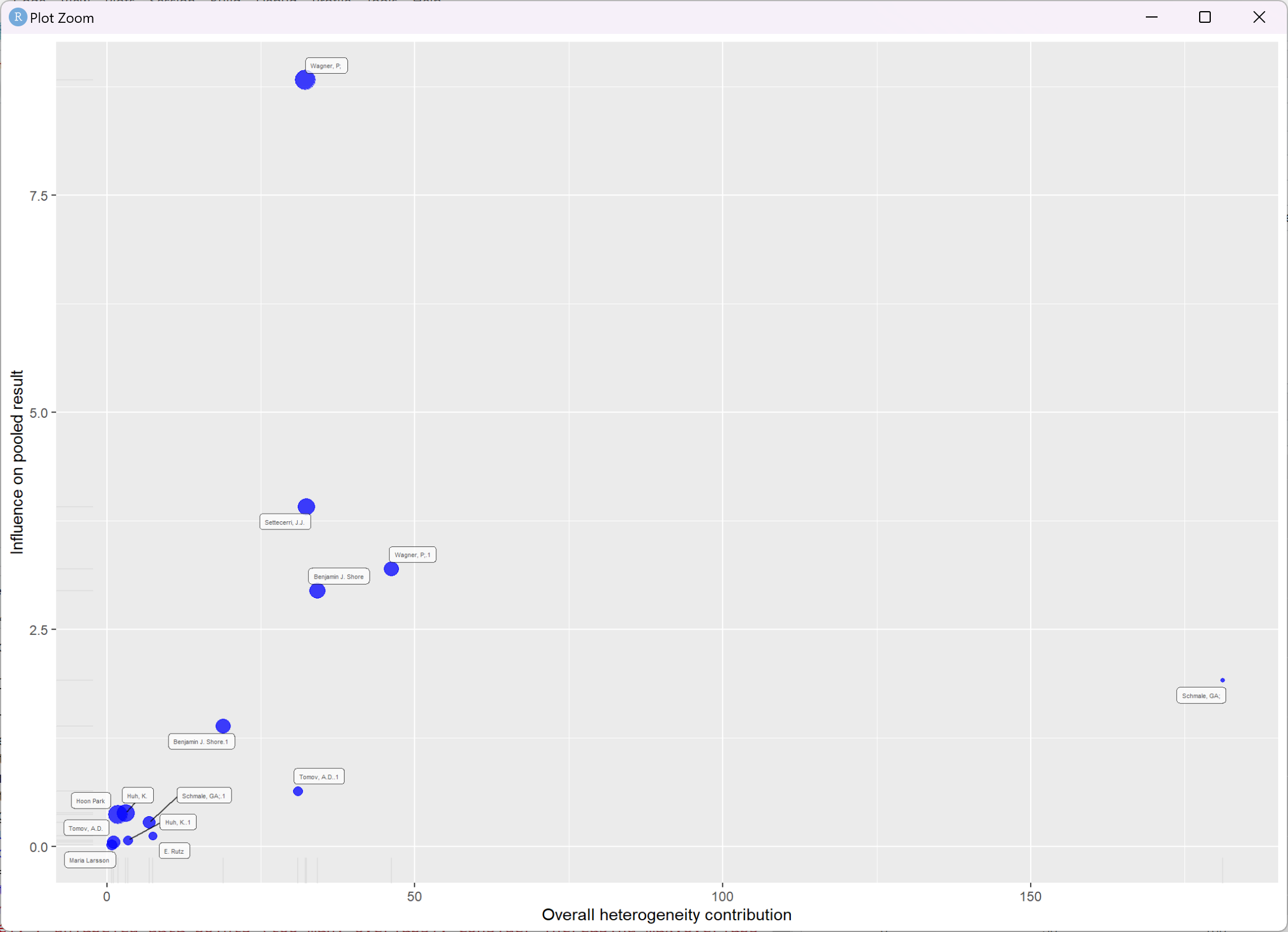


Supplementary Figure 31. Femur Osteotomy surgery, Migration Percentage


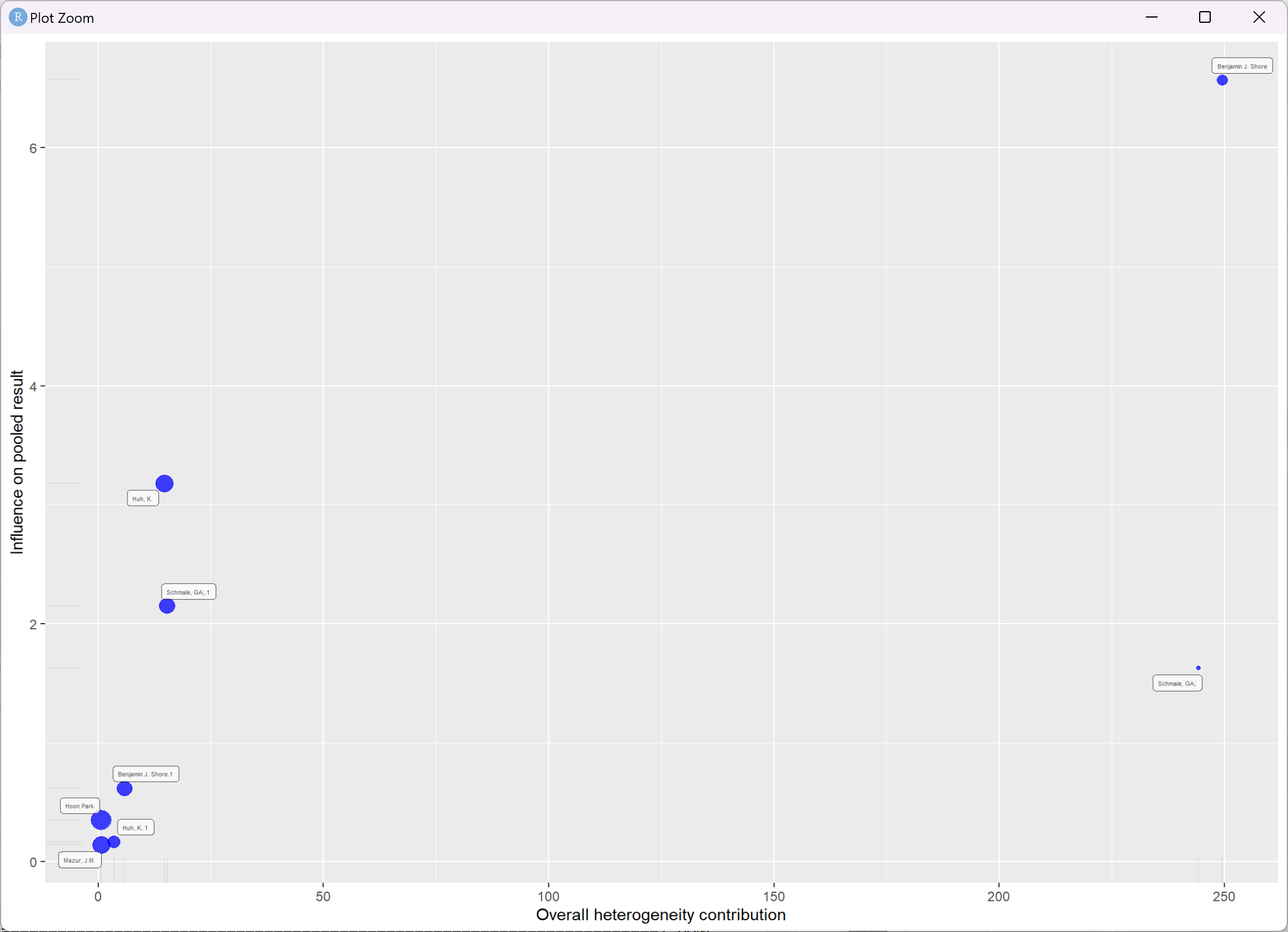


Supplementary Figure 32. Femur Osteotomy surgery, Neck Shaft Angle


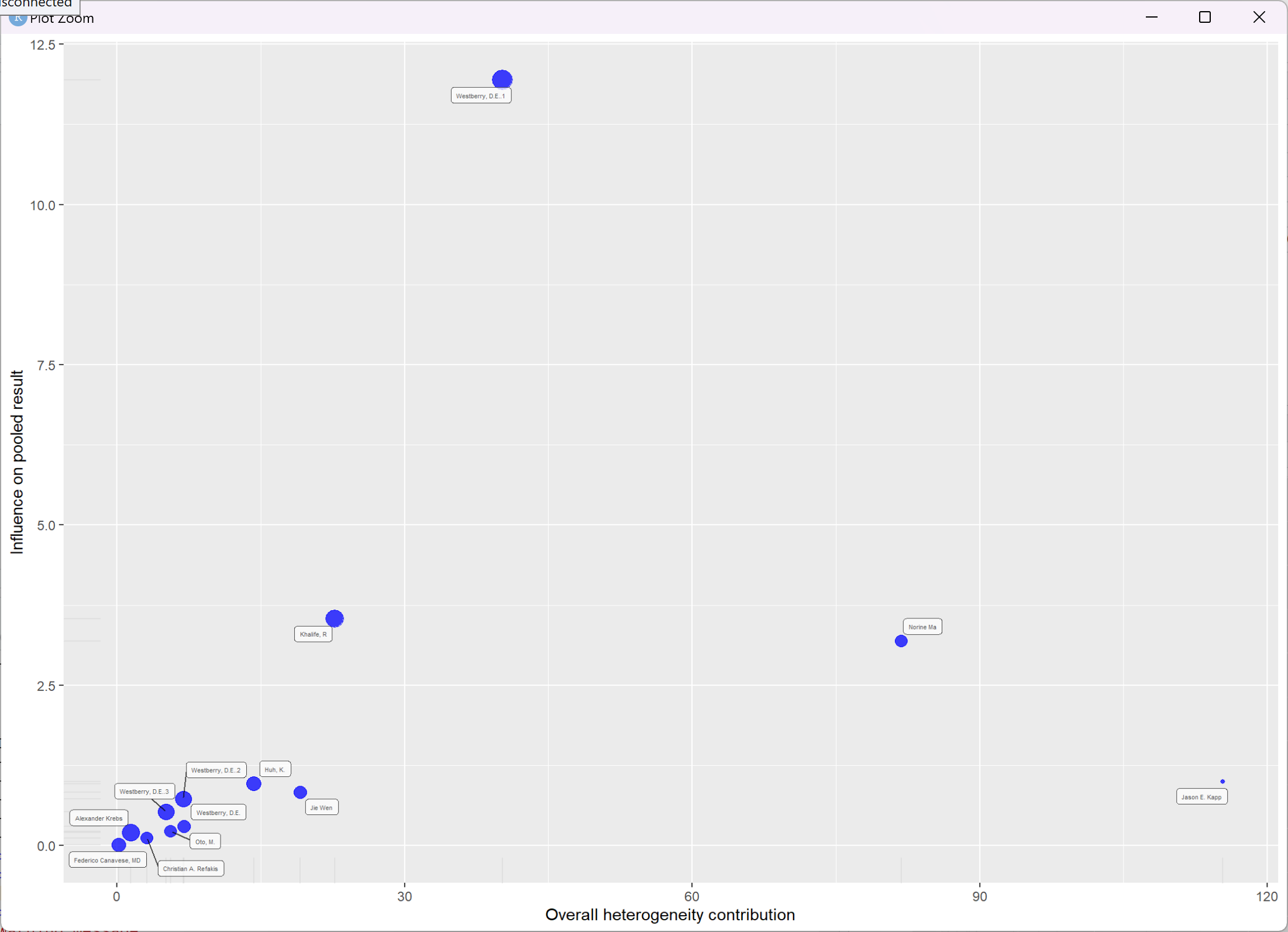


Supplementary Figure 33. Combination of Pelvic and Femur Osteotomy surgery, Acetabular Index


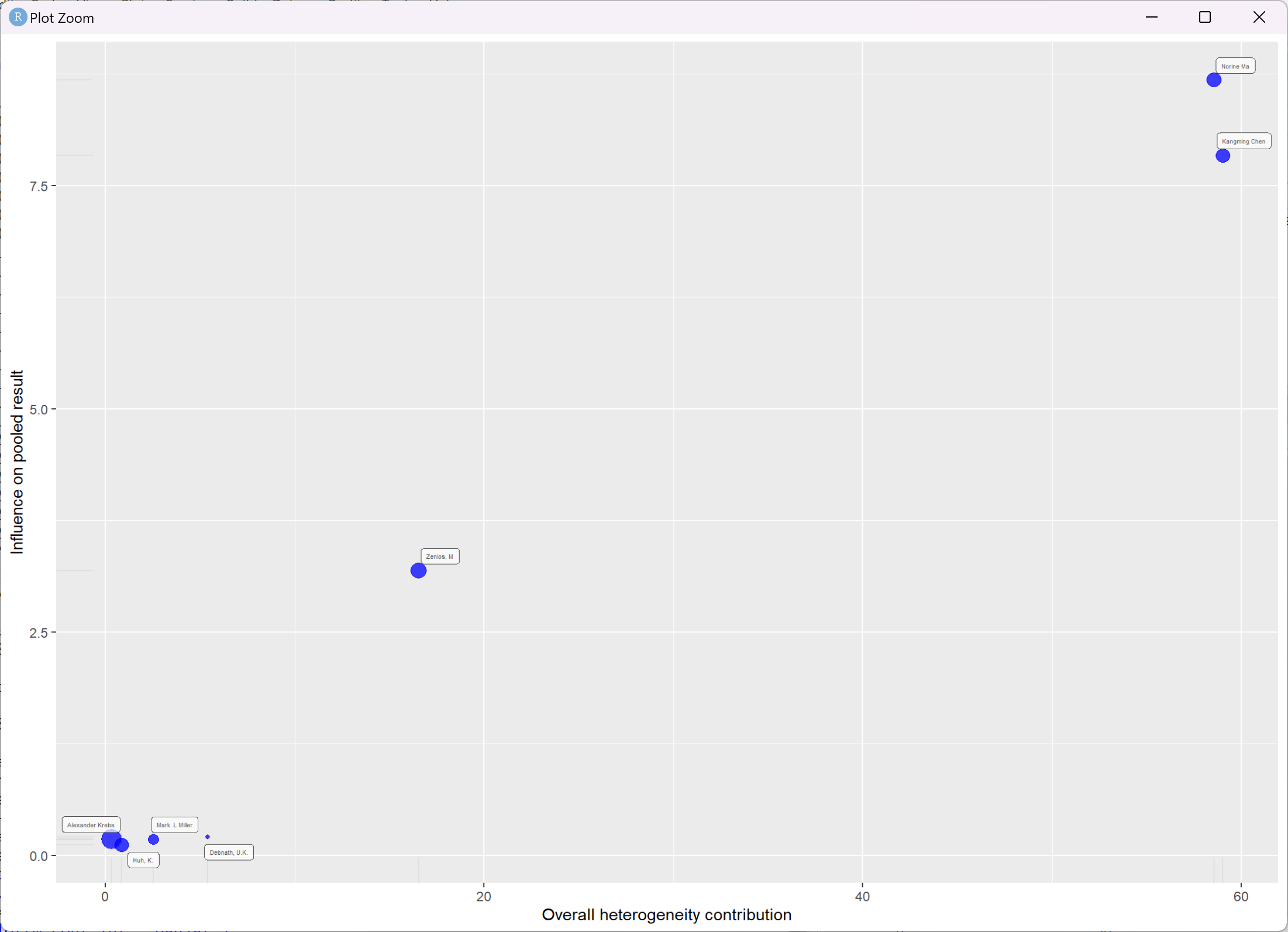


Supplementary Figure 34. Combination of Pelvic and Femur Osteotomy surgery, Center Edge Angle


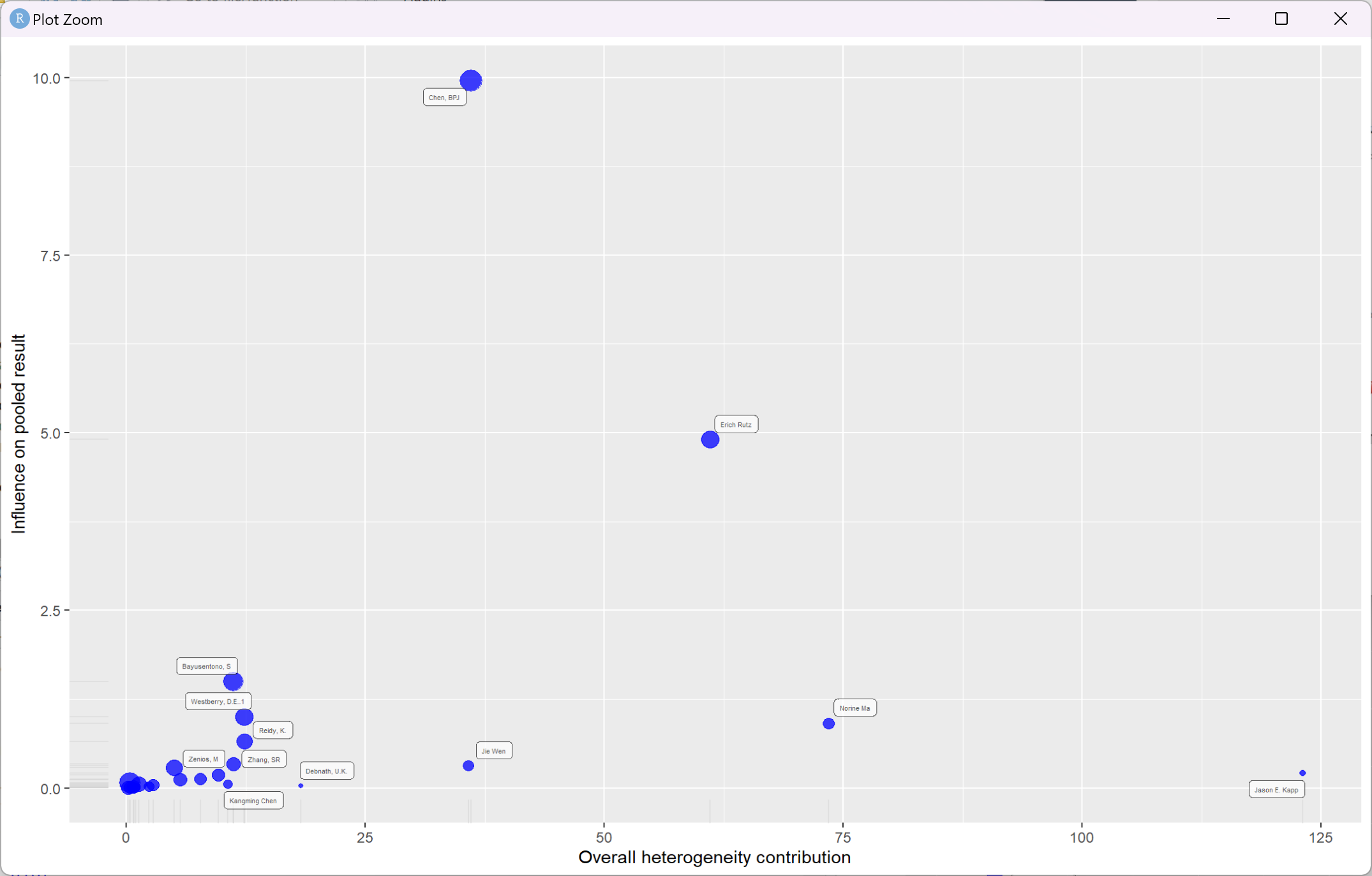


Supplementary Figure 35. Combination of Pelvic and Femur Osteotomy surgery, Migration Percentage


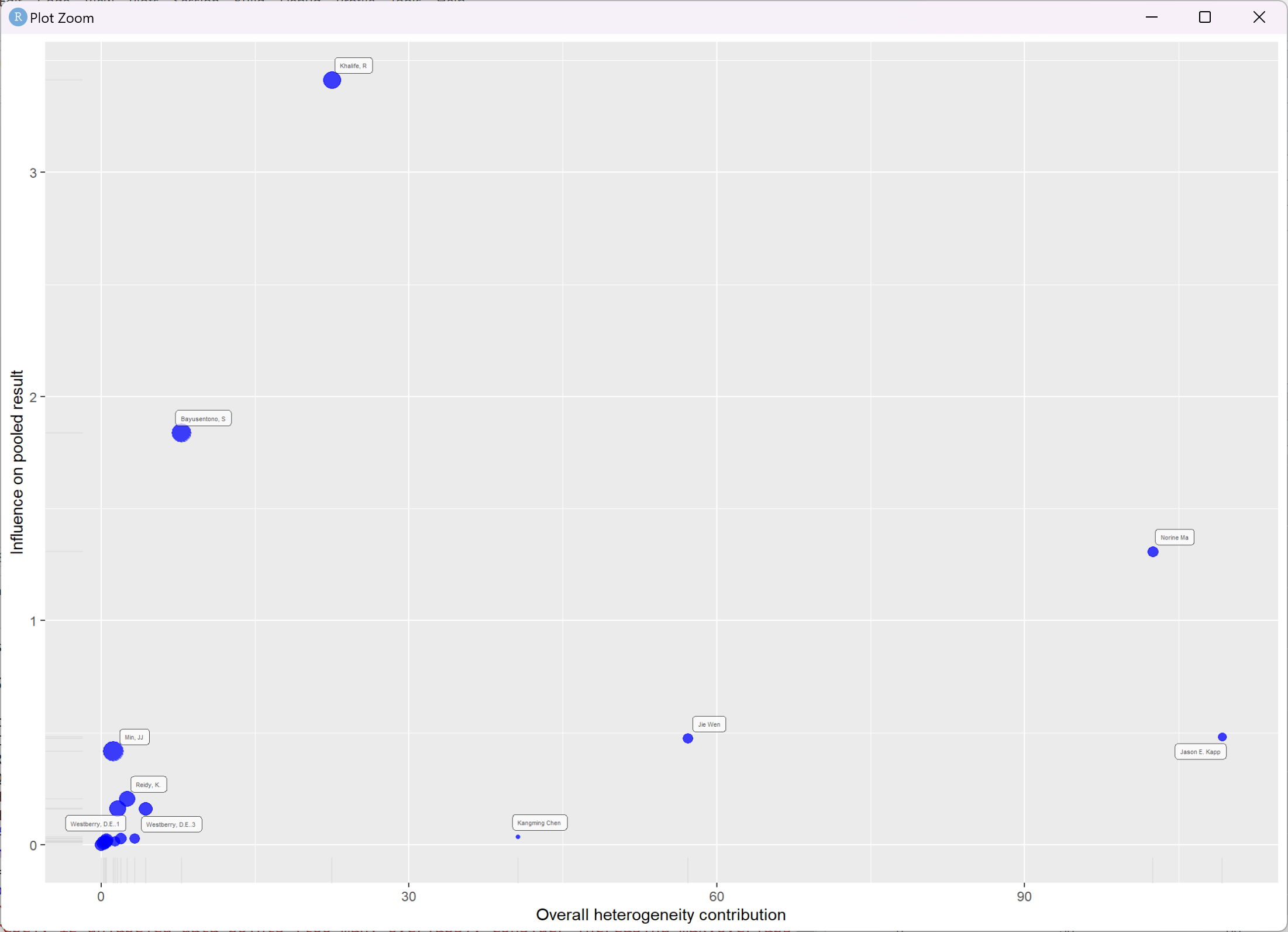


Supplementary Figure 36. Combination of Pelvic and Femur Osteotomy surgery, Neck Shaft Angle


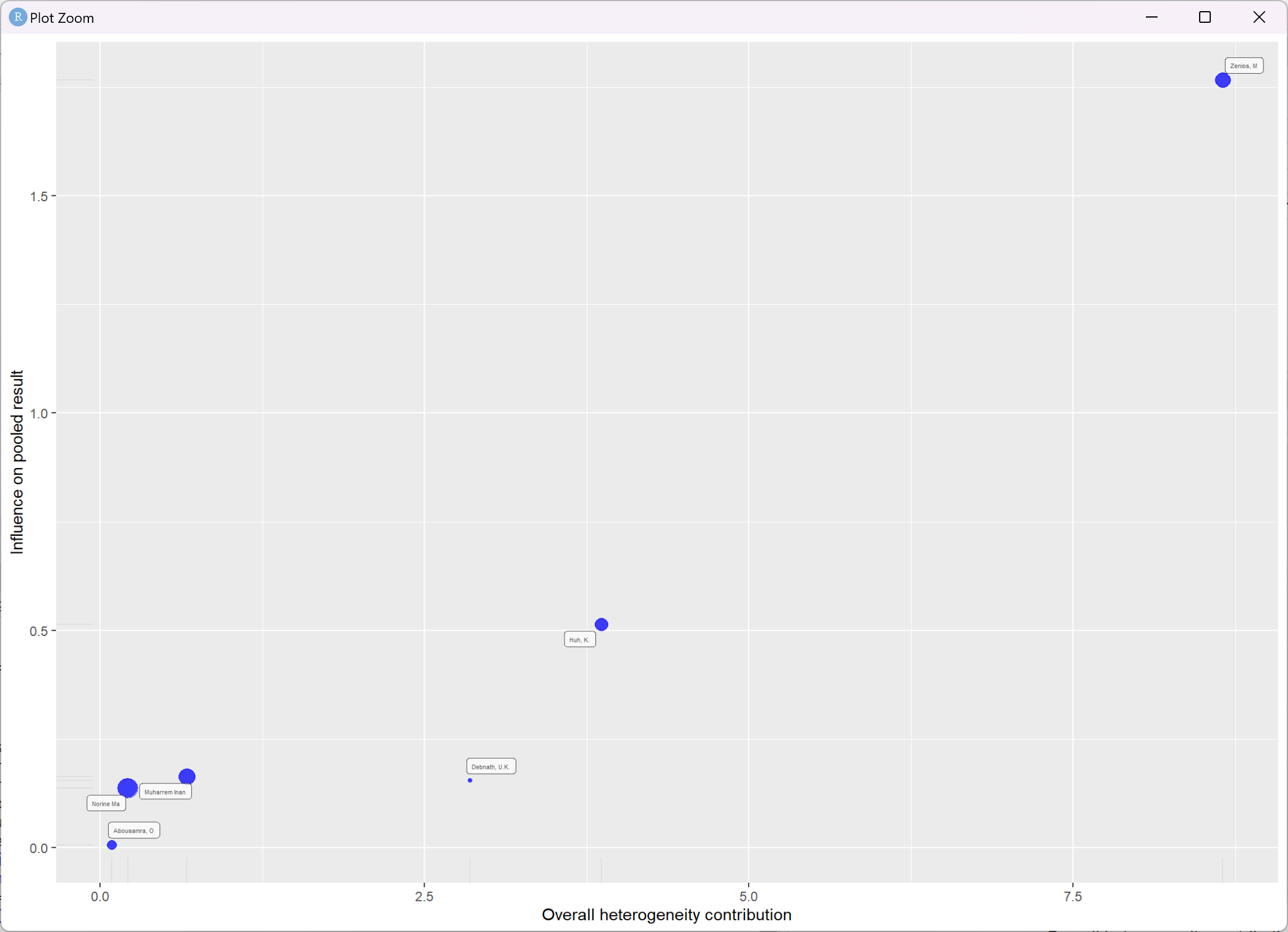


Supplementary Figure 37. Combination of Pelvic and Femur Osteotomy surgery, Sharp Angle


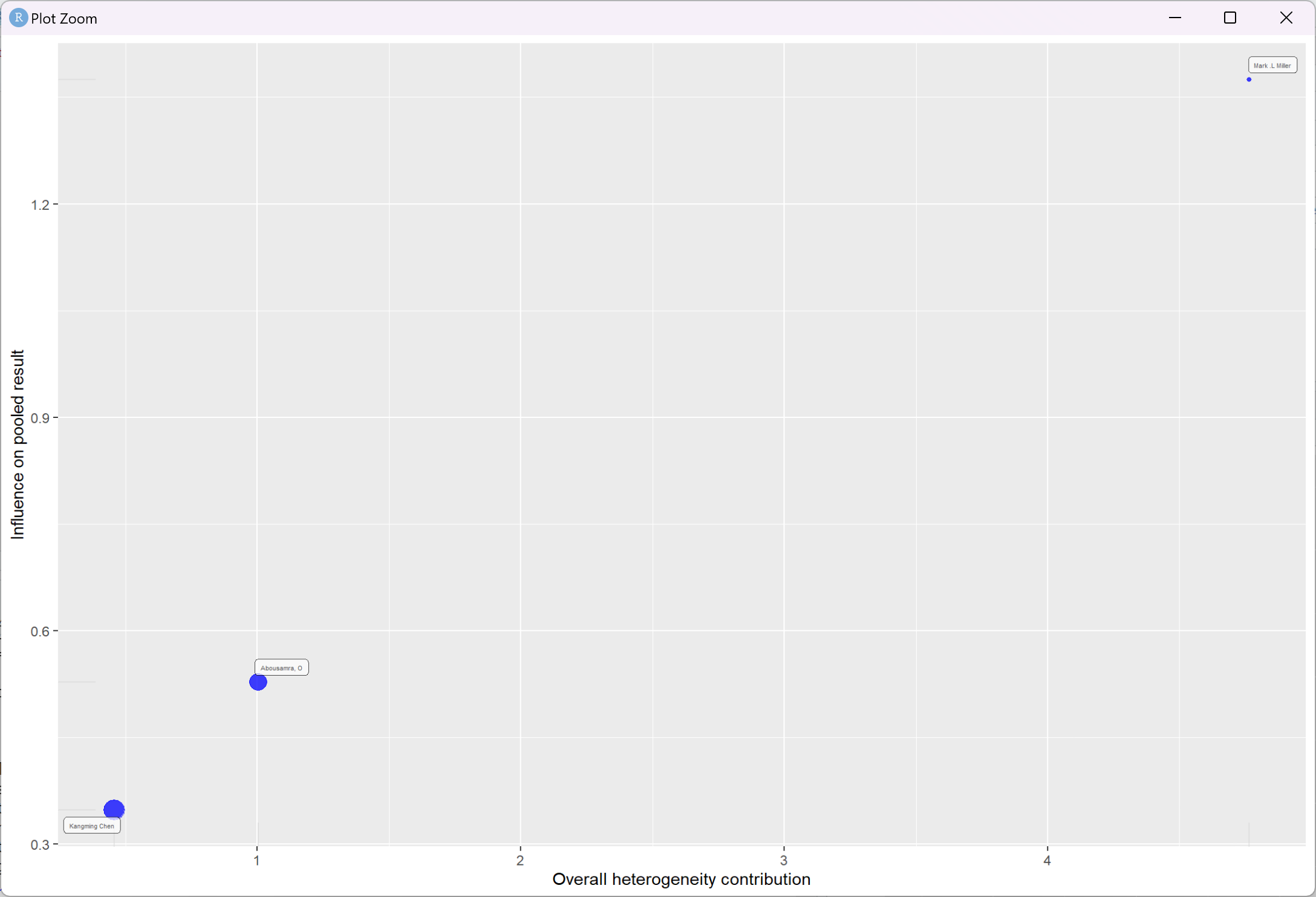


Supplementary Figure 38. Combination of Pelvic and Femur Osteotomy surgery, Tonnis Angle


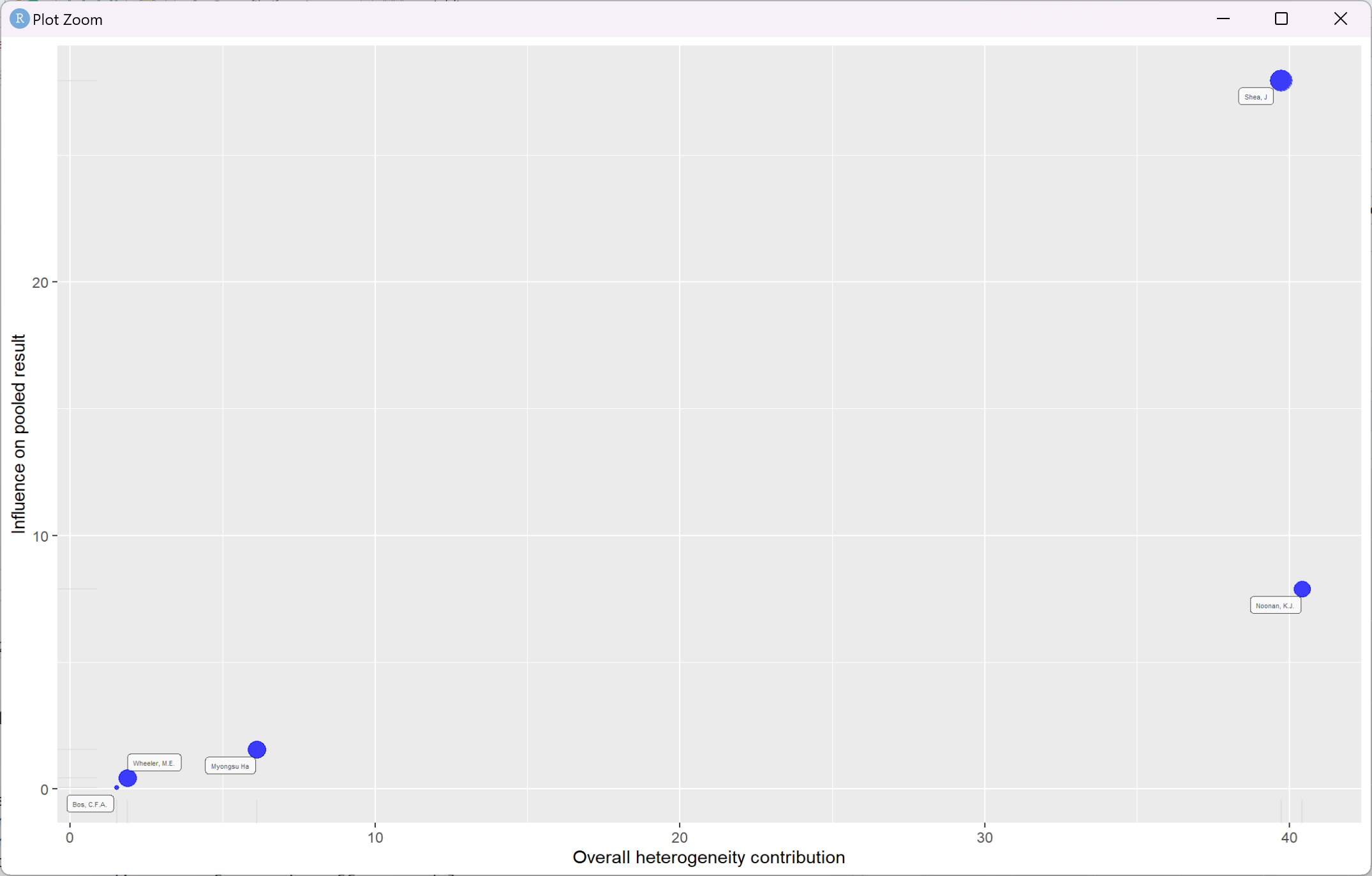


Supplementary Figure 39. Soft Tissue surgery, Acetabular Index


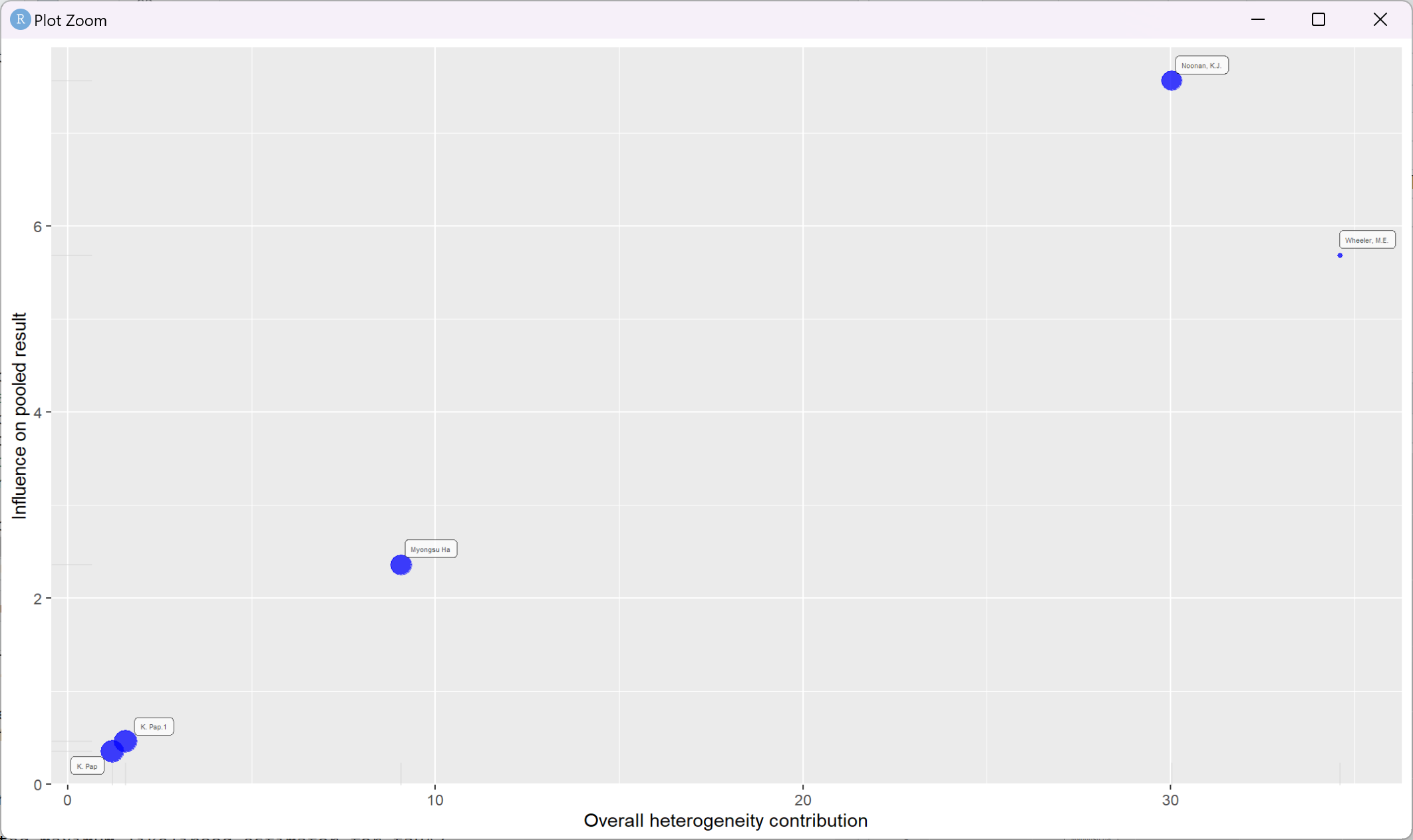


Supplementary Figure 40. Soft Tissue surgery, Center Edge Angle


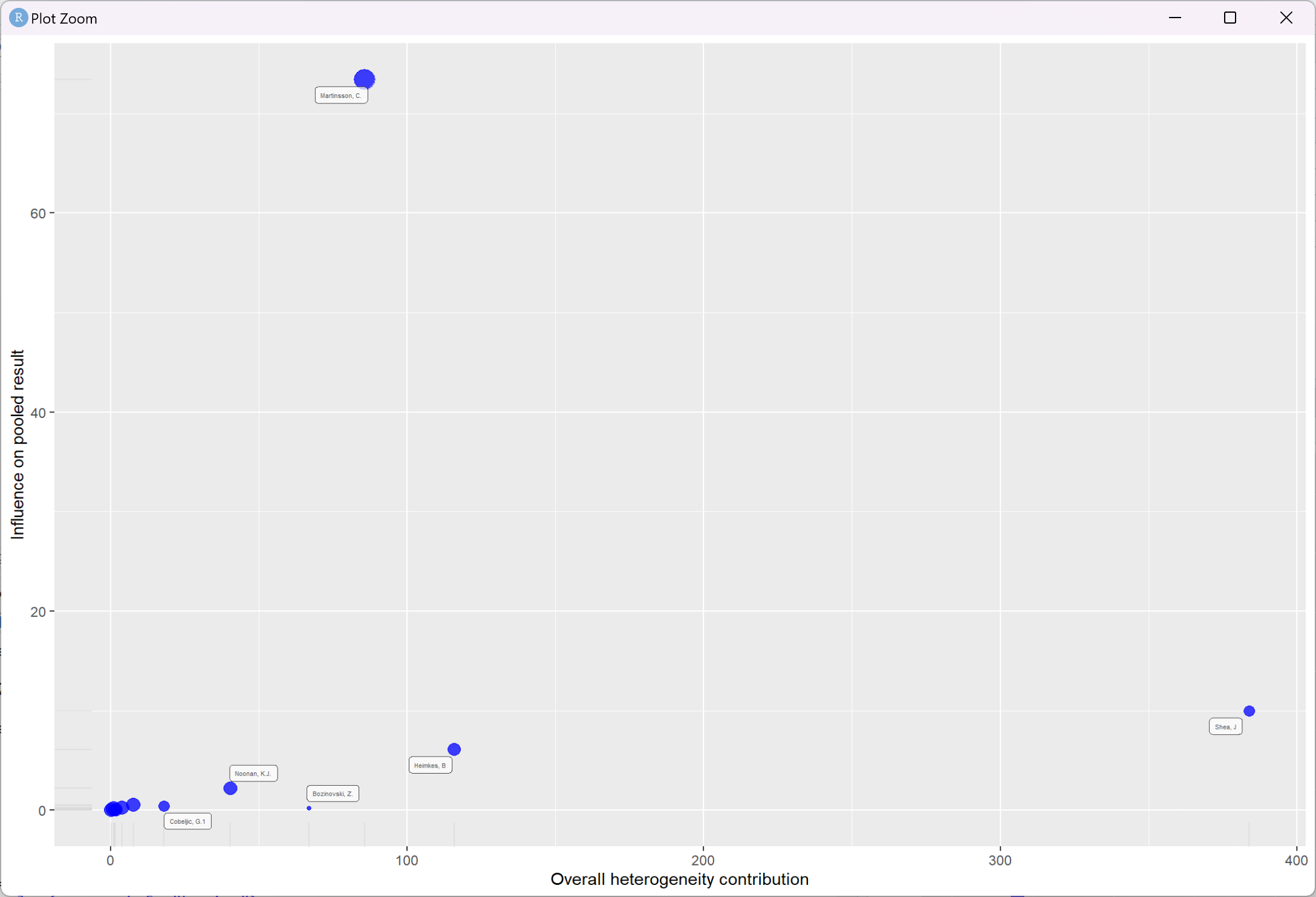


Supplementary Figure 41. Soft Tissue surgery, Migration Percentage


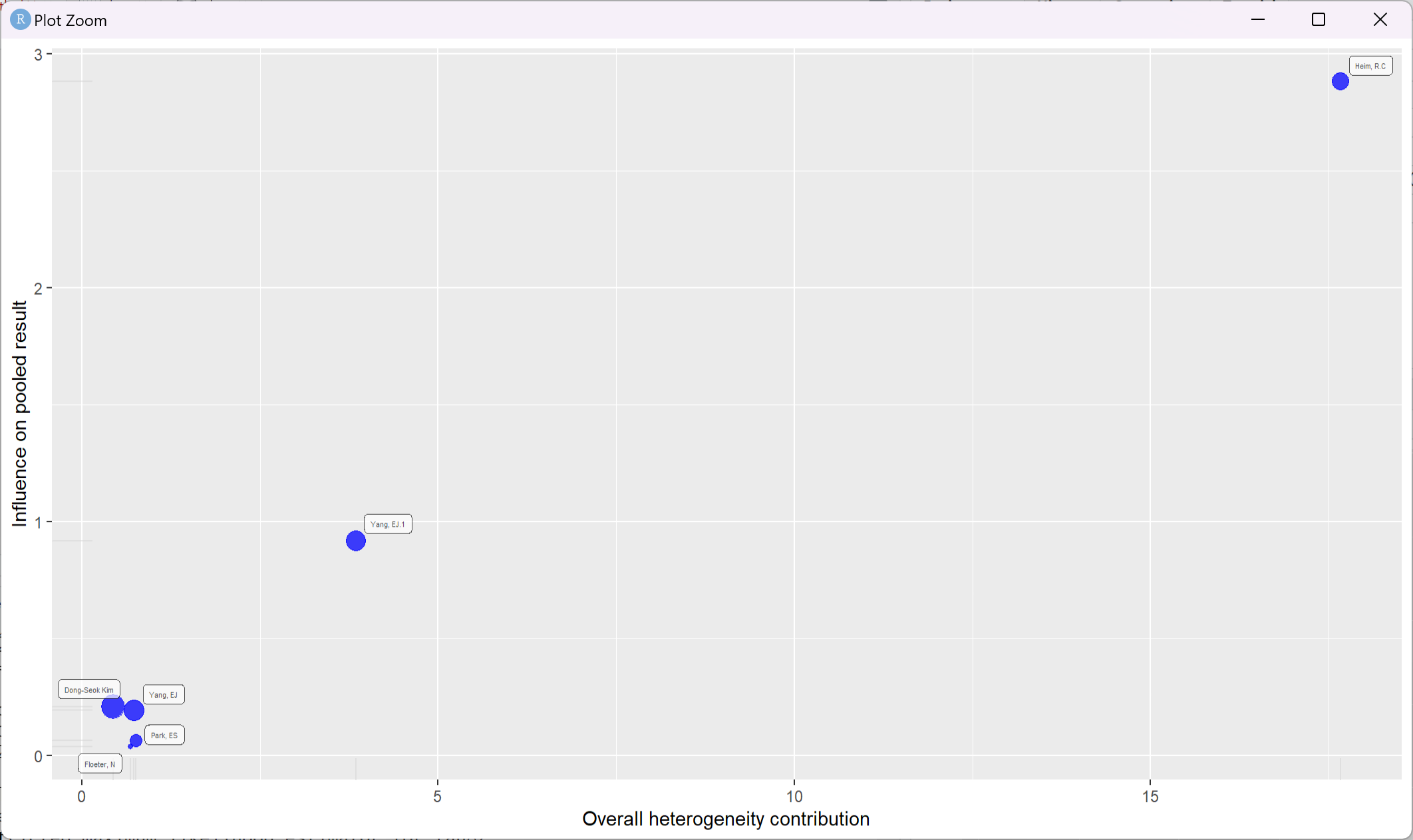


Supplementary Figure 42. Tone decrease, Migration Percentage


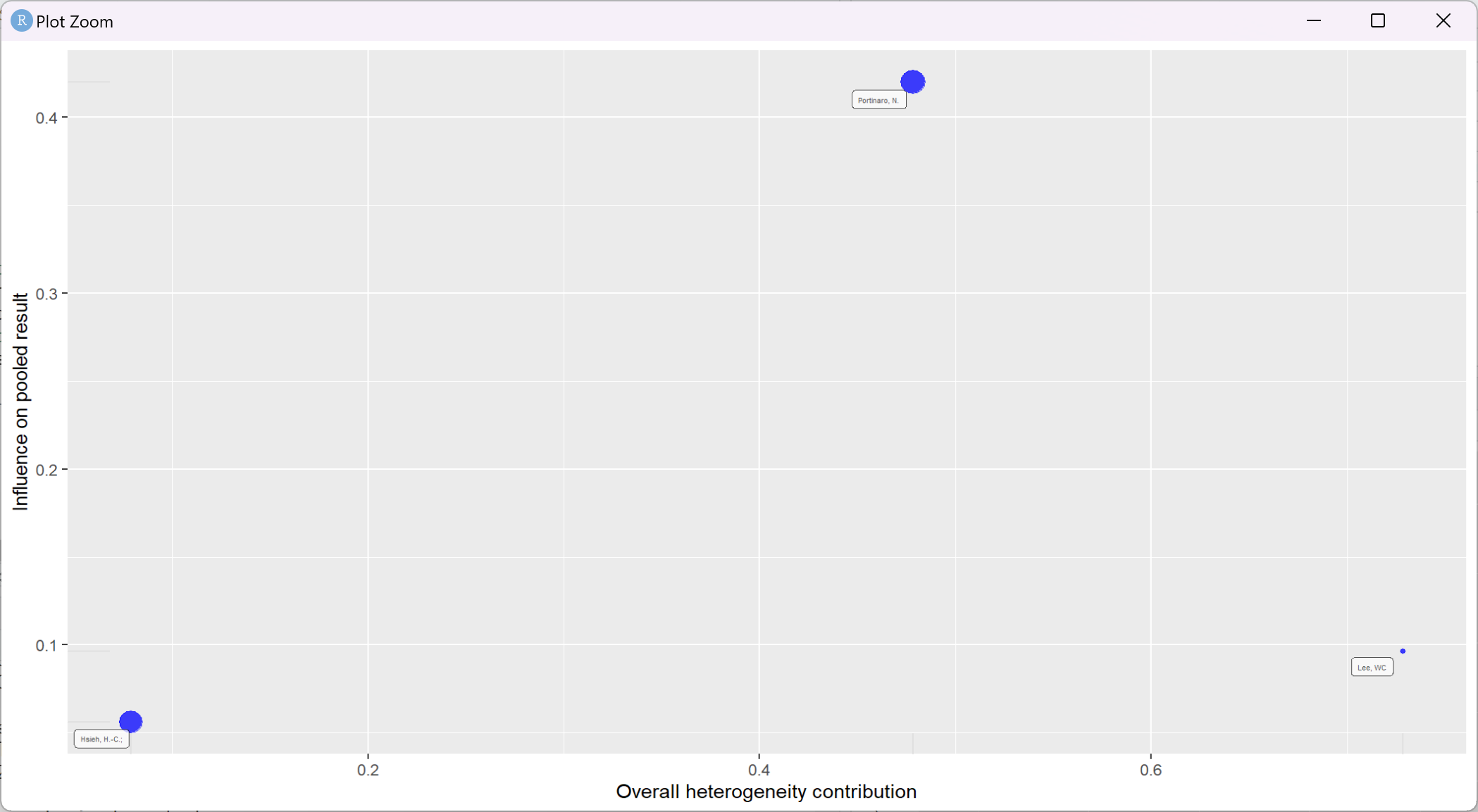


Supplementary Figure 43. Guided growth surgery, Migration Percentage


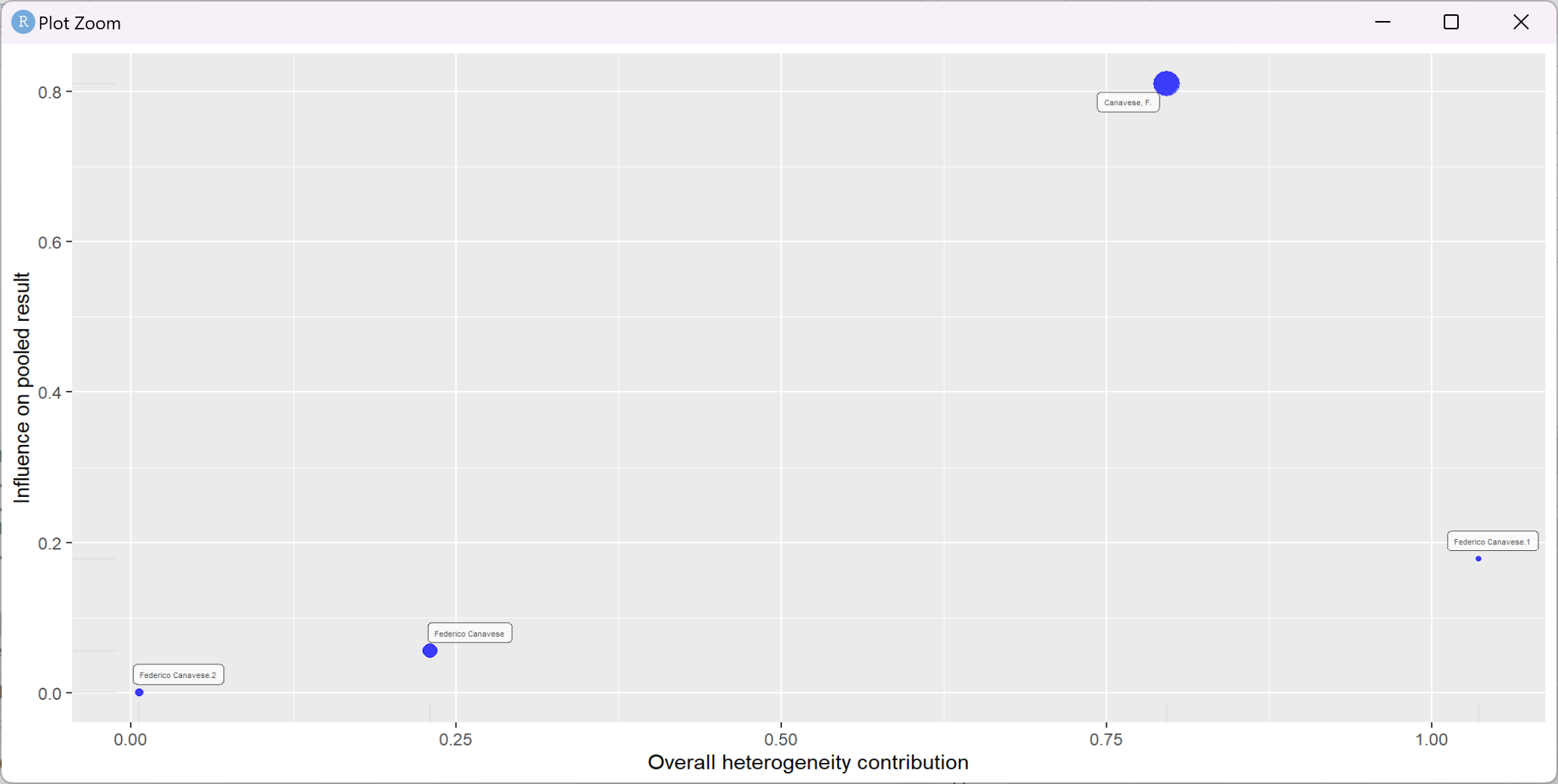


Supplementary Figure 44. Percutaneous Pelvic Osteotomy, Migration Percentage

**Appendix D (Funnel Plots):**


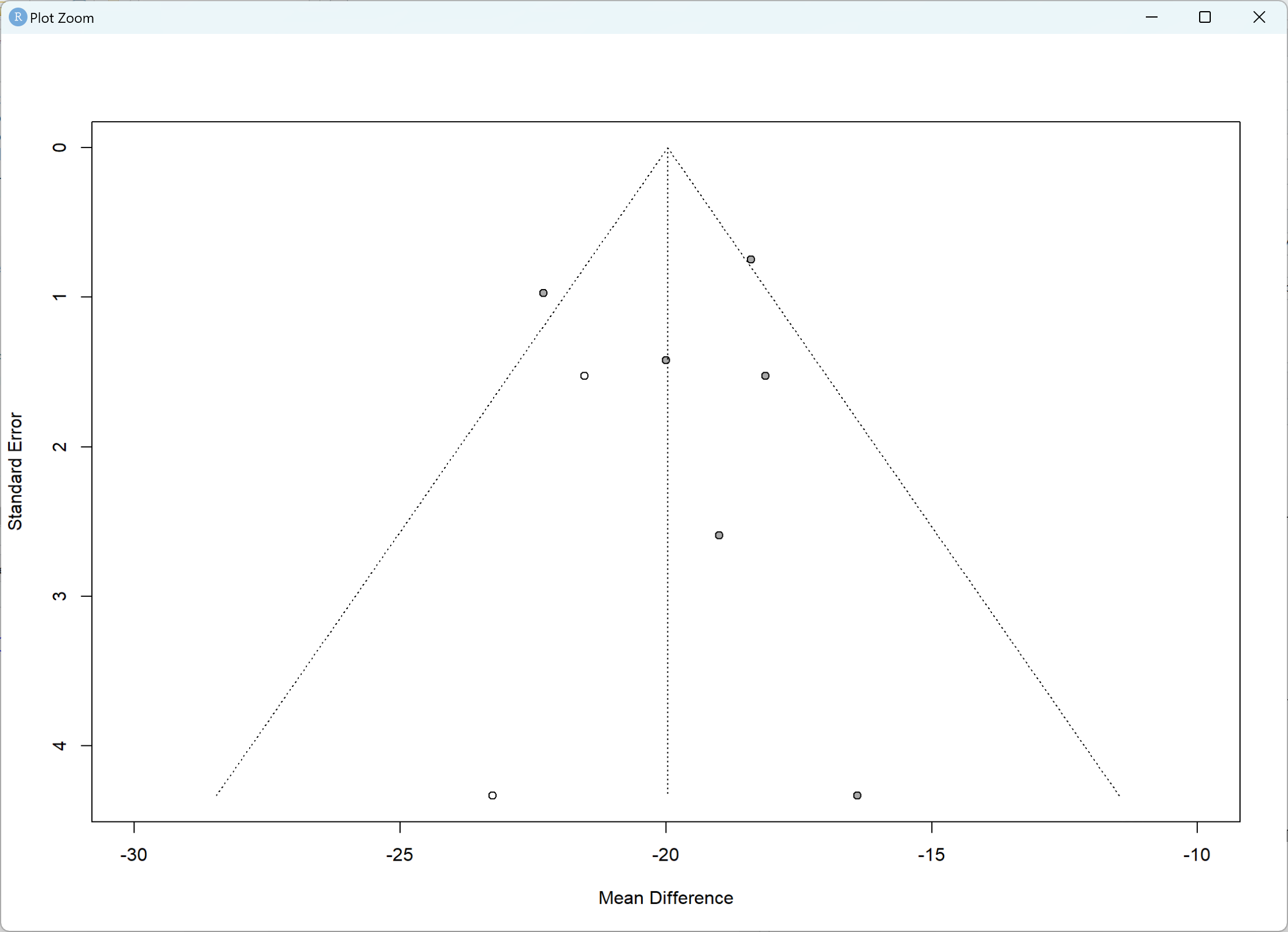


Supplementary Figure 45. Pelvic Osteotomy surgery, Acetabular index


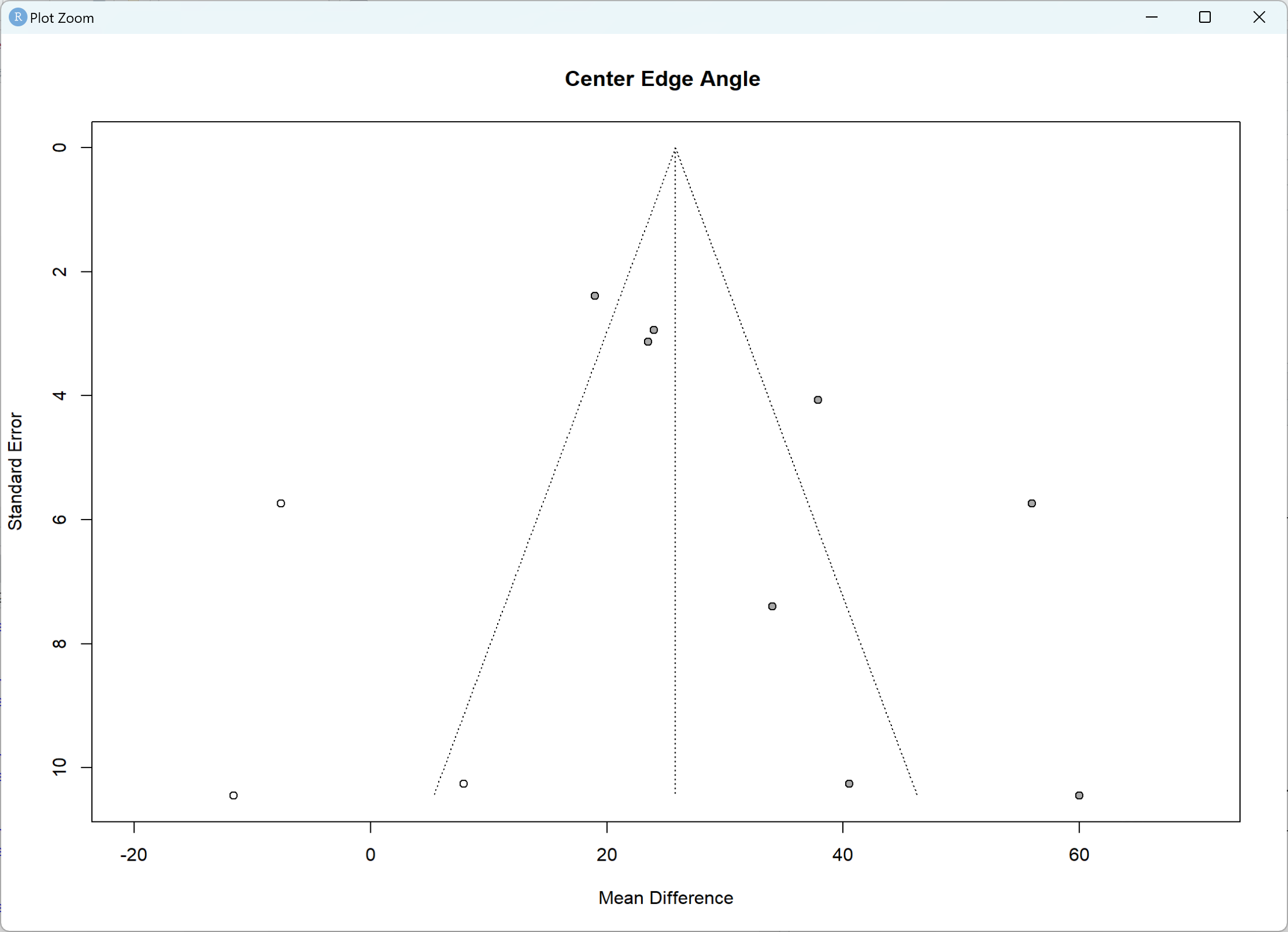


Supplementary Figure 46. Pelvic Osteotomy surgery, Center Edge Angle


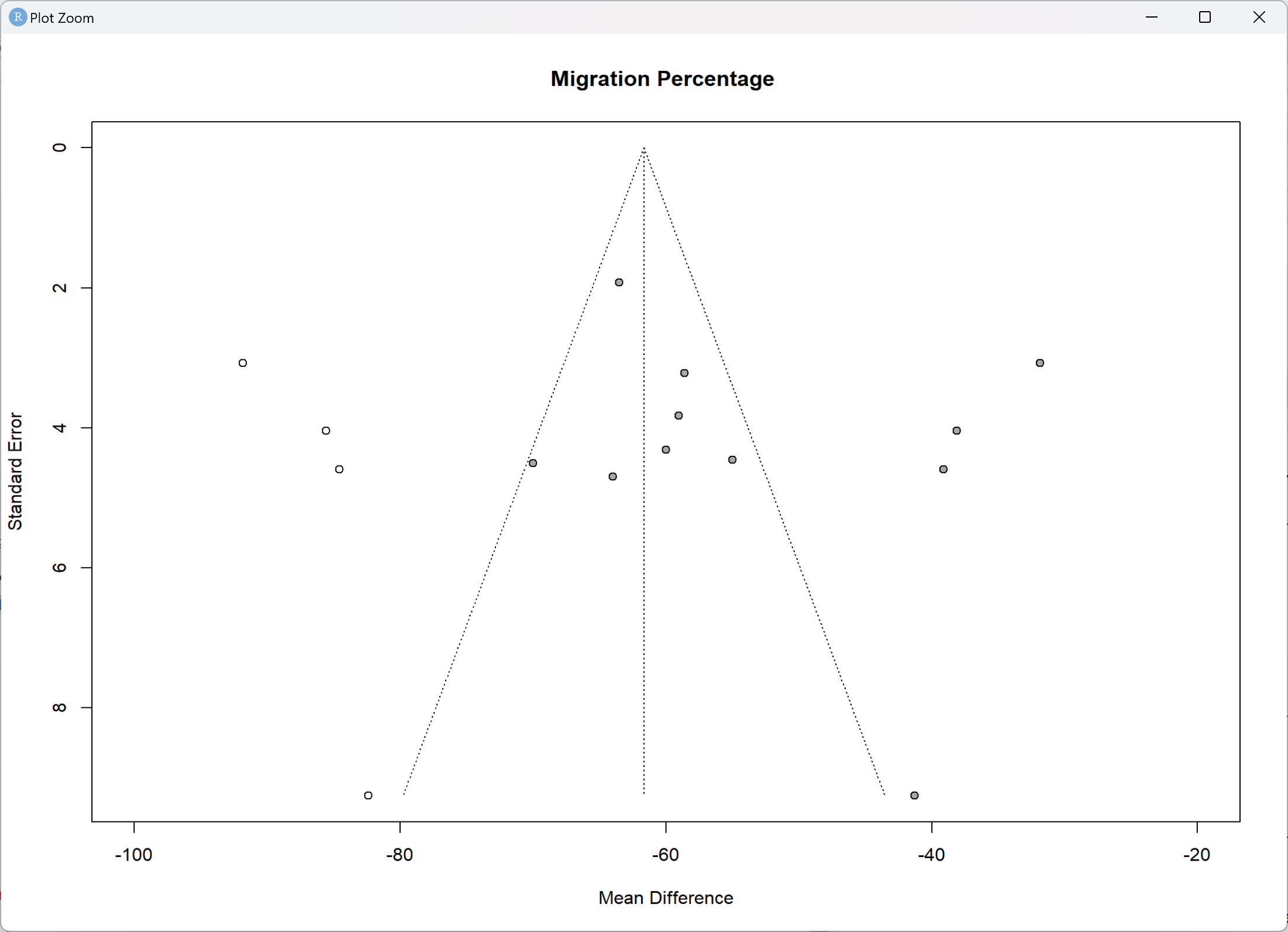


Supplementary Figure 47. Pelvic Osteotomy surgery, Migration Percentage


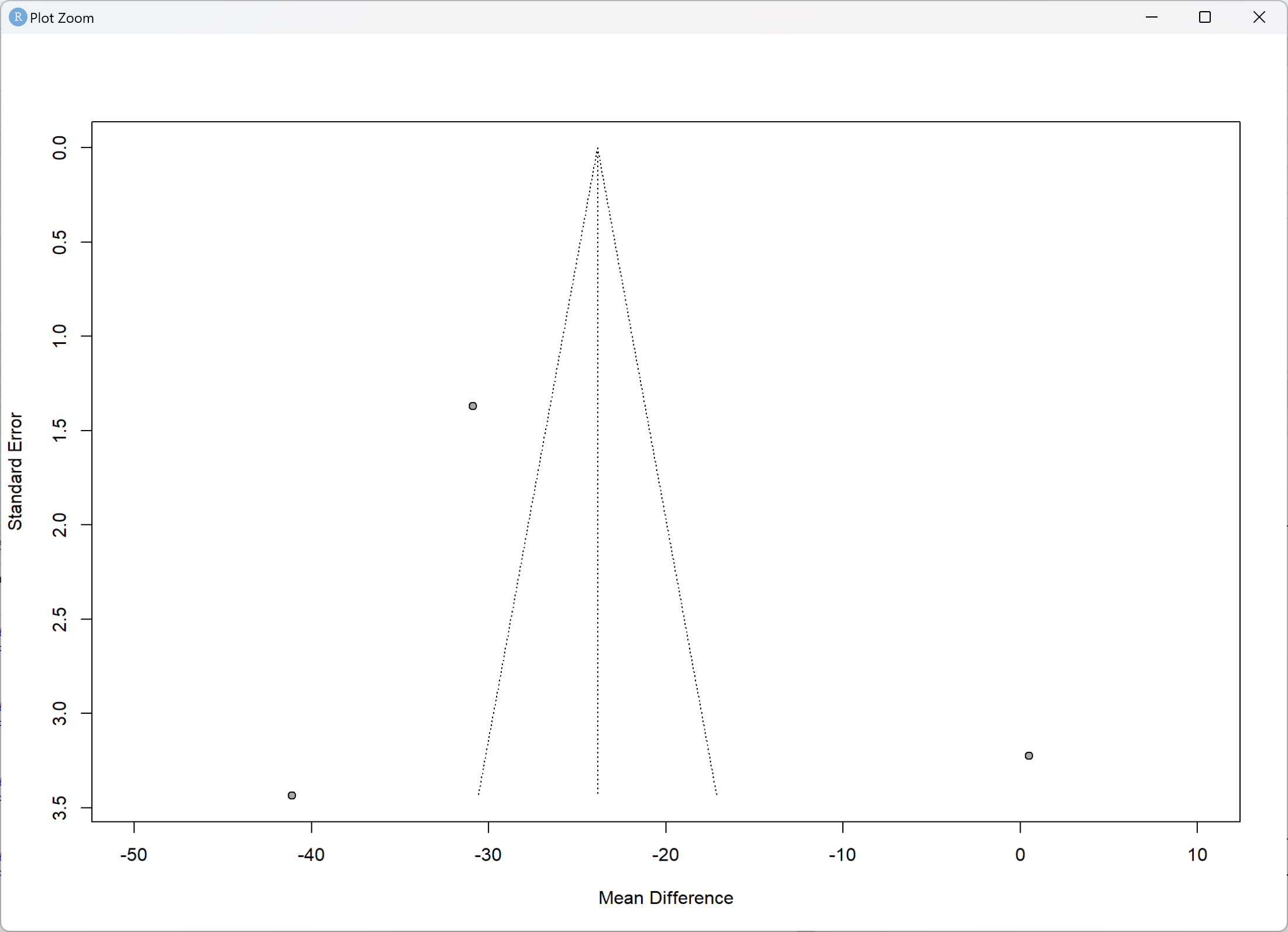


Supplementary Figure 48. Pelvic Osteotomy surgery, Neck Shaft Angle


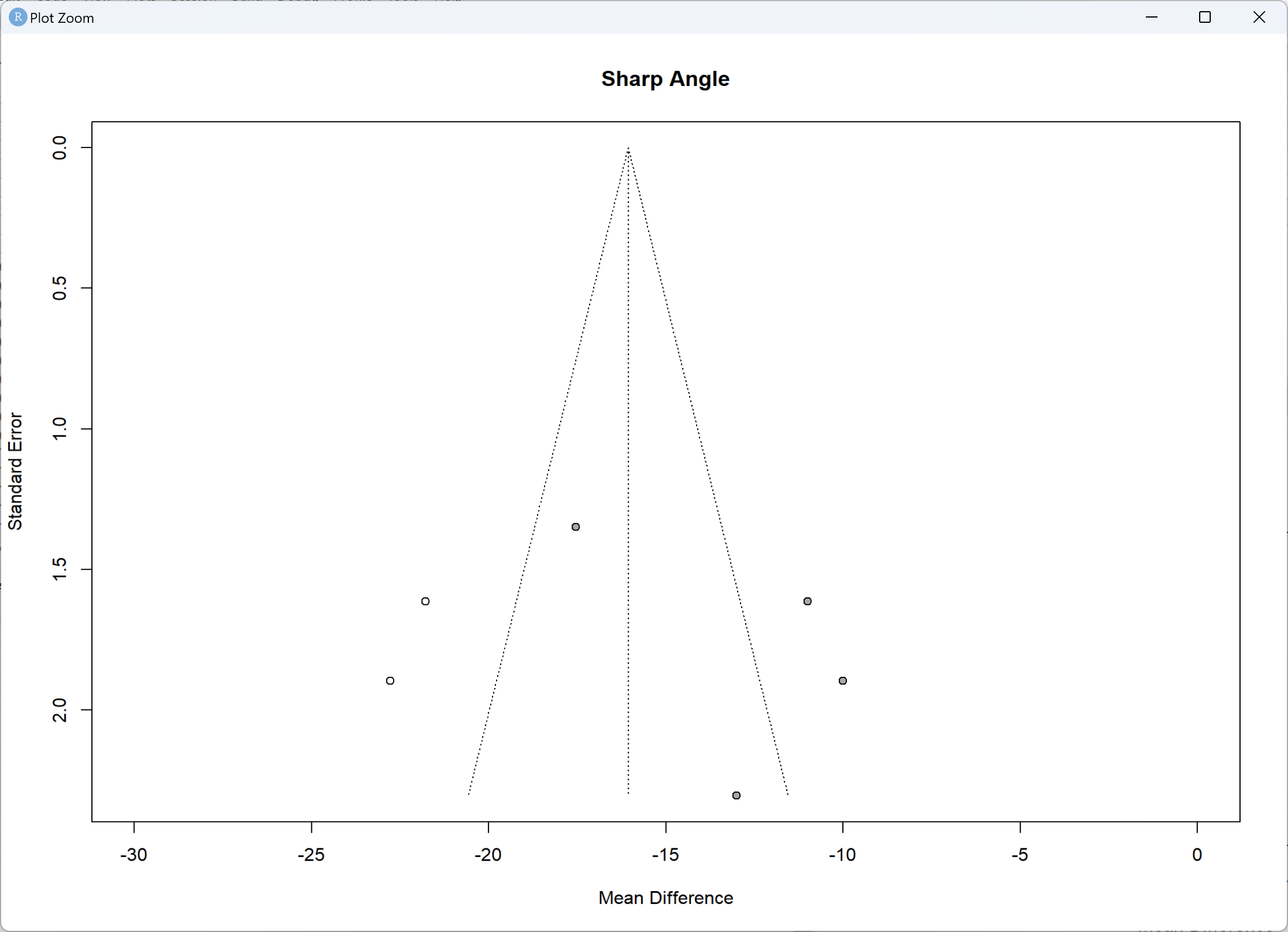


Supplementary Figure 49. Pelvic Osteotomy surgery, Sharp Angle


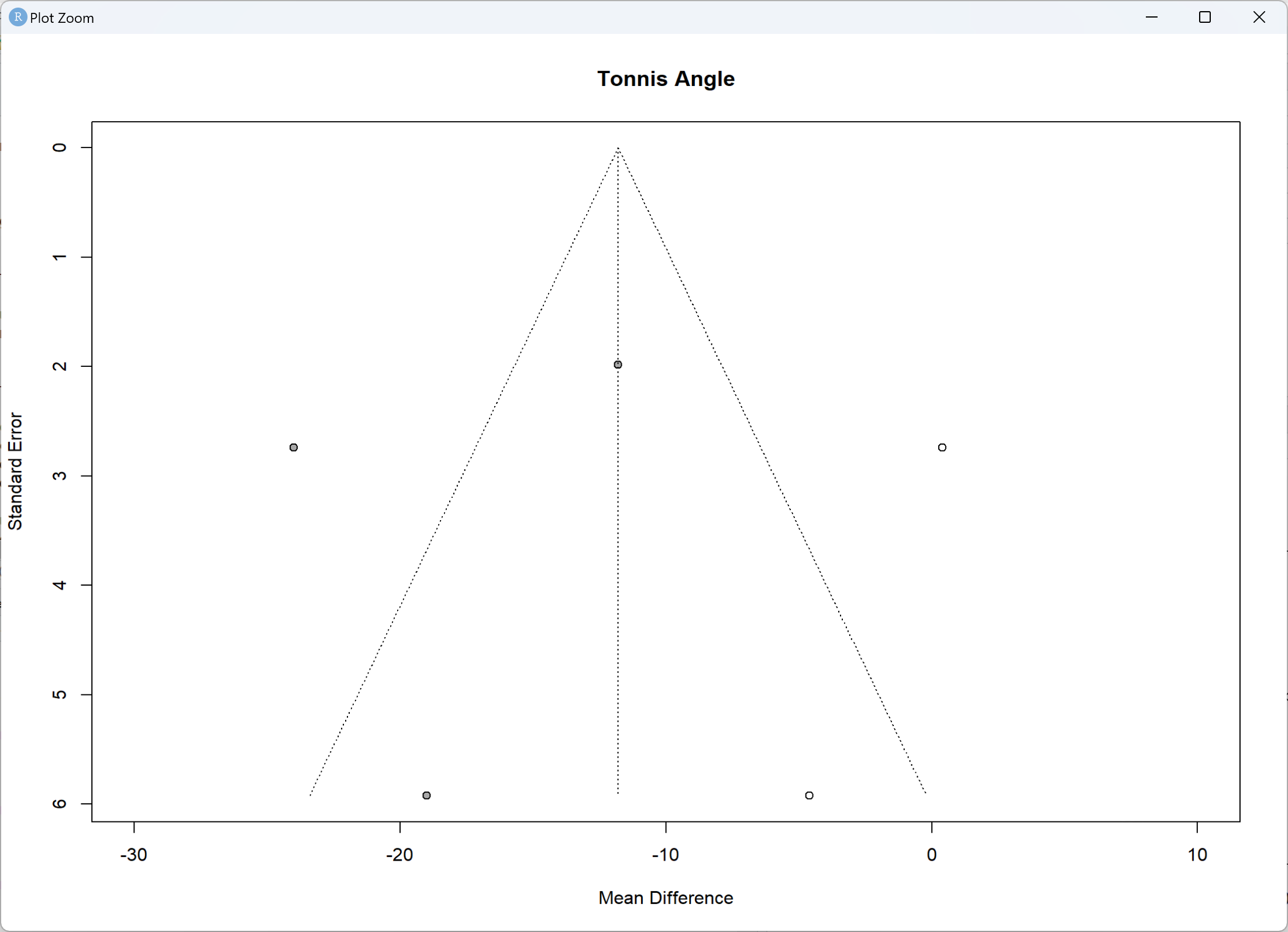


Supplementary Figure 50. Pelvic Osteotomy surgery, Tonnis Angle


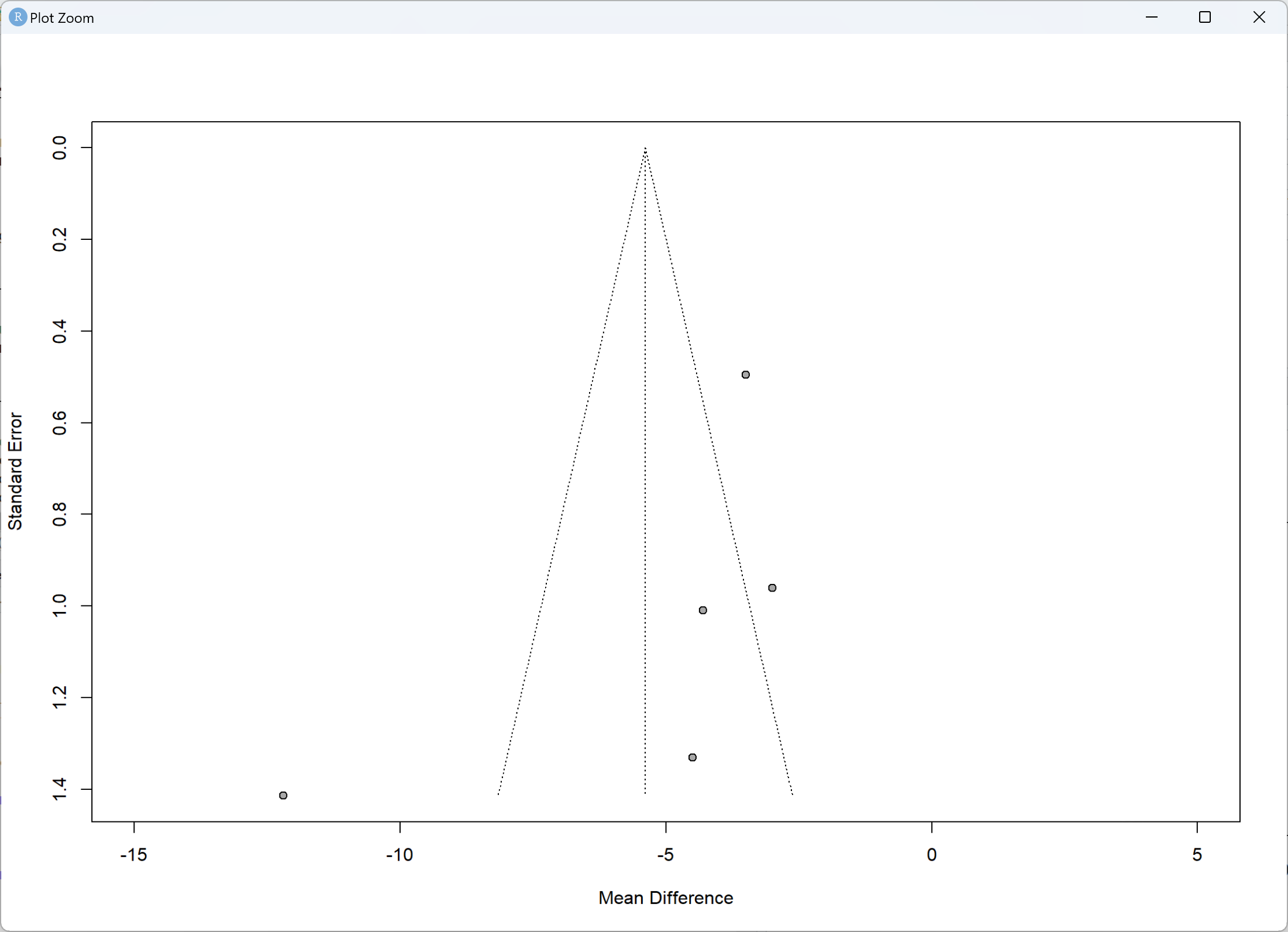


Supplementary Figure 51. Femur Osteotomy surgery, Acetabular Index


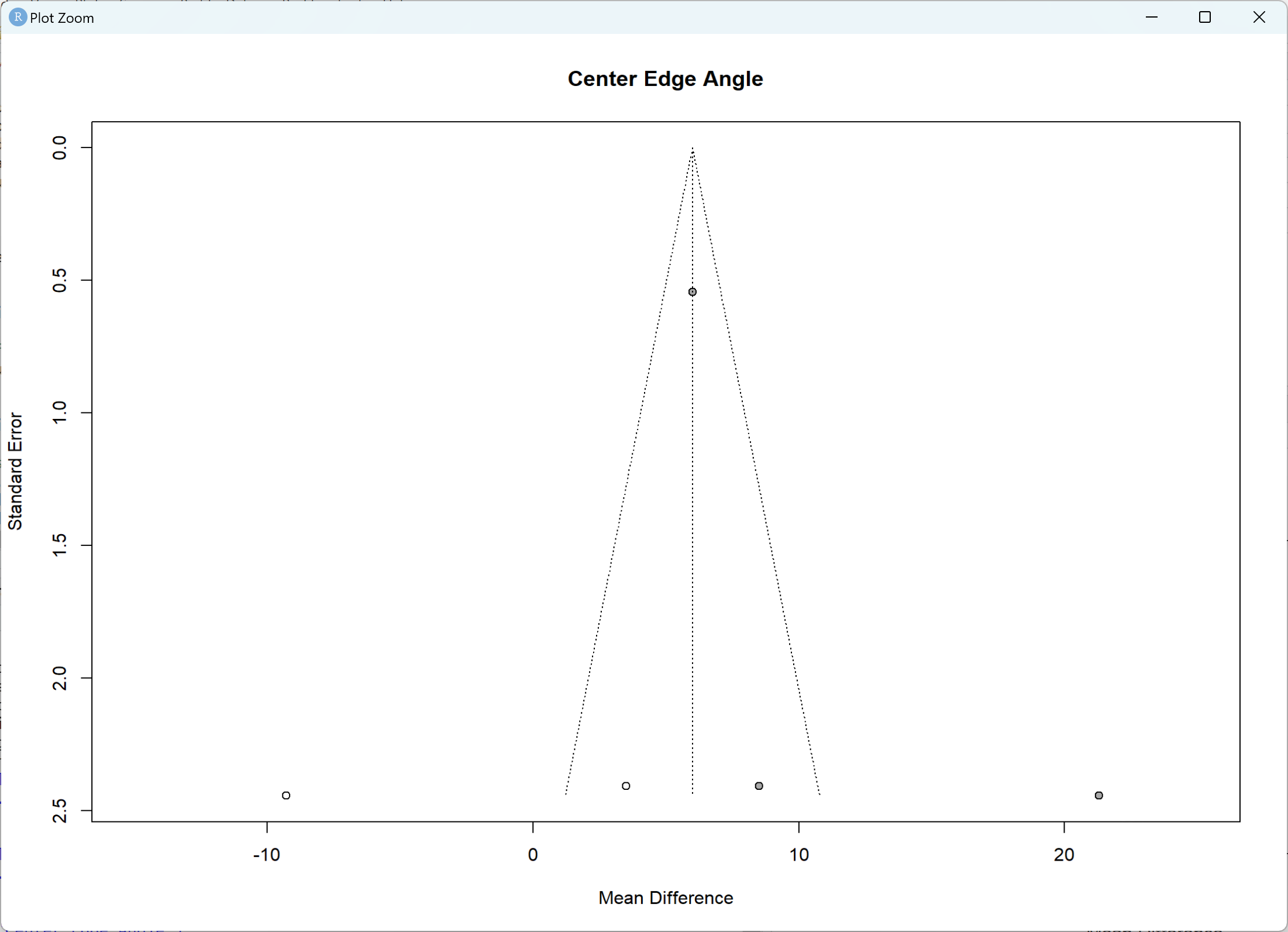


Supplementary Figure 52. Femur Osteotomy surgery, Center Edge Angle


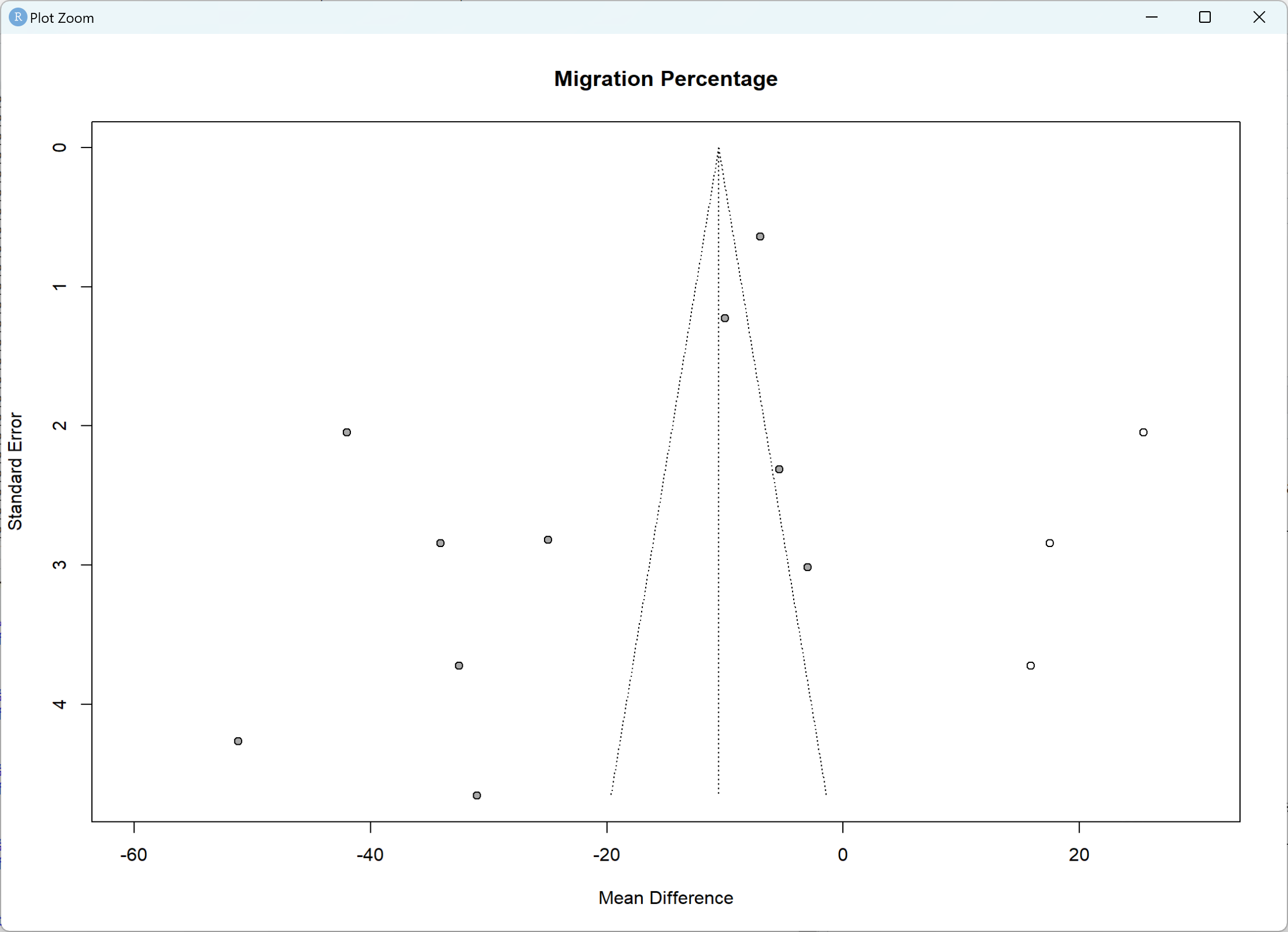


Supplementary Figure 53. Femur Osteotomy surgery, Migration Percentage


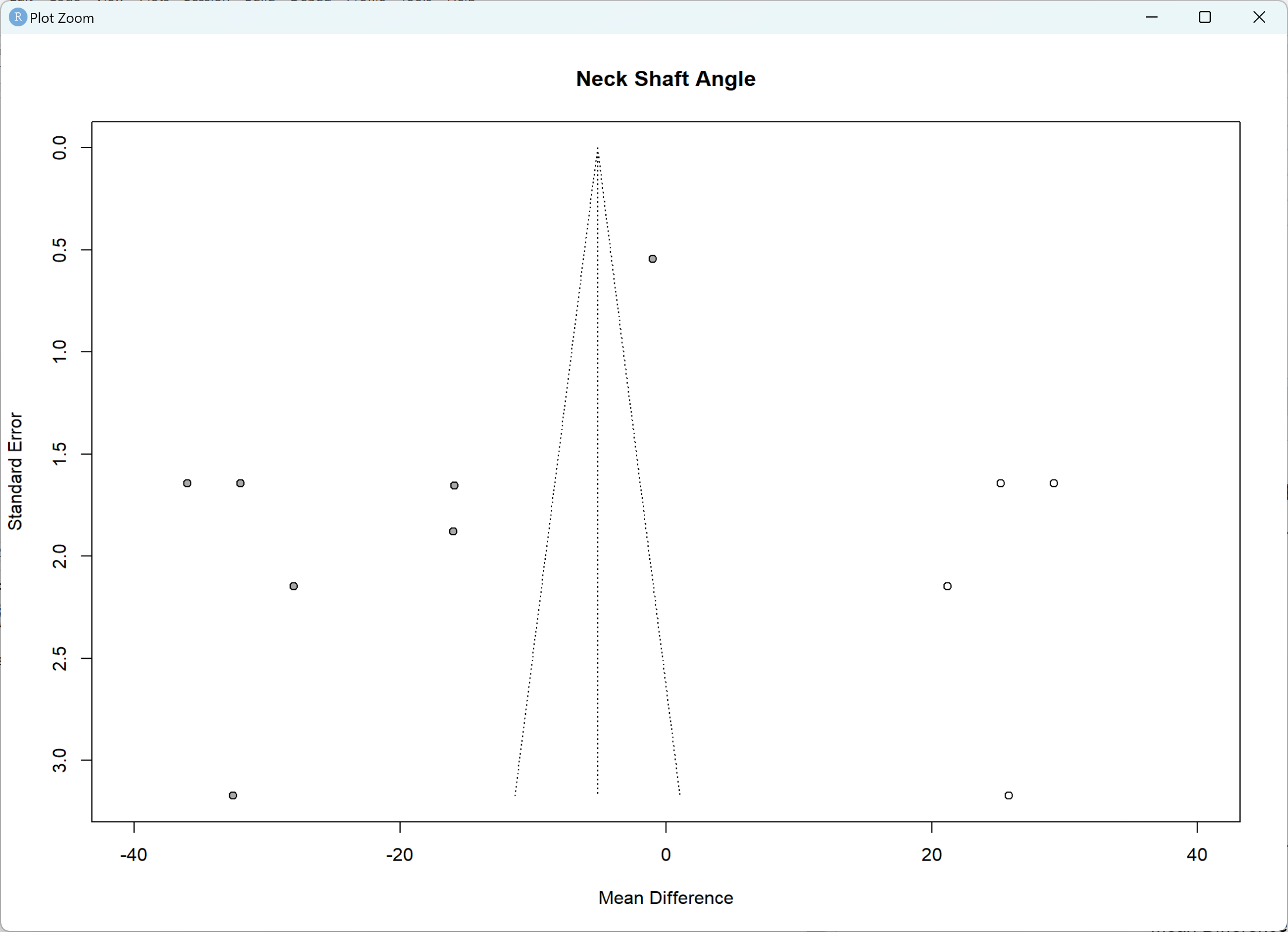


Supplementary Figure 54. Femur Osteotomy surgery, Neck Shaft Angle


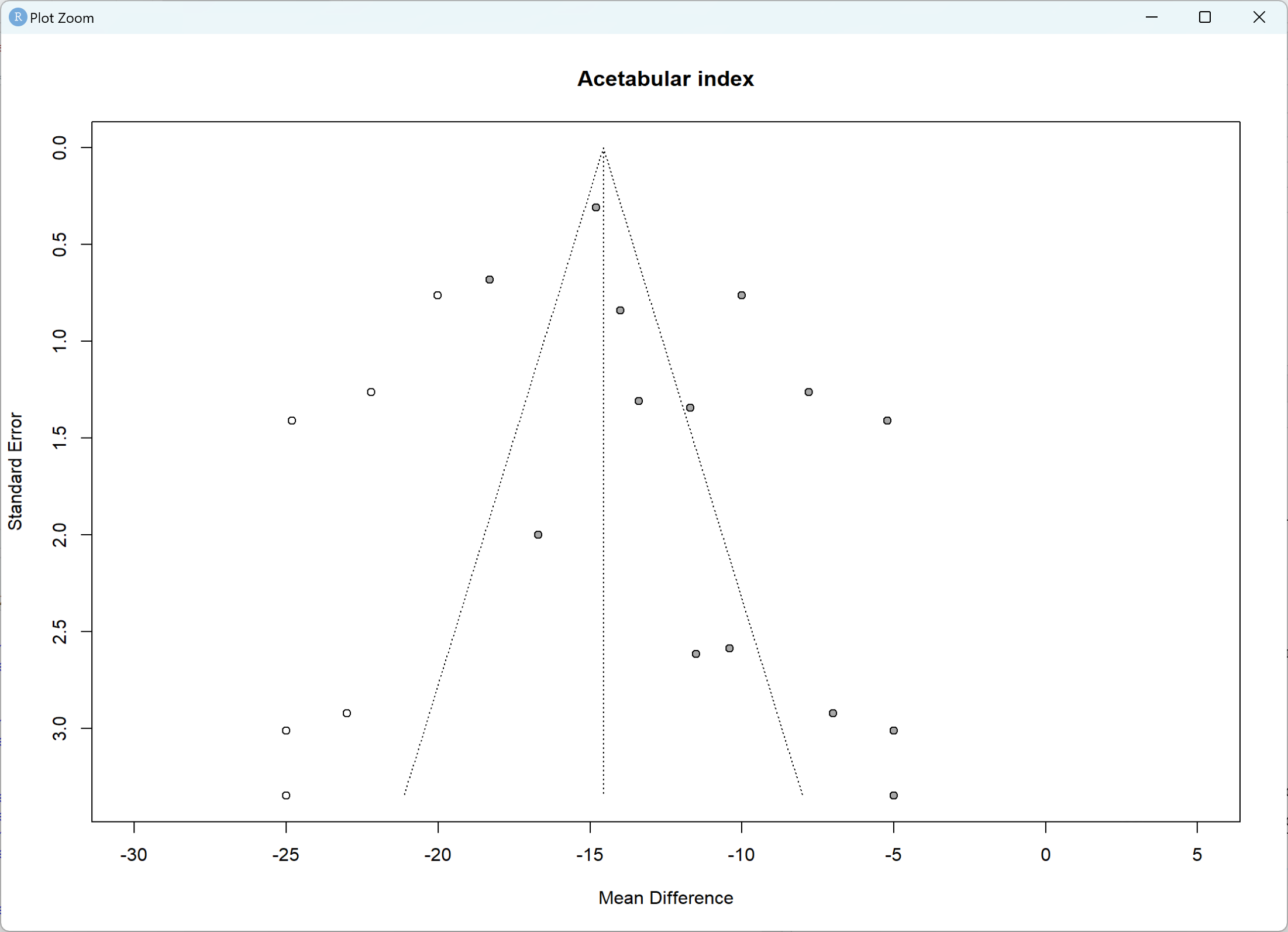


Supplementary Figure 55. Combination of Pelvic and Femur Osteotomy surgery, Acetabular Index


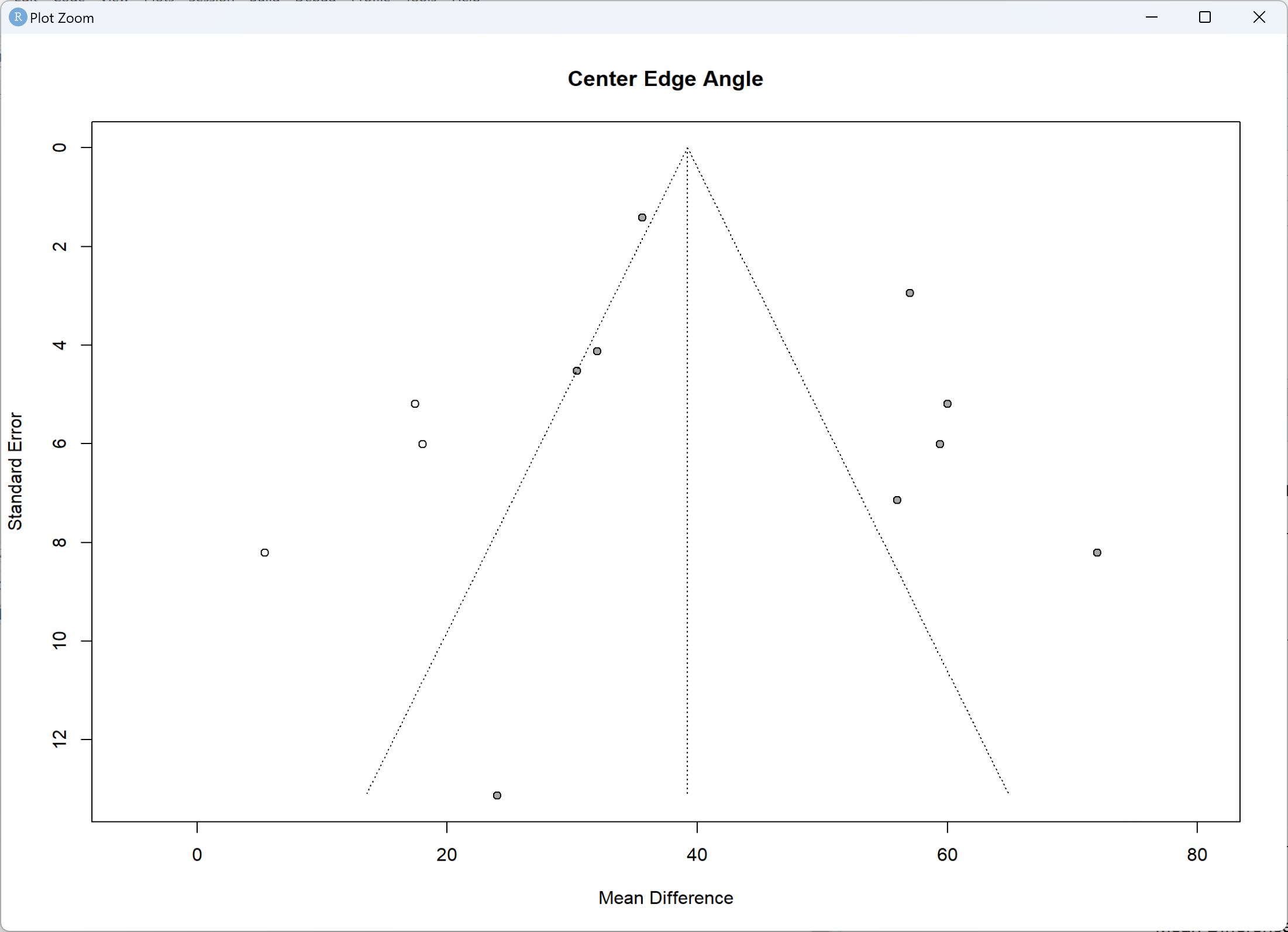


Supplementary Figure 56. Combination of Pelvic and Femur Osteotomy surgery, Center Edge Angle


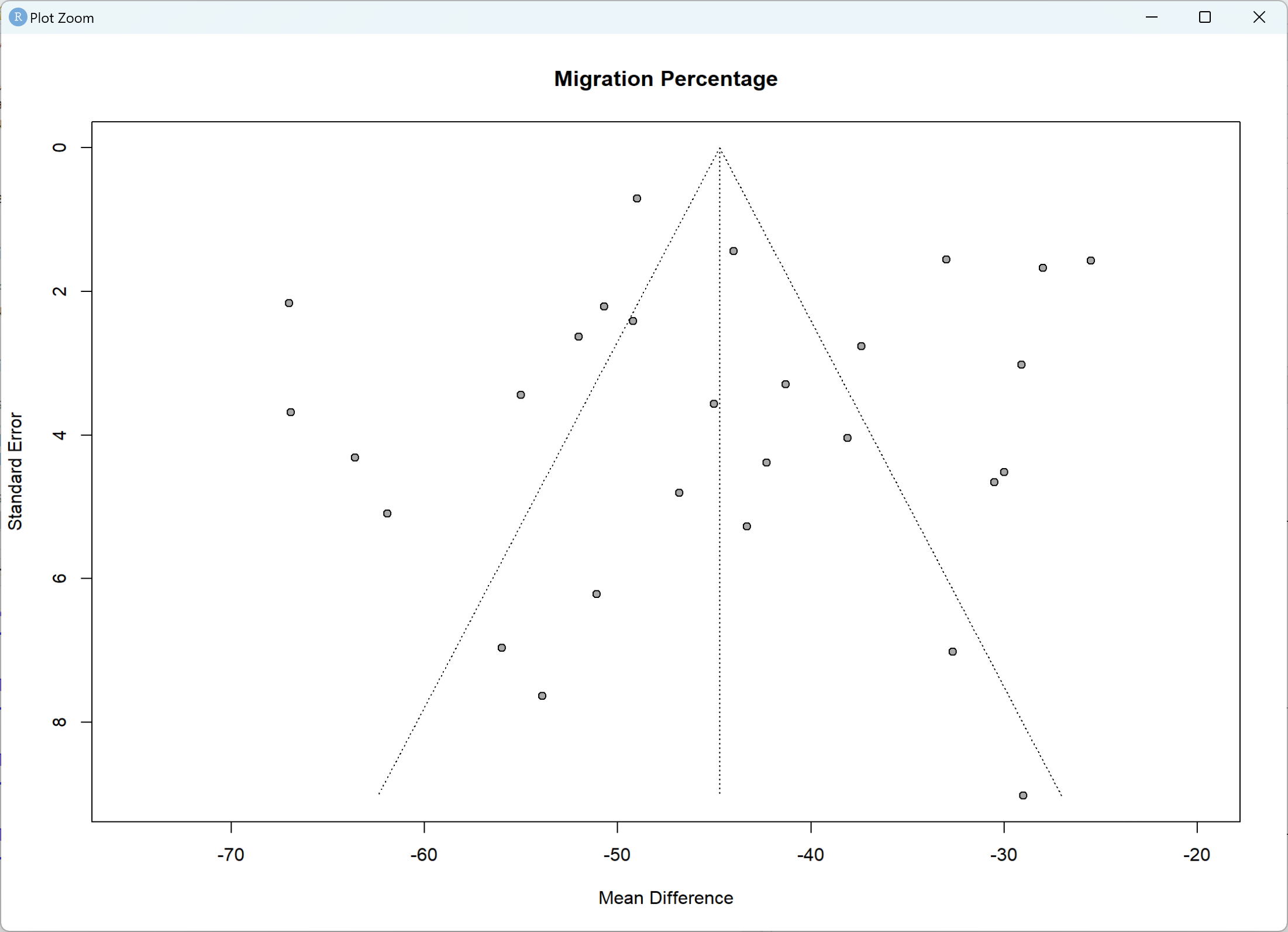


Supplementary Figure 57. Combination of Pelvic and Femur Osteotomy surgery, Migration Percentage


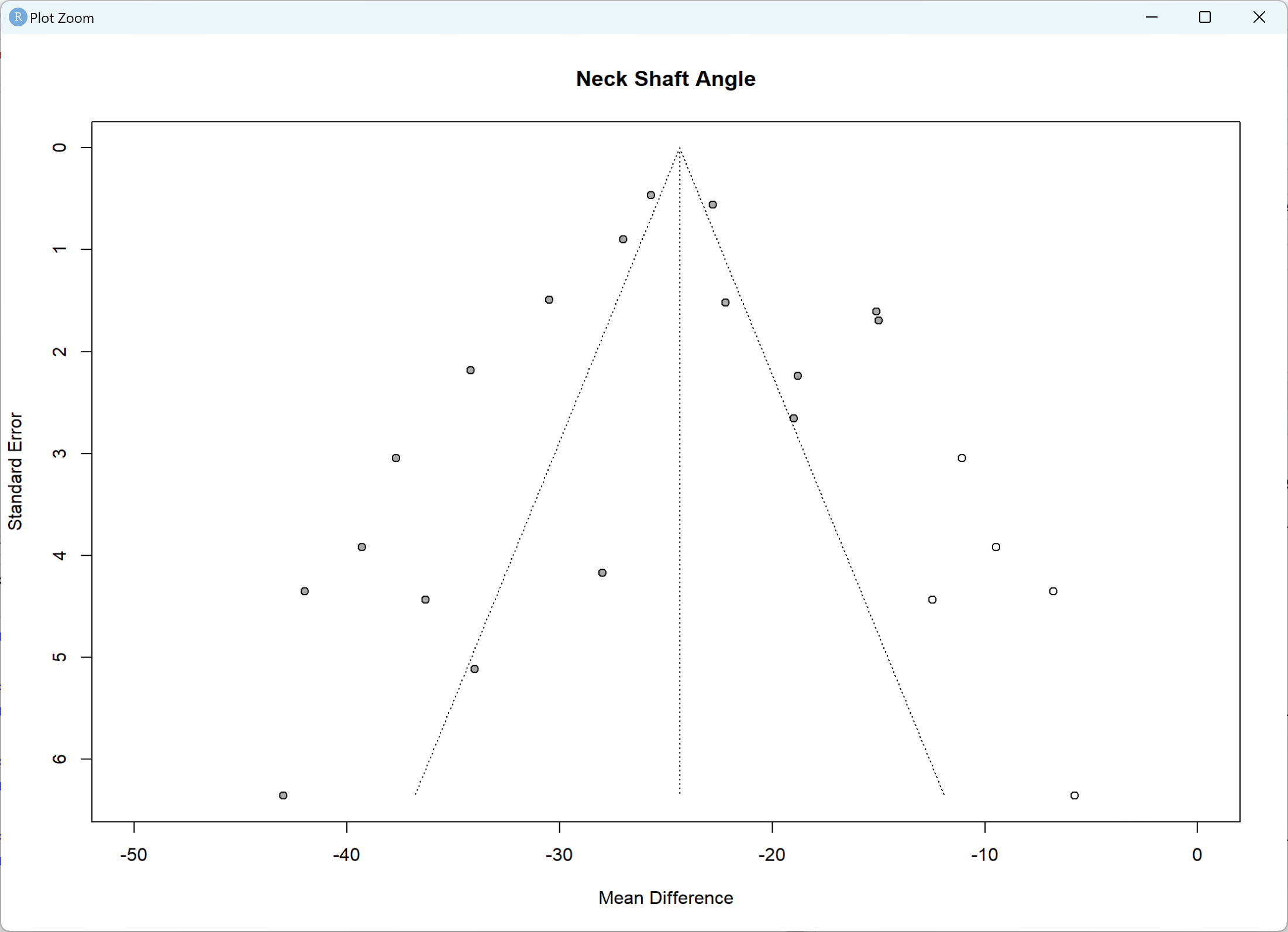


Supplementary Figure 58. Combination of Pelvic and Femur Osteotomy surgery, Neck Shaft Angle


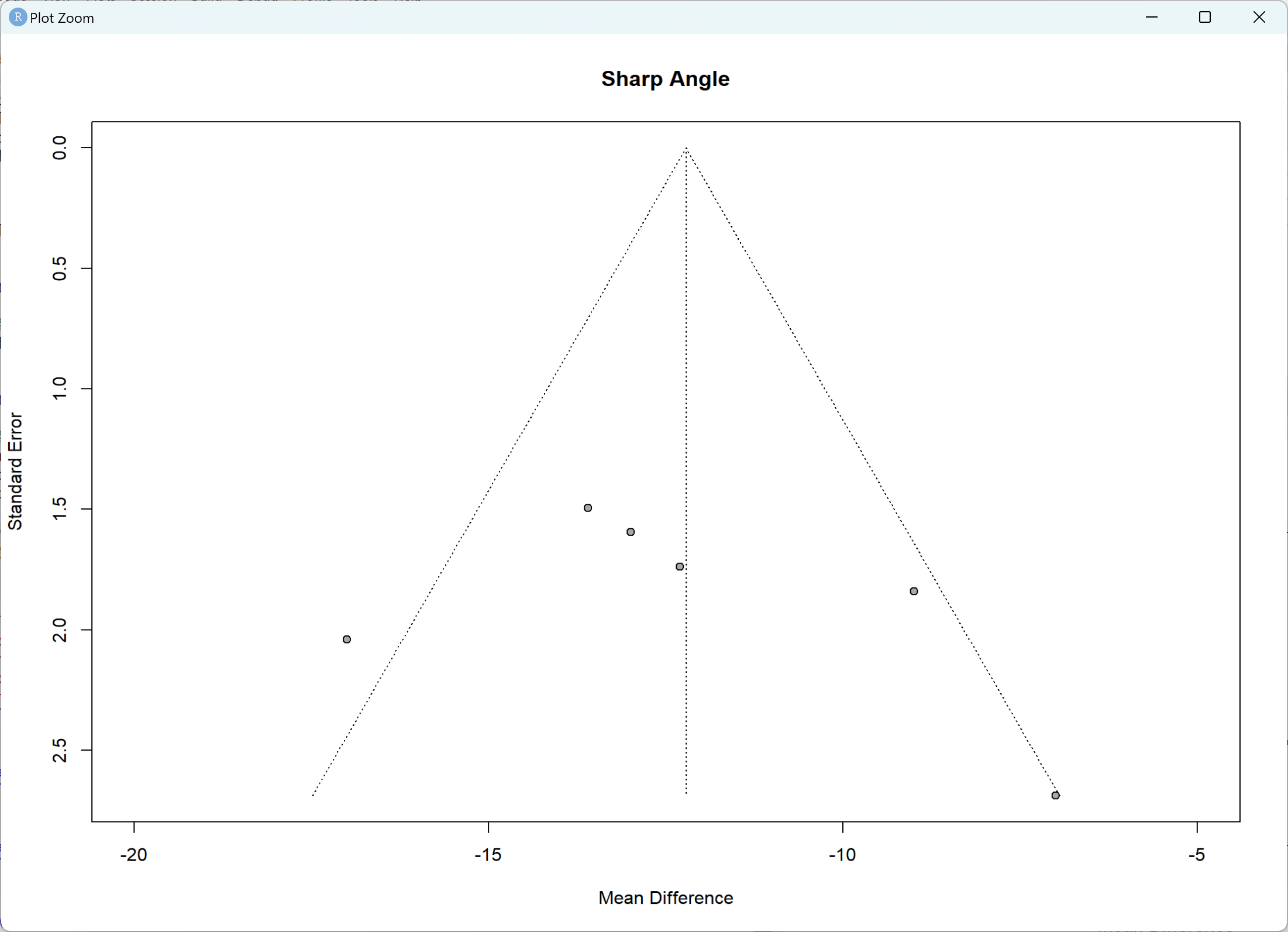


Supplementary Figure 59. Combination of Pelvic and Femur Osteotomy surgery, Sharp Angle


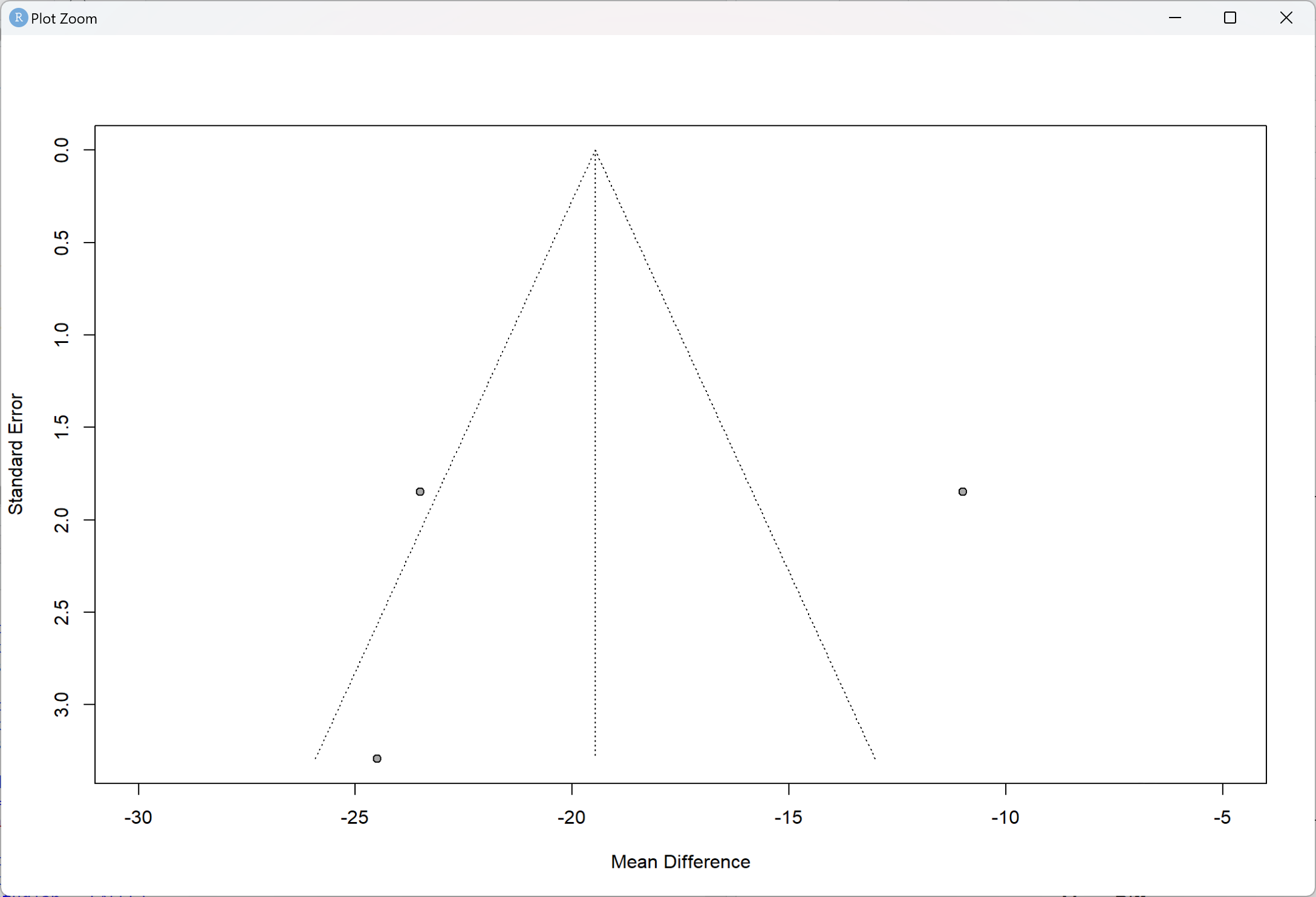


Supplementary Figure 60. Combination of Pelvic and Femur Osteotomy surgery, Tonnis Angle


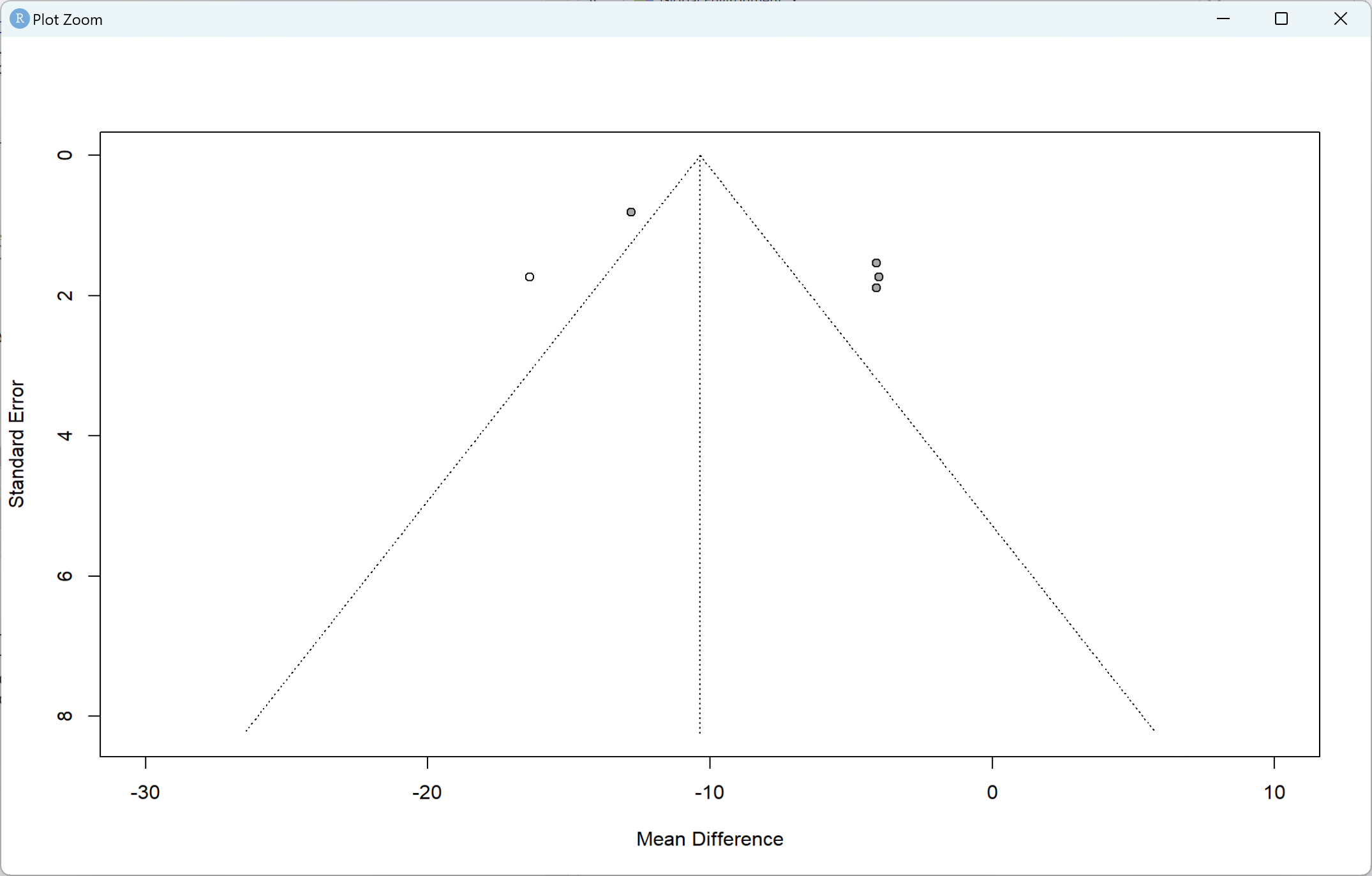


Supplementary Figure 61. Soft tissue surgery, Acetabular Index


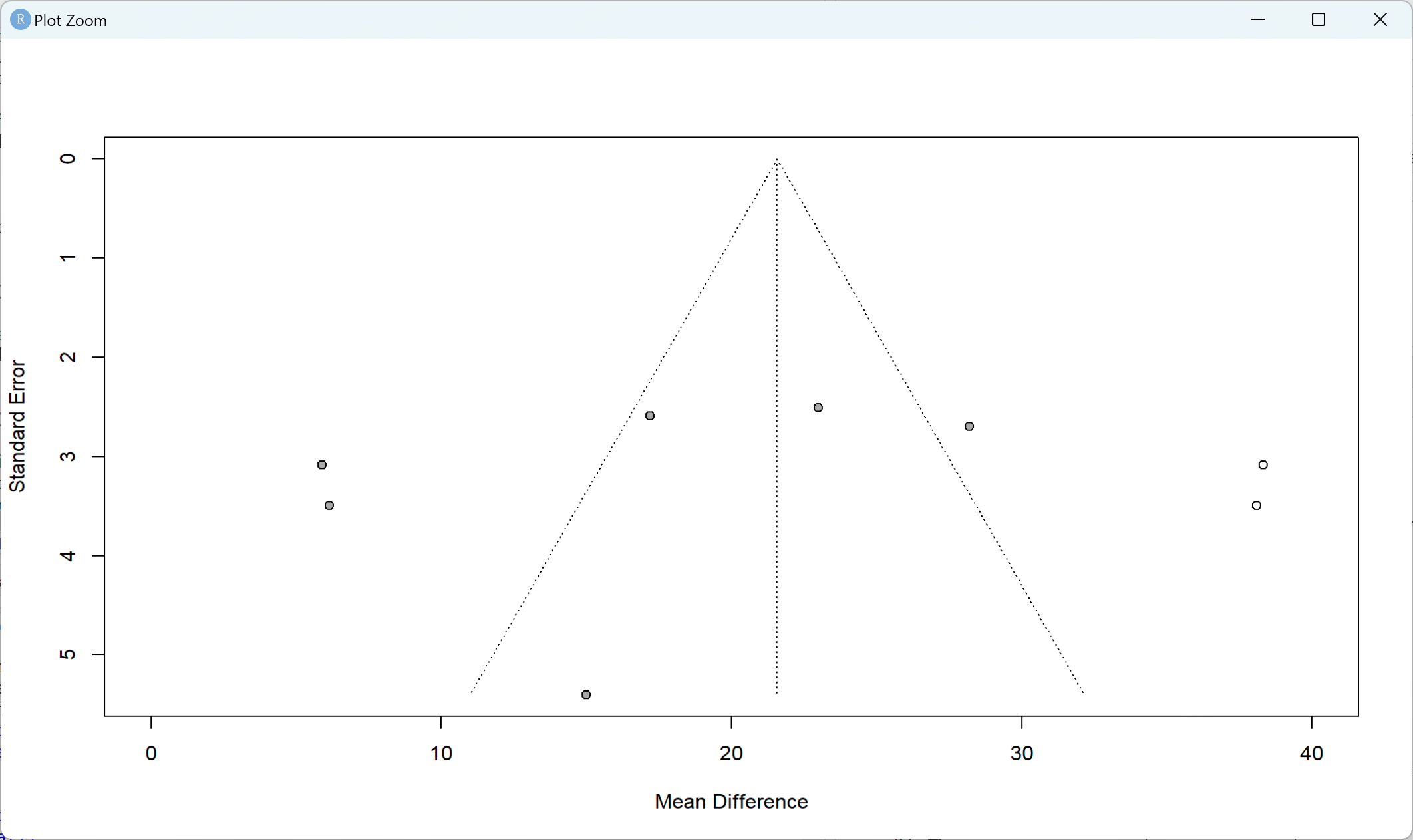


Supplementary Figure 62. Soft tissue surgery, Center Edge Angle


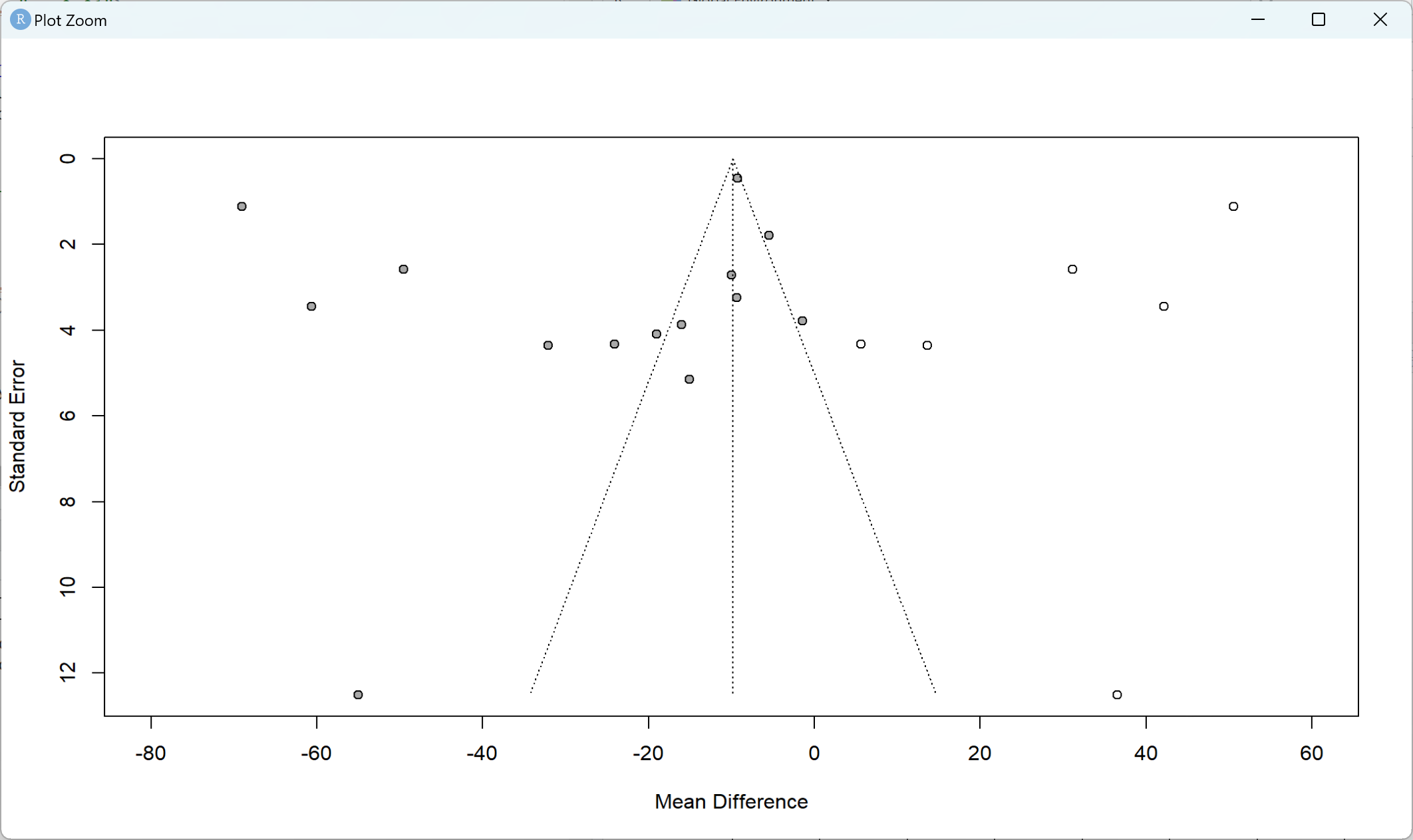


Supplementary Figure 63. Soft tissue surgery, Migration Percentage


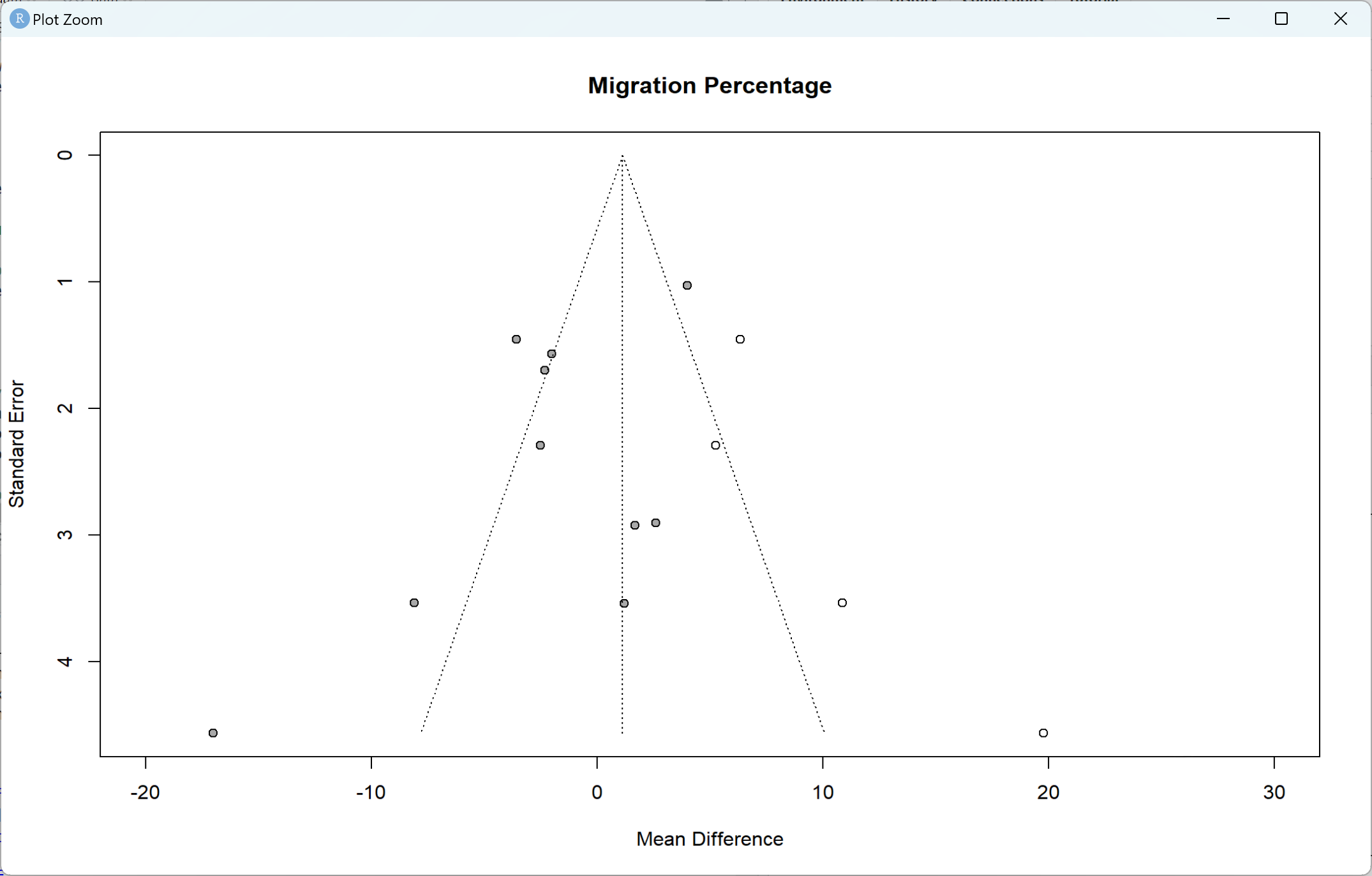


Supplementary Figure 64. Tone decrease surgery, Migration Percentage


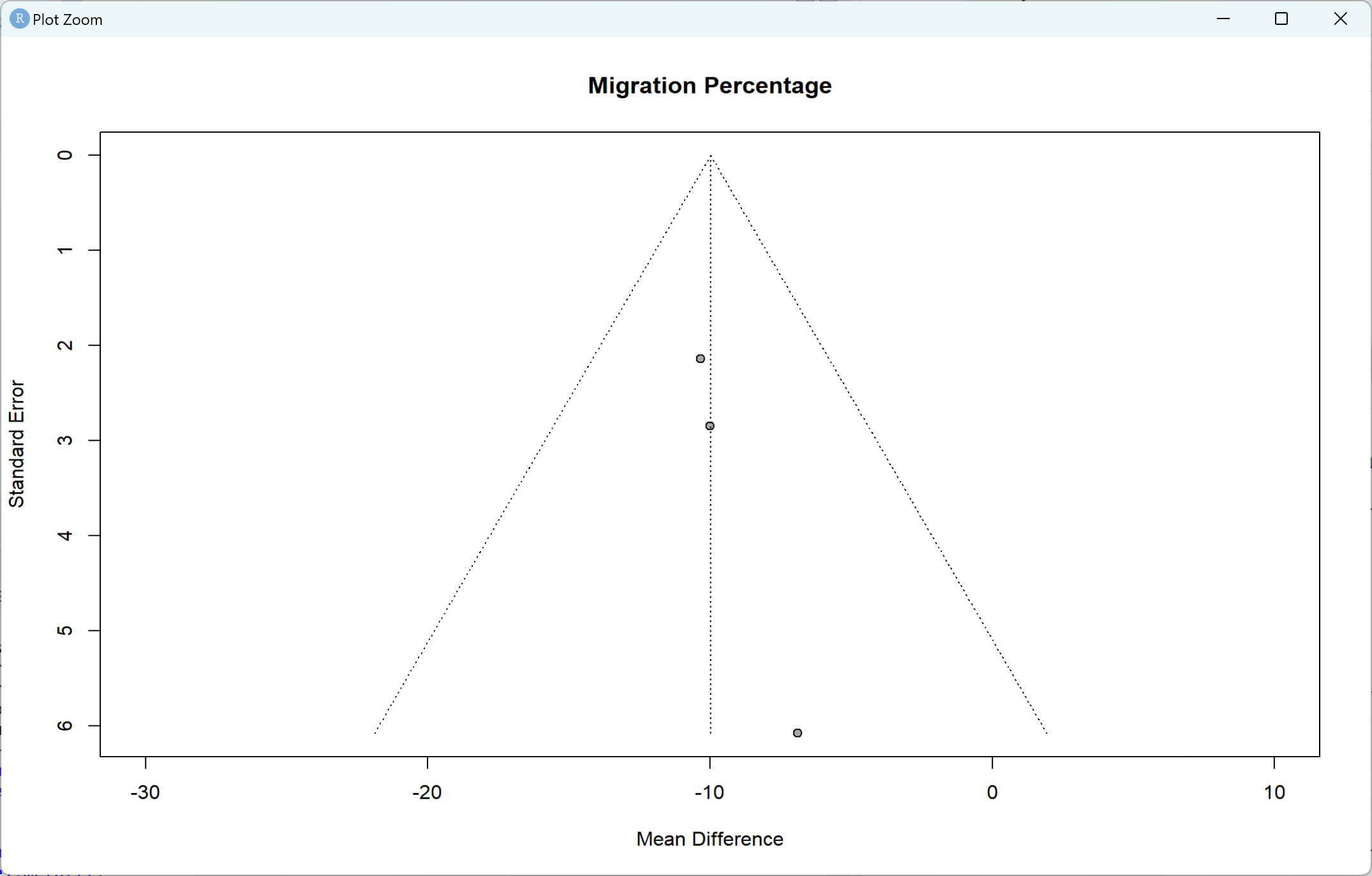


Supplementary Figure 65. Guided growth surgery, Migration Percentage


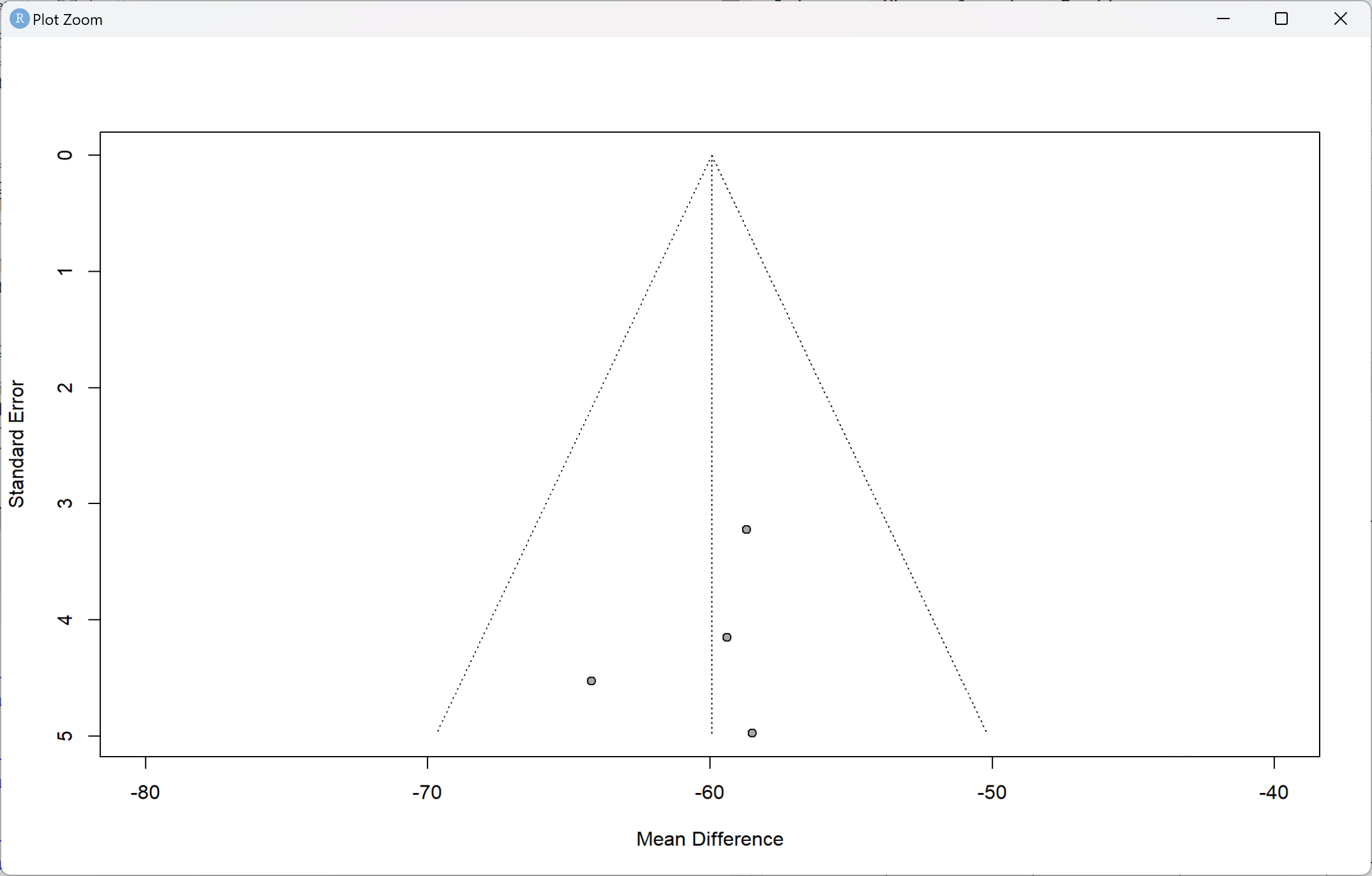


Supplementary Figure 66. Percutaneous Pelvic Osteotomy, Migration Percentage

**Appendix E (Leave-One-Out Meta-Analysis Results)**


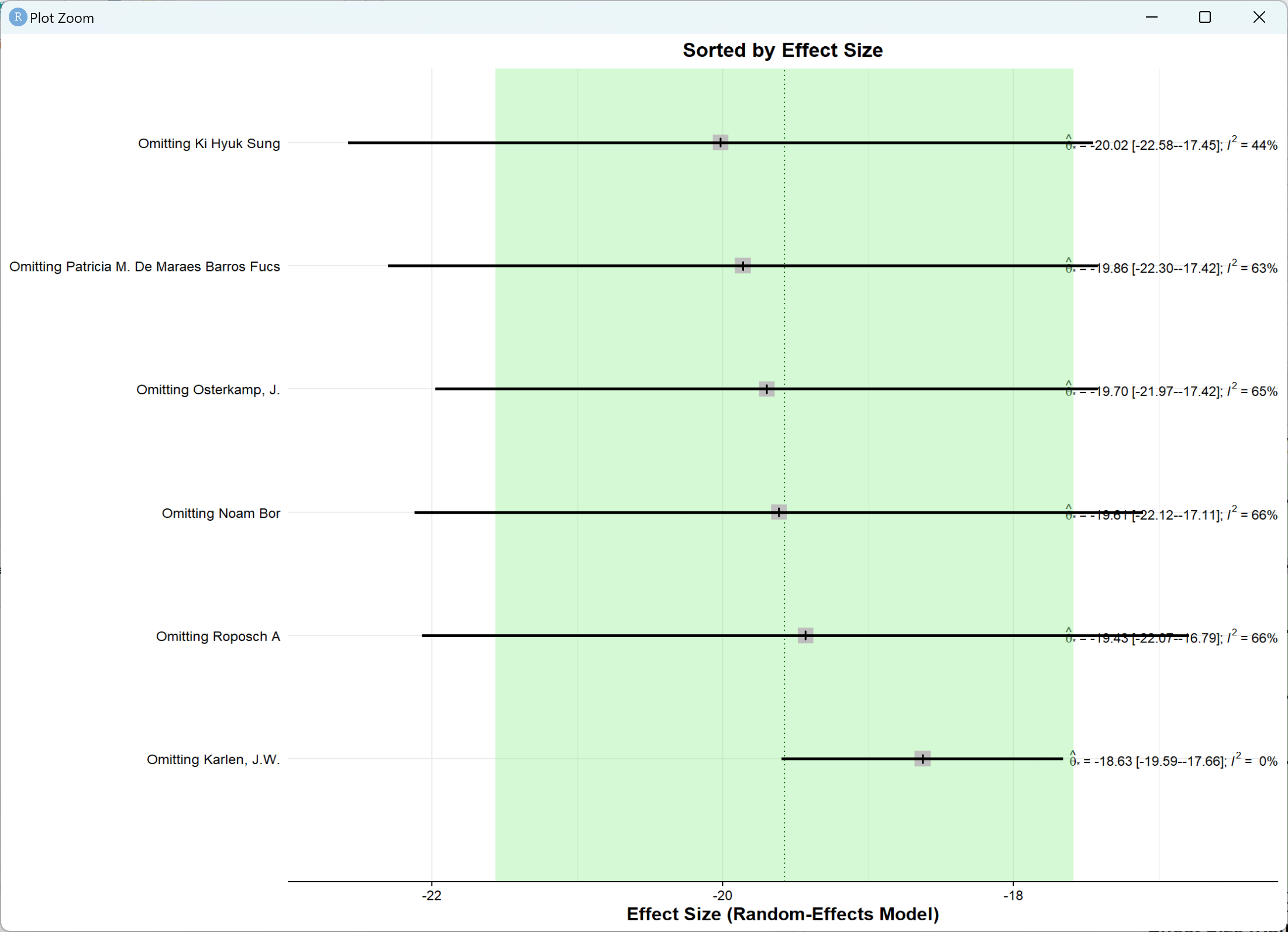


Supplementary Figure 67. Pelvic Osteotomy, Acetabular Index


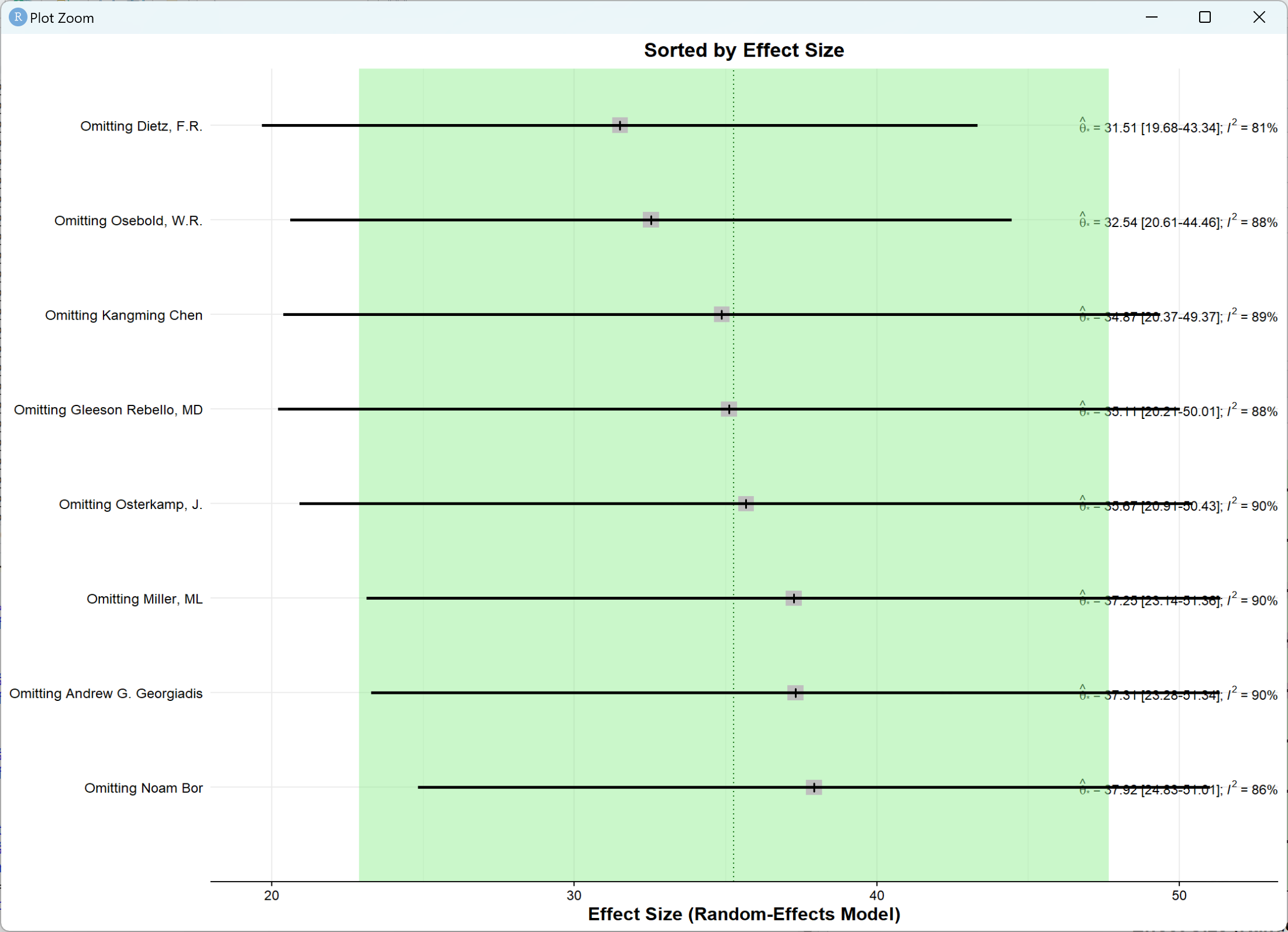


Supplementary Figure 68. Pelvic Osteotomy, Center Edge Angle


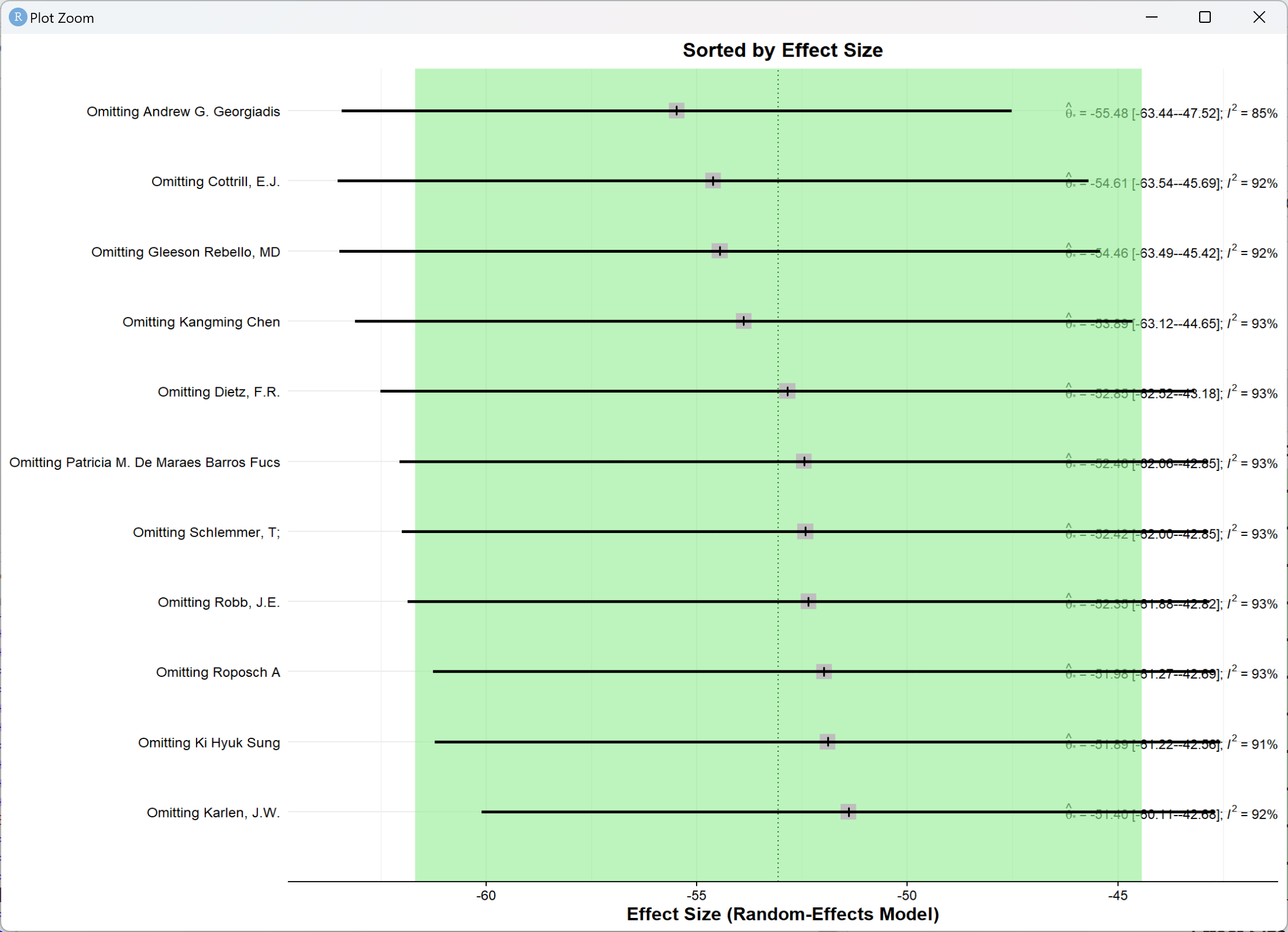


Supplementary Figure 69. Pelvic Osteotomy, Migration Percentage


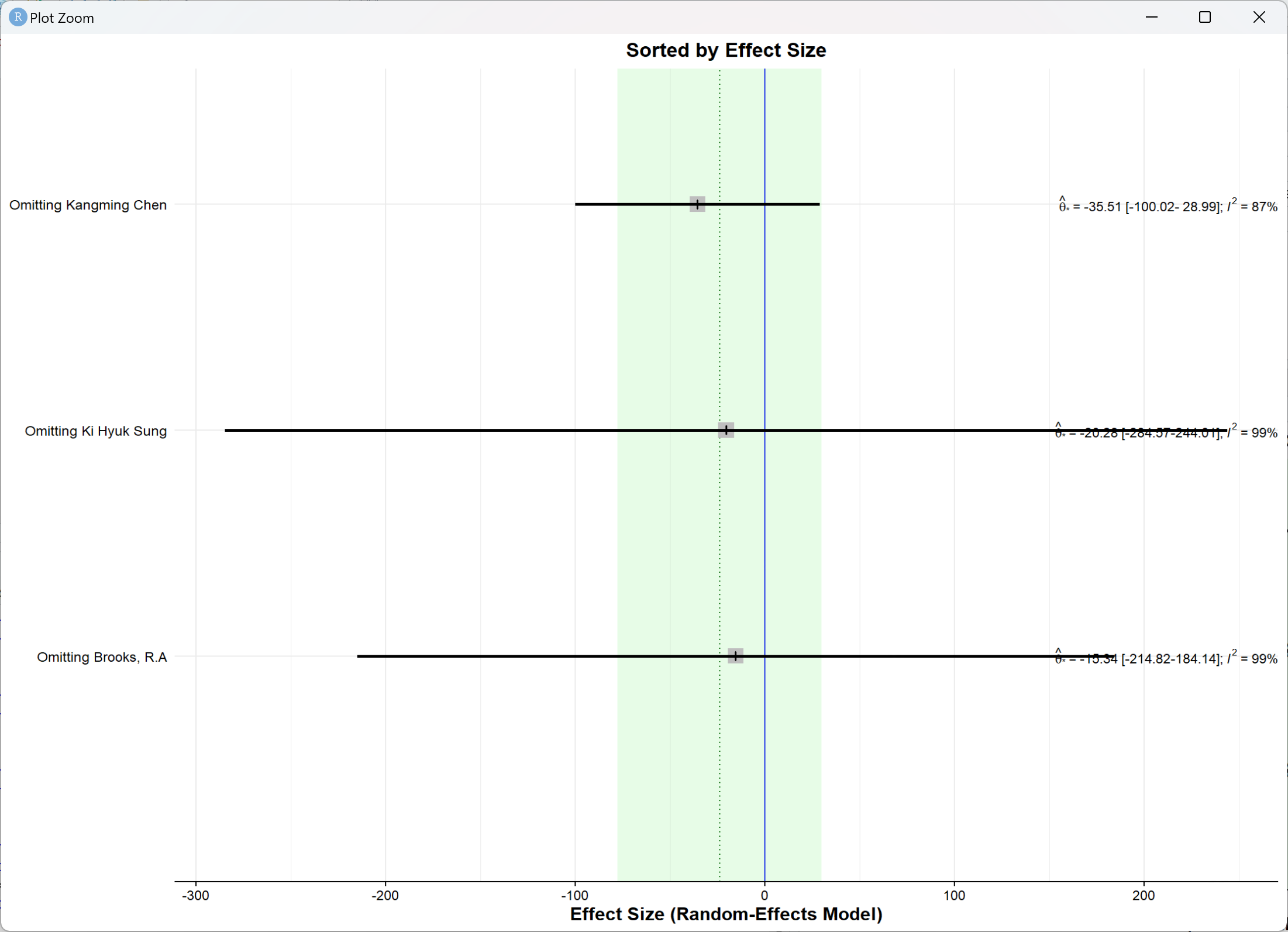


Supplementary Figure 70. Pelvic Osteotomy, Neck Shaft Angle


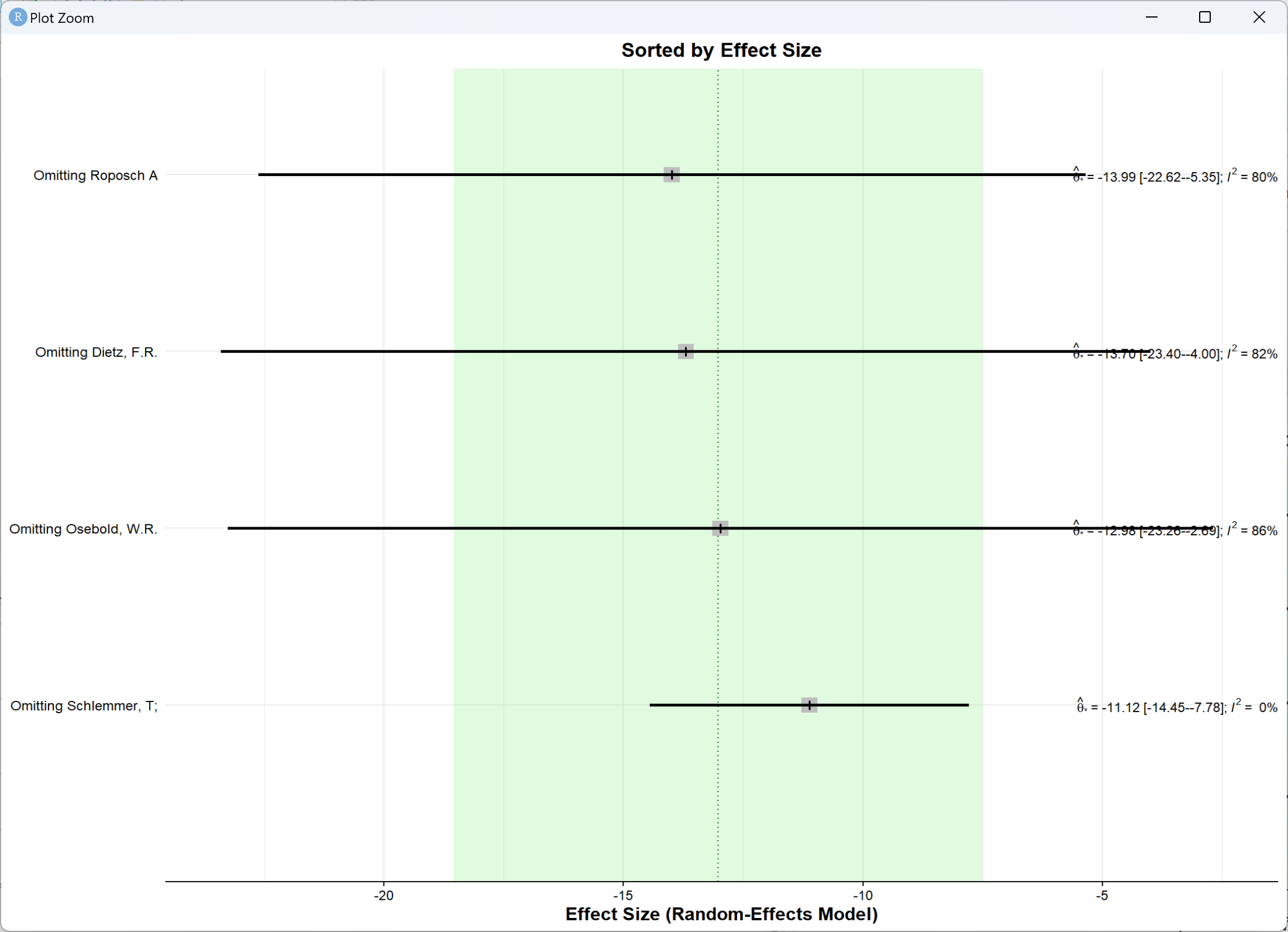


Supplementary Figure 71. Pelvic Osteotomy, Sharp Angle


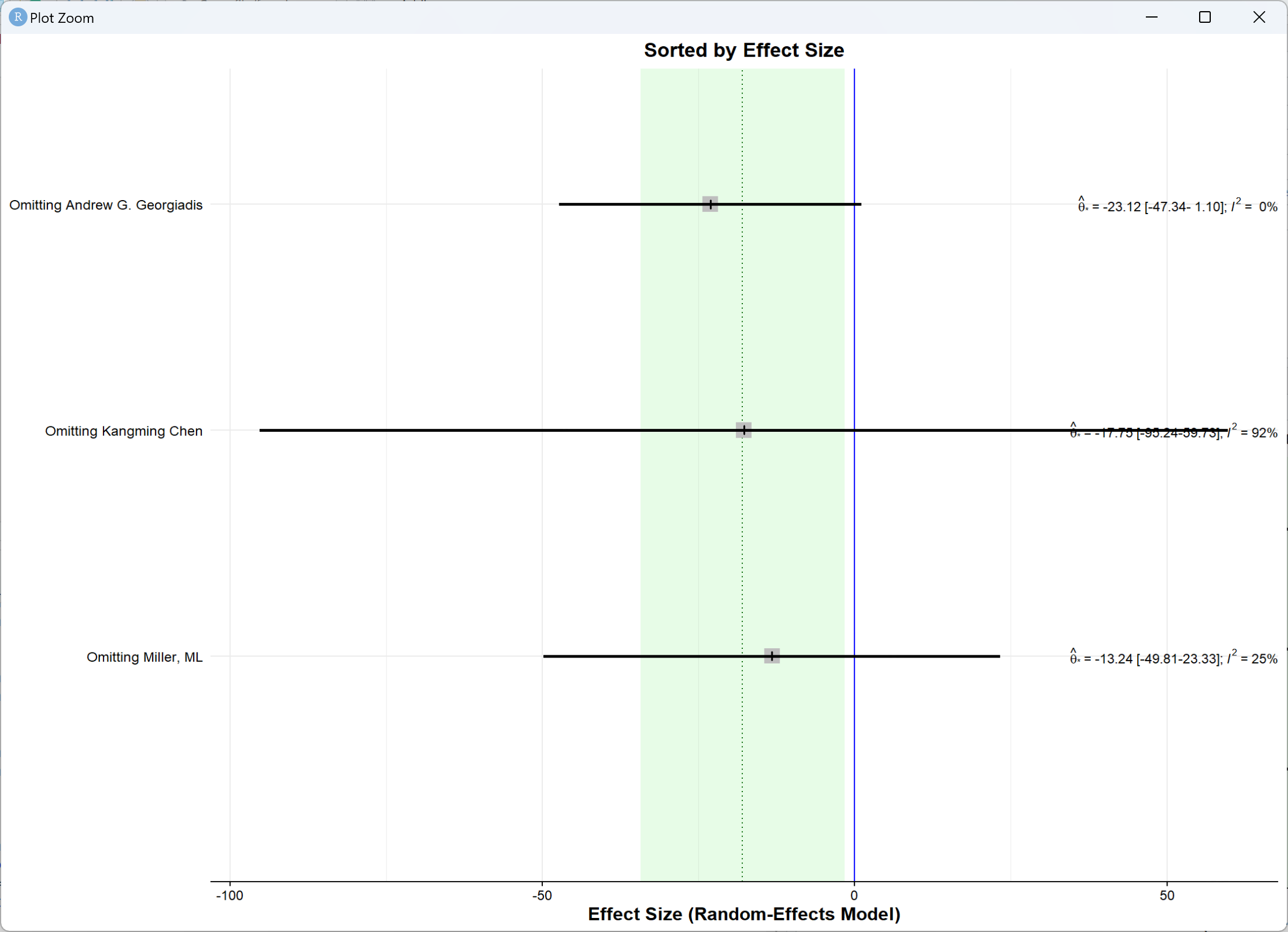


Supplementary Figure 72. Pelvic Osteotomy, Tonnis Angle


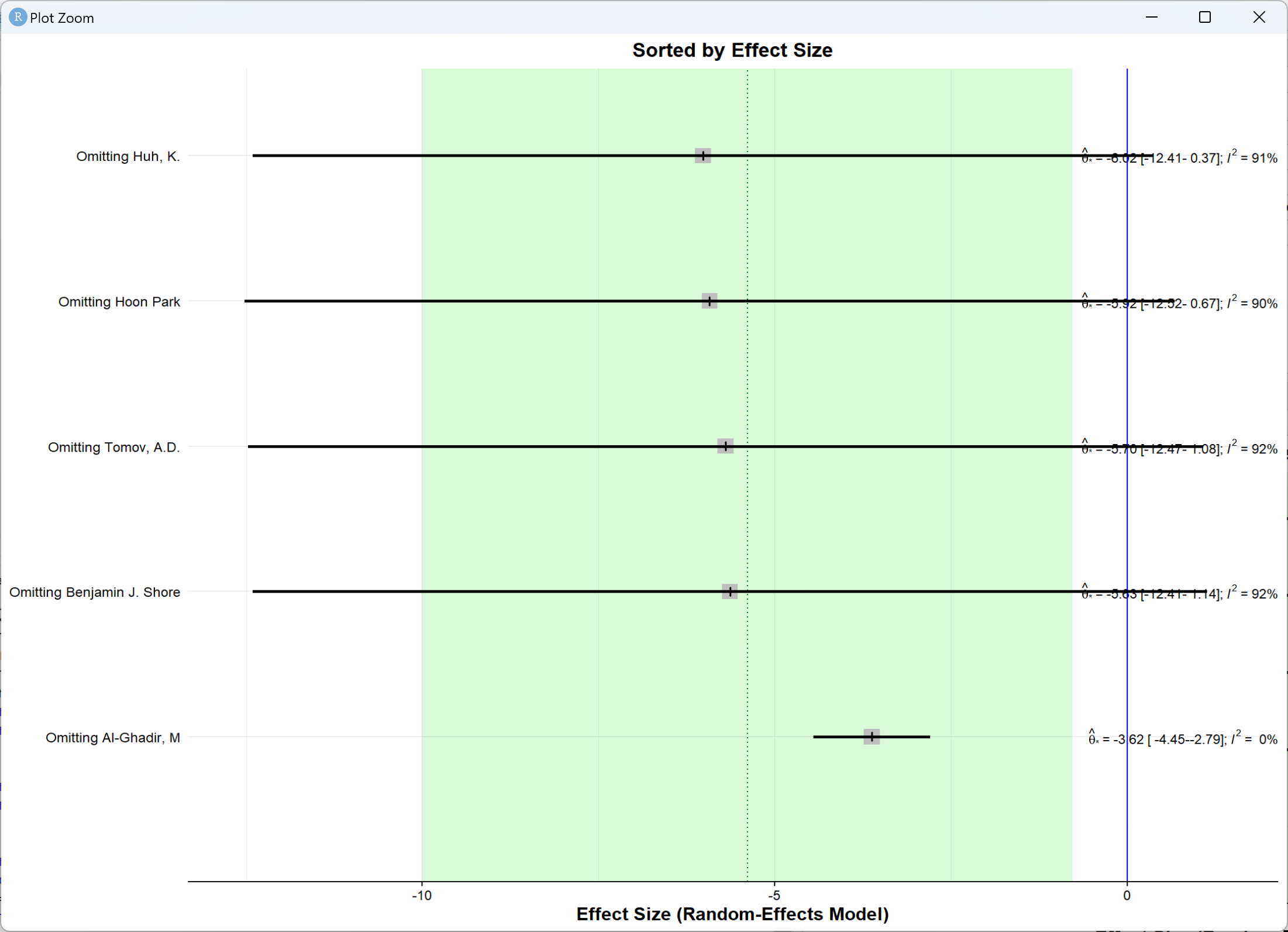


Supplementary Figure 73. Femur Osteotomy, Acetabular Index


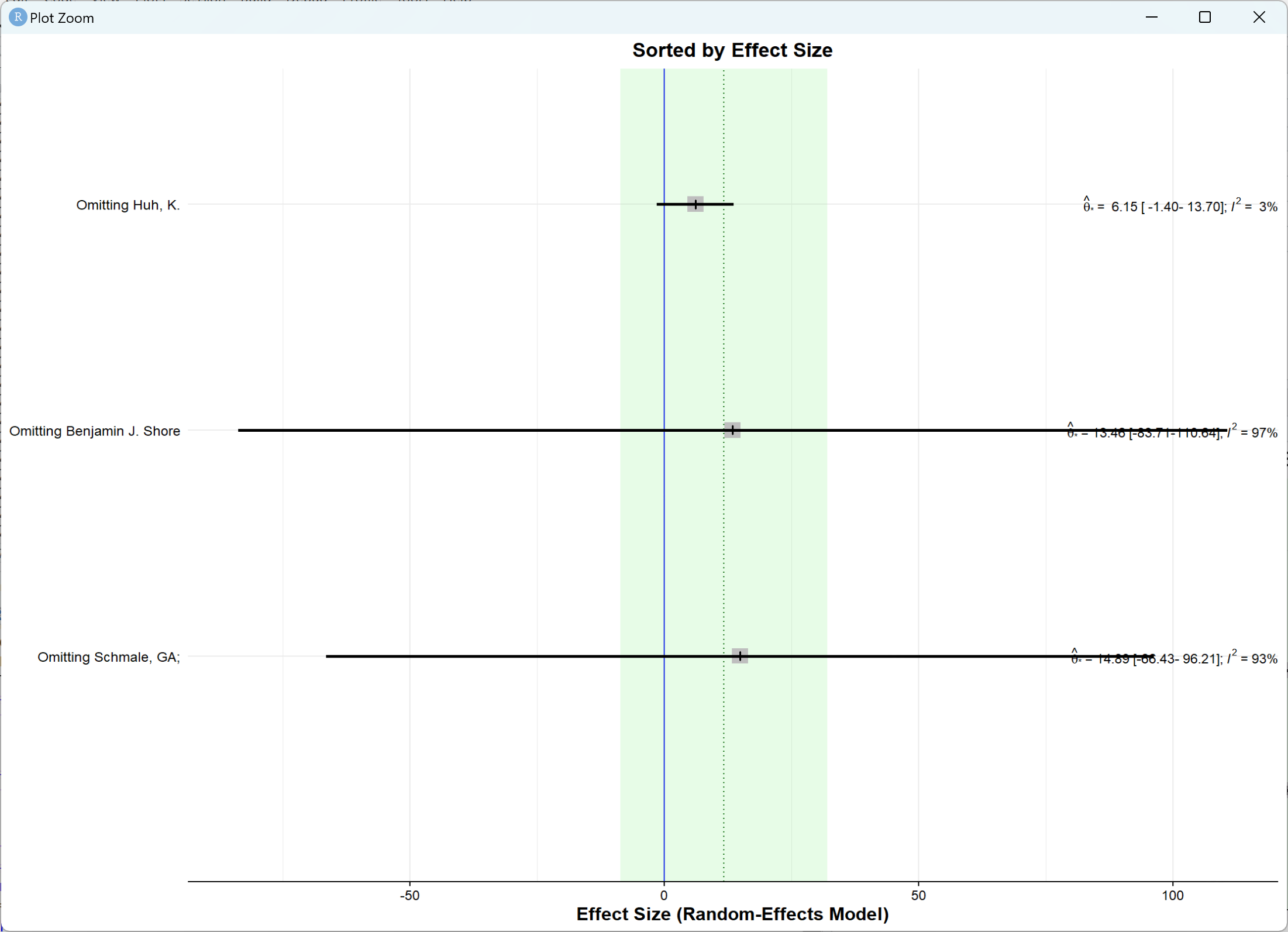


Supplementary Figure 74. Femur Osteotomy, Center Edge Angle


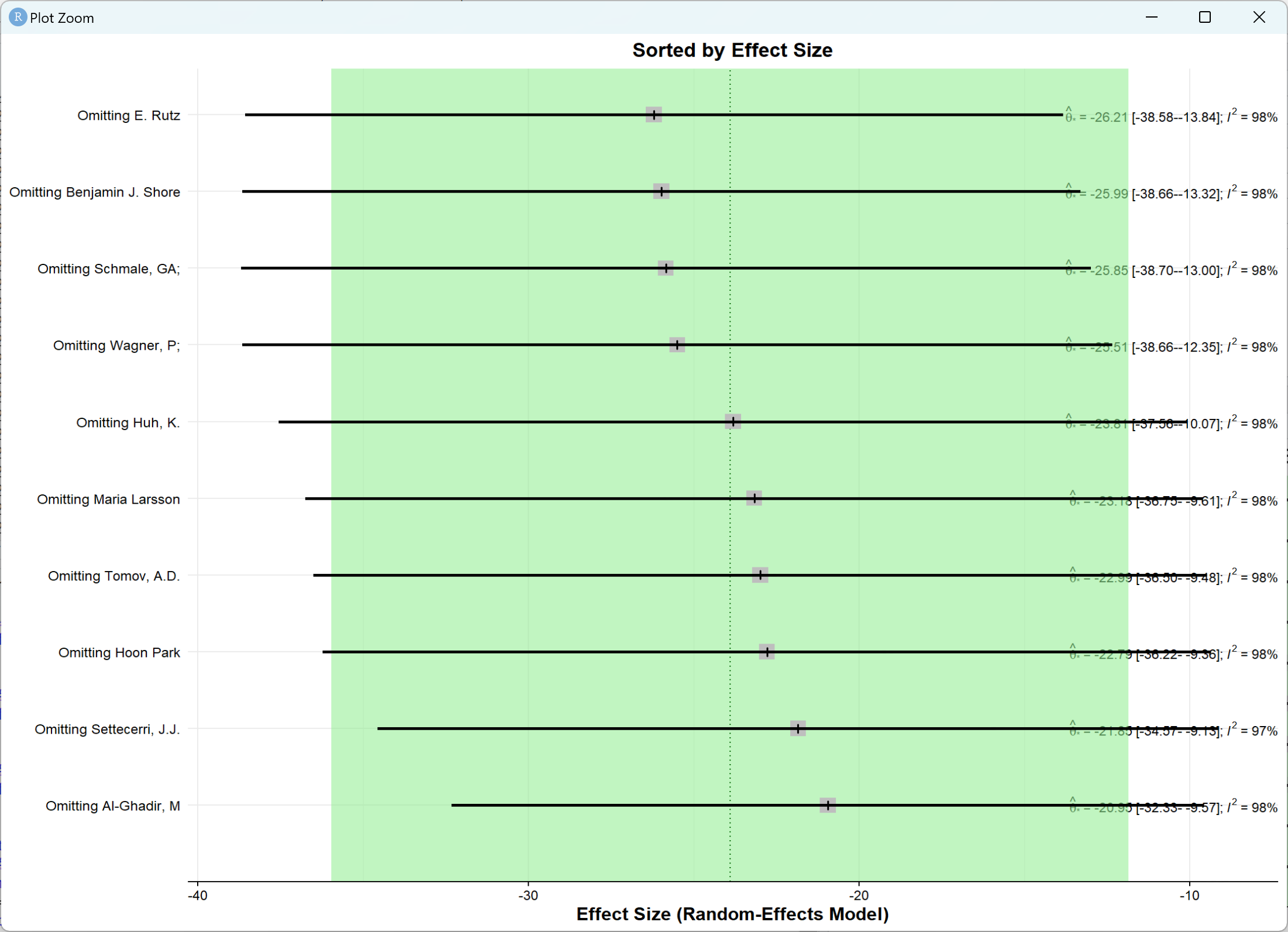


Supplementary Figure 75. Femur Osteotomy, Migration Percentage


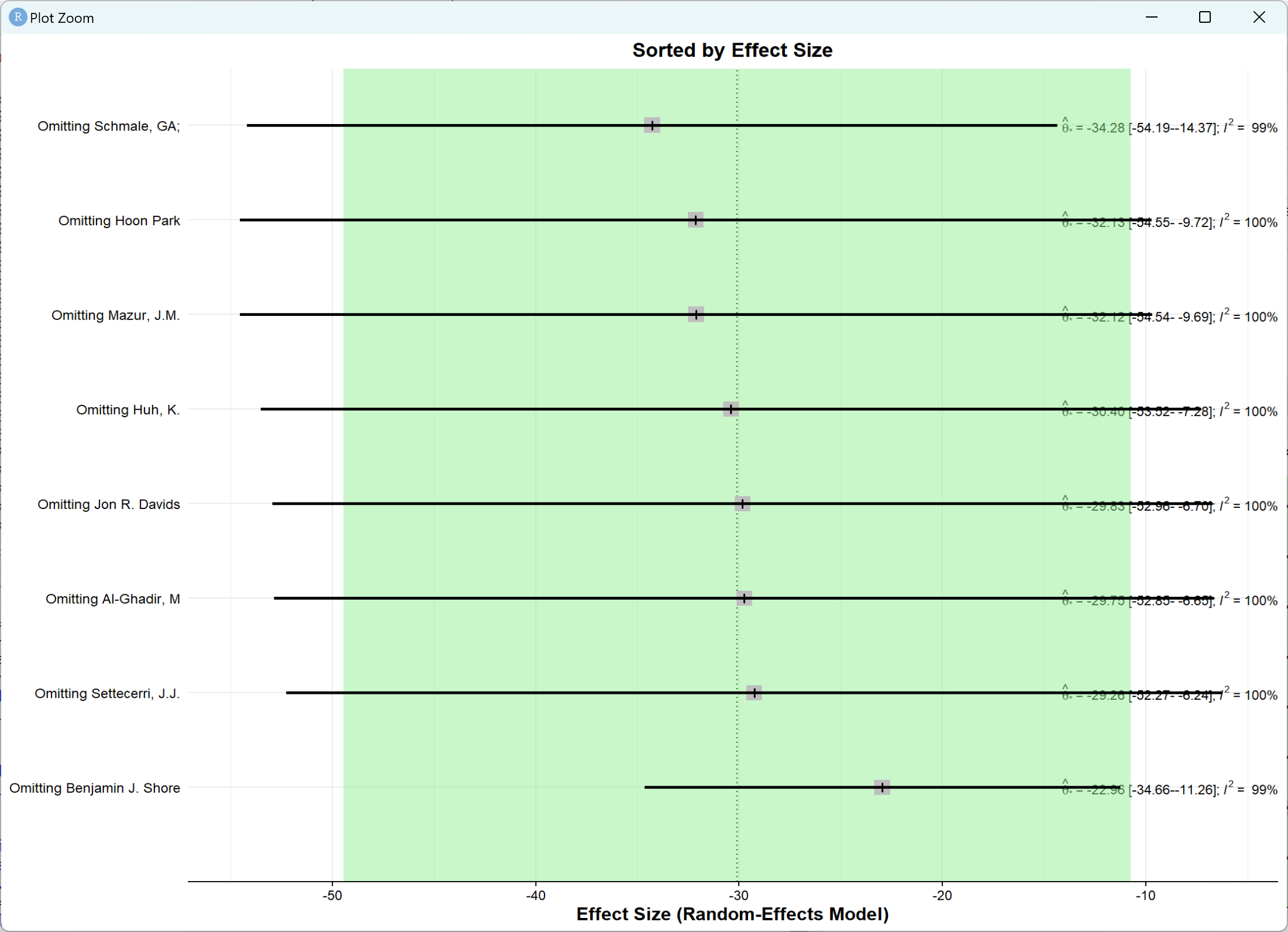


Supplementary Figure 76. Femur Osteotomy, Neck Shaft Angle


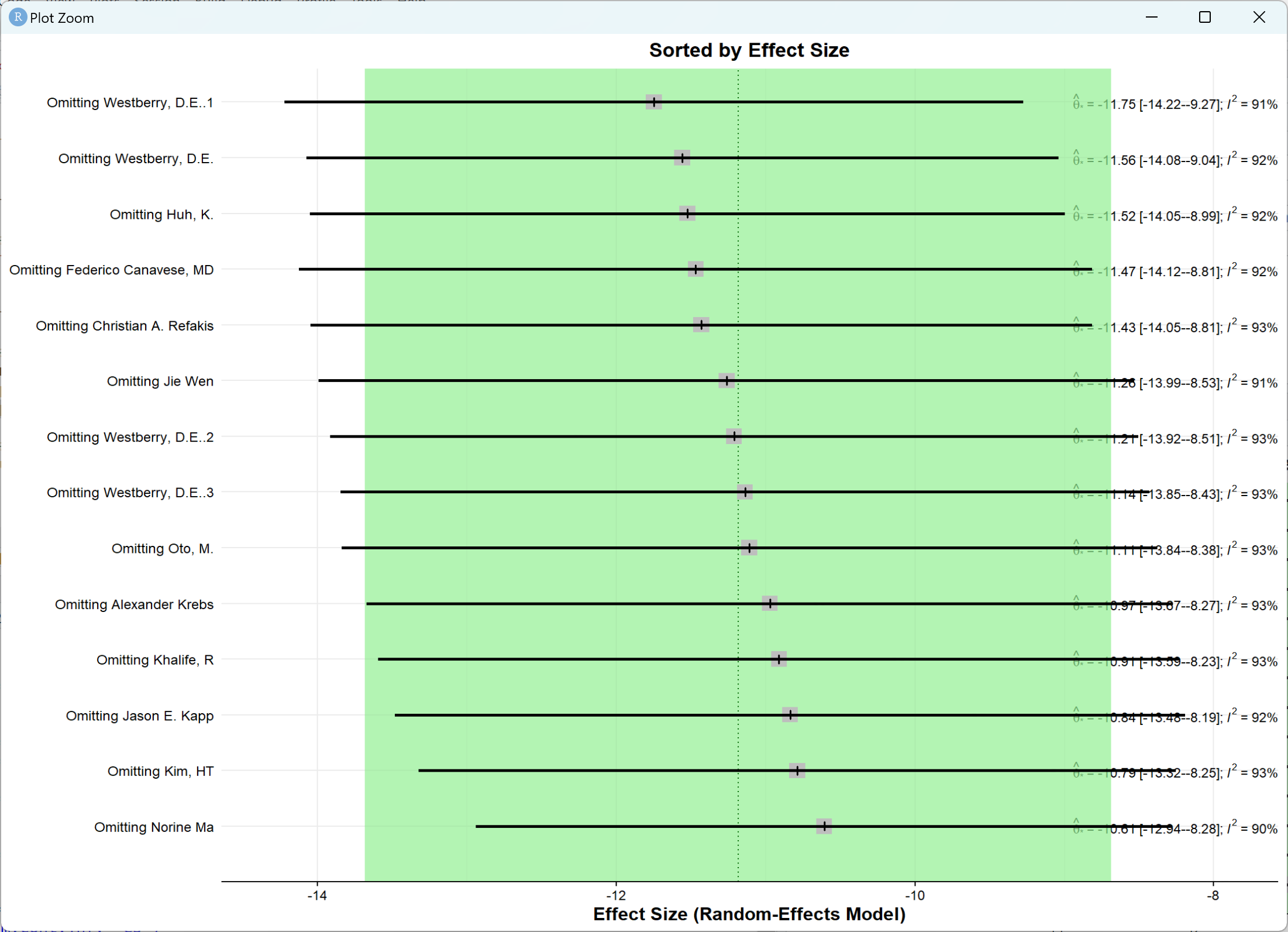


Supplementary Figure 77. Combination of Pelvic and Femur Osteotomy surgery, Acetabular Index


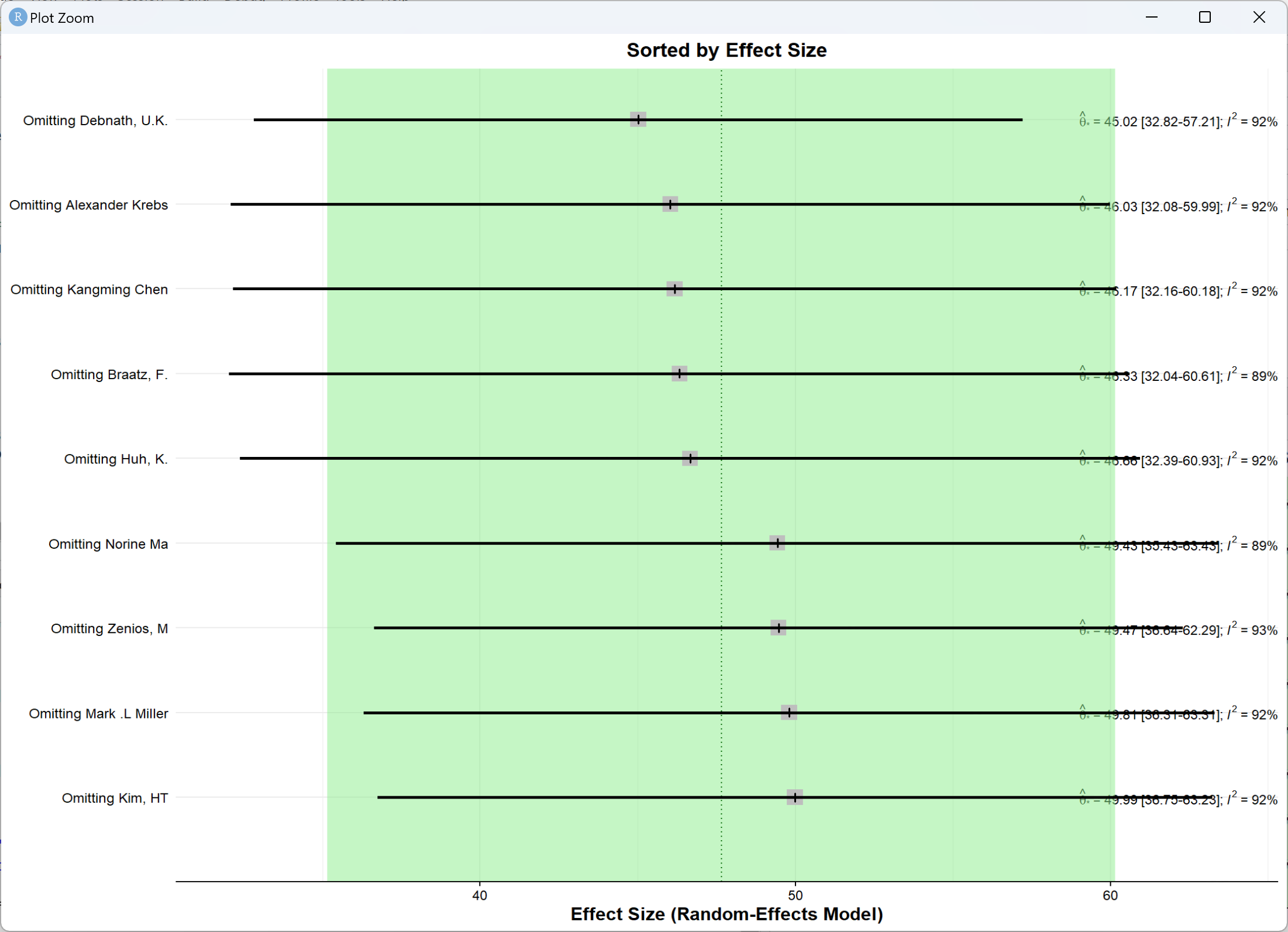


Supplementary Figure 78. Combination of Pelvic and Femur Osteotomy surgery, Center Edge Angle


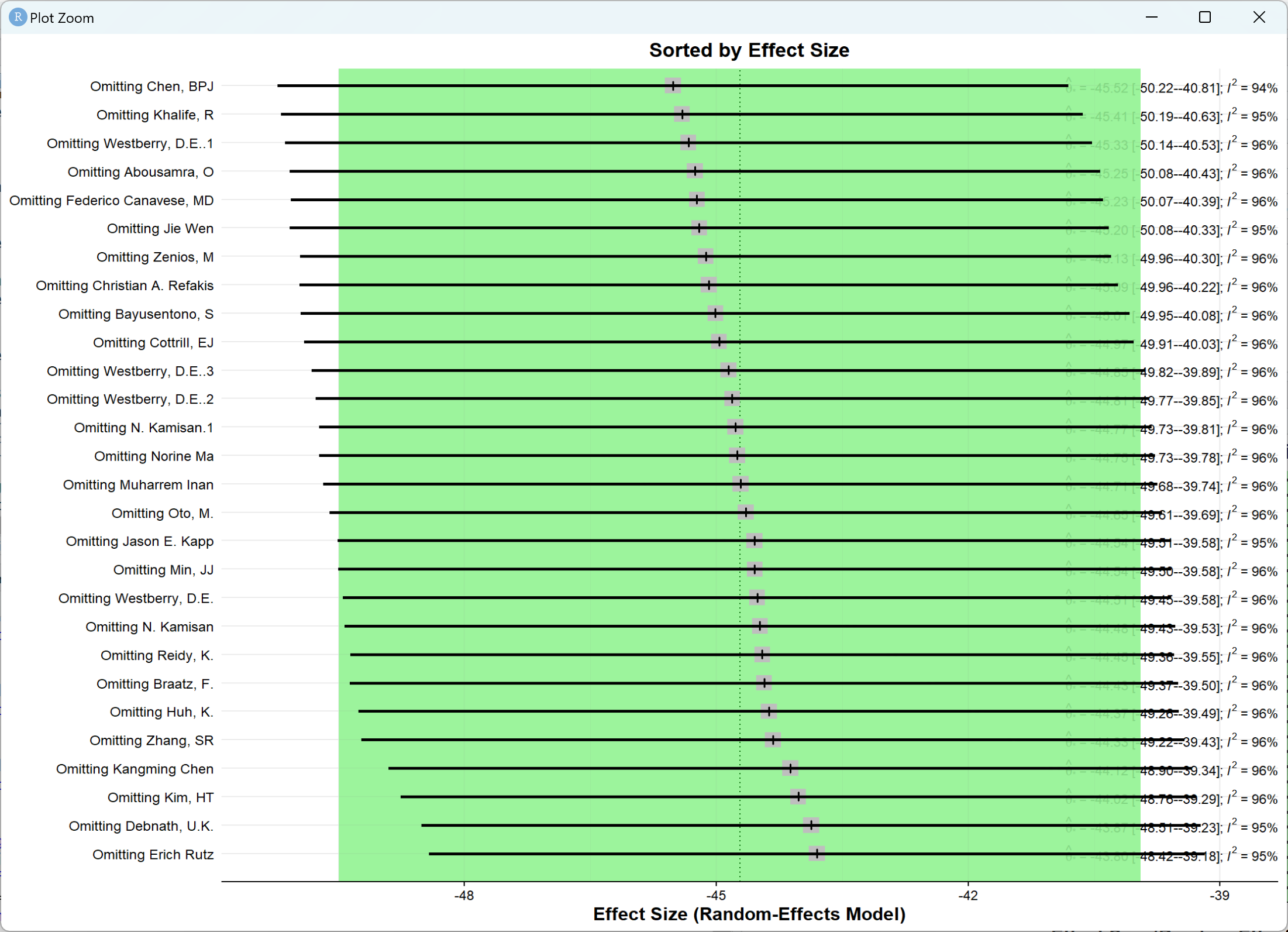


Supplementary Figure 79. Combination of Pelvic and Femur Osteotomy surgery, Migration Percentage


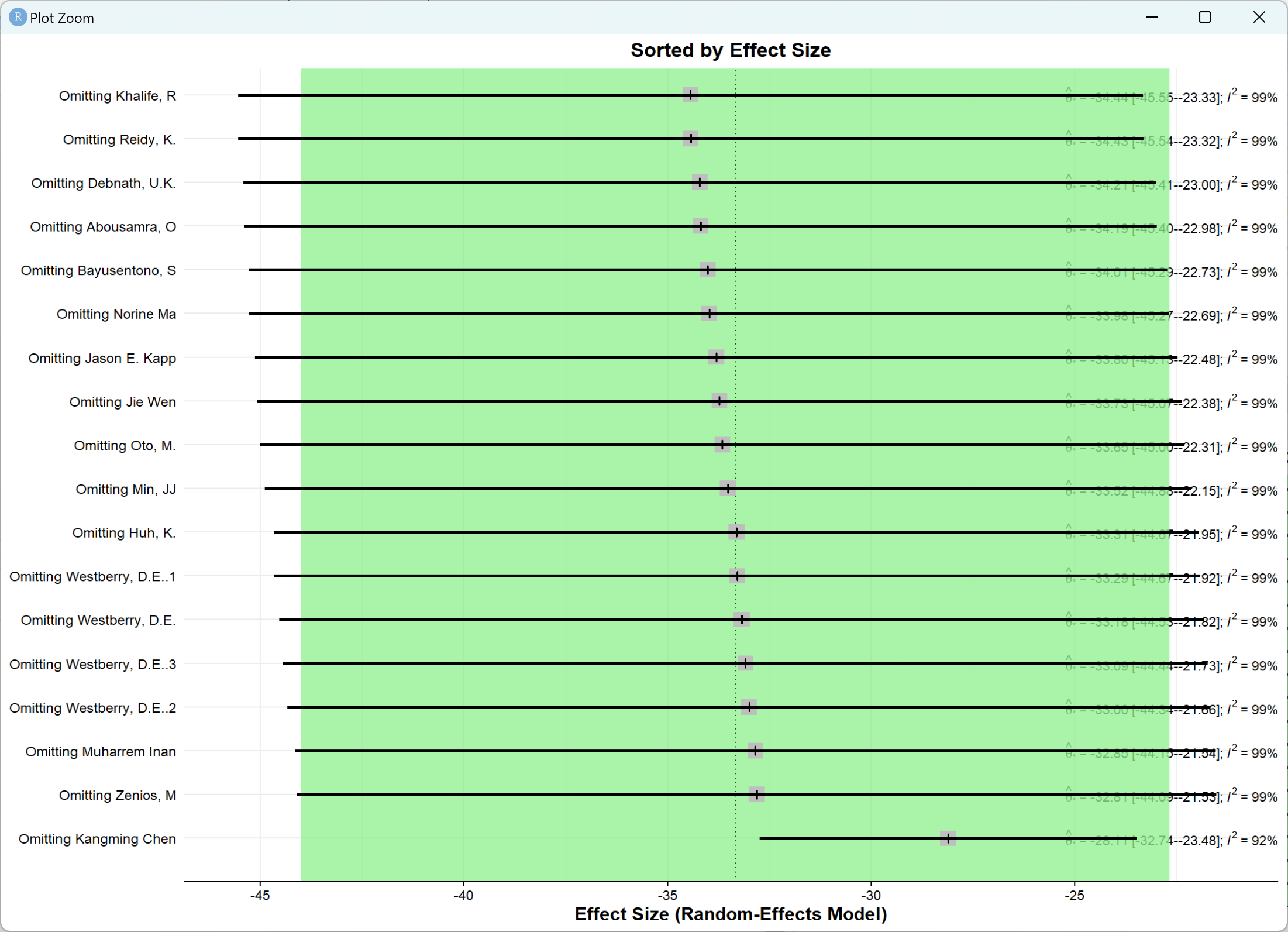


Supplementary Figure 80. Combination of Pelvic and Femur Osteotomy surgery, Neck Shaft Angle


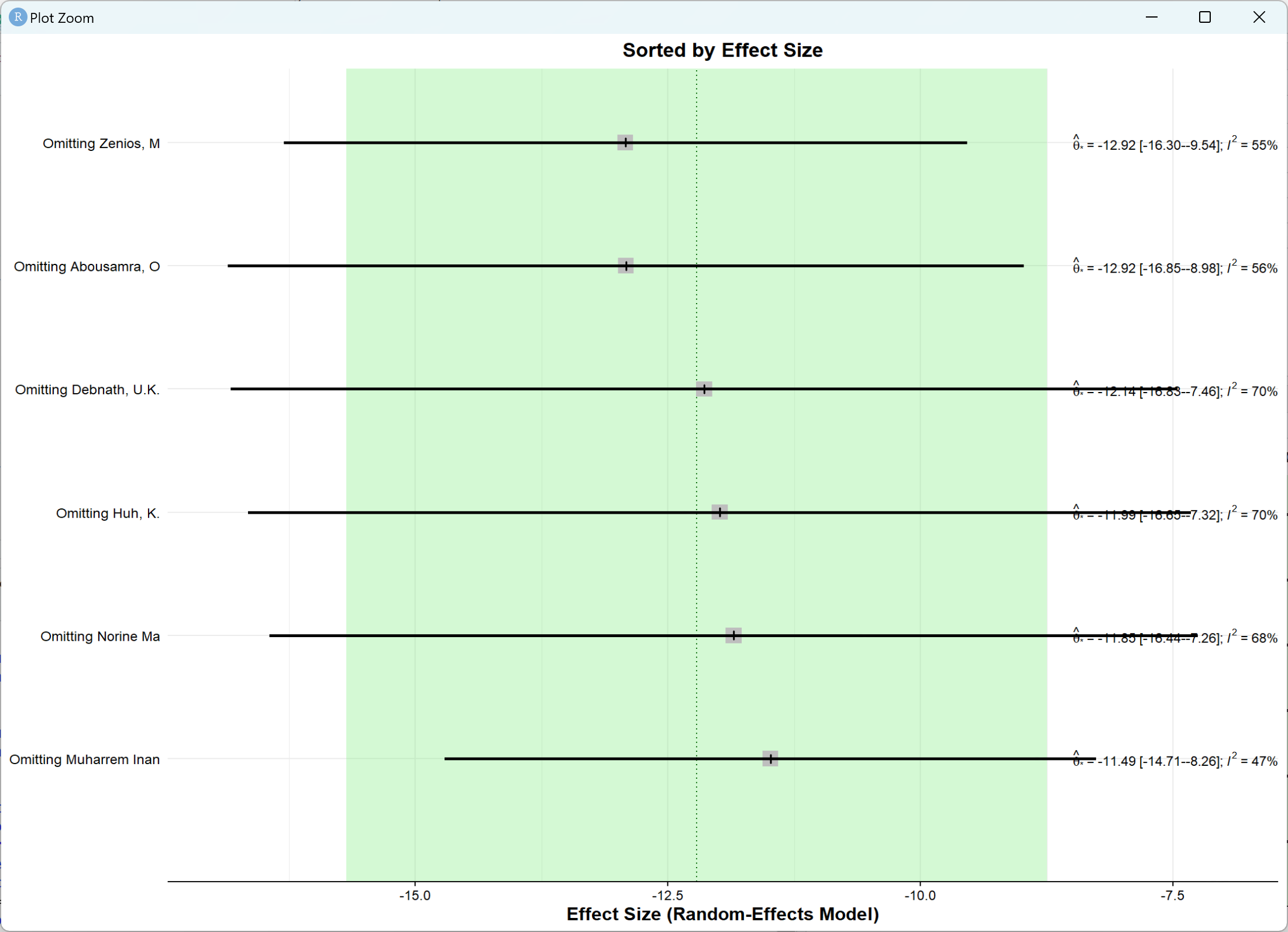


Supplementary Figure 81. Combination of Pelvic and Femur Osteotomy surgery, Sharp Angle


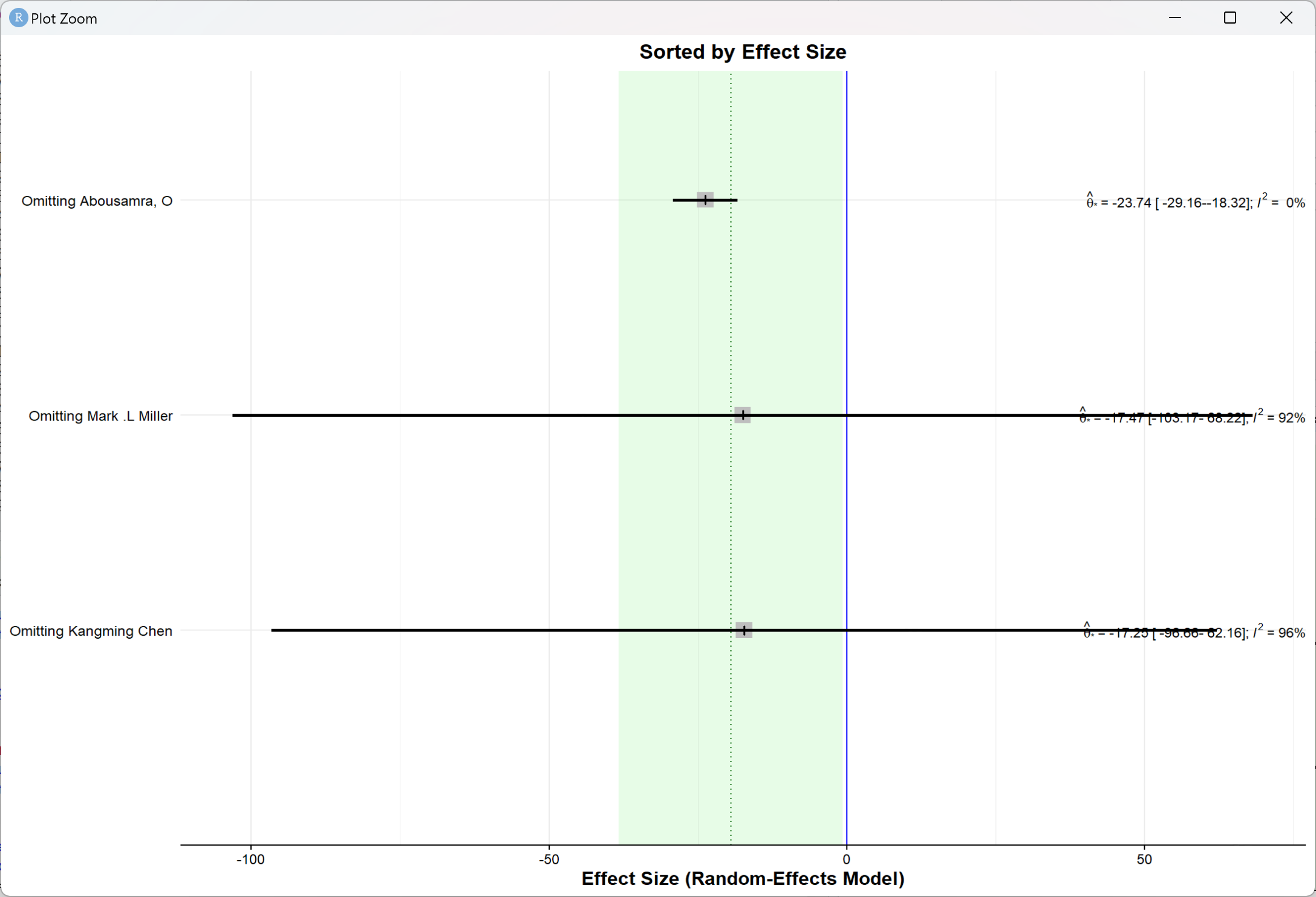


Supplementary Figure 82. Combination of Pelvic and Femur Osteotomy surgery, Tonnis Angle


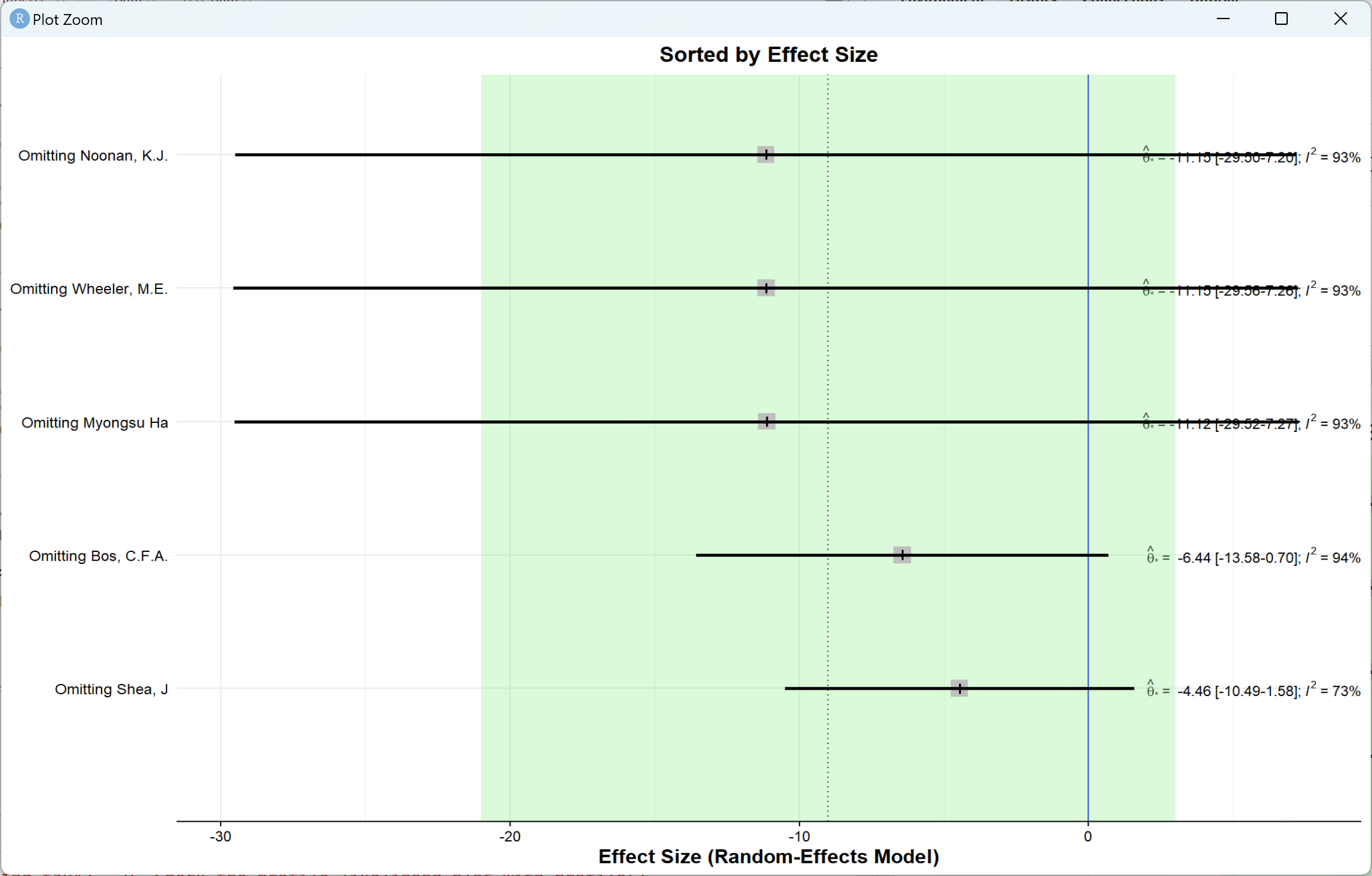


Supplementary Figure 83. Soft Tissue surgery, Acetabular Index


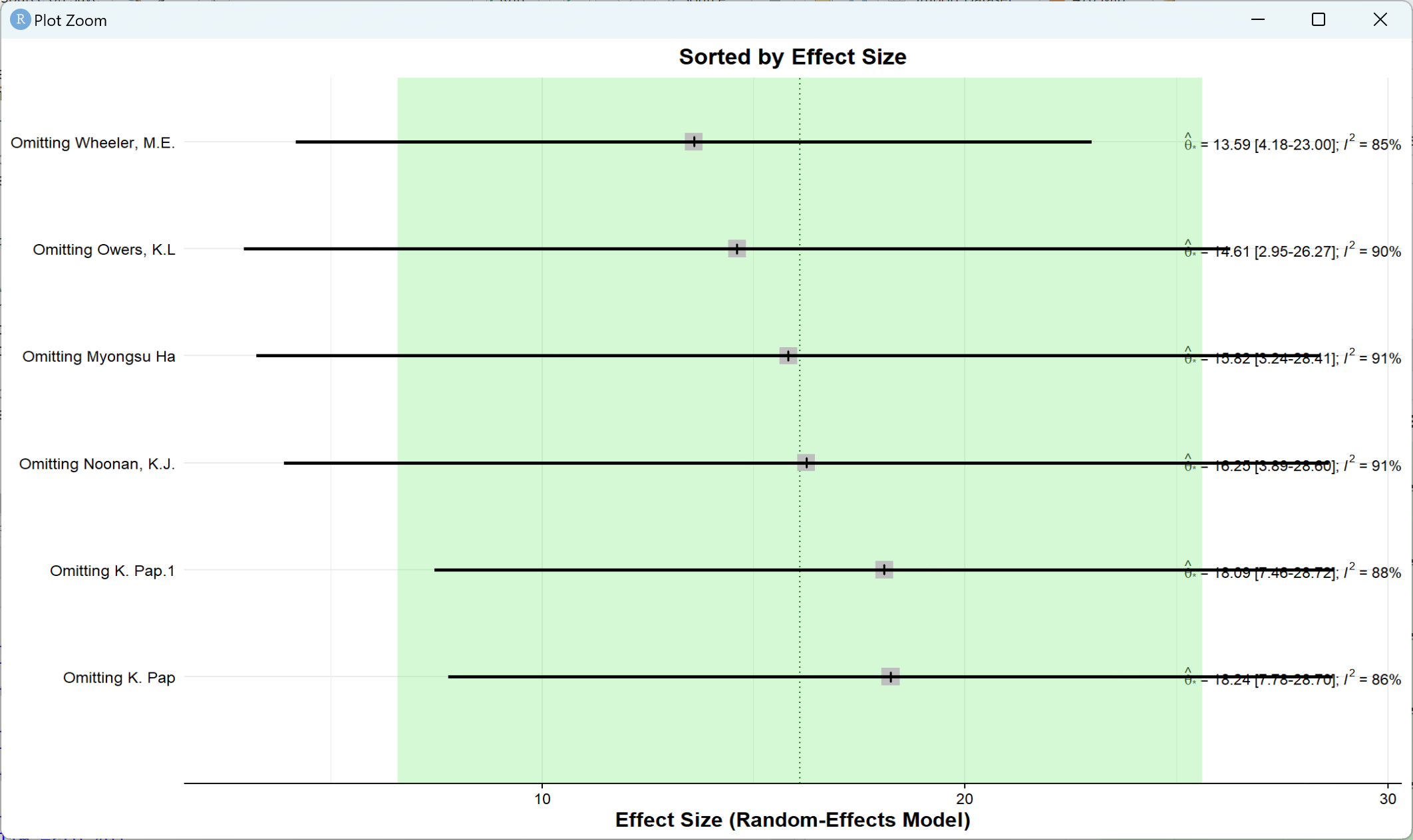


Supplementary Figure 84. Soft Tissue surgery, Center Edge Angle


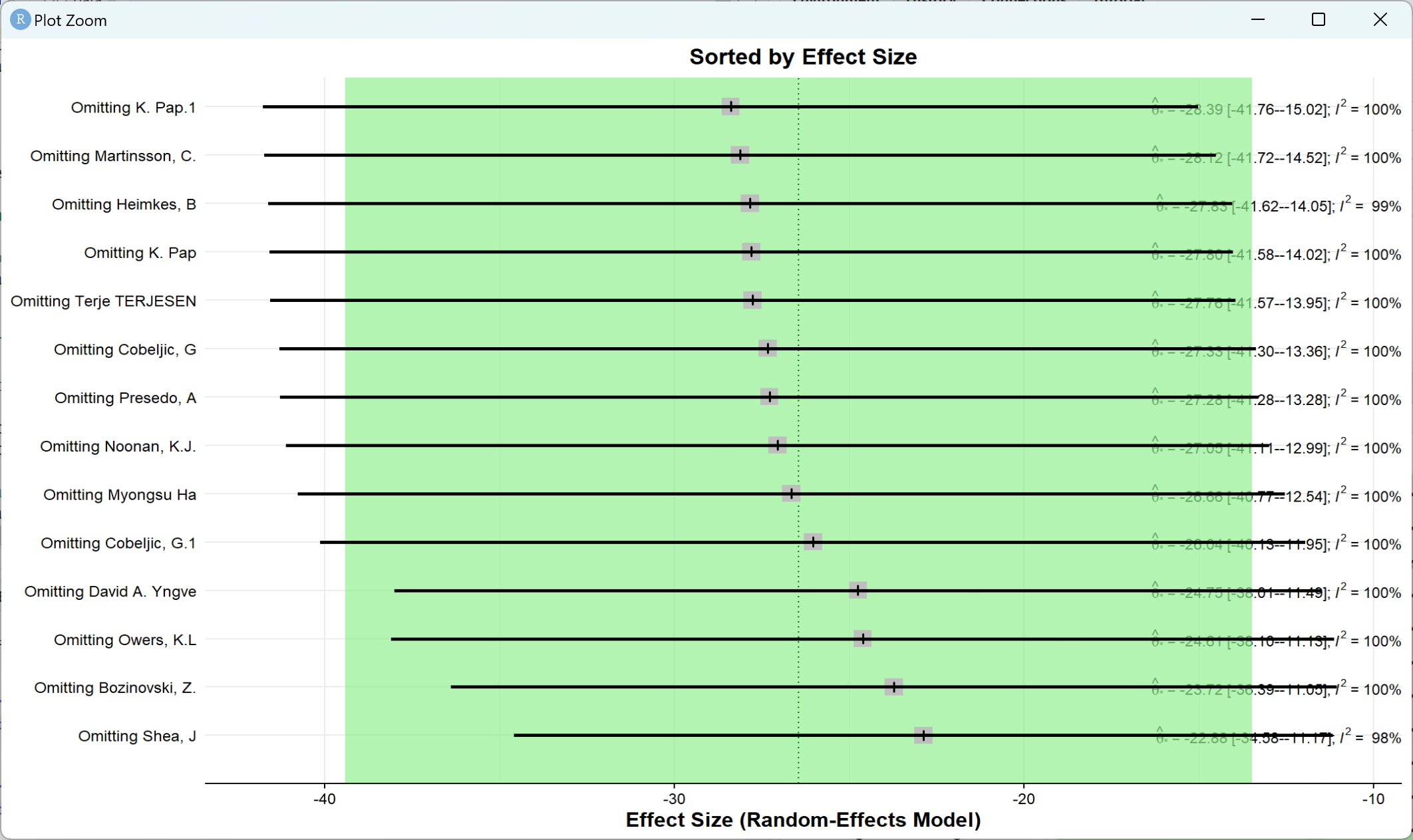


Supplementary Figure 85. Soft Tissue Surgery, Migration Percentage


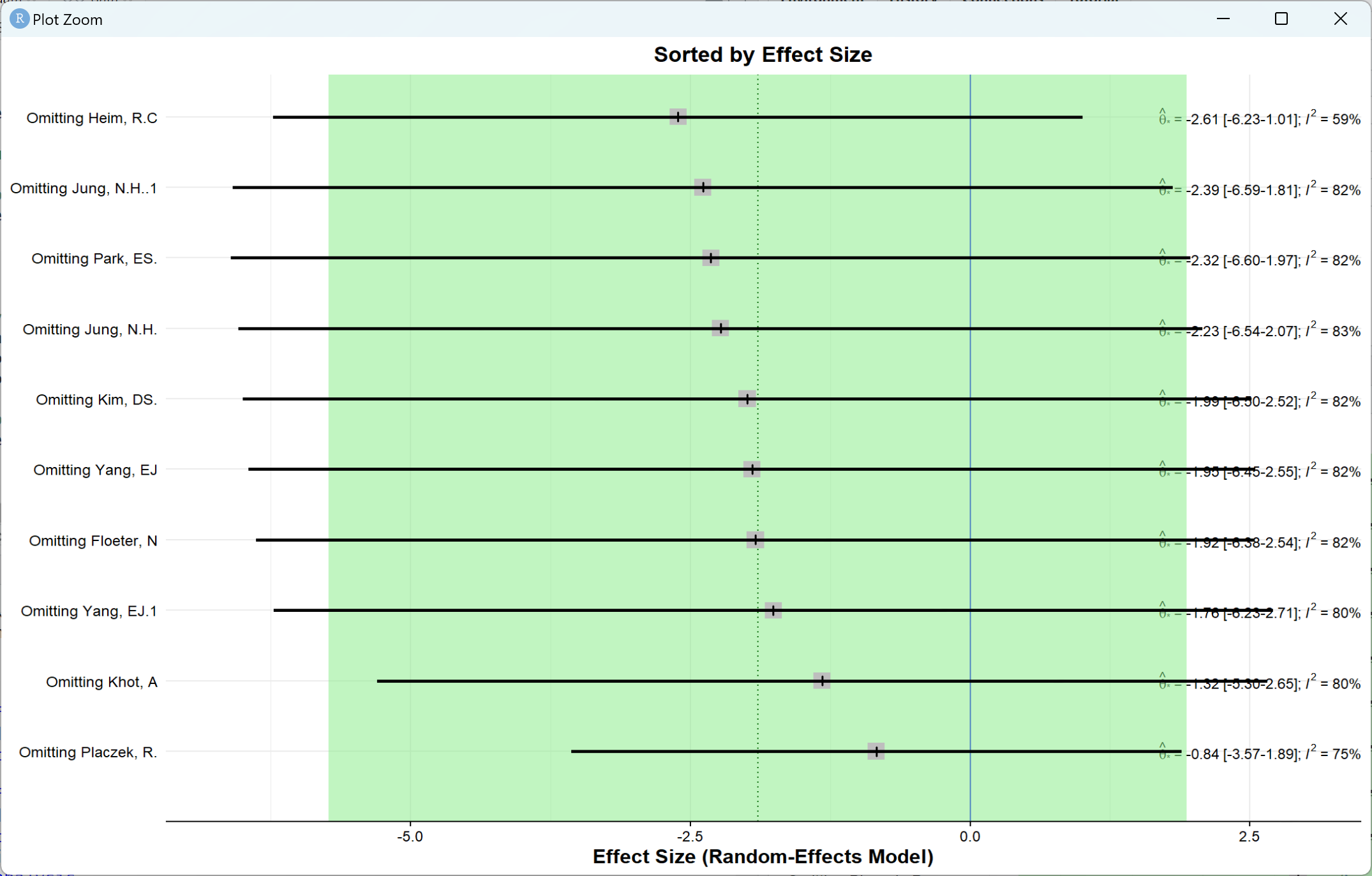


Supplementary Figure 86. Tone Decrease surgery, Migration Percentage


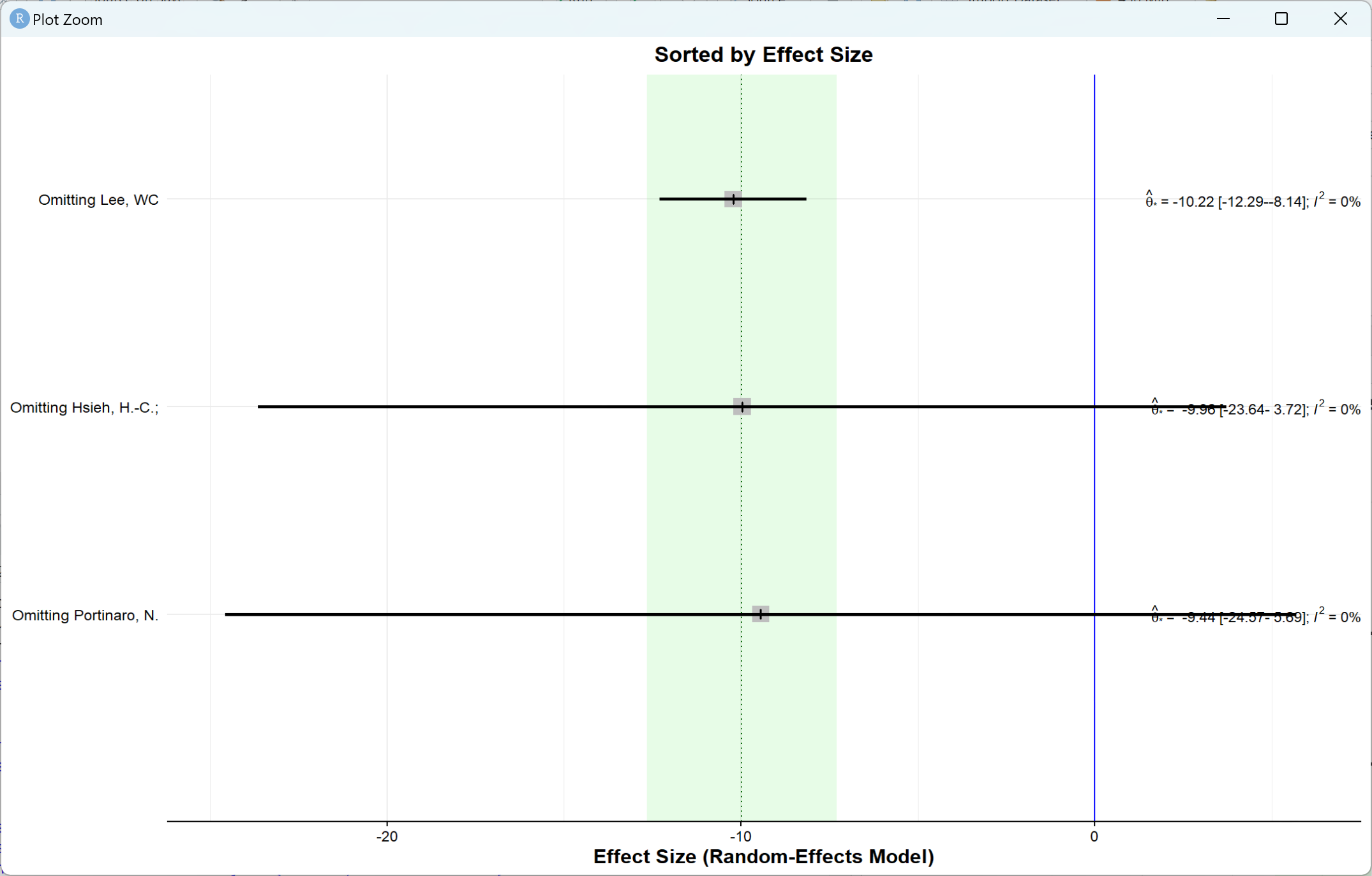


Supplementary Figure 87. Guided growth surgery, Migration Percentage


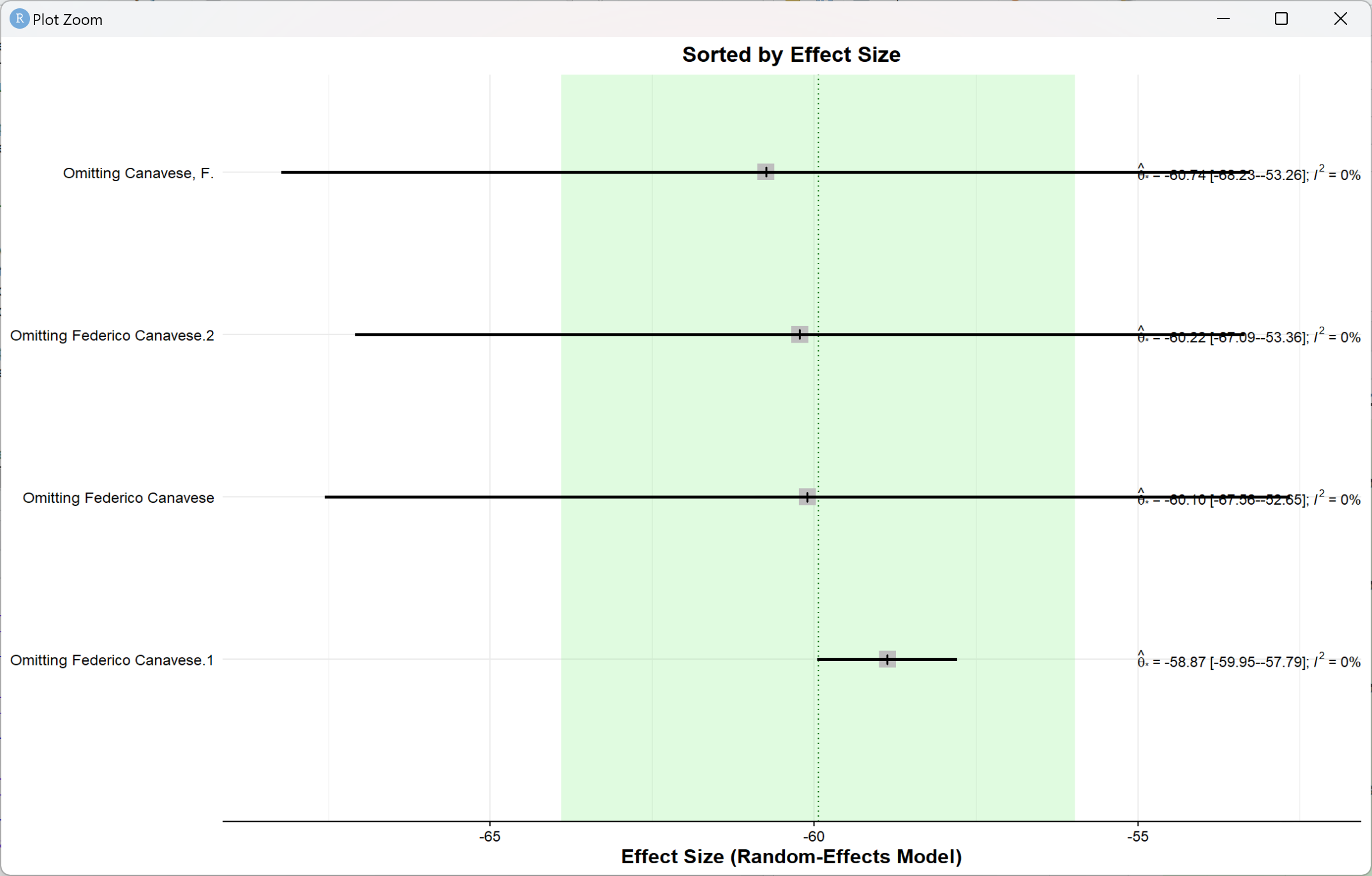


Supplementary Figure 88. Percutaneous Pelvic Osteotomy, Migration Percentage

**Appendix F ( Bias assessment)**


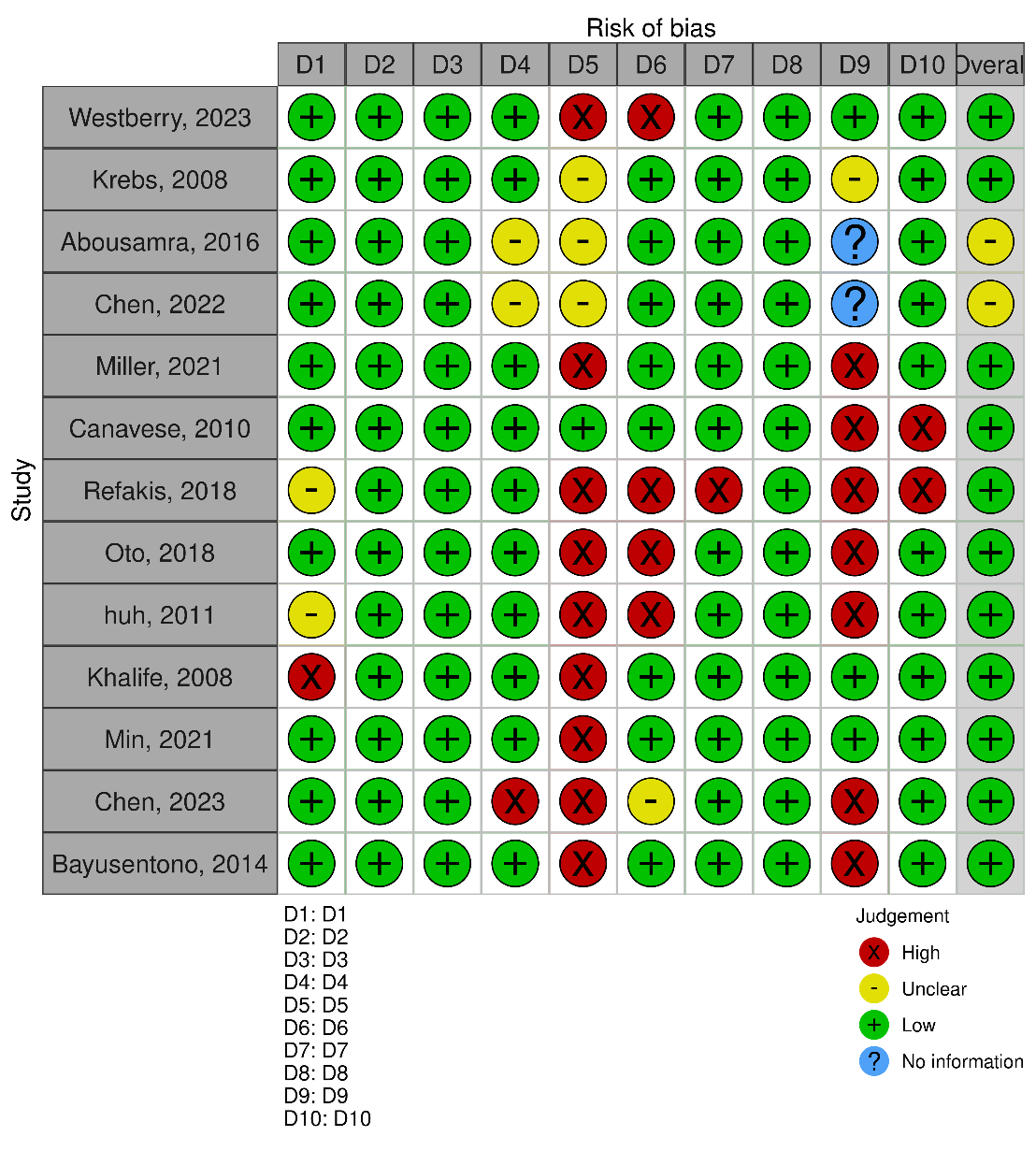


Supplementary figure 89. Combination of Femur Osteotomy and pelvic osteotomy surgery, Case series studies


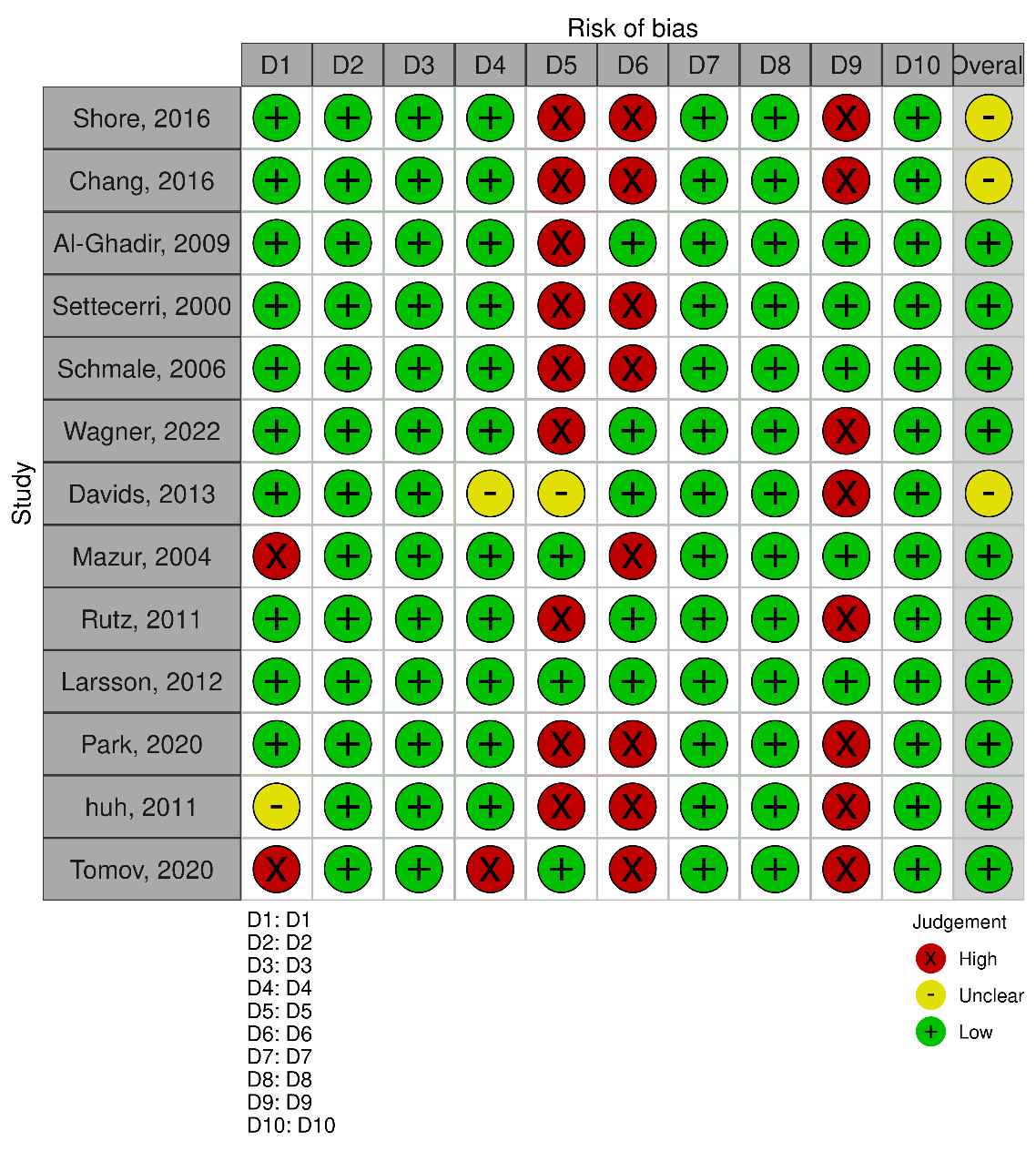


Supplementary figure 90. Femur Osteotomy Surgery, Case series studies


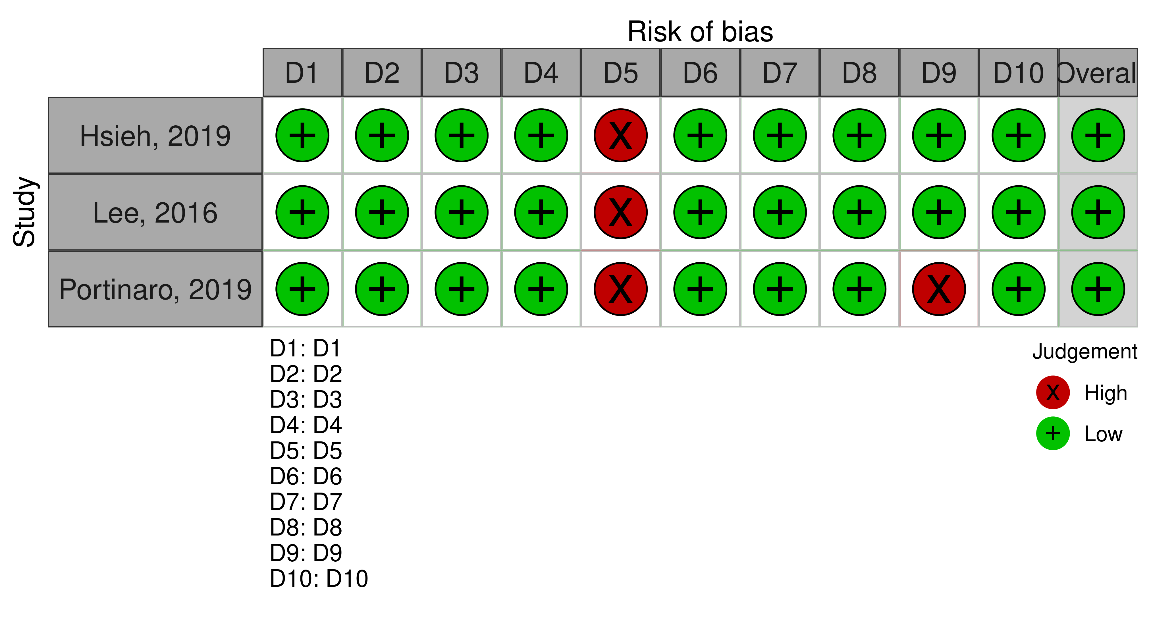


Supplementary Figure 91. Guided growth surgery, Case series studies


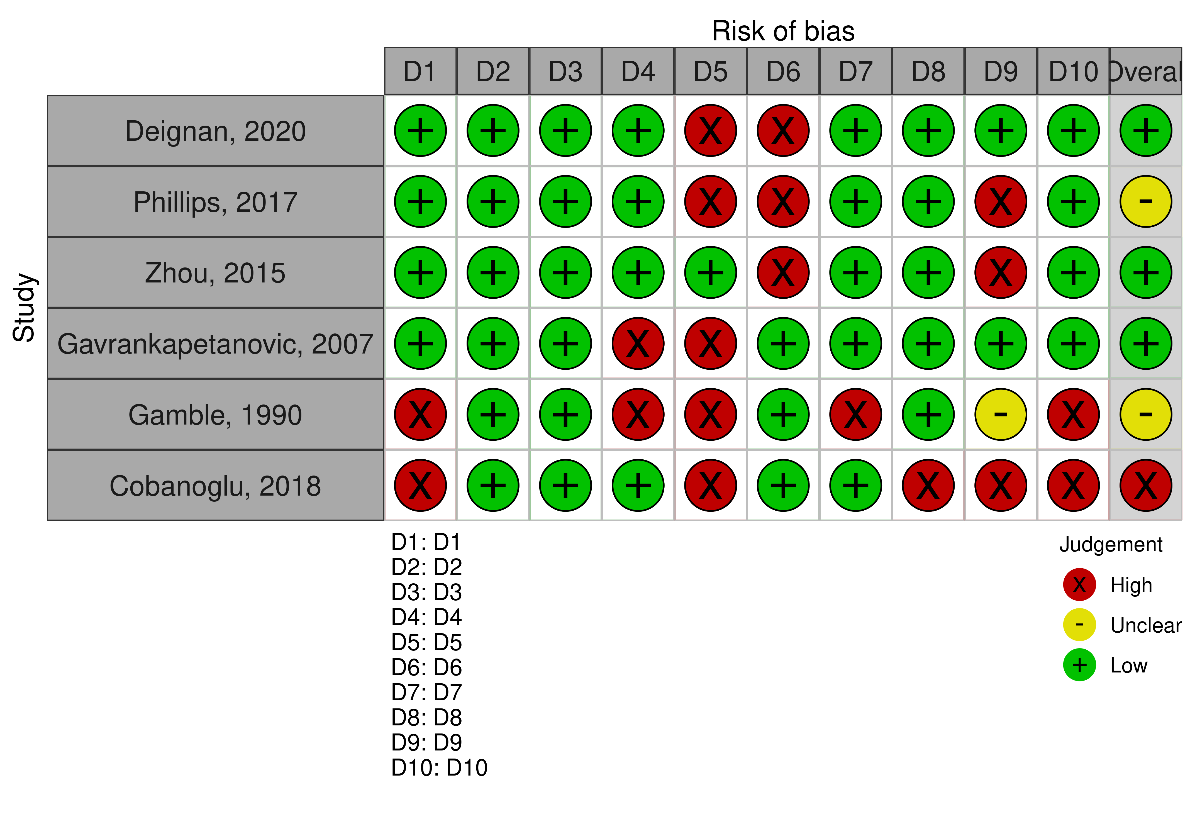


Supplementary Figure 92. Open reduction surgery, Case series studies


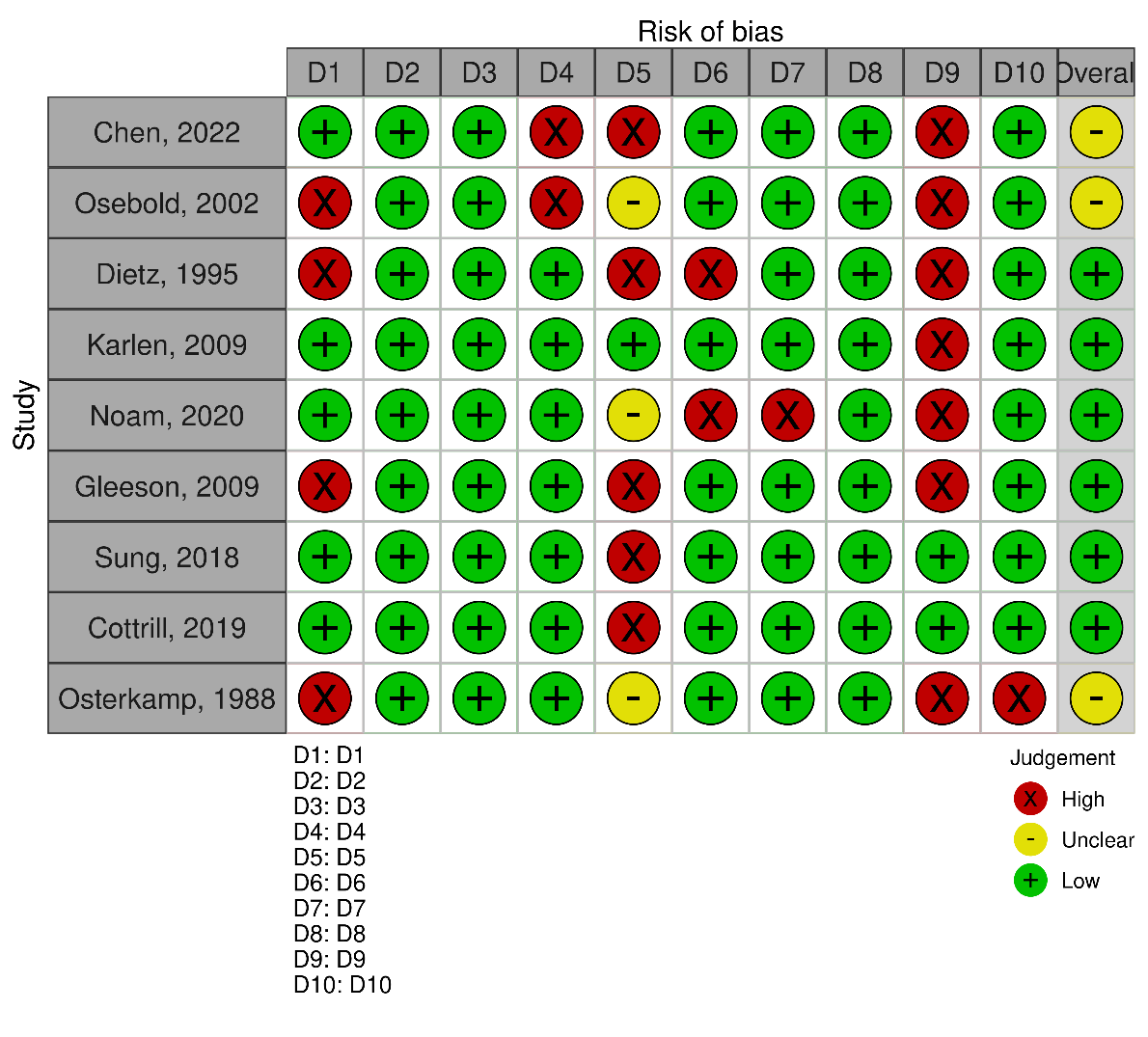


Supplementary Figure 93. Pelvic Osteotomy surgery, Case series studies


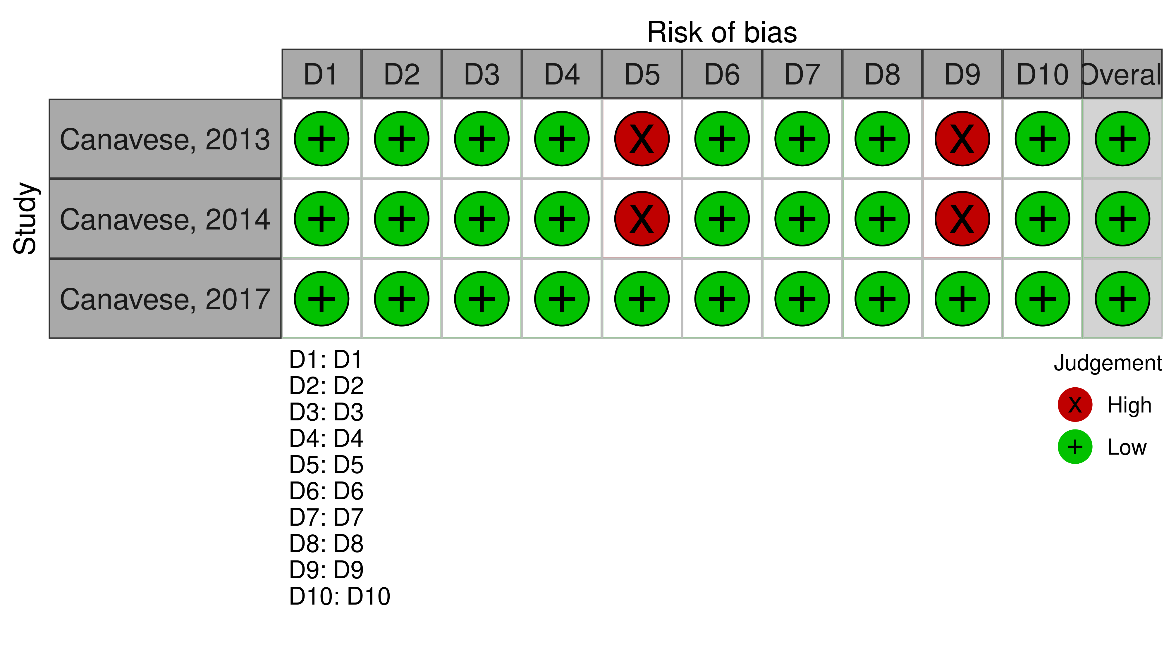


Supplementary Figure 94. Percutaneous Pelvic Osteotomy, Case series studies


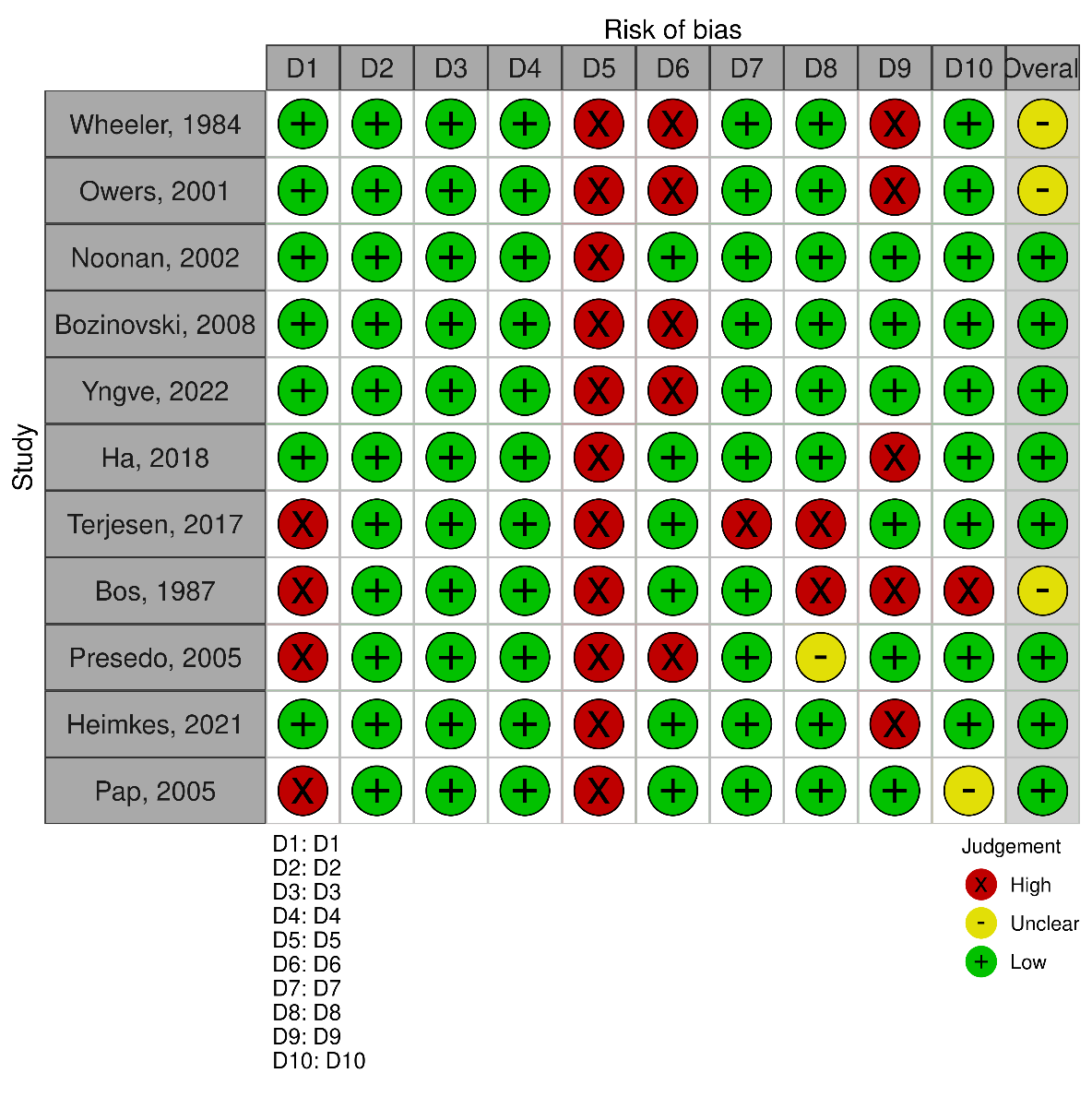


Supplementary Figure 95. Soft tissue surgery, Case series studies


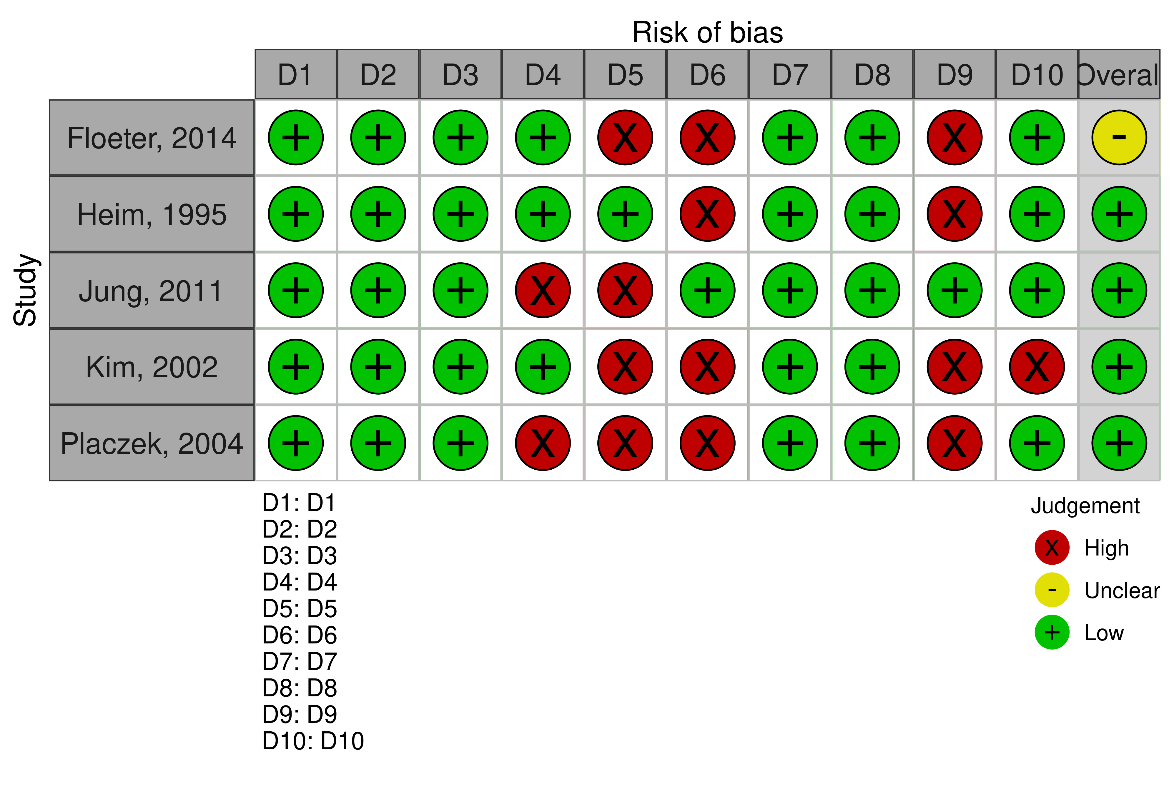


Supplementary Figure 96. Tone decrease surgery, Case series studies


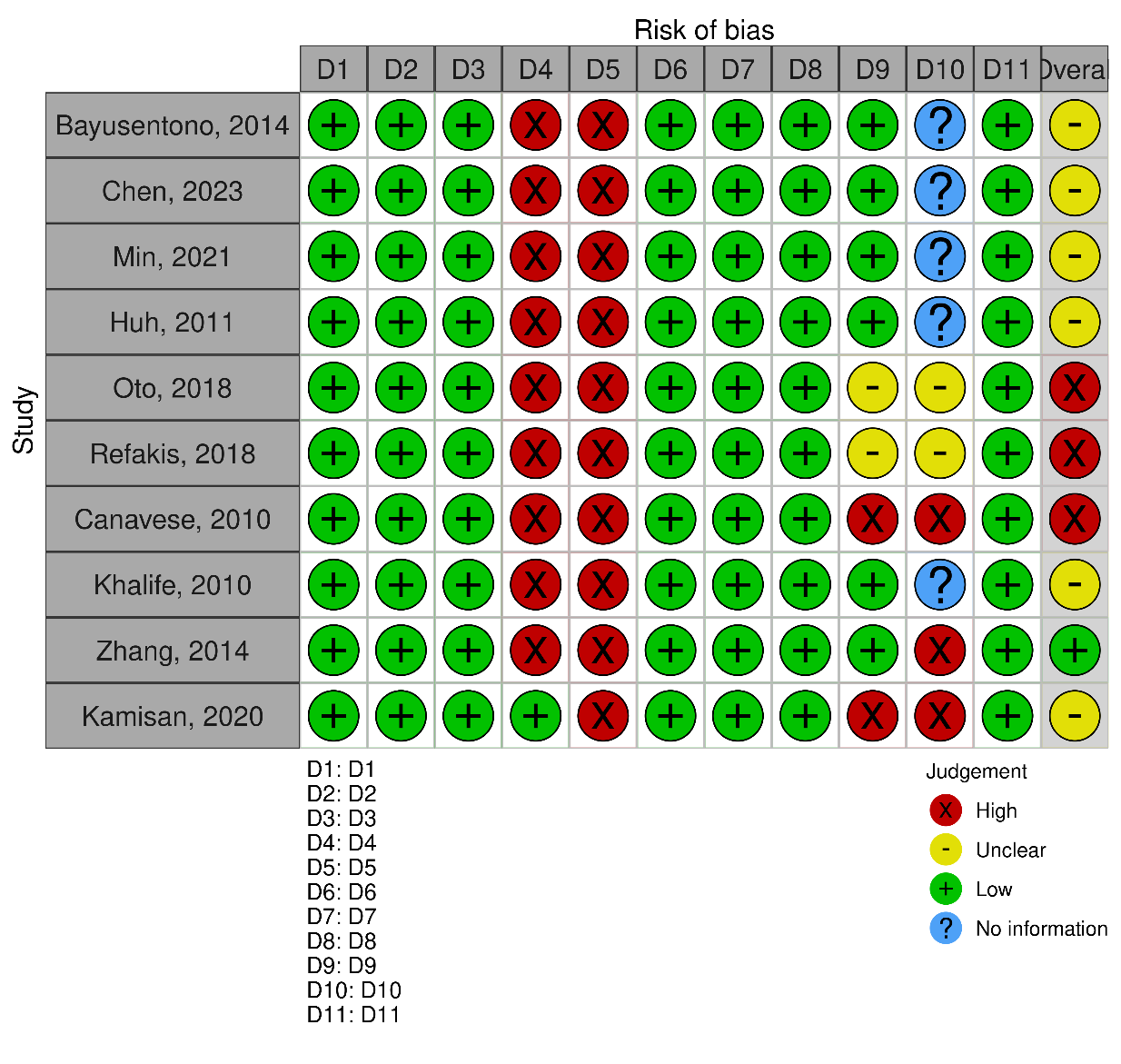


Supplementary Figure 97. Combination of Femur Osteotomy and pelvic osteotomy surgery, Cohort studies


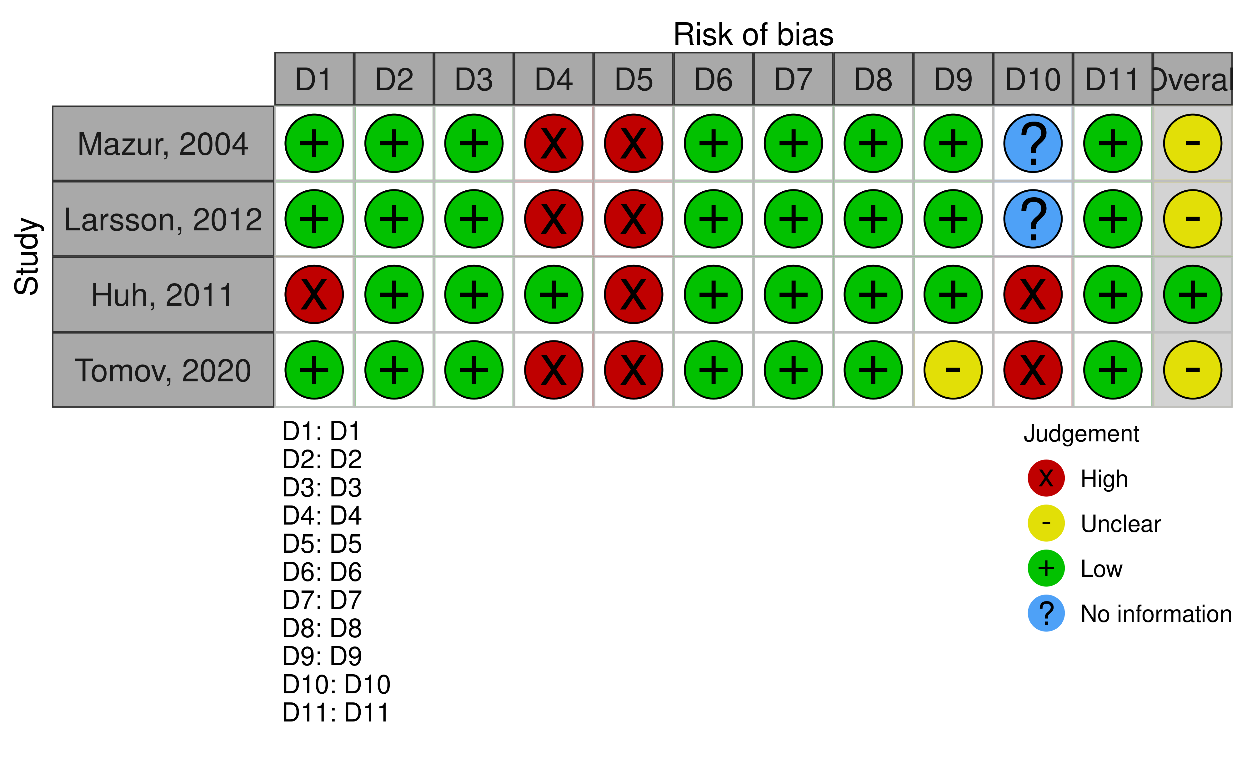


Supplementary Figure 98. Femur Osteotomy surgery, Cohort studies


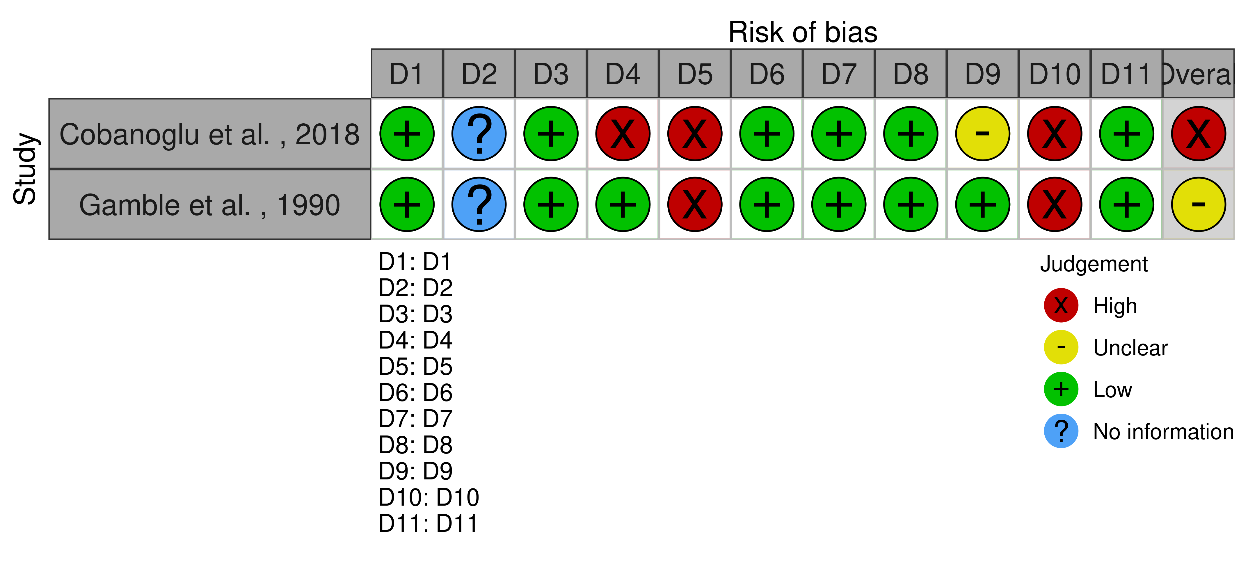


Supplementary Figure 99. Open reduction surgery, Cohort studies


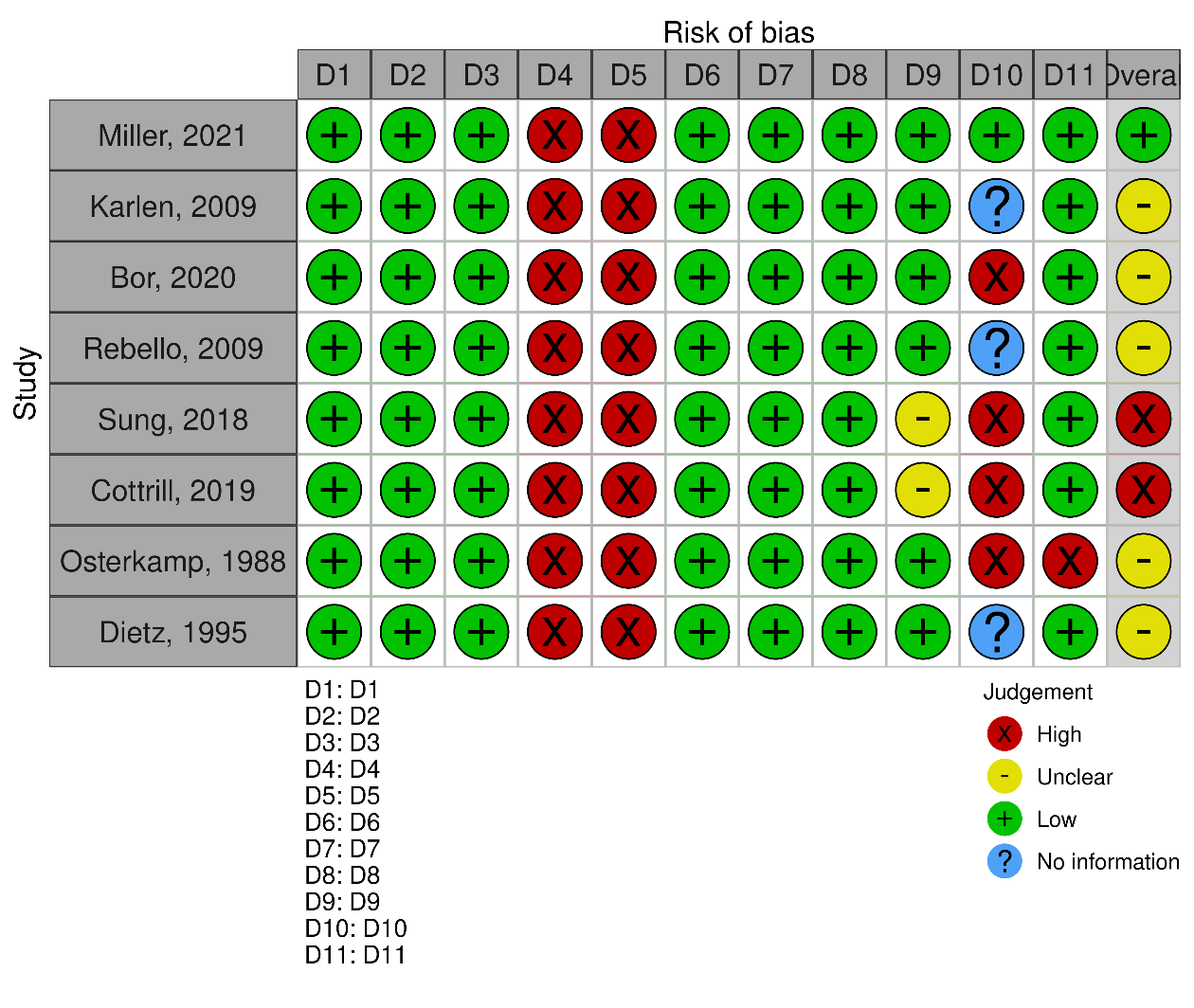


Supplementary Figure 100. Pelvic osteotomy surgery, Cohort studies

Supplementary Figure 101. Soft tissue surgery, Cohort studies

Supplementary Figure 102. Tone decrease surgery, Cohort studies

Supplementary Figure 103. Tone decrease surgery, RCT studies

**Appendix G (Subgroup Analysis)**

Supplementary Figure 104. Tone decrease surgery, Migration Percentage

Supplementary Figure 105. Soft tissue surgery, Migration Percentage

Supplementary Figure 106. Femoral Osteotomy, Migration Percentage

Supplementary Figure 107. Combined Pelvic and Femoral Osteotomy, Acetabular Index

Supplementary Figure 108. Combined Pelvic and Femoral Osteotomy, Center Edge Angle

Supplementary Figure 109. Combined Pelvic and Femoral Osteotomy, Migration Percentage
